# Solvent-assisted running-in strategy enables improved triboelectric nanogenerator output

**DOI:** 10.1038/s41467-026-74269-5

**Published:** 2026-06-11

**Authors:** Jun Zhao, Bin Ge, Xiangyu Feng, Yanjie Nie, Pengzhe Zhu, Yajie Zhang, Wei Wu, Xueyuan Li, Yongfeng Wang, Yijun Shi

**Affiliations:** 1 School of Mechanical Engineering, Beijing Institute of Technology, Beijing, P. R. China; 2 Key Laboratory for the Physics and Chemistry of Nanodevices, School of Electronics, Peking University, Beijing, P. R. China; 3 Division of Machine Elements, Luleå University of Technology, Luleå, Sweden

**Keywords:** Devices for energy harvesting, Mechanical engineering, Electrical and electronic engineering

## Abstract

The challenge of low electric outputs of triboelectric nanogenerators limits their large-scale practical applications. Although considerable efforts have been focused on improving the output, e.g., enhancing the surface charge densities of tribo-materials, the improvements are either too weak or too complicated. Here, we show that optimizing the running-in process with dimethyl sulfoxide solvent can increase the charge density of polyimide-based triboelectric nanogenerators to 2.5 mC m^−2^, a fourfold enhancement compared with untreated devices. The solvent-assisted running-in process removes the worn radicals, debris, or transferred materials on the contact surface, reducing electron transfer hindrance issues. Through time-of-flight secondary ion mass spectrometry, molecular dynamics simulations and density functional theory analyses at the molecular and electronic levels, the results indicate that running-in friction further induces the breakage of the N–C bonds in polyimide, resulting in the freedom to release amide groups. Together with the function of dimethyl sulfoxide-driven extraction, the amide-containing chains rearrange into “molecular brushes” towards contact surfaces, among which the highly electron-withdrawing C = O bonds are thus exposed and capture electrons from the counter tribolayer. This solvent-assisted running-in strategy improves electrical output in engineering polymers without material modification and clarifies how tribological running-in can be used to regulate triboelectric performances.

## Abstract (web summary)

The study demonstrates that treating polymer triboelectric devices with a solvent-assisted running-in process removes surface debris and rearranges molecular chains, leading to increased charge density and improved electrical output without altering the base material.

## Introduction

In the new era of the Internet of Things (IoT), the energy supply revolution requires clean and sustainable energy as an alternative to fossil fuels to meet the needs of Industry 4.0^1^. Thus, green and renewable energy plays a defining role in the world’s sustainable development, which is the priority trend of the entire human society. It is an attractive way to harvest environmental energy from solar, thermal, mechanical, and biochemical systems^2^. Among them, mechanical energy harvesting is one green and environmentally friendly method to cope with energy and environmental crises. Triboelectric nanogenerator (TENG), proposed and invented in 2012, is of great interest for harvesting energy from mechanical motions^3^. The triboelectric mechanism of TENGs is based on the coupling of triboelectrification and the electrostatic induction effect. With the merits of cost-effectiveness, easy fabrication, flexibility, diverse structure designs, abundant materials choices, environmental friendliness, etc., TENGs have been prepared for harvesting mechanical energy in the natural environment. In addition, TENGs are also considered a promising technology in self-powered sensors and exhibit a great advantage in IoT.

However, the challenge of low electric output of TENGs limits their applications. Considerable efforts have been focused on enhancing the output performance of TENGs, such as the methods of functionality regulation^4^, structure design^5^, charge excitation design^6^, and interface lubrication^7^, etc. For example, functionalizing radical anions onto the triboelectric material realized a maximal charge density of 1.2 mC m^−2^ with sliding-mode TENG in the open air^4^. Enhancing the charge accumulation via structure design led to a 2.3-fold increase in charge density (1.63 mC m^−2^) compared to a standard TENG under ambient conditions^5^. Under the high charge density, however, the charge decay inevitably occurs owing to the air breakdown phenomenon. It is found that in general, air breakdown is difficult to happen in an extremely low density of air molecules, but this vacuum is not common in our daily life and poses a great challenge to the packaging technology^8^. The integrated self-charge-pumping TENG was developed, which includes another layer-floating TENG as a charge pump to overcome air breakdown^6^. It means there are two TENGs, i.e., the main TENG and the pump TENG in the triboelectric system, so the structure of the self-charge-pumping TENG is very complex^6,9,10^.

The strategy of interface lubrication pushed the TENG to a new stage, in which the air breakdown is fully inhibited via filling lubricants in the air-micro-gaps between the triboelectric layers^7,11,12^, of which the advantages have been summarized in our recent work^13^. However, the triboelectric materials suffer from severe wear during the initial transient, i.e., running-in period, even under lubrication conditions^14^. Running-in period means two fresh triboelectric surfaces in relative sliding motion begin to experience intimate contact at the asperity level^15^. Thus, although interface lubrication can effectively decrease the wear of tribo-materials, it cannot address the issues during the running-in periods. In this case, untimely transitions to asperities wear, and materials transfer will cause interface charge shielding and lower electrification capability. The large, worn, and transferred materials are difficult to remove from contact surfaces. Therefore, considering running-in, it is highly desirable to develop running-in optimization strategies for maximizing the surface charge density.

Herein, we propose a solvent-assisted running-in strategy to enhance the interface lubrication of TENGs. That is, treating the tribo-materials, e.g., polyimide and aluminum (PI/Al), during running-in with a non-proton solvent. Worn free radicals, debris, or transferred materials on the contact surface can be easily removed after the running-in process. It is found that, during the running-in period, the friction force induces the breakage of the relatively unstable N–C bonds in PI, resulting in the release of amide groups. With the DMSO-driven extraction effect, the amide groups are transmitted towards contact surfaces as “molecular brushes”, and then the C=O bonds with strong electron-withdrawing capability are exposed, which significantly improves the electron affinity of PI. With an optimized selected solvent and running-in process, the charge density of PI-based TENGs (2.5 mC m^−2^) achieves a fourfold enhancement of untreated PI-based TENG in ambient conditions, exceeding the highest previously reported record by a factor of 1.53. Moreover, the output is obtained using commercially available engineering polymers without material modification, indicating a route for improving polymer-based TENGs.

## Results

### Optimizing the running-in process towards high surface charge density of TENGs

**Fig. 1:**
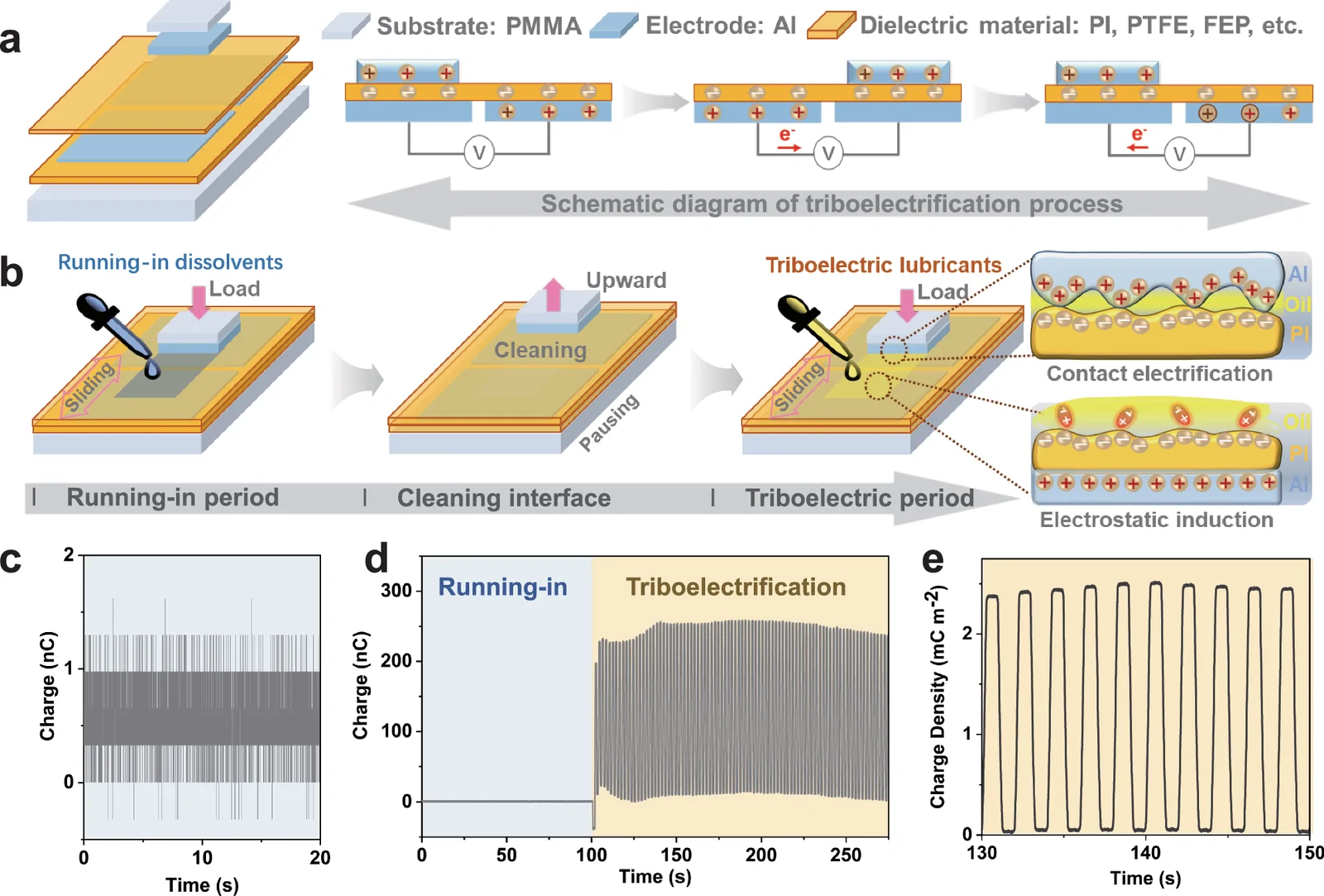
Demonstration of the TENG via optimizing the running-in process. **a** Schematic diagram of device structure and triboelectric mechanism. **b** Optimizing process for the running-in and triboelectrification. **c** Electric output during the running-in process. **d** Electric output for the triboelectric period. **e** Charge density during the triboelectric period.

In a traditional sliding TENG, material transferring from one tribo-layer to another inevitably happens during the running-in period, which causes interface charge shielding and lower charge density^13,16^. Here, we optimize the running-in process to improve the surface charge density of TENGs. Figure 1a shows the 3D structural scheme of the basic structure of the TENG. PI and Al were used as tribo-materials, which were adhered to flat square polymethyl methacrylate (PMMA) boards as the supporting substrate. The detailed fabrication process is presented in the “Methods” section. The working mechanism and the newly introduced running-in schematic diagram are shown in Fig. 1a, b. Firstly, the running-in period is operated with the lubrication of dissolvents such as dimethyl sulfoxide (DMSO), dimethylacetamide (DMA), dimethylformamide (DMF), etc. These dissolvents have the function of carrying worn free radicals and debris. Thereby, the contact surfaces are then fully cleaned after the running-in process. During the triboelectric generating period (Fig. 1b), a lubricant, e.g., hexadecane, squalane, paraffin oil, etc., is added to the sliding interface to address the air breakdown issue and to achieve high surface charge density.

Triboelectrification works on the coupled mechanism of contact electrification and electrostatic induction. Under dry conditions, electrons are usually transferred from the Al tribolayer to the PI surface during contact electrification. Electric outputs will be achieved when the Al tribolayer is sliding against the PI surfaces due to electrostatic induction. For the case of lubrication, a thin liquid film (normally less than several tens of nanometers) will be formed at the interface^7^. Due to the presence of roughness on contact surfaces, some small liquid pockets will be generated. When an external force is applied, the liquid film ruptures at certain asperities, and solid–solid contact becomes dominant at the electrical interface (Fig. 1b), which means the contact electrification occurs between the Al tribolayers and the PI surface. In contrast to dry friction in air (Supplementary Fig. S1), liquid lubricants can significantly suppress interfacial breakdown due to the higher breakdown field strength requirement and lower electrostatic field strength in the microgap between the Al tribolayer and the PI surface. Further, when the charged PI surface is submerged in the lubricant, the surface charge will not be screened by the nonpolar lubricants, which have a larger Debye length than polar liquids^12^. Because of the electrostatic induction between the charged PI surface and the Al electrode, electrons will flow from one Al electrode to another electrode under short-circuit conditions for an electric equilibrium (Fig. 1a).

Figure 1c–e shows the dynamic charge output curve of the TENG operating under the designed running-in and triboelectric periods. The charge value is nearly zero during the running-in period with DMSO dissolvent due to its high relative permittivity^7^. During the triboelectric generation period with hexadecane lubrication, the charge density reaches 2.5 mC m^−2^ for as-received commercial PI. Whereas, according to the reported results, the charge density of virgin PI is about 0.15 mC m^−2^ in the ambient condition^17,18^, which barely reaches 0.56 mC m^−2^ in a high-pressure gas environment^19^. Due to the limitation of triboelectric properties, the charge density can only be realized to 1.67 mC m^−2^ on the engineering tribo-materials^5^. These comparisons indicate that solvent-assisted running-in increases the charge density of engineering polymer-based TENGs.

### Influence of tribo-materials, lubricants, and dissolvents on charge densities of TENGs

**Fig. 2:**
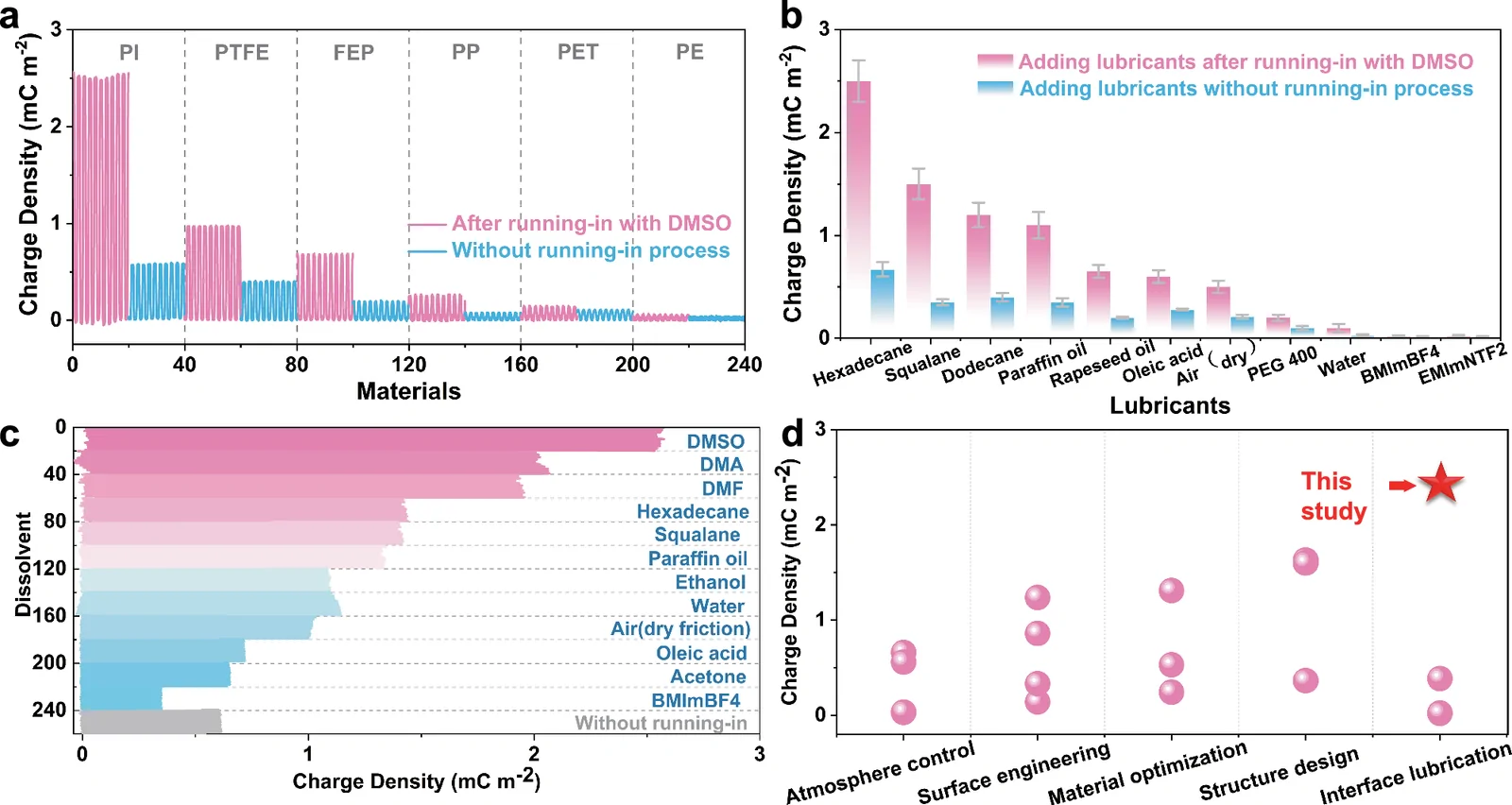
Output performance of the TENG with optimizing the running-in process. **a** Triboelectric charge density of different engineering tribo-materials lubricated by hexadecane for 20 s after running-in with DMSO. **b** Triboelectric charge density of engineering PI lubricated by different lubricants after running-in with DMSO. **c** Triboelectric charge density of engineering PI lubricated by hexadecane after running-in with different dissolvents. **d** Comparison of charge density of TENGs based on fundamental working modes^4,5,7,11,19,25–35^.

Compared with dry friction, proper lubrication can enhance the charge density of TENGs, which has been reported in previous works^7,11,12,20^. However, the previous lubrication enhancement work ignored the effect of the key role of the running-in. In this work, we find the running-in process plays an important role (Figs. 1 and 2). As shown in Fig. 2a, apart from the PI, the charge densities of conventional engineering materials, i.e., Polytetrafluoroethylene (PTFE), Fluorinated ethylene propylene (FEP), Polypropylene (PP), etc., are all boosted greatly by taking care of the running-in process. For example, there are about two-fold enhancements for the PTFE and FEP tribo-materials. Through the dissolvent-assisted running-in process in ambient conditions, the achieved surface charge density of FEP is about 0.7 mC m^−2^, which is much higher than the reported record (0.51 mC m^−2^) of the FEP tribo-material^21^. In addition, the PI-based TENG has much higher voltage and current than those without running-in treatment (Supplementary Fig. S2). The output voltage and current of the TENG lubricated by hexadecane after running-in with DMSO are about 680 V and 3.3 µA, respectively. In addition, the influence of lubricants during the triboelectric period is shown in Fig. 2b and Supplementary Fig. S2c, d. Under the lubrication of hexadecane, squalane, dodecane, etc., the PI-based TENGs have much higher charge density than those without running-in treatment. Among them, the enhancement of hexadecane is the best. It should be noted that even without lubrication (dry friction) during the triboelectric period, the surface charge density still increases from 0.15 mC m^−2^ (without running-in) to 0.36 mC m^−2^ (with running-in), which further confirms the key role of the running-in process on the output enhancement.

In general, the running-in process improves the solid–solid contact effectiveness^22^. Worn free radicals, debris, and transferred tribo-materials are generated at the same time, which will cause interface charge shielding and lower electrification capability^7^. The used dissolvents have the function of carrying worn radicals and debris, which is confirmed by the collected DMSO after the running-in process (Supplementary Fig. S3). The used DMSO is found to change color to yellow as a result of dissolving worn components, which display a strip-like shape with dimensions of several hundred nanometers (Supplementary Fig. S4). After that, the dissolvents are manually removed from the electric interface to achieve a clean surface/interface (Fig. 1b). It is found that dissolvents, such as DMSO, DMA, DMF, etc., during the running-in process can boost the electric output of PI (Fig. 2c and Supplementary Fig. S2e, f). The mechanism underlying this dissolvent treatment will be deeply discussed in the following parts of tribo-materials surface experimental characterization and molecular simulation. In addition, a high charge density of ~1.5 mC m^−2^ is achieved after hexadecane, squalane, or paraffin oil-assisted running-in treatment. It is worth noting that running-in treatment without any dissolvent (dry friction) can also enhance the triboelectric output (~1 mC m^−2^) (Fig. 2c). Differently, the charge density decreases when water, ethanol, or BMImBF_4_ is used during the running-in period. It is probably because the released ions from these proton/ion dissolvents are difficult to remove from the sliding interface, thus shielding the contact surface during the triboelectric period. For example, the electric outputs are very low through using the BMImBF₄, whether as the triboelectric lubricant (Fig. 2c) or as the running-in dissolvent (Fig. 2b). It has been widely identified in previous works^7,23,24^ that the released ions from liquids could adsorb on the electric surface and shield the electrostatic effect, which results in a lower electric output. It is found that there are large atomic concentrations of N, O, B, and F from BMImBF₄ on the running-in surface according to X-ray Photoelectron Spectroscopy (XPS) analysis on the electric surface of PI after running-in with BMImBF₄ (Supplementary Fig. S5). Time-of-flight secondary ion mass spectrometry (TOF-SIMS) was further performed to determine the interfacial accumulation of BMImBF₄ components (Supplementary Figs. S6 and 7). A much higher concentration of BMImBF₄-related anions, such as B^−^, F^−^, and BF_4_^−^, was detected in the running-in PI surface (Supplementary Fig. S6). There are also obvious concentrations of released cations, e.g., C_4_H_9_N_2_^+^, C_8_H_15_N_2_^+^, etc., appearing on the running-in PI according to the mass spectra and 2D distribution images (Supplementary Figs. S7 and S8).

Figure 2d summarizes the highest charge densities realized by recently reported methods for TENGs with typical working modes, i.e., vertical contact-separation mode, horizontal sliding mode, single-electrode mode, and freestanding mode. These methods include atmosphere control, surface engineering, material optimization, structure design, and interface lubrication. For example, the charge density could be boosted to 0.66 mC m^−2^ at the high vacuum condition (~10^−6^ torr)^25^. Via injecting radical anions onto the tribo-surface, a charge density of 1.2 mC m^−2^ was realized with a sliding mode TENG^4^. By regulating the contact barrier difference with an organic polymer on the inorganic high permittivity material^30^, the optimized inorganic tribo-material achieved a high charge density of 1.31 mC m^−2^. Designing the TENG’s structure with a space-accumulation effect^5^ could enhance the output performance with a much higher charge density of 1.63 mC m^−2^. The interface lubrication can reduce the friction and wear of TENGs significantly. However, this method achieved a relatively low charge density according to reported results^7,11,35^. The solvent-assisted running-in method used here yields a charge density of 2.5 mC m^−2^, compared with 1.63 mC m^−2^ reported for the space-accumulation approach^5^. The previous space-accumulation work also breaks the limit of the intrinsic charge density that defines the maximum charge density of triboelectric materials under vacuum conditions^8,17^. Further, the peak current and peak power density of the TENG are obtained under different external load resistance (Supplementary Figs. S9 and S10). According to the normalized peak power density as an essential metric^5,36–38^, the peak output power density of the TENG in this study reaches a maximum of 18.5 W m^−2^ Hz^−1^ at an external circuit load of 5 GΩ, which is also higher than other works (Supplementary Fig. S11), representing the advantage of the running-in optimization strategy in achieving high-performance TENGs. In contrast, the dry-frictional TENG as the standard sample has the lowest power density of 0.2 W m^−2^ Hz^−1^ at the external circuit load of 2 GΩ (Supplementary Fig. S10). It is found that the internal resistance of the TENG is high, which is similar to other work^4^. The main reason is that PI-based TENGs have a very low capacitance, which ultimately leads to a high internal resistance of the TENG^31^. Furthermore, the internal resistance of the lubricated TENGs is higher than that of the dry-frictional TENGs because the hexadecane lubricant is nonpolar and electrically insulating^7^.

### Analysis of the triboelectric surface after the running-in period

Triboelectrification originates from the movement of the two tribo-surface materials against each other, which, in most cases, experiences a friction force and wear. Here, we first compared the properties of the surface of PI after the running-in treatment with dry friction, hexadecane, and DMSO lubrication. The virgin PI in this study is smooth (roughness ~ 18 nm) (Supplementary Fig. S12), which matches a rough Al tribolayer (roughness ~ 162 nm) (Supplementary Fig. S13). During dry friction (Supplementary Fig. S14), the PI generates a very large coefficient of friction (COF) (~0.27) because of the direct contact between PI and Al during sliding. According to the Energy Dispersive Spectrometer (EDS) analysis, it also finds that many abrasive grains are transferred from the Al tribolayer to the dry-frictional PI surface (Supplementary Fig. S15). The transferred materials from one tribo-material to another will deteriorate the surface charge density. During the running-in period, added liquids, e.g., hexadecane and DMSO, can separate the contact asperities, thus making the COF decrease obviously (Supplementary Fig. S14). In addition, the interfacial liquids will capture worn radicals and debris and prevent them from adsorbing onto the PI surface. At the same time, there is little Al transferred on the PI surface (Supplementary Fig. S16). Instead, some materials are worn out from the PI surface (Supplementary Fig. S17) during the running-in process. The scratch depth of the running-in PI is about 400 nm when the hexadecane and DMSO are added. Whereas, the worn surface of the PI treated with DMSO is much rougher than that with the assistance of hexadecane. Thus, the DMSO-assisted running-in process has a surface grinding effect, so that the worn-out and transferred materials are difficult to adhere to the PI surface.

**Fig. 3:**
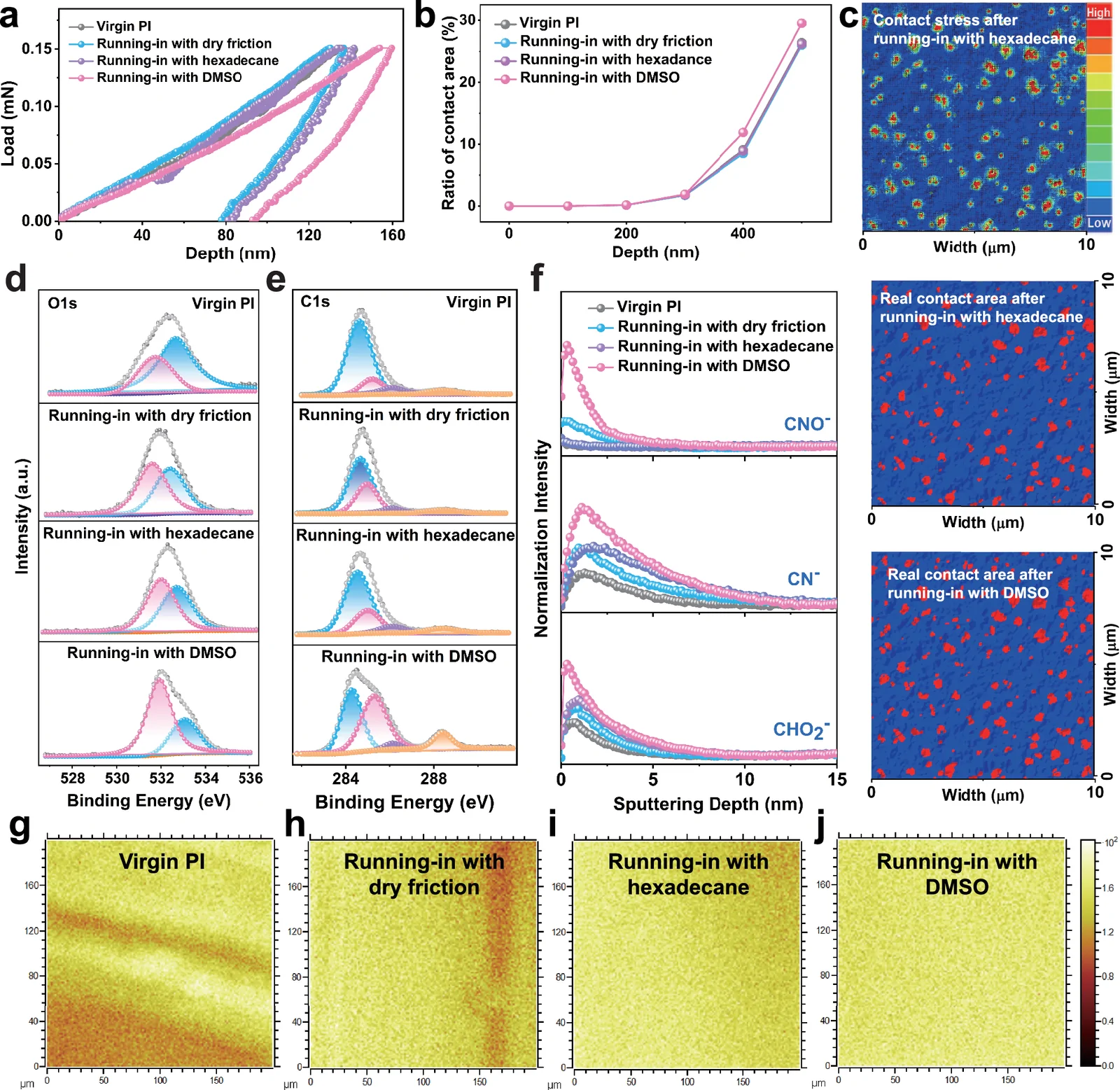
Physical and chemical properties of the triboelectric surface after the running-in period. **a** Load-displacement curve of the PI surfaces after the running-in period. **b** Ratio of the real contact area. **c** Contact stress distribution and real contact areas (red spots showing the in-of-contact zones). **d**, **e** High-resolution XPS spectra of O1s and C1s from the virgin PI, the PI after running-in with dry friction, hexadecane, and DMSO (4). **f** Content changes of CNO^-^, CN^-^, and CHO_2_^-^ fragments respectively along the depth direction. **g**–**j** TOF-SIMS images showing the distributions of the chemical fragment of CNO^-^.

The mechanical properties of the running-in surface are measured via nanoindentation shown in Fig. 3a and Supplementary Fig. S18. The average microhardness and modulus of the virgin PI are 0.52 GPa and 5.75 GPa, respectively (Supplementary Fig. S18). After the running-in with DMSO, the hardness and modulus of the PI surface are decreased by around 21% and 12%. Based on the triboelectric series list, electrons will be transferred from DMSO to PI when they are in contact^39^. Thus, DMSO will strongly adsorb on the PI surface due to the electrostatic force interaction. At the same time, the DMSO dissolvent with high polarity and short size can easily infiltrate into the PI matrix^40^. According to the previous study, the chemical chains of PI inside the contact area will be broken by shear stress^41^, which means that inter- and/or intra-molecule hydrogen bonds of PI will be damaged to differing degrees. Thus, the mechanical properties of PI are decreased significantly, and the surface becomes “softened”. The mechanical properties of the solid material determine the contact area directly, which plays an important role in the process of charge transfer. Firstly, the rough peaks of Al are in contact with the “softened” PI. Then, during friction, mismatched materials are easily worn and removed from the contact area for the case of the liquid-assisted running-in process, i.e., hexadecane or DMSO (Supplementary Fig. S17). In the end, the solid–solid contact effectiveness is improved significantly, resulting in a higher real contact area. Accordingly, the contact stress and contact area increase gradually with the increase of the depth (Fig. 3b and Supplementary Figs. S19 and 20) (details shown in “Methods”). Especially, the maximum contact stresses of the surfaces after running-in with hexadecane and DMSO are, respectively, about 0.98 GPa and 11.11 GPa at the depth of 400 nm (Fig. 3c). In addition, the numbers of red spots demonstrate that the contact state of the friction pairs (Fig. 3c and Supplementary Fig. S20). In this regard, the ratio value is 11.9% for the case of the PI after running-in with DMSO, whereas after running-in with hexadecane, the ratio value is about 8.9%. This thus means the real contact area size is increased by 33.7%, which is attributed to the lower mechanical properties of the PI after the running-in process.

It has been demonstrated that tribo-charges can only transfer at the areas of real contact^42,43^, so according to the previous theory, the tribo-charge of the TENG after the running-in with the hexadecane ~*Q*_*hexadecane*_ is:

1 $${Q}_{{{\rm{hexadecane}}}\,}={\sigma }_{{PI}}{A}_{r-{{\rm{hexadecane}}}}$$

${A}_{r-{{\rm{hexadecane}}}}$~ the real contact area after the running in with hexadecane, ${\sigma }_{{PI}}$~ the area density of tribo-charge transfer through ${A}_{r-{{\rm{hexadecane}}}}$, which is constant for the material pair (PI/Al). Equation (1) identifies the physical contact contribution to the charge transfer, which is also used in most cases of tribo-materials, e.g., the PI after the running-in with dry friction. However, it ignores the possibility of friction-induced chemical structure contributing to the charge transferring for the case of the running-in PI with the assistance of DMSO. Thus, the total tribo-charge ~*Q*_*T*_ of TENGs is attributed to both real contact areas (physical factor ~*Q*_*P*_) and induced molecular structures (chemical factor ~*Q*_*C*_). So, what is the situation for the running-in treatment of DMSO? Is there any contribution of the ${Q}_{C}$ for the PI after the running-in with DMSO? Here, a charge transfer model is proposed for the DMSO-assisted running-in PI in Eq. (2), in which the amount of charge is originated from strong electron-withdrawing groups ~${Q}_{C-{DMSO}}$ is considered:

2 $${Q}_{T-{DMSO}}={Q}_{P-{DMSO}}+{Q}_{C-{DMSO}}$$

Accordingly, the tribo-charge of DMSO-assisted running-in PI from the physical factor is obtained from the following Eq. (3):

3 $${Q}_{P-{DMSO}}={\sigma }_{{PI}}{A}_{r-{{\rm{DMSO}}}}=\frac{{Q}_{{{\rm{hexadecane}}}\,}}{{A}_{{{\rm{r}}}-{{\rm{hexadecane}}}\,}}{A}_{r-{DMSO}}$$

According to the results from Figs. 1 and 2, the values of ${Q}_{C-{DMSO}}$ and ${Q}_{P-{DMSO}}$ will be 80 nC and 180 nC, respectively. Thereby, the contribution ratio from the chemical factor for the tribo-charge of PI after the running-in with DMSO is about 31%.

Apart from the physical contact effect, the chemical factors, such as elemental composition and chemical structure of the tribo-surface, influence the charge density significantly after the running-in with DMSO. XPS quantitative analysis has been introduced to determine the species and content of chemical bonds on the surface of the running-in PI films. After the period of running-in, the contact surface was manually cleaned with acetone to remove the remnants of DMSO (Fig. 1b). It shows no S2p signal at the tested area (Supplementary Fig. S21 and Table S1), identifying that the residual DMSO has been completely removed after the running-in period. Regarding the peak fitting results of O1s and C1s spectra (Fig. 3d, e), the intensity of the C=O bond (~531.8 eV) for the running-in PI with DMSO is much stronger than the others. Moreover, based on statistical analysis, the content of the C=O bond in the virgin PI surface is about 11.3% (Fig. 3d, e and Supplementary Fig. S22). In comparison, it increases to 20% after the running-in with DMSO, which is also much larger than those after the running-in with dry friction and hexadecane, respectively. In addition, there is a very high content of C–N (~285.4 eV) on the surface of the running-in PI (Fig. 3e and Supplementary Fig. S23). In particular, the content value of the C–N bond in DMSO-assisted running-in PI is about 3.4 times that of the virgin PI. Whereas, the intensity of the C–O bond (286.1 eV) is much lower (Supplementary Fig. S24) in the case of the running-in with DMSO.

It is also found that there are few CO^-^ fragments on the running-in treated surface according to the result of TOF-SIMS (Supplementary Fig. S25). The N-related fragments (NH^-^) are obviously obtained on the running-in treated surface in contrast to the virgin PI (Supplementary Fig. S26). It means the C–N bond is easier to break and expose outward than that of the C–O bond in PI under the running-in process. The obtained NH^-^ and CHO_2_^-^ fragments are then formed on the running-in surface due to the presence of H_2_O and O_2_ molecules in DMSO and air^44^ (Supplementary Fig. S26 and Fig. 3f). It should be noted that the breakage thickness of the bond (C–N) is about 10 nm underneath the PI surface shown in Fig. 3f. Afterward, the new chemical bonds (-NH and -COOH) are formed with a thickness of lower than 5 nm (Supplementary Fig. S26 and Fig. 3f). The PI sample with the running-in with DMSO has a much higher intensity of NH^-^, CHO_2_^-^, and CN^-^ than those with dry friction and hexadecane lubrication. It is believed that these achieved polar chemical chains are exposed and outspread on the PI surface because of friction-driven DMSO-assisted solvent activation^45,46^. There is an obtained high intensity of C=O bonds after the running-in with DMSO, which confirms the DMSO solvation effect for extracting these polar chemical chains towards the contact surface. The breakage of C–N bonds not only improves the formation of CHO_2_^-^ and NH^-^ fragments but also weakens the steric hindrance of chemical groups. That is, the freedom degree of the imide group is released effectively. Together with the solvent activation of DMSO^45,46^, there is a remarkable increase in the imide group (CNO^-^) on the surface after the running-in with DMSO. The CNO^-^ is mainly located in the region with a depth of 0–8 nm (Fig. 3f). The 2D distribution of the fragment of CNO^-^ is shown in Fig. 3g–j. The detected intensity of CNO^-^ is much higher on the running-in treated PI, especially for the PI sample under the running-in with DMSO. Accordingly, complex physical and chemical reactions occur during the running-in process with DMSO. The C–N bonds in the PI molecule chains are mainly broken around carbonyl groups and benzenes, which results in the dangling bonds thus react with H_2_O and O_2_ so that some tribo-chemical products are achieved, such as the chain bindings (-CO-NH-, -CHO, -COOH, -NH-C). It is known that the C=O bonds in the imide group have strong electron-withdrawing capability, of which the electron-gain abilities are even stronger than the tribo-materials with the electronegativity atoms (F, Cl, etc.^47,48^). It is found that the binding of -CO-NH- has much higher electron affinity, which is confirmed by the highly negative Mulliken charge (Supplementary Fig. S27)^49^. The positive Mulliken charge of the -COOH binding is probably due to the acceptance abilities of the binding could be shielded by the outward-facing hydrogen atom.

### Molecular origin of the running-in process boosting the charge density

**Fig. 4:**
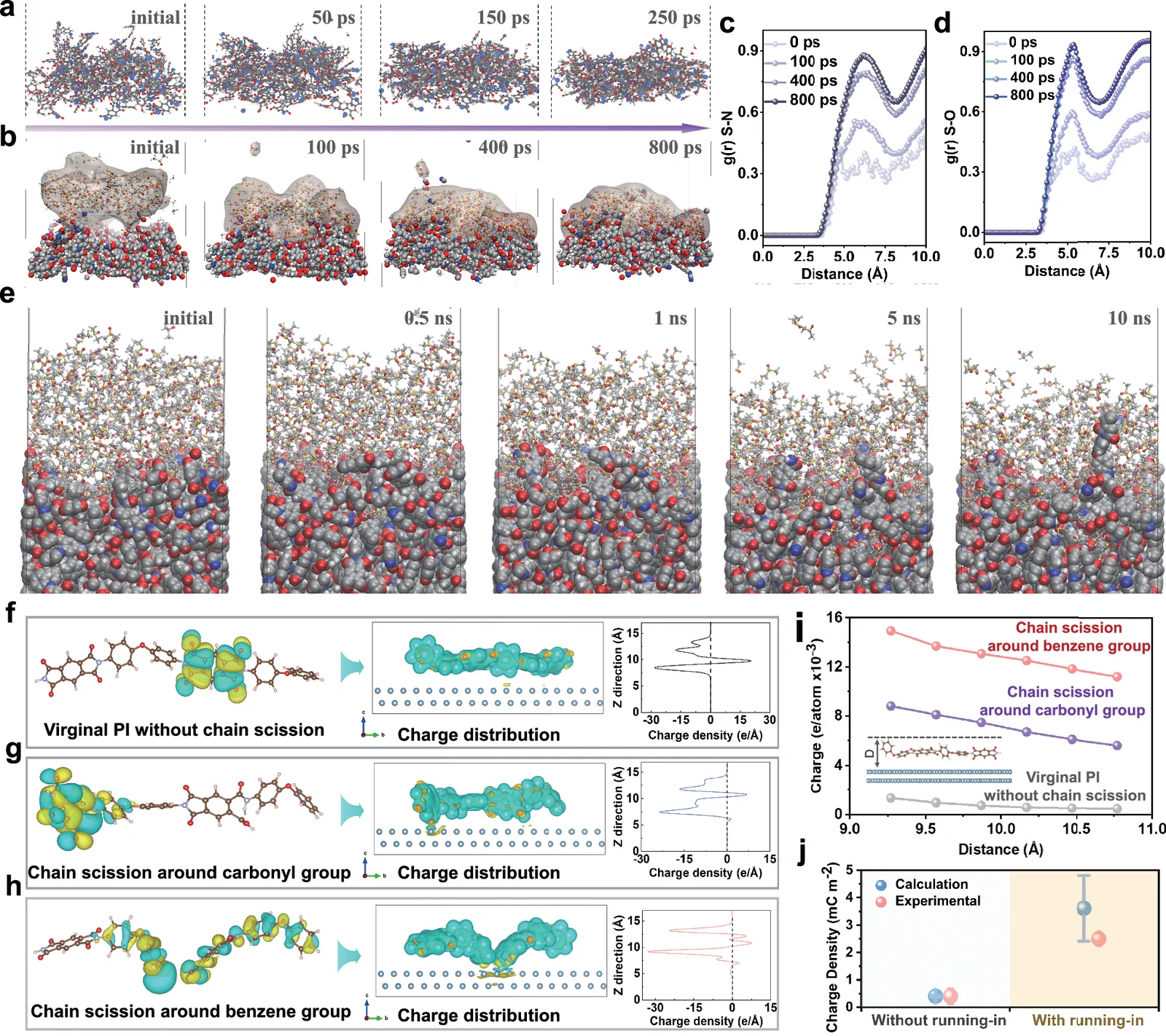
Triboelectric mechanism of PI with DMSO-assisted running-in process achieved via MD and DFT simulation. **a** Relaxation process of the PI bulk phase after friction with Al performed by classical MD. **b** Time-dependent dynamics of DMSO molecules infiltrating into the running-in PI. **c**, **d** Radial distribution function between the S atom of DMSO and the N, O atom in the running-in PI. **e** Time-dependent MD analysis of the extraction effect of DMSO molecule on the broken chemical chains, e.g., amide groups on the surface of the running-in PI. **f**–**h** Lowest unoccupied molecular orbital (LUMO), charge distribution, and 2D charge density difference of PIs. **i** Average number of electrons obtained by each atom of PIs. Inset demonstrating the distance between the top of the molecule and the contact interface. **j** Charge densities from the calculations according to Fig. 4i and from the experimental through lubrication without running-in process or lubrication after running-in with DMSO according to Fig. 2b.

As shown in Fig. 1, high charge density of TENGs can be achieved through two parts, i.e., the running-in process with the assistance of DMSO and the triboelectric period with the lubrication of hexadecane. Firstly, to further understand the DMSO-assisted solvent activation, the molecular models of the PI sliding against a virtual Al within DMSO were developed by a ReaxFF-based reactive molecular dynamics simulation and a polymer consistent force field-based classical simulation^50,51^ (“Methods”). It is found that the bulk phase-vacuum interface becomes shrunk and planarized after the kinetic relaxation process, which is attributed to strong non-covalent interactions (likely van der Waals forces) between the terminal long-chain functional groups (Supplementary Fig. S28). Then, to simulate a realistic friction process, a virtual Al substrate was applied to interact with the pre-equilibrated PI bulk model as shown in Fig. 4a. It can be seen that the friction force induces the breakage of the relatively unstable bonds (i.e., N–C) on the polymer surface, of which the simulation result agrees well with that from the experimental results (Fig. 3). The generated free radicals and debris with high polarity are mainly caused by lone pair effect of N atoms and O atoms. In addition, each broken bond leaves an unsaturated (radical-like) site on the polymer surface, thus creating highly polar chain-end functionalities. These highly polar chains can be strongly attracted by polar solvents (i.e., DMSO) via dipole-dipole interactions^52^. In contrast to the virgin bulk PI (Supplementary Fig. S28), the surface of PI changes to being rough obviously after friction, although it also becomes shrunk with further relaxation (Fig. 4a), which agrees with the experiment results after mechanical wear (Supplementary Fig. S17).

Accordingly, the time-dependent dynamics demonstrates that the DMSO molecules infiltrate the running-in PI with unsaturated functional groups (Fig. 4b). It can be clearly seen that the contact area between the solvent and polymer surface increases continuously. The PI surface is fully covered by DMSO molecules after relaxation for 800 ps. Thereby, the generated free radicals and debris will desorb from the surface and dissolve/disperse in DMSO (Supplementary Fig. S3), which will be removed after the running-in process (Fig. 1b). Furthermore, the variations of the radius distribution function are introduced here to identify the interaction between DMSO and PI, shown in Fig. 4c, d. The value of radius distribution function between the S atoms in DMSO and the N atoms in the running-in PI increases gradually at a distance ranging from 3.3 Å to 9.9 Å with the time relaxation (Fig. 4c), in which the peaks of the radius distribution function values are mainly distributed around 6 Å. It means the DMSO molecule effectively infiltrates into the molecular chains of PI, which is also confirmed by the result of radius distribution function between the S atoms in DMSO and the O atoms in the running-in PI (Fig. 4d). In this case, the broken chain scissions from PI are easily extracted, driven by the infiltration of DMSO (Supplementary Movie 1). Especially, the released polar amide groups are transmitted towards the contact interface as “molecular brushes” as shown in Fig. 4e. As a result, the imide group with strong electron-withdrawing capability is exposed and outspread on the PI surface, which significantly improves the electron affinity of PI. The configuration consists of a running-in polymer surface with unsaturated sites in contact with a layer of equilibrated DMSO molecules. For the case of virgin PI, the imide groups are mainly confined to the molecular chains of PI. For this case, the freedom degree of the imide groups is so limited that they cannot be extracted towards the contact interface with the assistance of DMSO (Supplementary Fig. S29 and Movie 2). These molecular-level simulation results confirm the working mechanism of DMSO solvent activation during the running-in process.

After running-in with DMSO, the contact surfaces are then fully cleaned manually, and a lubricant, e.g., hexadecane, is added to the sliding interface (Fig. 1b). The density functional theory simulation is thus introduced to demonstrate the atomic-level triboelectricity involving the activated groups of PI from the results of the simulation under the lubrication of hexadecane. It is found from the simulation that the N-related bonds in PI are broken, resulting in molecular chain scission at the C–N bonds during the running-in with DMSO (Fig. 4e). The bond scission events predominantly involve N–C bonds located adjacent to the imide linkages, leading to the formation of amine- and carbonyl-terminated fragments at the polymer surface, which is confirmed according to the TOF-SIMS results (Fig. 3f). In contrast, cleavages of C–C and C–O bonds are only occasionally observed within the simulated time window. This consistent pattern across independent simulations supports the proposed selective N–C dissociation mechanism. It is physically reasonable and aligns with reported density functional theory results in the literature^53,54^, where imide N–C bonds in aromatic polymers exhibit lower dissociation barriers (~3.0–3.5 eV) compared with C–C bonds (~3.8–4.0 eV). Therefore, it is believed that the ReaxFF trajectories provide a reliable qualitative basis for elucidating the dominant dissociation and solvent–polymer interaction mechanisms under frictional conditions. Accordingly, two molecular configurations in running-in PI with different bond breakage are thus introduced to demonstrate the atomic-level triboelectricity shown in Fig. 4f–h, which is in contrast to the virgin configuration of PI. The density functional theory is performed by using the Vienna Ab initio Simulation Package. The minimum value of binding energy represents the most stable contact state (Supplementary Fig. S30)^47^. For this case, the lowest unoccupied molecular orbital, as the electron acceptor orbital, is mainly located on the imide and benzene rings of virgin PI (Fig. 4f)^47^. So, there are electrons transferred between the available imide groups and Al surfaces. The imide groups have a significant the lowest unoccupied molecular orbital when their freedom degrees are released after the breakage of C–N bonds during the running-in with DMSO (Fig. 4g, h). It is found that when the breakage of the N–C bond occurs during running-in with DMSO, the -COOH and -CHO are generated because of incoming O_2_ and H_2_O molecules from the atmosphere, shown in Fig. 3f and Fig. 4g, h. Here, the lowest unoccupied molecular orbital is more easily located in the -CHO groups in contrast to that of the -COOH groups, which means for this case, electron transfer mainly occurs around the running-in induced -CHO groups rather than that of imide groups in the virgin PI.

On the other hand, the electron density differences at Al-PI contact interfaces are also shown in Fig. 4f–h. The electron-gain areas are mainly limited in the micro-contact interface between the Al surfaces and the released PI chains by the running-in process. To explore the charge redistribution at the Al/PI interface, the 2D charge density difference has also been calculated by density functional theory calculations. For the case of running-in PI with the N-related chain scission around benzene (Fig. 4h), there is a significant peak of electron loss at the interface of Al/PI (5 Å). At the same time, the electron gain in the nearby PI molecules is also obvious, which identifies that the electrons are transferred from the Al to the running-in PI. In contrast, the peak of electron gain is weaker for the case of the running-in PI with the fracture of N–C bonds around carbonyl groups (Fig. 4g). The peak of electron gain in the virgin PI sample at the interface (5 Å) is minimal (Fig. 4h).

In addition, running-in friction induces the breakage of the relatively unstable N–C bonds in PI, resulting in the freedom of releasing electron-acceptor groups, which means the running-in PI has higher flexibility. Together with the function of DMSO-driven extraction, the chemical chains containing amide groups are regulated to be “molecular brushes” outspread on contact surfaces. Thus, the distance between the C=O groups and the Al surface is significantly decreased for the broken PI. Accordingly, the effect of vertical relative positions on the charge transfer of PI-Al is also studied by changing the PI-Al interface distance. Considering the differences in the spatial structure of the PI molecule, the distance from the top of the PI molecule to the Al surface is utilized as the X-axis parameter shown in the inset of Fig. 4i. The amounts of transferred charges decrease with the increasing distance, which, however, are very different depending on the PI samples. For example, at a distance of 9.3 Å, the transferred electron of the running-in PI with the broken C–N bonds around carbonyl groups is 0.0149 e/atom, while the amount value is 0.0088 e/atom for the sample with the fracture of the C–N bond around benzenes. Accordingly, there is an average one order of magnitude higher amount of transferred charges for running-in PIs with the N-related chain scission in contrast to the virgin PI. Herein, it is greatly expected to obtain the charge density of PI regarding the density functional theory analysis. Accordingly, the charge density of PI is 3.6 ~ 4.4 mC m^−2^ based on the transferred charge of PI (“Methods”). It should be noted that the interface of PI/Al is hypothetical at a full contact state (ratio of contact area ~ 100%). Considering the practical contact in the Al/PI interface with a real ratio of contact area of about 10% (Fig. 3b), i.e., 90% of interfacial atoms cannot contribute to charge transfer. Accordingly, as shown in Fig. 4j, the calculated charge density (0.36 ~ 0.44 mC m^−2^) is thus similar to the experimental result of PI (average ~0.4 mC m^−2^) through adding lubricants of hexadecane, squalane, dodecane, and paraffin oil without running-in process (Fig. 2b). For the case of running-in with DMSO, the calculated value of charge density ranges from 2.36 to 4.86 mC m^−2^ regarding the transferred electron at the assumed condition of broken C–N bonds (Fig. 4i), meaning the value range contains the experimental charge density (2.5 mC m^−2^) of PI lubricated by hexadecane after running-in with DMSO (Fig. 2a). The density functional theory results identify the attractive optimizing method for the triboelectric enhancement, and also agree with the experimental results very well (Fig. 4j).

### Fast DMSO-assisted running-in strategies towards enhancing electric output, durability, and triboelectric series

**Fig. 5:**
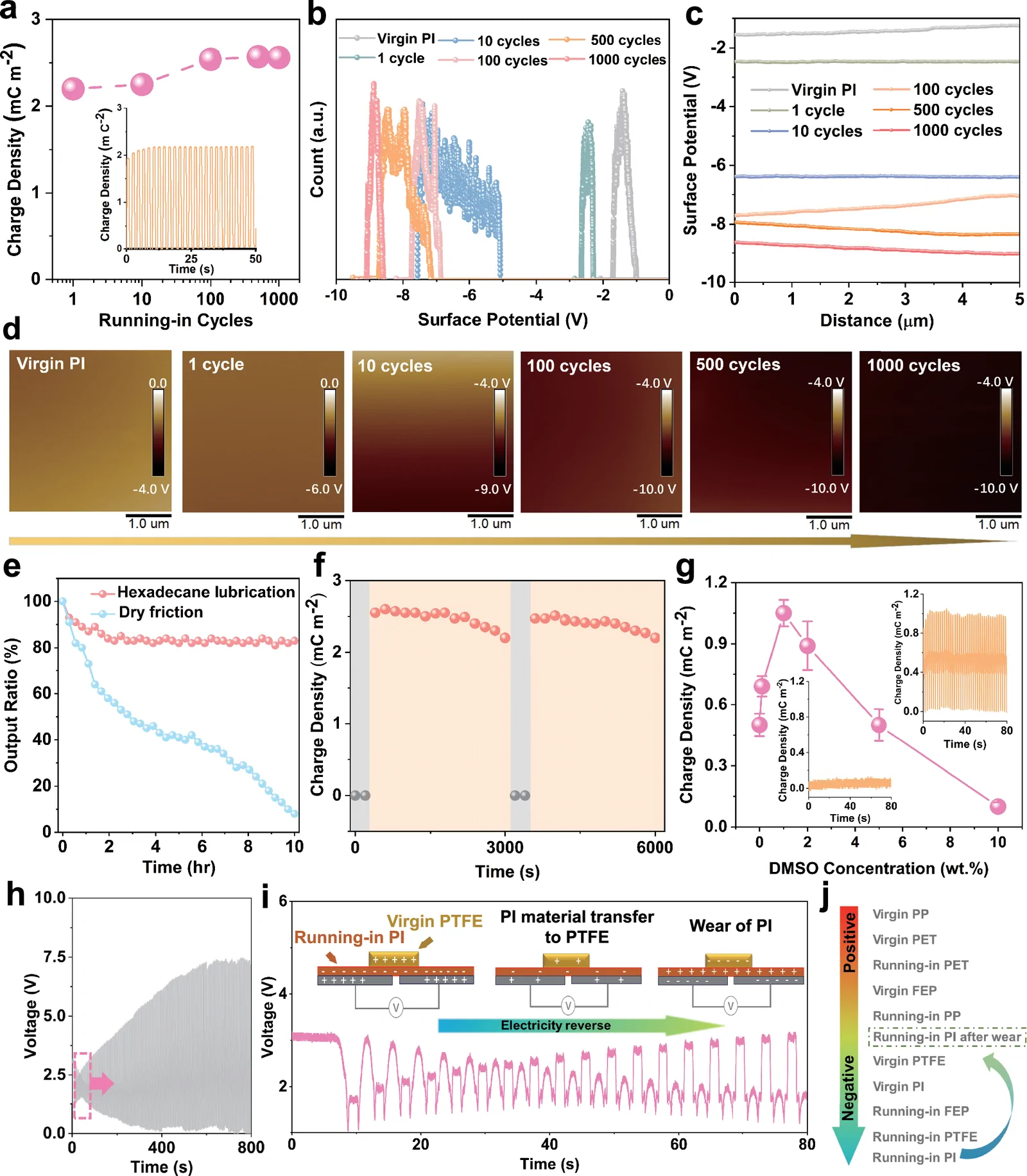
Fast DMSO-assisted running-in strategies towards triboelectric enhancement and reversal effect. **a** Relationship between the running-in cycle and the triboelectric output. **b** Relative pixel distribution with respect to the surface potential value after running-in with DMSO. **c** Extracted line scan profile with respect to the surface potential value after running-in with DMSO. **d** Surface potential profile of the PI surfaces after running-in with DMSO. **e** Output ratio of the charge density for the hexadecane-lubricated TENG after running-in with DMSO compared with the dry-frictional TENG in the long-term test. **f** Charge density of the running-in PI with hexadecane lubrication after intermittent running-in processes. **g** Charge density of the virgin PI lubricated by the mixture of hexadecane and DMSO. **h**, **i** Triboelectric output of the friction pairs between the running-in PI with virgin PTFE, the inset in Fig. 5i demonstrating the electric reversal mechanism of the friction pairs. **j** Triboelectric series of engineering dielectric materials for lubricated sliding modes considering the running-in optimization.

The high surface charge density of TENGs can be achieved via the DMSO-assisted running-in process that induces surface molecular chain scission and rearrangement, making electron-withdrawing groups toward the triboelectric surface. In the area of tribology, the duration of the running-in period is uncertain to some extent, and sometimes it takes thousands of sliding cycles^15^. Surprisingly, it is found in Fig. 5a that the high charge density ~ 2.2 mC m^−2^ is able to be achieved just with one running-in cycle! It means the scission and rearrangement of molecular chains occur quickly under the friction-induced DMSO-solvation. After 100 cycles of running-in, the electric output increases to as high as 2.5 mC m^−2^, meaning the triboelectric polarity of the PI changes to much more negative through the running-in optimization. Accordingly, the surface potential of the PI surface using Kelvin Probe Force Microscopy (KPFM) are carried out (Fig. 5b–d), and it is clearly obtained that the surface potential changes obviously after the running-in process. From the extracted pixel distribution (Fig. 5b) with respect to the surface potential value, the change occurred after the first cycle, and there is a significant change in the surface potential after running-in for 100 cycles (Fig. 5c). It is evident from the KPFM results that the proposed running-in process leads to a much lower surface potential, which is correlated with a higher output in the TENG. A lower surface potential, implying a more negative potential, indicates a stronger electron-withdrawing ability^55^. In contrast, the virgin PI surface exhibits a relatively high potential on the surface. This observation aligns with our findings that the charge density is able to be achieved with one running-in cycle (Fig. 5a). After 100 cycles of running-in, the triboelectric polarity of the PI changes to much more negative (surface potential ~ −6.98 V). It is because the scission and rearrangement of molecular chains occur quickly through the running-in optimization. Thus, the surface potential via KPFM can successfully identify the change in the chemical structure of PI surfaces.

In addition, the dielectric analysis of the electric materials is also a good way to explain triboelectrification. Scanning force microscopy based on capacitance and electrostatic force is used for measuring the dielectric properties of insulating films at the nanoscale^56–58^. The detection principle involves defining the phase change of the probe under actual vibration state, in contrast to the free vibration phase in Electric Force Microscopy^59,60^. The relationship between the phase of probe vibration and voltage can be expressed as Eq. (4) (“Methods”):

4 $$\tan \varphi \propto A{\left({V}_{{tip}}-{V}_{S}\right)}^{2},A=-\frac{Q}{K}\frac{1}{2}\frac{{\partial }^{2}C}{{\partial Z}^{2}}$$

where *φ* is the phase of probe vibration, ${V}_{{tip}}$ is the probe voltage, ${V}_{S}$ is the surface potential of the sample, *Z* is the distance between the probe and sample, the lifting height, and *C* is the system capacitance composed of the probe and sample. It means that there is a quadratic function relationship between tan φ and the electric potential difference (*V*_*tip*_−*V*_*s*_), in which the factor *A* is the coefficient of the quadratic term. The factor *A* is related to the capacitance between the probe and the sample, according to the Eq. (4). According to the results of various probe-sample capacitance models^59,60^, the probe-sample capacitance increases with the increase of the dielectric constant *ε*, so the absolute value of the factor *A* has a positive relationship with the dielectric constant *ε* of the sample. Therefore, EFM can be used to detect the changes in local dielectric properties of PIs. For example, the phase evolution ~*φ* of the running-in PI under different tip voltages compared with the virgin PI is shown in Supplementary Fig. S31. Through establishing the quadratic function relationship between the tan *φ* and the electric potential difference (*V*_*tip*_−*V*_*s*_) (Supplementary Fig. S32), the running-in PI has a larger absolute value of the factor *A* (~0.00112) than that of the virgin PI (~0.00101), so the local dielectric property of the PI is enhanced after running-in process.

After that, the surface roughness of the running-in PI is determined by AFM surface topography measurements. The surface roughness of the running-in PIs increases with the increase of the running-in cycle, whereas the difference in the local surface roughness is not notable (≥500 cycles) (Supplementary Figs. S33 and S34). Further, the in-depth profiles of the molecule fragments are carried out after running-in at different cycles via TOF-SIMS (Supplementary Figs. S35 and S36). It is found that the intensity of CNO^-^ obviously increases with the increase of running-in cycles. The detected intensity of CNO^-^ in the 2D distribution is much higher on the running-in PI after more than 10 cycles (Supplementary Figs. S35a and S36). It is also noted that after running-in for 100 cycles, the change trends of the intensity are not obvious, e.g., for CN^-^ and NH^-^ fragments (Supplementary Fig. S35b, c). After 1000 cycles, there is an available signal of TOF-SIMS on the CO^-^ fragment probably due to surface oxidation during running-in (Supplementary Fig. S35d). Thus, with the DMSO-driven extraction effect, the polar chemical chains are regulated to be “molecular brushes” outspread on surfaces, which could be strongly enhanced by the cycles of the running-in process. The frictional energy consumption based on the work done of friction forces is obtained at different running-in cycles according to the COF (Supplementary Fig. S37) and sliding length^61^. In this regard, the surface potential decreases dramatically at an energy consumption of ~0.2 N·m (10 cycles) (Supplementary Fig. S38). Over the value of the energy consumption, the detected TOF-SIMS intensity of CNO^-^ in the 2D distribution is much higher (Supplementary Figs. S35a and S36). Further, it should be noted that within a frictional energy consumption of ~2.07 N·m (100 cycles), the differences of the TOF-SIMS intensity are not obvious, e.g., for CNO^-^, CN^-^, and NH^-^ (Supplementary Fig. S35a–c). At the same time, the difference in surface potential is relatively low over the frictional energy consumption, which means the friction-consumed energy ~2.07 N·m is required for achieving reliable structural evolution and high electric outputs of PIs (100 cycles) (Supplementary Fig. S38). The running-in PI for 100 cycles is thus focused on for studying the influence factors on the electric output after the fast DMSO-assisted running-in process as follows.

The charge leakage behavior of the TENG is carried out as shown in Supplementary Fig. S39. The dissipation of the charge decay is very low as similar to previous work^30^. After 2500 s, the residual charges on the surface are 99% of the original charges because the charged surface is fully covered by the hexadecane, so that the air breakdown and leakage are prevented successfully^12^. In addition, the dielectric leakage current is as low as 1 pA with a bias voltage from 200 V to 1000 V (Supplementary Fig. S40)^62^. It should be noted that the leakage current of the TENG after running-in is consistent with that of a virgin PI-based sample, which means the running-in process does not influence the charge decay and leakage.

In addition, the influence of environmental stresses, e.g., gas, humidity, and temperature, is also carried out for the lubricated TENG after the running-in process, in contrast to the TENG under dry friction. As shown in Supplementary Fig. S41, the electric output of the lubricated TENG maintains a relatively high retention rate of 90% at a high humidity of 60%RH, which has a much better performance of humidity resistance than that under dry friction. It is because the electric interface is fully covered with the lubricant so that the water is difficult to diffuse into the Al tribolayer and the PI surface^63^. Further, it is found that the gas atmospheres, e.g., O_2_, N_2_, and CO_2_ have little impact on the electric output of the lubricated TENGs after running-in with DMSO (Supplementary Fig. S41b–d), which is very different from that of the TENG under dry friction. At the beginning of dry friction, the effect of O_2_ on the TENGs is not obvious when the concentration is lower than 50% (Supplementary Fig. S41b). Whereas, the ratio of voltage output is reduced by about 40% as the O_2_ concentration gradually exceeds 70% because of the adsorption of O_2_ onto the contact surface under dry friction^64^. Similarly, the electric output of the dry-frictional TENG gradually decreases with the increase of N_2_ concentration (Supplementary Fig. S41c). Both the lubricated and dry-frictional TENGs keep a high and stable electric value in a CO_2_ atmosphere^64^ (Supplementary Fig. S41d). Furthermore, a systematic investigation of the impact of environmental temperature on the electrical performance of TENGs is essential, considering their application across diverse regions worldwide, where recorded extreme temperature can be as high as 55 °C^65^. Although the electric output decreases dramatically with the increase of temperature, the reduction in the output ratio is less obvious up to 50 °C (Supplementary Fig. S42). On the other hand, the change in the device operation, i.e., sliding frequency, can directly affect the electric output of TENGs according to the second part of the equation from the first principle theory^66^. With the increase of sliding frequency, the output current increases obviously (Supplementary Fig. S43a), meaning the value of output current is proportional to the frequency, which aligns with previous works^5,67^. However, the electric interface between the Al tribolayer and the PI surface will be further separated by the lubricant because of the hydrodynamic effect according to liquid lubrication theory^68^. Thus, the output charge decreases slightly at a high sliding frequency (Supplementary Fig. S43b). In this case, all compared electric performances are carried out in the same environmental condition and device operation.

The long-term stability of TENGs is very important for the practical application, which is examined as shown in Fig. 5e. After more than 10 h (~18,000 cycles), the lubricated TENG exhibits superior electric durability and maintains 86% output (>2 mC m^−2^) which is still much higher than previous works (Fig. 2d). Although there is a little attenuation on the electric output, the charge density of the lubricated TENG significantly increases within a fast running-in process of 100 cycles (Fig. 5f). The high electric output of the TENG is attributed to the regulation of amide-containing chemical chains into “molecular brushes” that extend toward the contact surfaces. These outspread groups could be worn during triboelectrification, probably resulting in a decrease in the electric output. In contrast, the same TENG device retains only 9% electric output under dry friction in air. In addition, the COF of the dry-frictional TENG is two times as much as that of the lubricated TENG (Supplementary Fig. S44a). The PI surface suffers severe wear after dry friction in contrast to the PI surface with the hexadecane lubrication (Supplementary Fig. S44b). Thus, the TENG with the hexadecane lubrication has good electric durability and tribological performance.

For the above tests, after the running-in process, the contact surfaces are then fully cleaned manually to remove the residual DMSO (Fig. 1b). Another electric test is carried out here without any cleaning action. It is found that there is still a high electric output (~1.2 mC m^−2^) when the hexadecane lubricant (~1 mL) is directly added after running-in with a low volume of DMSO (~0.2 mL) (Supplementary Fig. S45), meaning the solvent-assisted running-in process could be further simplified. This study further provides a liquid lubricant via a mixture of DMSO with hexadecane to improve the output efficiency and to enhance the durability of the TENGs shown in Fig. 5g. The charge density of the TENG increases firstly with the increase of DMSO in hexadecane. The output value of charge density increases from 0.6 mC m^−2^ to as high as 1 mC m^−2^ at a proper concentration of 1 wt%. From then on, the value decreases because the high concentration of DMSO could result in the conduction of the contact interface. The charged DMSO molecule is also easy to adsorb onto the surface and generate a shielding effect. The charge density of the TENG decreases to as low as 0.1 mC m^−2^ at a high concentration of DMSO in hexadecane (10 wt%). In contrast to the dry friction, the COF is much lower for the case of hexadecane lubrication (Supplementary Fig. S14) because the hexadecane fluid has the capability of separating the two parts of the contact surfaces, resulting in friction and wear reduction^7^. Accordingly, utilizing DMSO as a triboelectric additive in lubricants can generate a solvation effect under friction induction (Fig. 5g). It is also found that the change in the COF is not obvious when the DMSO is added to hexadecane (Supplementary Fig. S46). Therefore, the non-proton solvents can be used as additives in lubricants to improve the performance of triboelectricity and tribology.

Accordingly, the PI after the treatment with DMSO has a stronger electron-capturing capability than that of PTFE, FEP, etc., which means the electronegativity of the PI after running-in with DMSO is higher. So, it is wondered what the triboelectric behavior would be if the Al tribolayer is replaced by PTFE, i.e., the PI in contact with PTFE. As shown in Fig. 5h–j, the virgin PTFE is chosen as an electric material instead of the upper Al tribolayer (inset of Fig. 5i). The interface electrons are apparently transferred from PTFE to the running-in PI, which is indeed observed for short charging cycles, confirming the stronger electron-capturing capability of the running-in PI than that of PTFE. It is also found that, for long cycles, the magnitudes of the electric signal start to decrease and the polarities of electricity are reversed, i.e., the running-in PI started charging positively and the PTFE negatively. The electric reversal is probably because the chemical groups or ions in the running-in PI transferred to PTFE during sliding friction due to wear^69^. Together with the results in Fig. 2, the triboelectric series, which considers the running-in effect, is proposed for the case of lubricated sliding modes (Fig. 5j). The triboelectric polarity of the running-in PI is the most negative while the virgin PP has the most positive state regarding the results of Fig. 2. The ranking of the running-in PI after wear (inset of Fig. 5i) changes behind to virgin PTFE in the triboelectric series shown in Fig. 5j. The key factors that influence the triboelectricity of TENGs are summarized according to triboelectric mechanism analysis (Figs. 4 and 5). The chemical chains containing amide groups outspread towards contact surfaces through running-in optimization. Firstly, the running-in cycles directly influence the structure evolution of PIs. Especially, after 100 cycles of running-in, the triboelectric polarity of the PI changes to much more negative through the running-in optimization (Fig. 5b). Running-in friction can induce the breakage of the relatively unstable N–C bonds in PI, resulting in the freedom to release amide groups. In addition, although the electric output can be enhanced after running-in under the dry condition (Fig. 2c), the running-in dissolvent is necessary to regulate the chemical chains containing amide groups outspread towards contact surfaces, among which the C = O bonds with strong electron-withdrawing capability are thus exposed and capture electrons from the contacting tribolayer. Further, it is known that the triboelectric performances will be significantly boosted via introducing nonpolar liquids in the electric interface, whereas the ionization property of dissolvents should be focused on for the running-in process, rather than the solvent polarities. Non-proton dissolvents, e.g., DMSO, DMA, and DMF, have the key contributions to the triboelectricity in contrast to the proton and/or polar dissolvents (Fig. 2c). It is because the released ions from proton and/or polar dissolvents could adsorb on the electric surface (Supplementary Figs. S7 and S8).

## Discussion

In summary, this study shows that solvent-assisted control of the running-in process can improve the output of TENGs made from engineering tribo-materials including PI, PTFE, and FEP. The as-received commercial PI after the solvent-assisted running-in process achieves a surface charge density of 2.5 mC m^−2^ and a power density of 18.5 W m^−2^ Hz^−1^.

Accordingly, we have summarized the key roles for elevating the charge density on the basis of the solvent-assisted running-in process. Firstly, worn radicals, debris, and transferred tribo-materials are generated during the running-in period, which always causes interface charge shielding and lowers electrification capability. Non-proton solvents such as DMSO, DMA, and DMF are able to fully dissolve/disperse them, making them easily removed from the running-in surface. In contrast, proton/ion solvents will release ions and adsorb on the electric surface, thus shielding the surface and preventing triboelectric charge generation.

Secondly, the solvation effect of DMSO provides a relatively soft electric surface of PI after the running-in period. As a result, the real contact surface between the running-in PI and Al tribolayer is increased by 33.7% compared with that of the virgin PI. Here, we further proposed the quantitative contribution of physical and chemical effects on triboelectricity for the case of DMSO-assisted running-in optimization. The contribution ratio of the physical contact is about 69%, while that of chemical groups is 31%.

Thirdly, the friction force induces the breakage of the chemical chains around the unstable N–C bonds, releasing the freedom degree of amide groups. With the DMSO-driven extraction effect, the chemical chains containing amide groups are regulated to be “molecular brushes” outspread on surfaces, among which the C=O bonds with strong electron-withdrawing capability are thus exposed and capture electrons from the contacting Al.

It is worth mentioning that the high charge density is able to be achieved within one running-in cycle! Apart from that, the optimized running-in process has been applied to enhance the triboelectric durability, which will also rearrange the triboelectric series of engineering dielectric tribo-materials for lubricated sliding modes. This study also provides a liquid lubricant to improve the output efficiency of TENGs. These findings link tribological running-in with triboelectric charge generation and suggest a practical route for improving polymer-based TENGs.

## Methods

### Fabrication of sliding TENGs

The slide-mode TENG was composed of two plates using flat square polymethyl methacrylate (PMMA) boards with 4 mm thickness as the supporting substrate. The upper and lower plates of TENGs were Al electrodes attached to the PMMA with a width of 1 cm. The thickness of the Al electrode is 0.06 mm. Kapton film (PI, 0.05 mm thick), PTFE film (0.05 mm thick), and other films were used as received. To ensure good contact, four cushions within double-sided tapes were put under the reciprocating (the lower) plate.

### Frictional and electrical measurement

The slide-mode TENG tests were carried out by using the reciprocating mode of the tribometer (UMT-CETR, Germany) in an atmospheric environment (temperature: 25 °C; humidity: 22%RH). 1 mL dissolvent, e.g., DMSO, is added to the electric PI during the running-in process. After that, the dissolvent was removed by utilizing a dust-free paper, and the running-in PI surface was then cleaned with 1 mL of acetone, which was also removed similarly. The programmable electrometer (6514, Keithley Instruments model) was utilized to test the electric output of the TENG. For studying the influence of environmental stress, the gases (N_2_, O_2_, and CO_2_) were purchased from Sichuan Shunlong Xinteng Chemical Co., Ltd. and delivered by silicone tube. The N_2_ sensor (ZY-20N, Jiande Shukang Instrument Co., Ltd, China), the O_2_ sensor (CY-12C, Jiachang Electronic Technology Co., Ltd, China), and the CO_2_ sensor (RS/F6-CO_2_-N01-2Y, Renke Control Technology Co., Ltd, China) were applied to measure the concentration of gases. The environmental temperature and humidity were monitored with the temperature sensor (TH-30PRO, Anymetre, China). For the leakage I–V test, an insulation resistance meter (TH2684A, China) was used as a high voltage source, and the leakage current of the TENG was measured by this insulation resistance meter as well.

### Sample characterization

The three**-**dimensional (3D) images and roughness of contact surfaces were investigated using a Zygo NewView 7300 instrument (Zygo Corporation, Middlefield, CT, USA). The microstructures of the Al and PI surfaces were studied by a field-emission scanning electron microscope (S-4800 FESEM, Hitachi, Japan). The elemental distribution in the rubbing surface was studied with energy dispersive X-ray spectroscopy (EDX). The surface topography, surface potential, and electrical phase after the running-in process were measured with AFM (Atomic Force Microscope), KPFM (Kelvin Probe Force Microscope), and EFM (Electric Force Microscopy). The AFM/KPFM/EFM measurements were conducted with the Dimension ICON (Bruker, Germany). All measurements were performed using a Pt-coated probe (Multi75E-G-10). In EFM measurements, the scanning bias voltage is in the range of 0 V to 10 V, and the lift distance is 100 nm.

### Finite element calculations

Practical engineering surfaces frequently exhibit non-Gaussian height distributions and anisotropic topographic characteristics, presenting challenges for traditional Gaussian models. The Fourier filtering approach, which governs surface statistics through manipulation of frequency-domain energy distribution, enables precise matching of both target power spectral density and height distribution. Here, this study employs a fast Fourier filtering algorithm to generate fractal surfaces characterized by specified power spectral density profiles^70^. The power spectral density slope is −2(H + 1), where H is the Hurst exponent. Three characteristic parameters impact surface topography: root-mean-square slope (m_02_ + m_20_)^1/2^, Hurst exponent H, and wavelength ratio λ_l_/λ_s_. The Nayak parameter α = m_00_·m_40_/m_20_^2^, which characterizes the breadth of the surface spectrum, is provided for each case, where m_ij_ (i, j = 0, 2, 4) represents the surface spectral moments. For validation, the finite element results of real contact area fraction versus dimensionless normal pressure have been compared against established solutions from the literature using identical rough surface parameters (m_02_ + m_20_)^0.5^ = 0.1, H = 0.8, λ_l_/λ_s_ = 4, L/λ_l_ = 8)^71^. Good agreement was achieved with the boundary element method results by Yastrebov et al.^72,73^, who applied periodic boundary conditions. Our results are also reasonably placed within the range of results given by Couto et al.^74^, who used segment-based and node-based methods to calculate the real contact area. Concerning mesh independence, the finite element model was verified with three mesh densities of 256, 512, and 1024 elements per direction based on previous work^71^. The real contact area shows a maximum difference of only 3.11% under different mesh densities, while the ABAQUS output (CAREA) shows 8.81%. For the finest mesh with m = 1024, the difference between the two methods is only 2.66%. In this study, the mesh density of m = 1024 was adopted for all calculations to ensure solution accuracy.

The mechanical parameters for the Johnson-Cook constitutive model are based on aluminum, with the following values now explicitly stated here : initial yield stress A = 324 MPa, hardening constant B = 114 MPa, strain hardening exponent n = 0.42, and strain rate constant C = 0.002. The Young’s modulus is E = 70 GPa and Poisson’s ratio is ν = 0.33. The dimensionless yield strength Y/E ranges from 0.002 to 0.01, covering the parametric study requirements. The contact is solved using the penalty function method with appropriate penalty stiffness scaling. To address uncertainty, five realizations are performed for each loading case under the deterministic power spectral density to account for the randomness inherent in the generated rough surfaces. This approach ensures the reliability and statistical significance of the results.

Further, the finite element simulations of contact mechanics are conducted using ABAQUS/2021. Employing a fractal surface defined by a specific power spectral density, the Johnson-Cook constitutive model and penalty-function contact algorithm are implemented to resolve elastoplastic deformation within the asperity hierarchy. Asperity deformation, governed by plasticity-modified Hertzian contact theory, transitions from elastic to elastoplastic and ultimately fully plastic states when normal load exceeds the material yield limit^75^. The plasticity index ψ dictates regime transitions (Eq. (5)):

5 $${\psi }_{i}=\frac{{E}^{*}}{H}\sqrt{\frac{\sigma }{{R}_{i}}}$$

where H denotes material hardness, ${R}_{i}$ the asperity radius of curvature, and σ the root-mean-square roughness. The fully plastic regime adheres to the Johnson-Cook hardening law^76^, governed by (Eq. (6)):

6 $${\sigma }_{y}=(A+B{\varepsilon }_{p}^{n})(1+C{\mathrm{ln}}{\varepsilon }^{*})$$

where ${\sigma }_{y}$ denotes equivalent yield stress, A the initial yield stress, B the hardening constant, and C the strain rate constant, ${\varepsilon }_{p}^{n}$ represents equivalent plastic strain, and ${\varepsilon }^{*}$ the normalized strain rate.

The contact constraint is resolved via the penalty function method, transforming the constrained problem into an unconstrained minimization of the total potential energy Π, which comprises material deformation energy and contact penalty energy (Eq. (7)):

7 $$\varPi={\int }_{\!\!\!\!\varOmega }\sigma :d\varepsilon d\varOmega+{\int }_{{\!\!\!\!\varGamma }_{c}}\frac{1}{2}{K}_{n}{\delta }^{2}d\varGamma$$

where the total potential energy Π includes contributions from the Cauchy stress tensor σ and the infinitesimal strain tensor ε; the normal penalty stiffness ${K}_{n}$ corresponds to the interfacial spring stiffness per unit area; and the normal penetration depth δ is the signed distance from a node to the master surface. The penalty stiffness ${K}_{n}$ scales with material properties and discretization resolution as (Eq. (8))^77^:

8 $${K}_{n}=\alpha \cdot \frac{{E}^{*}}{h}$$

where α denotes a dimensionless stiffness scaling factor, ${E}^{*}$ the equivalent elastic modulus, and h the characteristic element size. Optimal α selection enforces $\delta \le 0.01h$, eliminating numerical instabilities from ill-conditioned stiffness matrices.

### Molecular dynamics

The initial polyimide substrate is built at the atomic level using the Moltemplate software, consisting of 25 linear chains with a degree of polymerization of 4, totaling 3500 atoms. The structural optimization is performed via polymer consistent force field-based classical molecular dynamics^51^ for 500 ps under the NPT ensemble at 298.15 K and 0.1 MPa to initialize the system for ReaxFF-based reactive simulations. Pressure regulation is achieved via the Parrinello–Rahman barostat^78^, set at 1 bar, with a 1.0 ps coupling time and a compressibility of 2 × 10⁻⁵ bar⁻¹. Semi-isotropic pressure coupling is applied to maintain a tension-free polymer interface. Before conducting simulations of the running-in process, the structural optimization for the PI is performed by classical molecular dynamics for 500 ps to minimize system energy and ensure structural stability. Subsequently, to simulate a realistic friction process, a virtual aluminum (Al) substrate is applied to interact with the entire polyimide bulk model. The Al (111) surface is modeled by cleaving a 2-layer slab from the bulk FCC aluminum (a = 4.038 Å). A vacuum spacing of 15 Å is introduced normal to the surface to decouple periodic slabs. During geometry optimization, the entire Al (111) slab is held fixed to simulate a rigid surface, and only the adsorbed molecule was allowed to relax. A total of 5 × 10⁶ iterations (equivalent to 250 ps) are performed at the target temperature to ensure a complete pyrolysis reaction via ReaxFF-molecular dynamics under NVT ensemble. In all simulations, a time step of 0.5 fs was used for numerical integration, and all covalent bonds involving hydrogen atoms are constrained using the LINCS algorithm^79^ to allow stable and efficient simulations. Each simulation employs a Berendsen thermostat with a damping constant of 100 fs for temperature control.

ReaxFF reactive molecular dynamics^50^ is employed in the simulations of the DMSO-assisted running-in process for 800 ps using the LAMMPS software^80^. Throughout the production simulations, the system is maintained at a constant temperature of 300 K using the V-rescale thermostat^81^, employing a time constant of 0.1 ps for thermal coupling. Packmol is used to randomly pack DMSO solvent molecules above the polymer slab under spatial constraints, ensuring physically plausible initial configurations. The time evolution of DMSO infiltration on the polymer surface with different bond breakages is observed. Within polymer consistent force field, bonded interactions (bond stretching, angle bending, dihedrals) used harmonic and Ryckaert-Bellemans forms as appropriate, while nonbonded van der Waals forces employed a 9–6 Lennard-Jones potential (Eq. (9)):

9 $$\phi \left({r}_{{ij}}\right)={\varepsilon }_{{ij}}\left[2{\left(\frac{{\sigma }_{{ij}}}{{r}_{{ij}}}\right)}^{9}-3{\left(\frac{{\sigma }_{{ij}}}{{r}_{{ij}}}\right)}^{6}\right]$$

Atomic partial charges for both polyimide and DMSO are assigned according to standard PCFF parameters for each atom type. Long-range electrostatic interactions are handled via the particle-particle particle-mesh algorithm with an appropriate cutoff of 14 Å. This force-field assignment ensures a consistent treatment of polar interactions between the polymer and the DMSO solvent. Each system underwent a 10 ns simulation, and trajectory data are collected at 1 ps intervals. Post-processing and visualization of the trajectories are carried out using VMD and OVITO software packages^82^. To ensure numerical stability of the reactive simulations, a small time step of 0.1 fs is adopted to accurately capture bond-breaking and formation. The system temperature is controlled using the Berendsen thermostat with a damping constant of 100 fs.

### Density functional theory

Density functional theory is utilized as implemented in the Vienna Ab initio simulation package in the calculations^83^. The exchange-correlation potential is described by using the generalized gradient approximation of Perdew-Burke-Ernzerhof. The projector augmented-wave method is employed to treat interactions between ion cores and valence electrons^84^. The plane-wave cutoff energy was fixed to 450 eV. Given structural models were relaxed until the Hellmann-Feynman forces were smaller than −0.02 eV/Å and the change in energy smaller than 10^−5 ^eV was attained. Grimme’s DFT-D3 methodology was used to describe the dispersion interactions among all the atoms in adsorption models. An implicit solvation model was employed to account for solvent effects of hexadecane, with a dielectric constant of 2.05 used to describe the solvent environment.

### Calculation of charge density

The calculation method of surface charge needs to first determine the number of molecules on the surface, the space-occupied volume of the single atom, and the surface charge of the single atom. The number of molecules (Eq. (10))^85,86^:

10 $$N=\frac{m}{M}\cdot {N}_{A}$$

*N* ~ the number of molecules, *m* ~ the mass of PI, *M* is the molar mass, which is ~766.7 g/mol according to the molecular configuration in Fig. 4f, and *N*_*A*_ ~ the Avogadro’s number (~6.022 × 10^23 ^mol^−1^). Accordingly, the space-occupied volume of the single atom (Eq. (11))^87,88^:

11 $${V}_{{atom}}=\frac{{V}_{{mol}}}{n}=\frac{{V}_{{total}}}{N\cdot n}=\frac{\frac{m}{\rho }}{N\cdot n}$$

*V*_*atom*_ ~ the space-occupied volume of the single atom, *V*_*mol*_ ~ the space-occupied volume of the single molecule, *V*_*total*_ ~ the total volume of PI, *n* ~ the atom number of the single molecule (~80), *ρ* ~ the density of PI (~1.45 g cm^−3^).

It is hypothesized that the space-occupied structure of the single atom is spherical or cubic in PI, and each atom in the PI molecule is involved in contact with a flat electrode. Thus, the contact areas can be obtained respectively in the case of the spherical and cube structures (Eq. (12))^89,90^:

12 $$\begin{array}{ccc}{A}_{{atom}}=\pi {r}^{2}=\pi \cdot {\left(\frac{3{V}_{{atom}}}{4\pi }\right)}^{\frac{2}{3}} & {{\rm{or}}} & {A}_{{atom}}={a}^{2}={\left({V}_{{atom}}\right)}^{\frac{2}{3}}\end{array}$$

where *A*_*atom*_ ~ the contact area of the single atom. The surface charge density (Eq. (13)):

13 $$\sigma=\frac{q}{{A}_{{atom}}}$$

*σ* ~ the surface charge density, *q* ~ the transferred charge of the single atom from the results of density functional theory (Fig. 4i). For example, the transferred charge is ~0.00134 e for the virgin PI without chain scission. Accordingly, the charge densities via above calculations are 3.6 mC m^−2^ and 4.4 mC m^−2^ for cases of spherical or cube structures, respectively.

## Supplementary information

Supplementary Information

Description of Additional Supplementary Files

Supplementary Movie 1

Supplementary Movie 2

Transparent Peer Review file

## Source data

Source Data

## Data Availability

All data supporting the results of this study are provided in this article and the Supplementary Materials. Any other information can be requested from the corresponding author. Source data are provided with this paper.

## References

[1] Stadlober, B., Zirkl, M. & Irimia-Vladu, M. Route towards sustainable smart sensors: ferroelectric polyvinylidene fluoride-based materials and their integration in flexible electronics. *Chem. Soc. Rev.* **48**, 1787–1825 (2019). 30776029 10.1039/c8cs00928g

[2] Wang, Z. L. On Maxwell’s displacement current for energy and sensors: the origin of nanogenerators. *Mater. Today* **20**, 74–82 (2017).

[3] Fan, F. R., Tian, Z. Q. & Wang, Z. L. Flexible triboelectric generator!. *Nano Energy* **1**, 328–334 (2012).

[4] Yang, P. et al. Radical anion transfer during contact electrification and its compensation for charge loss in triboelectric nanogenerator. *Matter* **6**, 18 (2023).

[5] He, W. C. et al. Boosting output performance of sliding mode triboelectric nanogenerator by charge space-accumulation effect. *Nat. Commun.* **11**, 8 (2020). 32848138 10.1038/s41467-020-18086-4 PMC7450049

[6] Xu, L., Bu, T. Z., Yang, X. D., Zhang, C. & Wang, Z. L. Ultrahigh charge density realized by charge pumping at ambient conditions for triboelectric nanogenerators. *Nano Energy* **49**, 625–633 (2018).

[7] Wu, J., Xi, Y. H. & Shi, Y. J. Toward wear-resistive, highly durable and high performance triboelectric nanogenerator through interface liquid lubrication. *Nano Energy* **72**, 9 (2020).

[8] Liu, D. et al. Standardized measurement of dielectric materials’ intrinsic triboelectric charge density through the suppression of air breakdown. *Nat. Commun.* **13**, 10 (2022). 36224185 10.1038/s41467-022-33766-z PMC9556570

[9] Wang, J. et al. An ultrafast self-polarization effect in barium titanate filled poly(vinylidene fluoride) composite film enabled by self-charge excitation triboelectric nanogenerator. *Adv. Funct. Mater.* **32**, 10 (2022).

[10] Wu, H. Y. et al. Ultrahigh output charge density achieved by charge trapping failure of dielectric polymers. *Energy Environ. Sci.* **16**, 2274–2283 (2023).

[11] Zhou, L. L. et al. Simultaneously enhancing power density and durability of sliding-mode triboelectric nanogenerator via interface liquid lubrication. *Adv. Energy Mater.* **10**, 9 (2020).

[12] Chung, S. H. et al. Nonpolar liquid lubricant submerged triboelectric nanogenerator for current amplification via direct electron flow. *Adv. Energy Mater.* **11**, 8 (2021).

[13] Zhao, J. & Shi, Y. J. Boosting the durability of triboelectric nanogenerators: a critical review and prospect. *Adv. Funct. Mater.* **33**, 42 (2023).

[14] Lu, P., Powrie, H. E., Wood, R. J. K., Harvey, T. J. & Harris, N. R. Early wear detection and its significance for condition monitoring. *Tribol. Int.* **159**, 10 (2021).

[15] Berman, D., Deshmukh, S. A., Sankaranarayanan, S., Erdemir, A. & Sumant, A. V. Macroscale superlubricity enabled by graphene nanoscroll formation. *Science* **348**, 1118–1122 (2015). 25977372 10.1126/science.1262024

[16] Li, P. P. et al. Toward robust macroscale superlubricity on engineering steel substrate. *Adv. Mater.* **32**, 8 (2020). 10.1002/adma.202002039 32715515

[17] Zhang, C. L. et al. Surface charge density of triboelectric nanogenerators: theoretical boundary and optimization methodology. *Appl. Mater. Today* **18**, 8 (2020).

[18] Zhao, Z. H. et al. Selection rules of triboelectric materials for direct-current triboelectric nanogenerator. *Nat. Commun.* **12**, 8 (2021). 34344892 10.1038/s41467-021-25046-z PMC8333059

[19] Fu, J. J. et al. Achieving ultrahigh output energy density of triboelectric nanogenerators in high-pressure gas environment. *Adv. Sci.* **7**, 8 (2020). 10.1002/advs.202001757 PMC7740098 33344120

[20] Chung, J. et al. Dielectric liquid-based self-operating switch triboelectric nanogenerator for current amplification via regulating air breakdown. *Nano Energy* **88**, 8 (2021).

[21] Liu, Z. Q. et al. Fabrication of triboelectric polymer films via repeated rheological forging for ultrahigh surface charge density. *Nat. Commun.* **13**, 10 (2022). 35835779 10.1038/s41467-022-31822-2 PMC9283396

[22] Blau, P. J. On the nature of running-in. *Tribol. Inter.* **38**, 1007–1012 (2005).

[23] Zhao, J. et al. Real-time and online lubricating oil condition monitoring enabled by triboelectric nanogenerator. *Acs Nano* **15**, 11869–11879 (2021). 34170109 10.1021/acsnano.1c02980 PMC8320232

[24] Yoo, D., Jang, S., Cho, S., Choi, D. & Kim, D. S. A liquid triboelectric series. *Adv. Mater.* **35**, 11 (2023). 10.1002/adma.202300699 36947827

[25] Wang, J. et al. Achieving ultrahigh triboelectric charge density for efficient energy harvesting. *Nat. Commun.* **8**, 8 (2017). 28729530 10.1038/s41467-017-00131-4 PMC5519710

[26] Han, K. et al. Effects of environmental atmosphere on the performance of contact-separation mode TENG. *Adv. Mater. Technol.* **4**, 8 (2019).

[27] Wang, S. H. et al. Molecular surface functionalization to enhance the power output of triboelectric nanogenerators. *J. Mater. Chem. A* **4**, 3728–3734 (2016).

[28] Lee, J. W. et al. High-output triboelectric nanogenerator based on dual inductive and resonance effects-controlled highly transparent polyimide for self-powered sensor network systems. *Adv. Energy Mater.* **9**, 11 (2019).

[29] Li, S. Y. et al. Manipulating the triboelectric surface charge density of polymers by low-energy helium ion irradiation/implantation. *Energy Environ. Sci.* **13**, 896–907 (2020).

[30] Wang, J. et al. Remarkably enhanced charge density of inorganic material via regulating contact barrier difference and charge trapping for triboelectric nanogenerator. *Adv. Funct. Mater.* **33**, 11 (2023).

[31] Wang, H. L., Guo, Z. H., Zhu, G., Pu, X. & Wang, Z. L. Boosting the power and lowering the impedance of triboelectric nanogenerators through manipulating the permittivity for wearable energy harvesting. *ACS Nano* **15**, 7513–7521 (2021). 33856770 10.1021/acsnano.1c00914

[32] Bhatta, T. et al. Siloxene/PVDF composite nanofibrous membrane for high-performance triboelectric nanogenerator and self-powered static and dynamic pressure sensing applications. *Adv. Funct. Mater.* **32**, 15 (2022).

[33] He, W. C. et al. Capturing dissipation charge in charge space accumulation area for enhancing output performance of sliding triboelectric nanogenerator. *Adv. Energy Mater.* **12**, 9 (2022).

[34] Yu, B., Yu, H., Huang, T., Wang, H. Z. & Zhu, M. F. A biomimetic nanofiber-based triboelectric nanogenerator with an ultrahigh transfer charge density. *Nano Energy* **48**, 464–470 (2018).

[35] Wang, K. Q. et al. Hexadecane-containing sandwich structure based triboelectric nanogenerator with remarkable performance enhancement. *Nano Energy* **87**, 8 (2021).

[36] Niu, S. M. et al. Theoretical study of contact-mode triboelectric nanogenerators as an effective power source. *Energy Environ. Sci.* **6**, 3576–3583 (2013).

[37] Luo, Y. J. et al. Advanced energy harvesting from low-frequency ocean waves for lithium-ion battery applications. *Energy Environ. Sci.* **18**, 4821–4832 (2025).

[38] Gao, Y. K. et al. Spontaneously established reverse electric field to enhance the performance of triboelectric nanogenerators via improving Coulombic efficiency. *Nat. Commun.* **15**, 11 (2024). 38755131 10.1038/s41467-024-48456-1 PMC11099027

[39] Liu, J. J. et al. Nonaqueous contact-electro-chemistry via triboelectric charge. *J. Am. Chem. Soc*. **146**, 31574–31584 (2024). 10.1021/jacs.4c09318 39527749

[40] Shao, Z. Z. & Vollrath, F. The effect of solvents on the contraction and mechanical properties of spider silk. *Polymer* **40**, 1799–1806 (1999).

[41] Hu, C. et al. Exploration on the tribological mechanisms of polyimide with different molecular structures in different temperatures. *Appl. Surf. Sci.* **560**, 10 (2021).

[42] Li, S. M., Zhou, Y. S., Zi, Y. L., Zhang, G. & Wang, Z. L. Excluding contact electrification in surface potential measurement using Kelvin probe force microscopy. *Acs Nano* **10**, 2528–2535 (2016). 26824304 10.1021/acsnano.5b07418

[43] Wang, Z. L. & Wang, A. C. On the origin of contact-electrification. *Mater. Today* **30**, 34–51 (2019).

[44] Sun, X. et al. Controlling the triboelectric properties and tribological behavior of polyimide materials via plasma treatment. *Nano Energy* **102**, 16 (2022).

[45] Liu, Y. Y. et al. Is the solvent activation strategy before heat treatment applicable to all reverse osmosis membranes? *J. Membr. Sci.* **665**, 10 (2023).

[46] Ishige, R., Song, C. L., Hara, S., Ando, S. & Kazarian, S. G. Analysis of spatial orientation distribution of highly oriented polyimide film using micro ATR-FTIR spectroscopic imaging method. *Polymer* **221**, 14 (2021).

[47] Wu, J., Wang, X. L., Li, H. Q., Wang, F. & Hu, Y. Q. First-principles investigations on the contact electrification mechanism between metal and amorphous polymers for triboelectric nanogenerators. *Nano Energy* **63**, 7 (2019).

[48] Zou, H. Y. et al. Quantifying the triboelectric series. *Nat. Commun.* **10**, 9 (2019). 30926850 10.1038/s41467-019-09461-x PMC6441076

[49] Wu, Y. L. & Wright, A. I. Why does thionating a carbonyl molecule make it a better electron acceptor? *Phys. Chem. Chem. Phys.* **25**, 1342–1348 (2023). 36537028 10.1039/d2cp05186a

[50] Wang, N. et al. Thermodynamic simulation of the RDX-aluminum interface using ReaxFF molecular dynamics. *J. Phys. Chem. C.* **121**, 14597–14610 (2017).

[51] Sun, H., Mumby, S. J., Maple, J. R. & Hagler, A. T. An ab initio CFF93 all-atom force field for polycarbonates. *J. Am. Chem. Soc.* **116**, 2978–2987 (1994).

[52] Liu, Y. N. et al. Flexible supercapacitors with high capacitance retention at temperatures from −20 to 100 °C based on DMSO-doped polymer hydrogel electrolytes. *J. Mater. Chem. A* **9**, 12051–12059 (2021).

[53] Zhou, S. R., Zhang, L., Zou, L., Ayubi, B. I. & Wang, Y. W. Mechanistic study of atomic oxygen erosion on polyimide under electric fields: a molecular dynamics and density functional theory approach. *Molecules* **29**, 11 (2024). 10.3390/molecules29225353 PMC11596869 39598742

[54] Marochkin, I. I. & Dorofeeva, O. V. Amide bond dissociation enthalpies: effect of substitution on N-C bond strength. *Comput. Theor. Chem.* **991**, 182–191 (2012).

[55] Li, Y. et al. Advanced dielectric materials for triboelectric nanogenerators: principles, methods, and applications. *Adv. Mater.* **36**, 53 (2024). 10.1002/adma.202314380 38517171

[56] Fumagalli, L., Ferrari, G., Sampietro, M. & Gomila, G. Quantitative nanoscale dielectric microscopy of single-layer supported biomembranes. *Nano Lett.* **9**, 1604–1608 (2009). 19271767 10.1021/nl803851u

[57] Fumagalli, L., Ferrari, G., Sampietro, M. & Gomila, G. Dielectric-constant measurement of thin insulating films at low frequency by nanoscale capacitance microscopy. *Appl. Phys. Lett.* **91**, 3 (2007).

[58] Houssat, M., Villeneuve-Faure, C., Dignat, N. L. & Cambronne, J. P. Nanoscale mechanical and electrical characterization of the interphase in polyimide/silicon nitride nanocomposites. *Nanotechnology* **32**, 13 (2021). 10.1088/1361-6528/ac13ea 34256368

[59] Qi, G. C. et al. Quantifying surface charge density by using an electric force microscope with a referential structure. *J. Phys. Chem. C.* **113**, 204–207 (2009).

[60] Qi, G. C. et al. Characteristic capacitance in an electric force microscope determined by using sample surface bias effect. *J. Appl. Phys.* **103**, 4 (2008).

[61] Sherwood, B. A. & Bernard, W. H. Work and heat-transfer in the presence of sliding friction. *Am. J. Phys.* **52**, 1001–1007 (1984).

[62] Wang, J. et al. Boosting the charge density of triboelectric nanogenerator by suppressing air breakdown and dielectric charge leakage. *Adv. Energy Mater.* **14**, 11 (2024).

[63] Heo, D. et al. Nano-oil-barrier-based fluttering triboelectric nanogenerator. *Adv. Sci.* **12**, 10 (2025). 10.1002/advs.202502278 PMC12376523 40391798

[64] Li, W. T. et al. Adsorption-induced surface potential modulation and electron transfer of solid-solid triboelectric nanogenerators under gas atmospheres. *Nano Energy* **143**, 12 (2025).

[65] Merlone, A. et al. Temperature extreme records: World Meteorological Organization metrological and meteorological evaluation of the 54.0 °C observations in Mitribah, Kuwait and Turbat, Pakistan in 2016/2017. *Int. J. Climatol.* **39**, 5154–5169 (2019).

[66] Wang, Z. L. On the first principle theory of nanogenerators from Maxwell’s equations. *Nano Energy* **68**, 10 (2020).

[67] Zi, Y. L. et al. Harvesting low-frequency (<5 Hz) irregular mechanical energy: a possible killer application of triboelectric nanogenerator. *Acs Nano* **10**, 4797–4805 (2016). 27077467 10.1021/acsnano.6b01569

[68] Gohar, R. & Cameron, A. Optical measurement of oil film thickness under elasto-hydrodynamic lubrication. *Nature* **200**, 458–459 (1963).

[69] Baytekin, H. T., Baytekin, B., Incorvati, J. T. & Grzybowski, B. A. Material transfer and polarity reversal in contact charging. *Angew. Chem. -Int. Ed.* **51**, 4843–4847 (2012). 10.1002/anie.201200057 22422707

[70] Hu, Y. Z. & Tonder, K. Simulation of 3-D random rough surface by 2-D digital filter and Fourier analysis. *Int. J. Mach. Tools Manuf.* **32**, 83–90 (1992).

[71] Zhang, H. B., Liu, S. L. & Wang, W. Z. On contact spots details of rough surface contact using morphologic image processing. *Mech. Mach. Theory* **192**, 21 (2024).

[72] Yastrebov, V. A., Anciaux, G. & Molinari, J. F. The role of the roughness spectral breadth in elastic contact of rough surfaces. *J. Mech. Phys. Solids* **107**, 469–493 (2017).

[73] Yastrebov, V. A., Anciaux, G. & Molinari, J. F. On the accurate computation of the true contact-area in mechanical contact of random rough surfaces. *Tribol. Inter.* **114**, 161–171 (2017).

[74] Carneiro, A. M. C., Carvalho, R. P. & Pires, F. M. A. Representative contact element size determination for micromechanical contact analysis of self-affine topographies. *Int. J. Solids Struct.* **206**, 262–281 (2020).

[75] Zhao, Y. W., Maietta, D. M. & Chang, L. An asperity microcontact model incorporating the transition from elastic deformation to fully plastic flow. *J. Tribol. -Trans. ASME* **122**, 86–93 (2000).

[76] Sirigiri, V. K. R., Gudiga, V. Y., Gattu, U. S., Suneesh, G. & Buddaraju, K. M. A review on Johnson Cook material model. In *13th International Conference on Materials, Processing and Characterization (ICMPC))*. SI edn (Elsevier, 2022).

[77] Pantano, A. & Averill, R. C. A penalty-based finite element interface technology. *Comput. Struct.* **80**, 1725–1748 (2002).

[78] Parrinello, M. & Rahman, A. Polymorphic transitions in single crystals: a new molecular dynamics method. *J. Appl. Phys.* **52**, 7182–7190 (1981).

[79] Hess, B., Bekker, H., Berendsen, H. J. C. & Fraaije, J. LINCS: a linear constraint solver for molecular simulations. *J. Comput. Chem.* **18**, 1463–1472 (1997).

[80] Thompson, A. P. et al. LAMMPS-a flexible simulation tool for particle-based materials modeling at the atomic, meso, and continuum scales. *Comput. Phys. Commun.* **271**, 34 (2022).

[81] Bussi, G., Donadio, D. & Parrinello, M. Canonical sampling through velocity rescaling. *J. Chem. Phys.* **126**, 7 (2007). 10.1063/1.2408420 17212484

[82] Humphrey, W., Dalke, A. & Schulten, K. V. M. D. Visual molecular dynamics. *J. Mol. Graph.* **14**, 33–38 (1996). 8744570 10.1016/0263-7855(96)00018-5

[83] Lyons, J. L. & Van de Walle, C. G. Computationally predicted energies and properties of defects in GaN. *npj Comput. Mater.* **3**, 10 (2017).

[84] Lee, M., Youn, Y., Yim, K. & Han, S. High-throughput ab initio calculations on dielectric constant and band gap of non-oxide dielectrics. *Sci. Rep.* **8**, 8 (2018). 30287929 10.1038/s41598-018-33095-6 PMC6172237

[85] Becker, P. History and progress in the accurate determination of the Avogadro constant. *Rep. Prog. Phys.* **64**, 1945–2008 (2001).

[86] Piacenza, G., Legsai, G., Blaive, B. & Gallo, R. Molecular volumes and densities of liquids and solids by molecular mechanics-estimation and analysis. *J. Phys. Org. Chem.* **9**, 427–432 (1996).

[87] Gavezzotti, A. The calculation of molecular volumes and the use of volume analysis in the investigation of structured media and of solid-state organic reactivity. *J. Am. Chem. Soc.* **105**, 5220–5225 (1983).

[88] Girolami, G. S. A simple “back of the envelope” method for estimating the densities and molecular volumes of liquids and solids. *J. Chem. Educ.* **71**, 962–964 (1994).

[89] Heyde, K. & Wood, J. L. Shape coexistence in atomic nuclei. *Rev. Mod. Phys.* **83**, 55 (2011).

[90] Bohr, N. On the constitution of atoms and molecules. *Philos. Mag.* **26**, 857–875 (1913).

