# Supplementary material for: Solvent-assisted running-in strategy enables improved triboelectric nanogenerator output
Source: Nat Commun. 2026 Jun 11;17:8971. doi: 10.1038/s41467-026-74269-5 (PMC13507328; doi:10.1038/s41467-026-74269-5)
Supplement: Supplementary file 1 [file 41467_2026_74269_MOESM1_ESM.pdf]

Supplementary Information

Solvent-assisted running-in strategy enables improved triboelectric nanogenerator output

Jun Zhao[1,*], Bin Ge[1], Xiangyu Feng[1], Yanjie Nie[2], Pengzhe Zhu[1], Yajie Zhang[2,*], Wei Wu[1], Xueyuan, Li[1], Yongfeng Wang[2], Yijun Shi[3,*]

[1] School of Mechanical Engineering, Beijing Institute of Technology, Beijing 100081, PR China

[2] Key Laboratory for the Physics and Chemistry of Nanodevices, School of Electronics, Peking University, Beijing 100871, China

[3] Division of Machine Elements, Luleå University of Technology, Luleå SE-971 87, Sweden

[*]Emails: zhaojun@bit.edu.cn; yjzhang11@pku.edu.cn; yijun.shi@lut.se

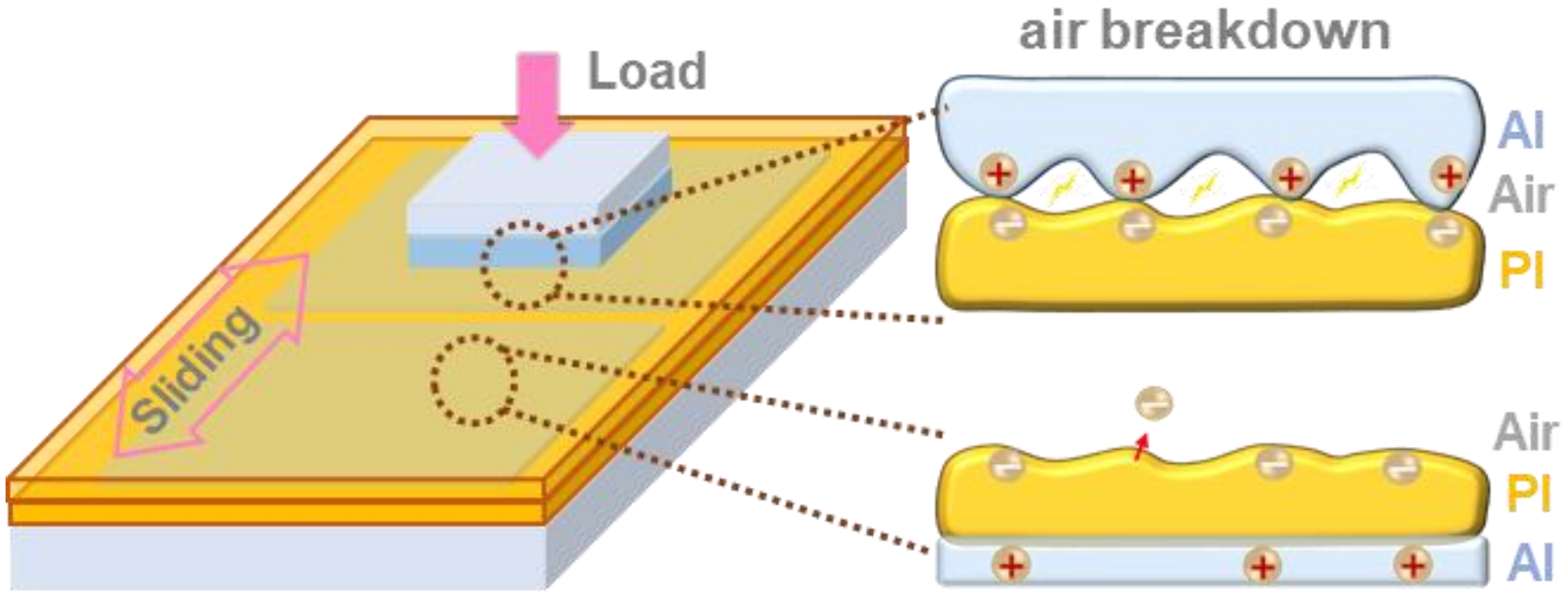

Figure S1 Triboelectrification mechanism diagram of the PI-Al friction pair under dry friction in air

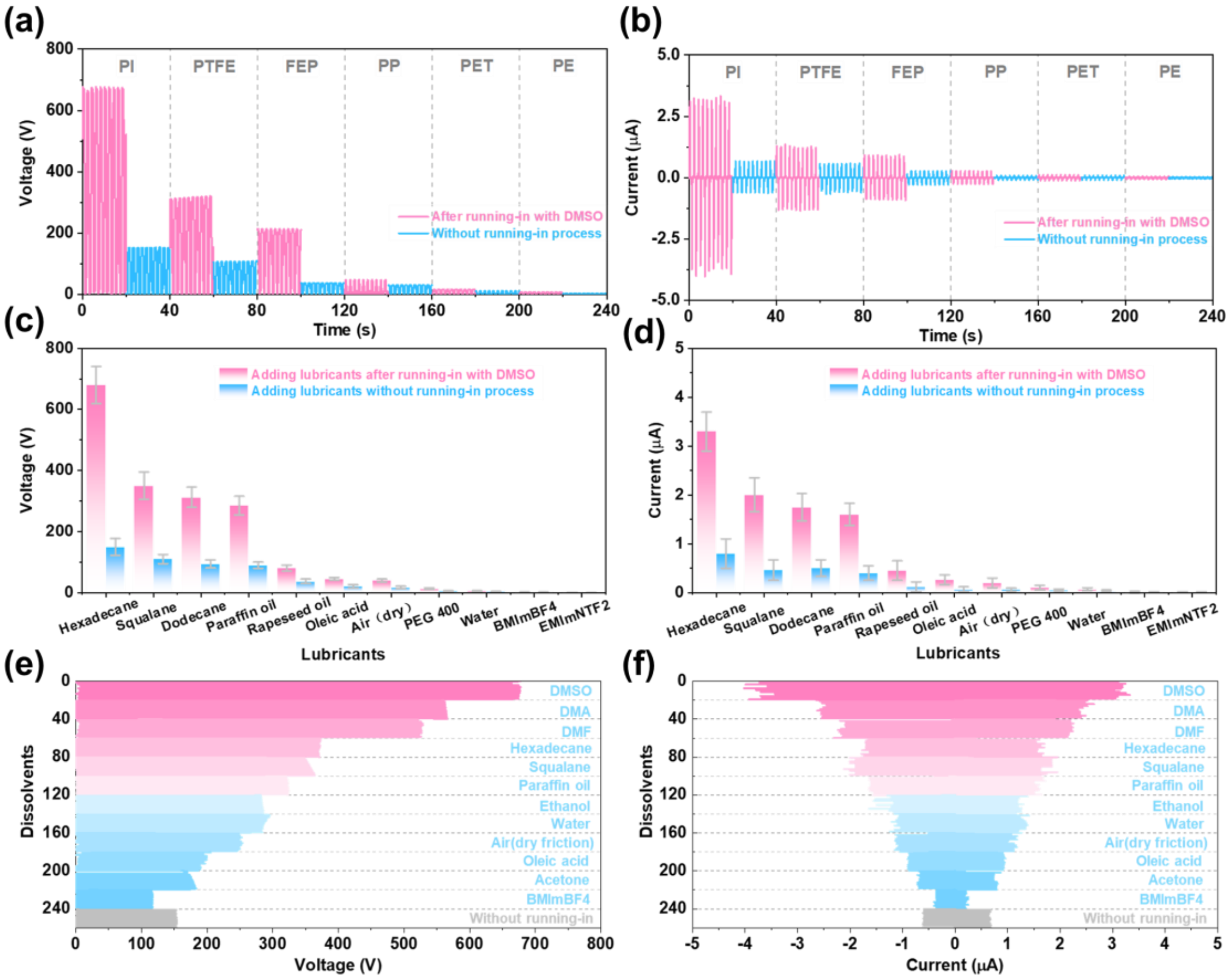

Figure S2 Output voltage (a) and current (b) of different engineering tribo-materials lubricated by hexadecane after running-in with DMSO. Output voltage (c) and current (d) of the engineering PI lubricated by different lubricants after running-in with DMSO. Output voltage (e) and current (f) of the engineering PI lubricated by hexadecane after running-in with different dissolvents.

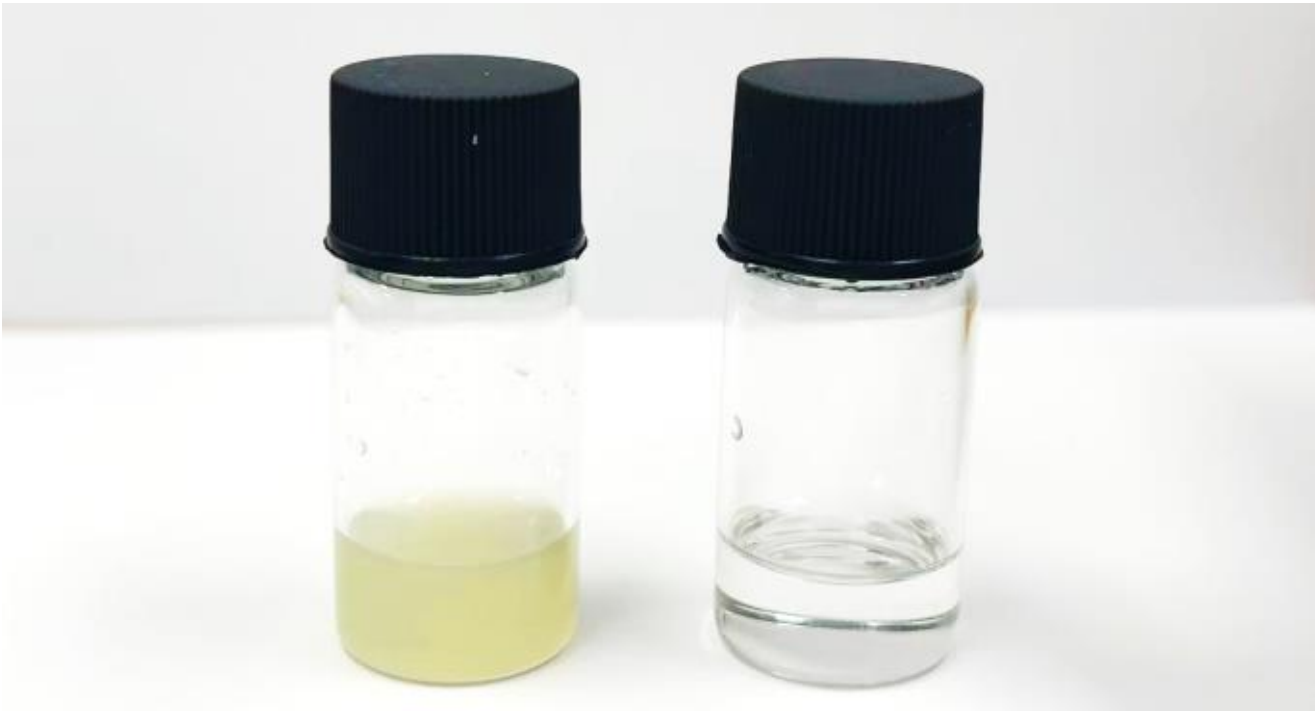

Figure S3 Pictures of the collected DMSO from the electric surface after running-in for 1000 cycles (at the left) compared with the virgin DMSO (at the right)

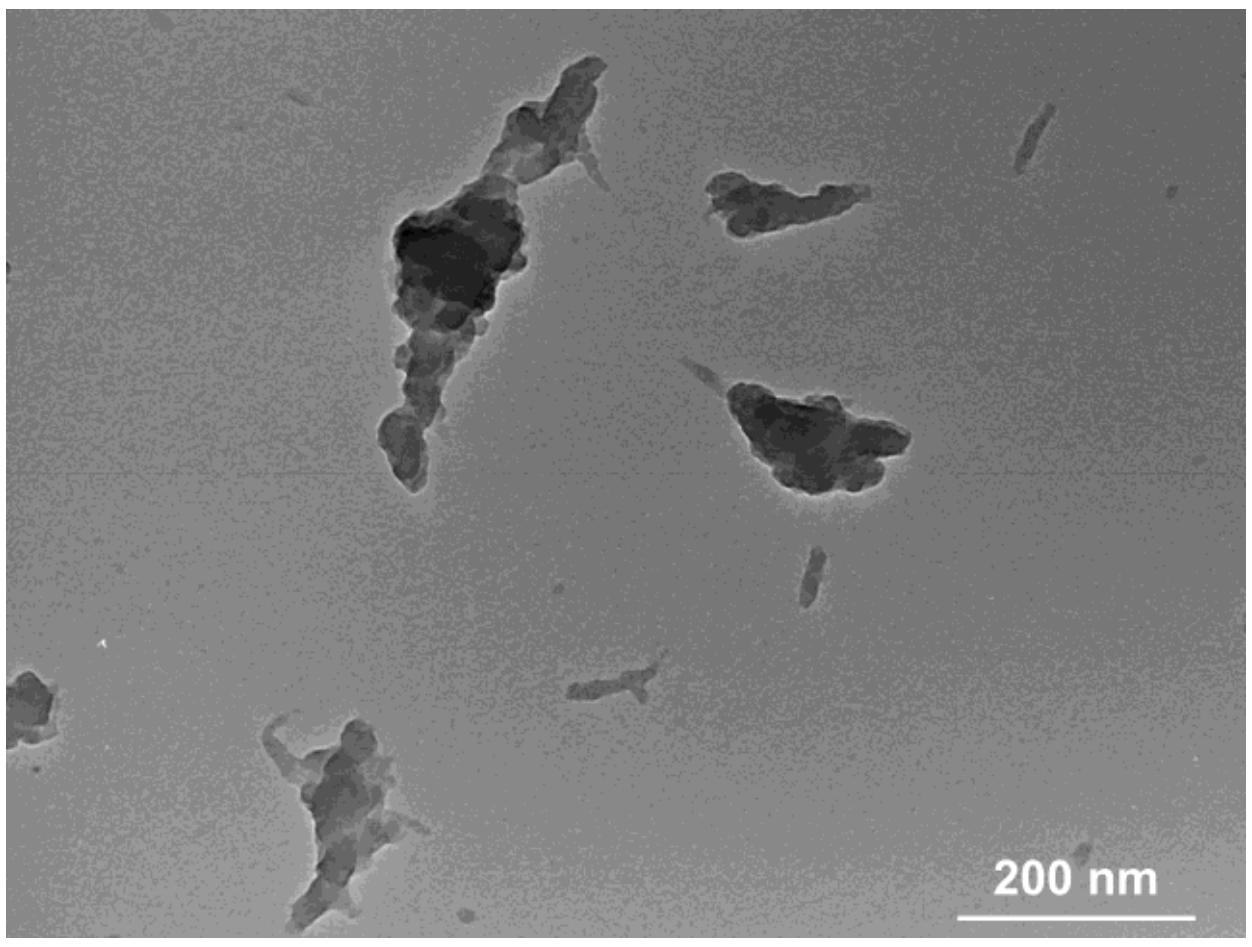

Figure S4 Transmission electron microscopy (TEM) image of the worn debris collected from the electric surface (from Figure S3)

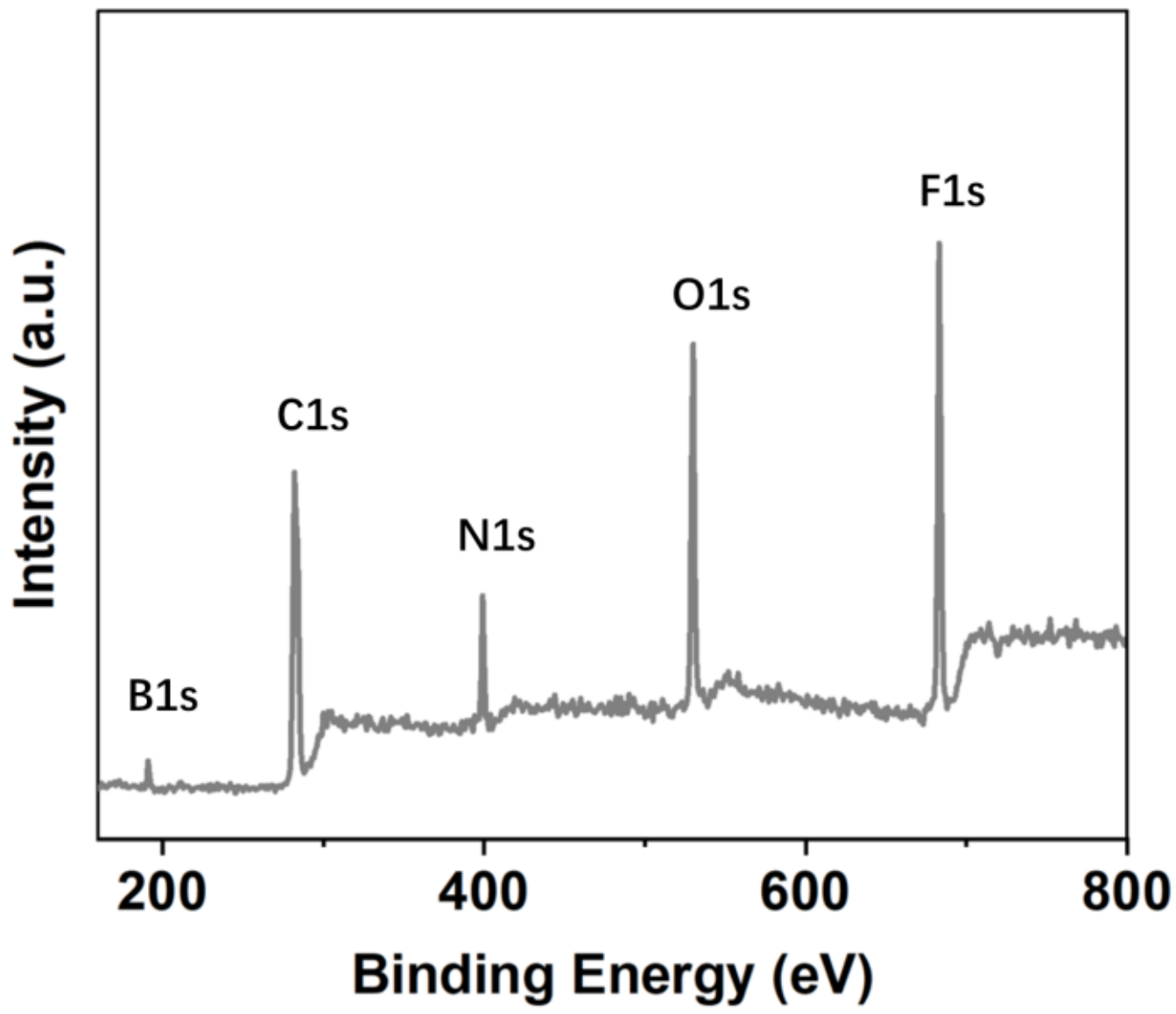

Figure S5 XPS survey spectra of the PI surfaces after running-in with $BMImBF_4$

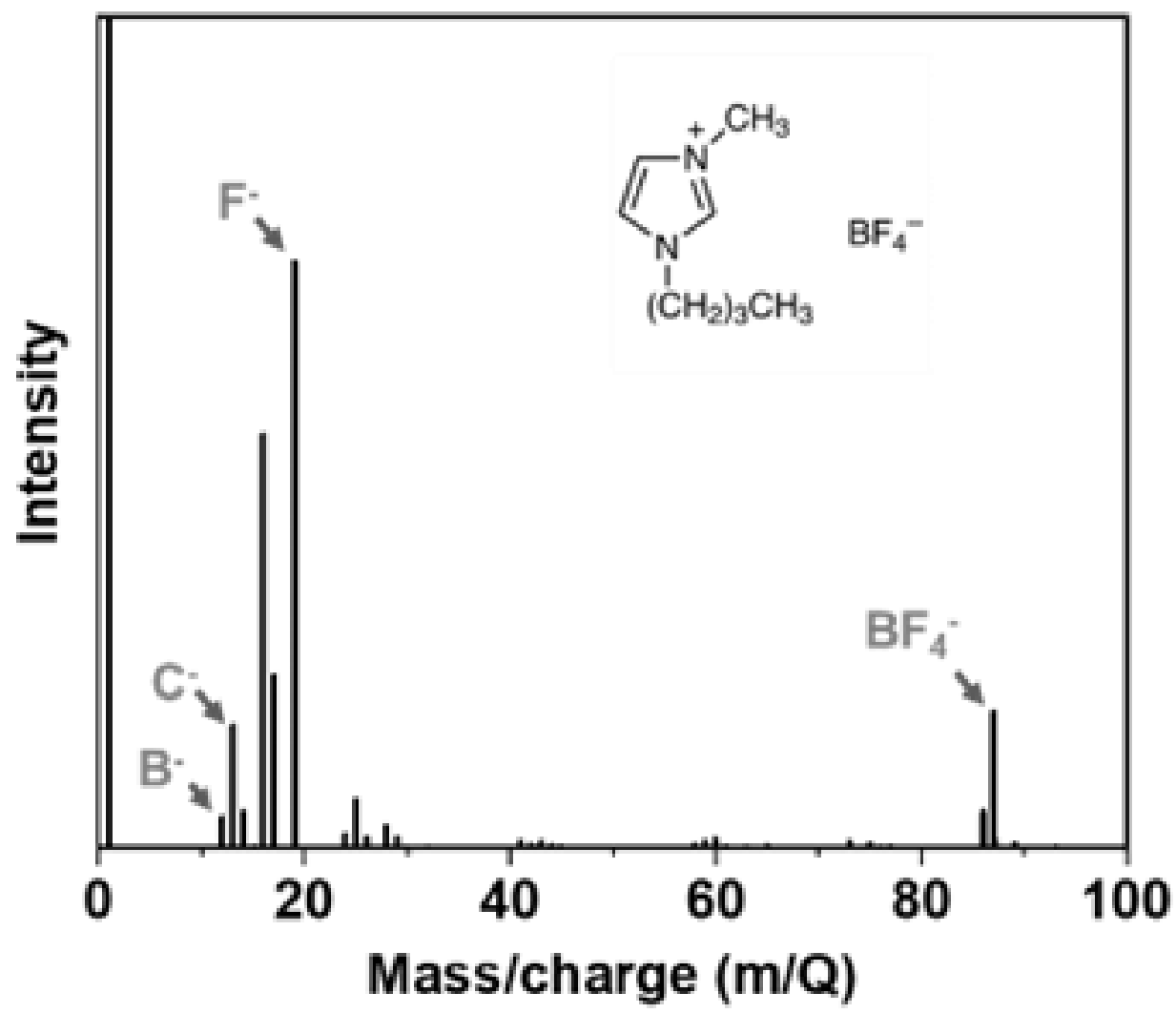

Figure S6 TOF-SIMS mass spectra of the anion on the PI surface after running-in process with $BMImBF_4$, inset showing the molecular structure of $BMImBF_4$

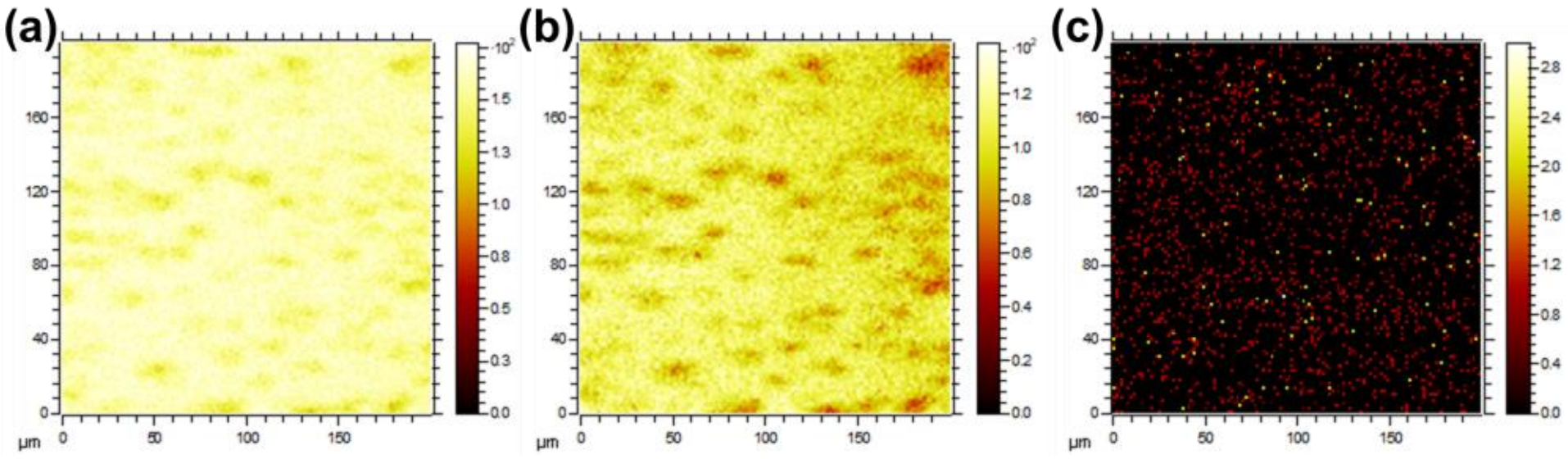

Figure S7 2D anion distribution images derived from the PI surface after running-in process with $BMImBF_4$: F^- (a), BF_4^- (b), and B^- (c)

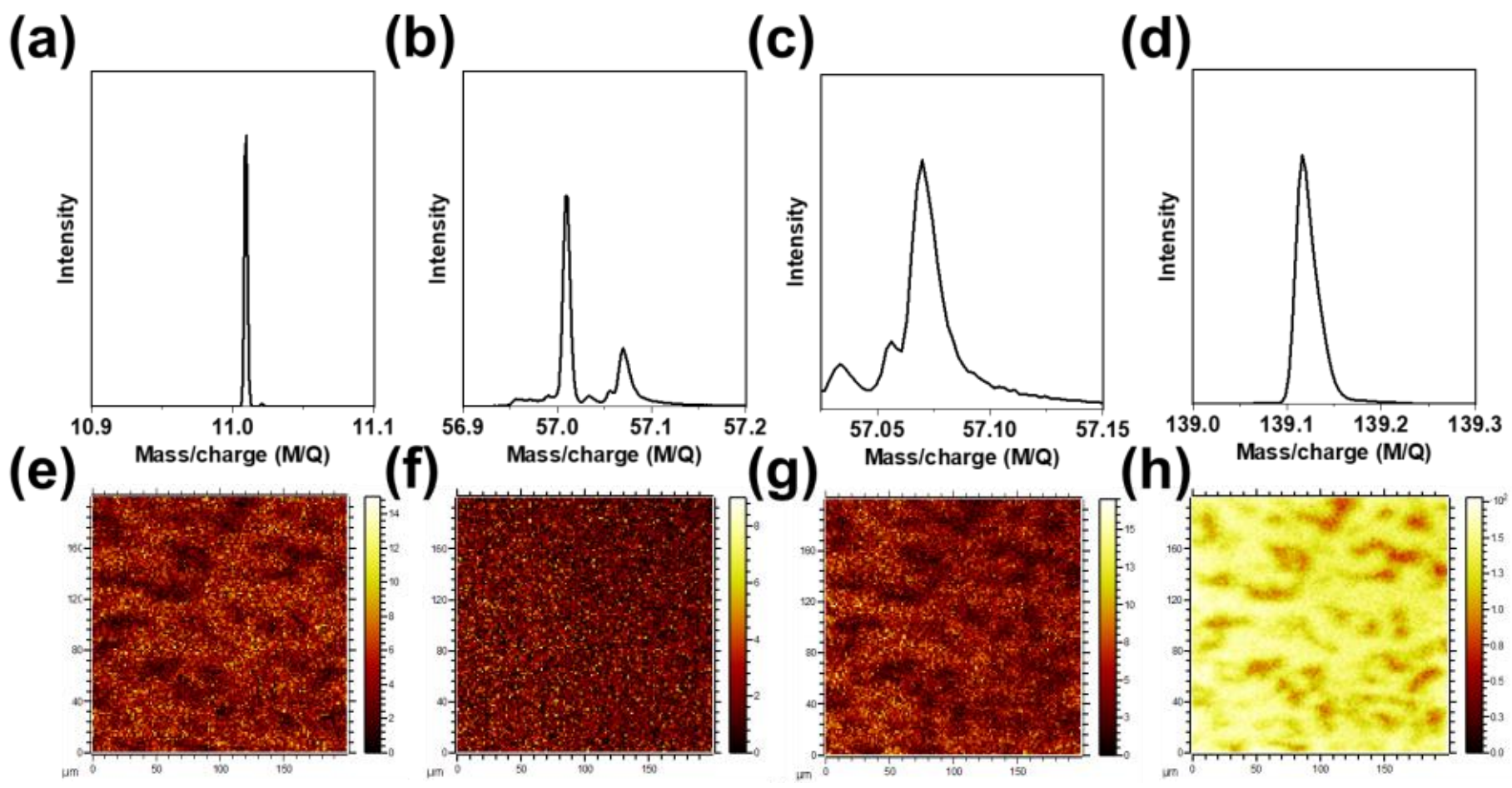

Figure S8 The TOF-SIMS mass spectra and 2D distribution images of the cation on the PI surface after running-in process with $BMImBF_4$: B^+ (a, e), $C_4H_9^+$ (b, f), $C_4H_9N_2^+$ (c, g), $C_8H_{15}N_2^+$ (d, h)

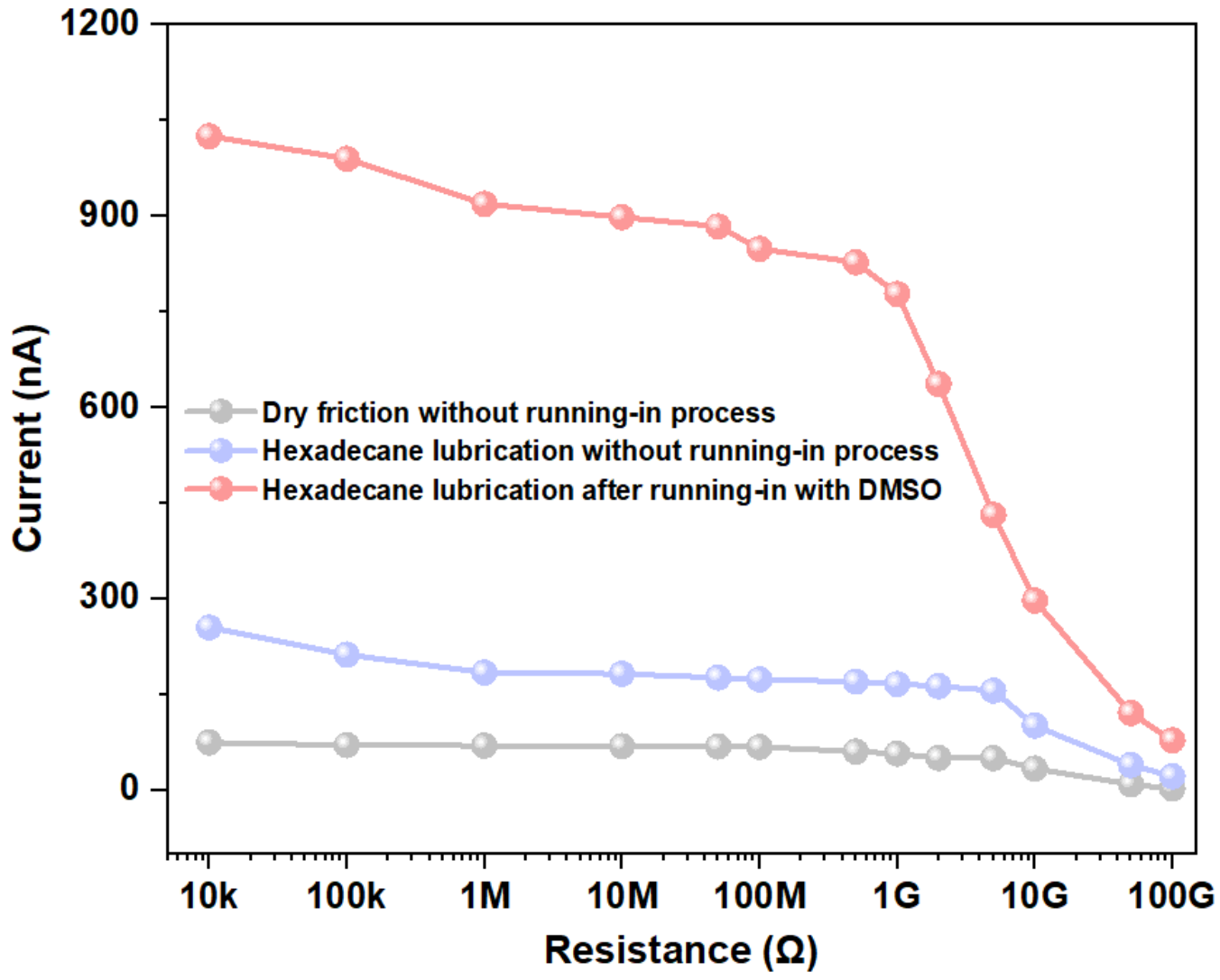

Figure S9 Output current under various external loads of the PI-based TENG.

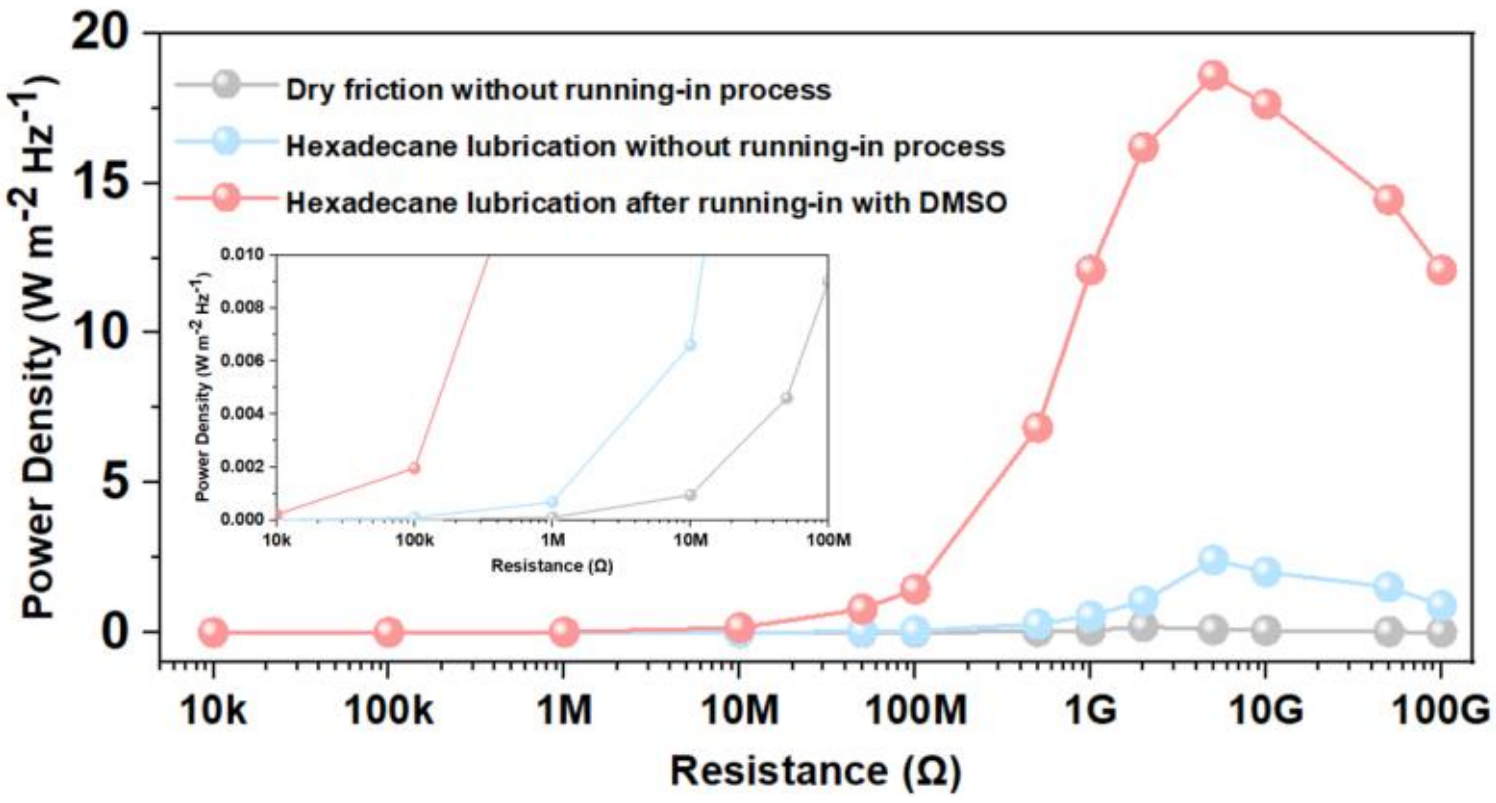

Figure S10 Output peak power density under various external loads of the PI-based TENG.

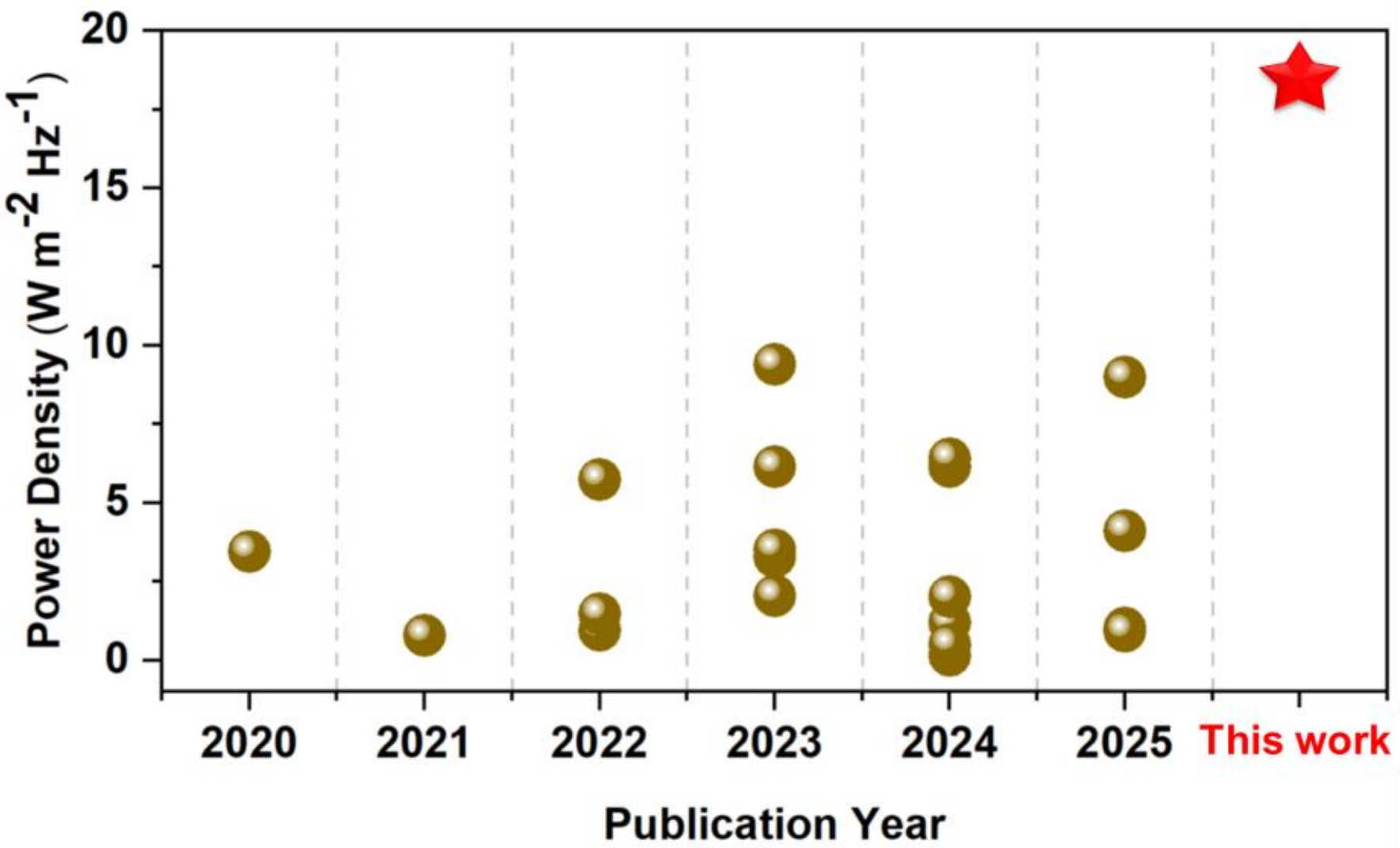

Figure S11 A comparison of the power density of TENGs in recent years[1-20]

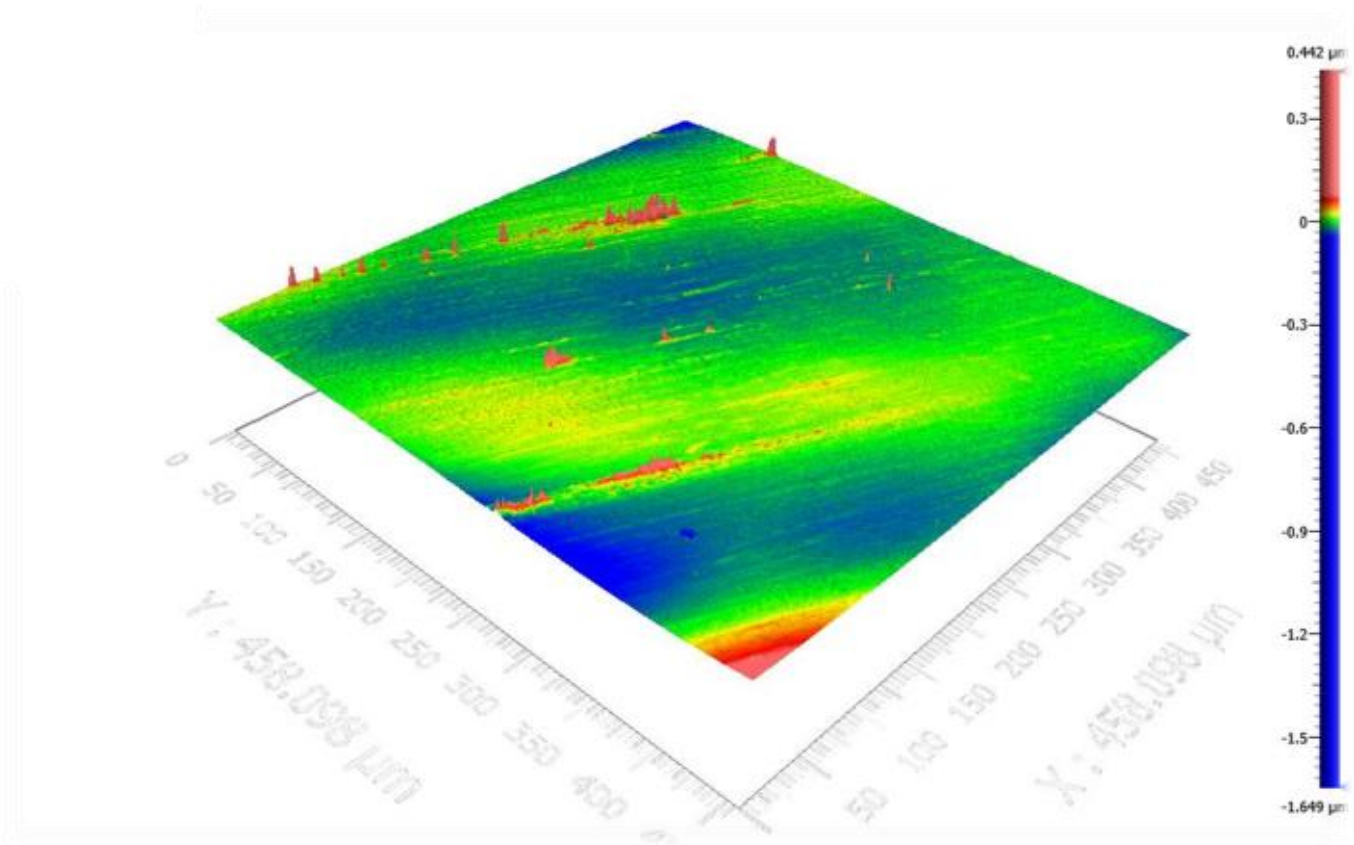

Figure S12 3D optical graph and cross-sectional depth profiles of the PI tribo-material with a surface roughness of 18 nm

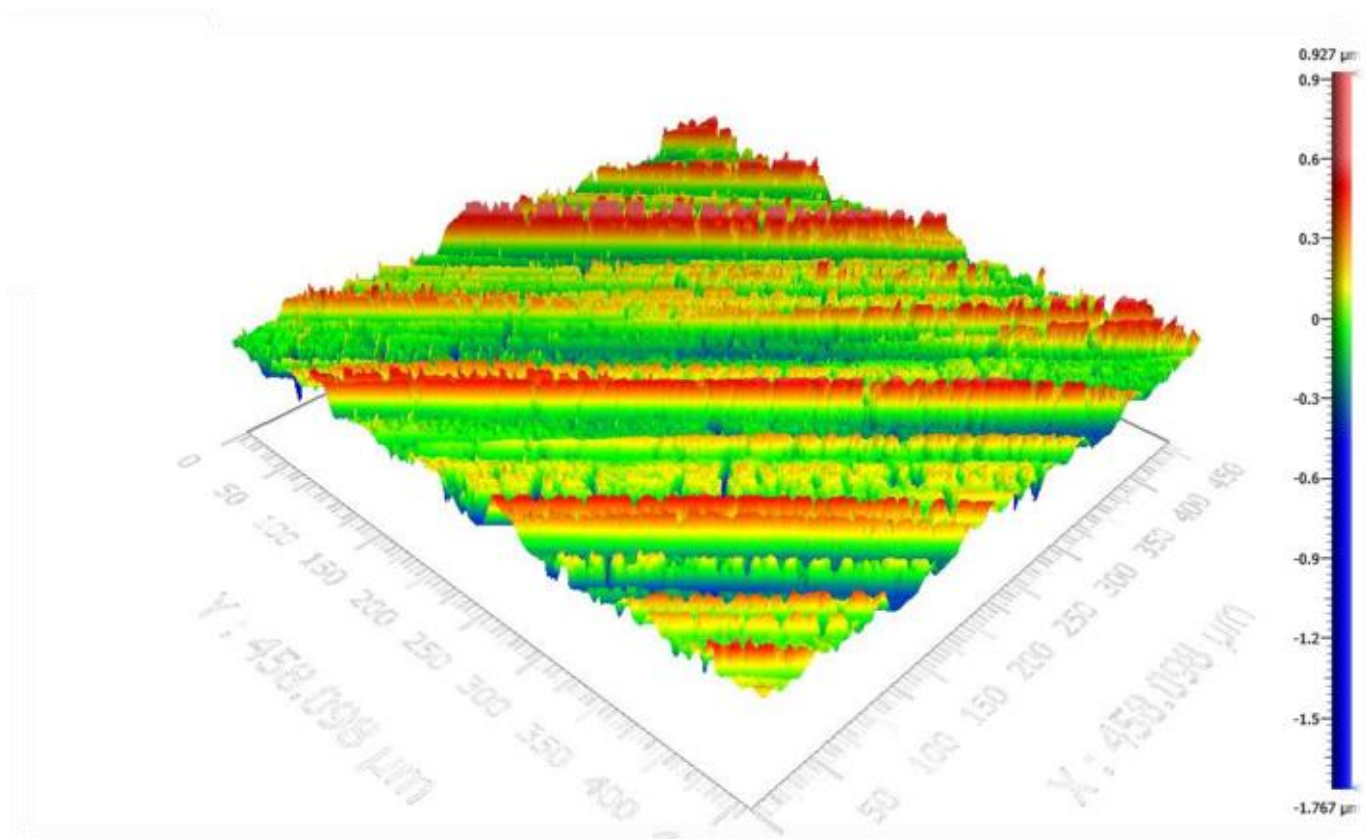

Figure S13 3D optical graph and cross-sectional depth profiles of the Al tribolayer with a surface roughness of 162 nm

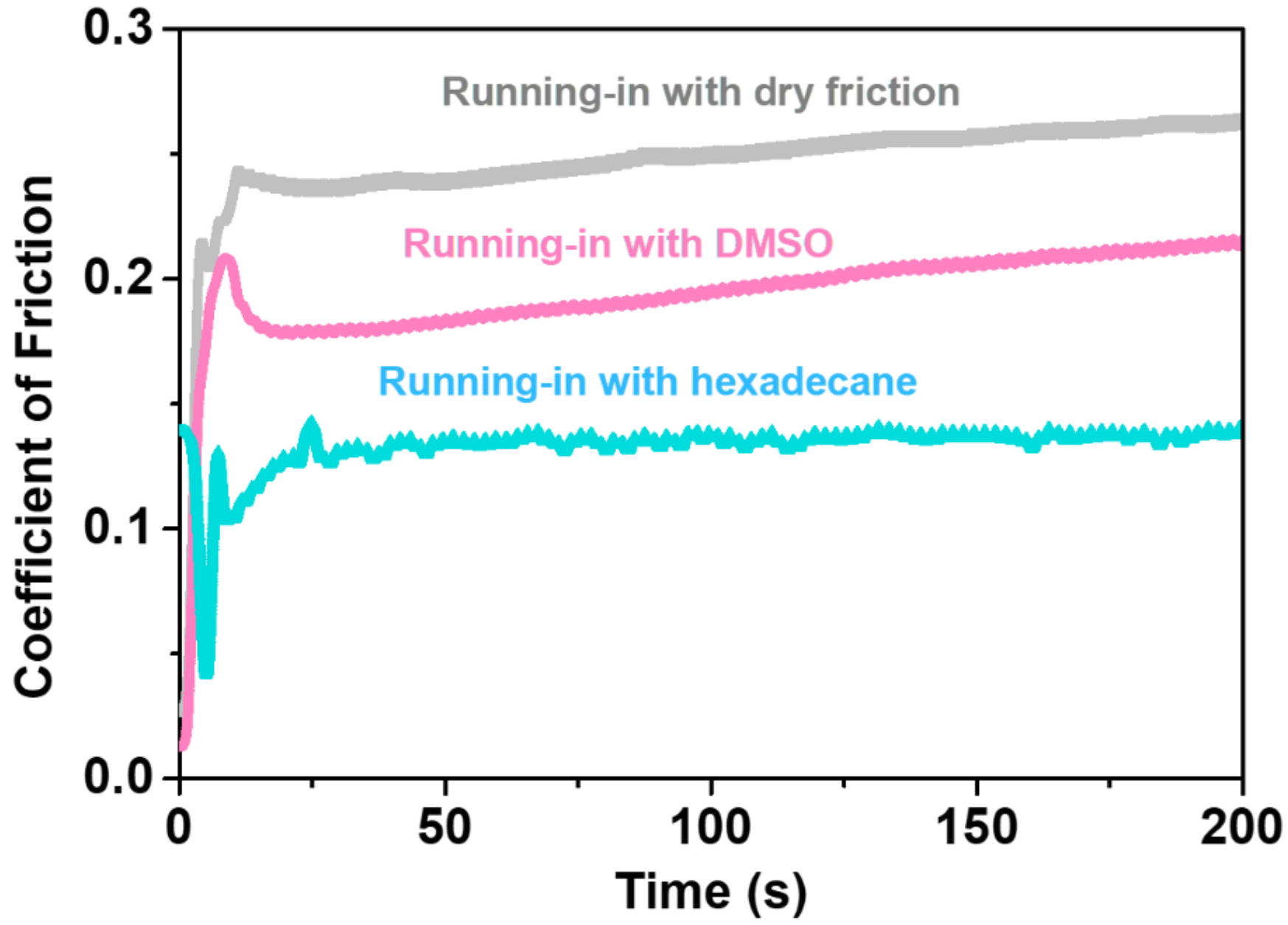

Figure S14 The coefficient of friction of TENGs during the running-in period

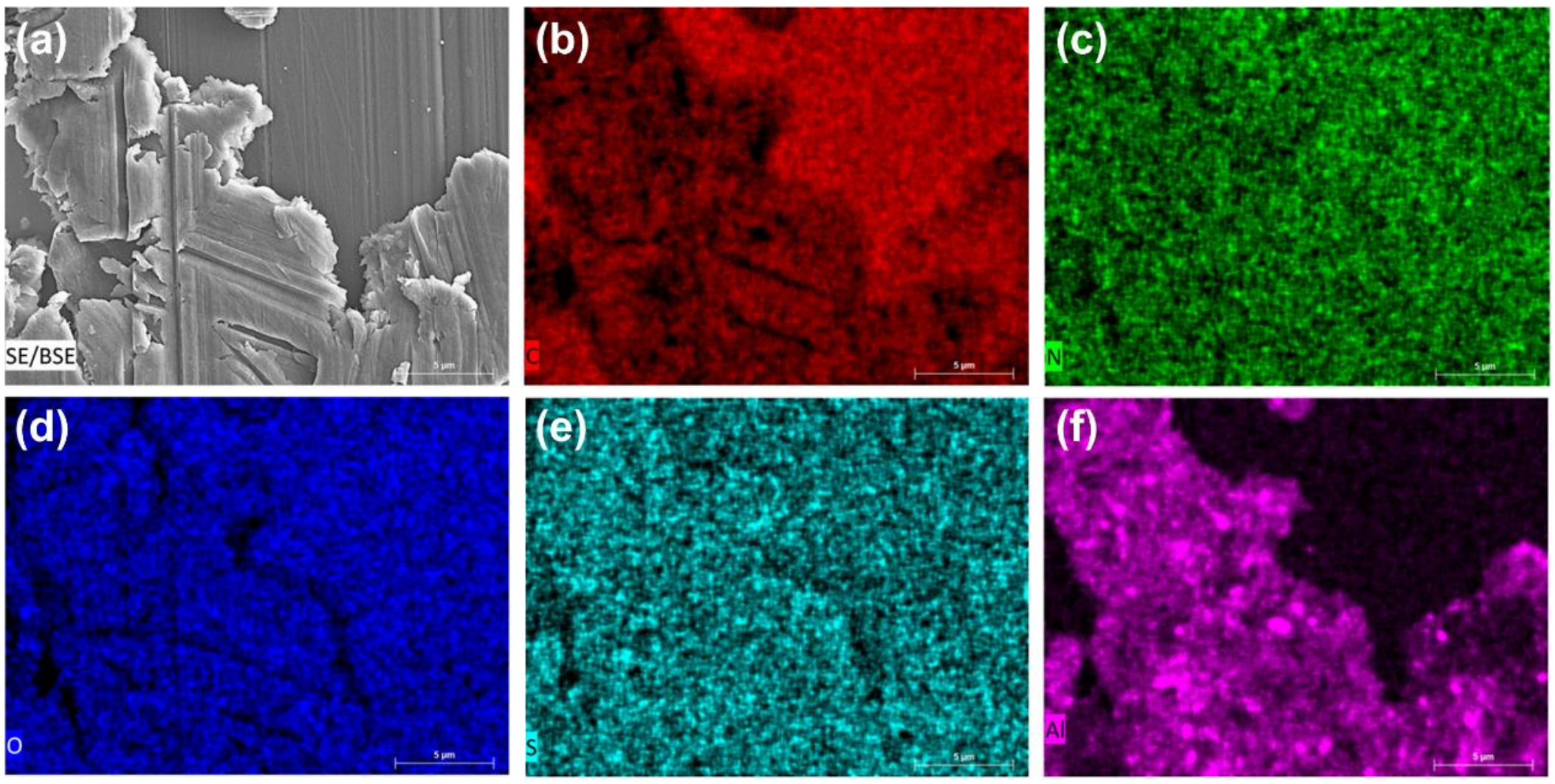

Figure S15 The SEM morphology of PI surface after running-in with dry friction (a), the EDS mapping analysis of the transfer materials: C(b), N(c), O(d), S(e), Al(f).

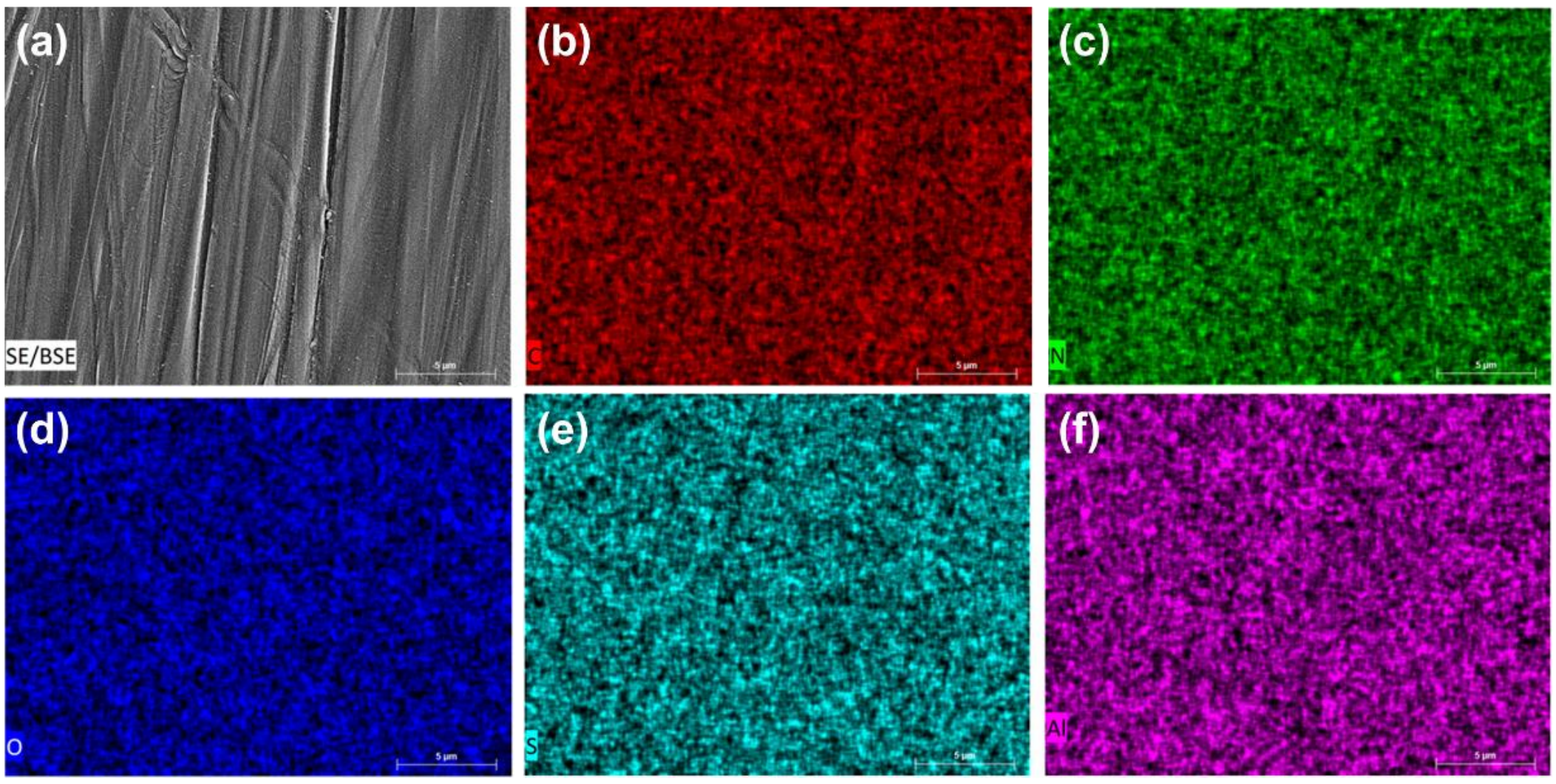

Figure S16 The SEM morphology of PI surface after running-in with DMSO (a), the EDS mapping analysis of the transfer materials: C (b), N (c), O (d), S (e), Al (f).

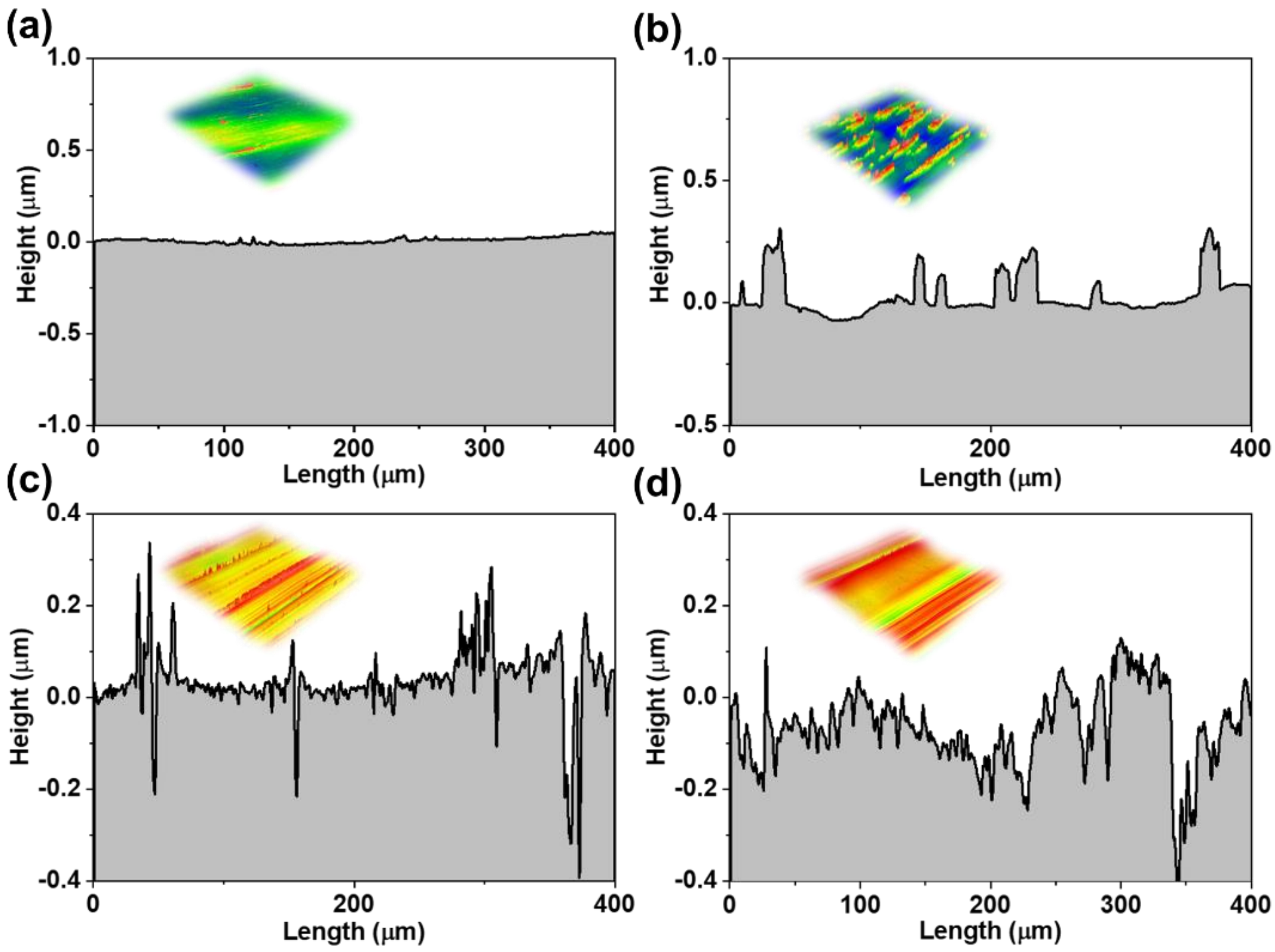

Figure S17 Cross-sectional depth profile of the virgin PI (a), the PI after running-in, respectively with dry friction (b), hexadecane (c), and DMSO (d), the insets showing the 3D optical images.

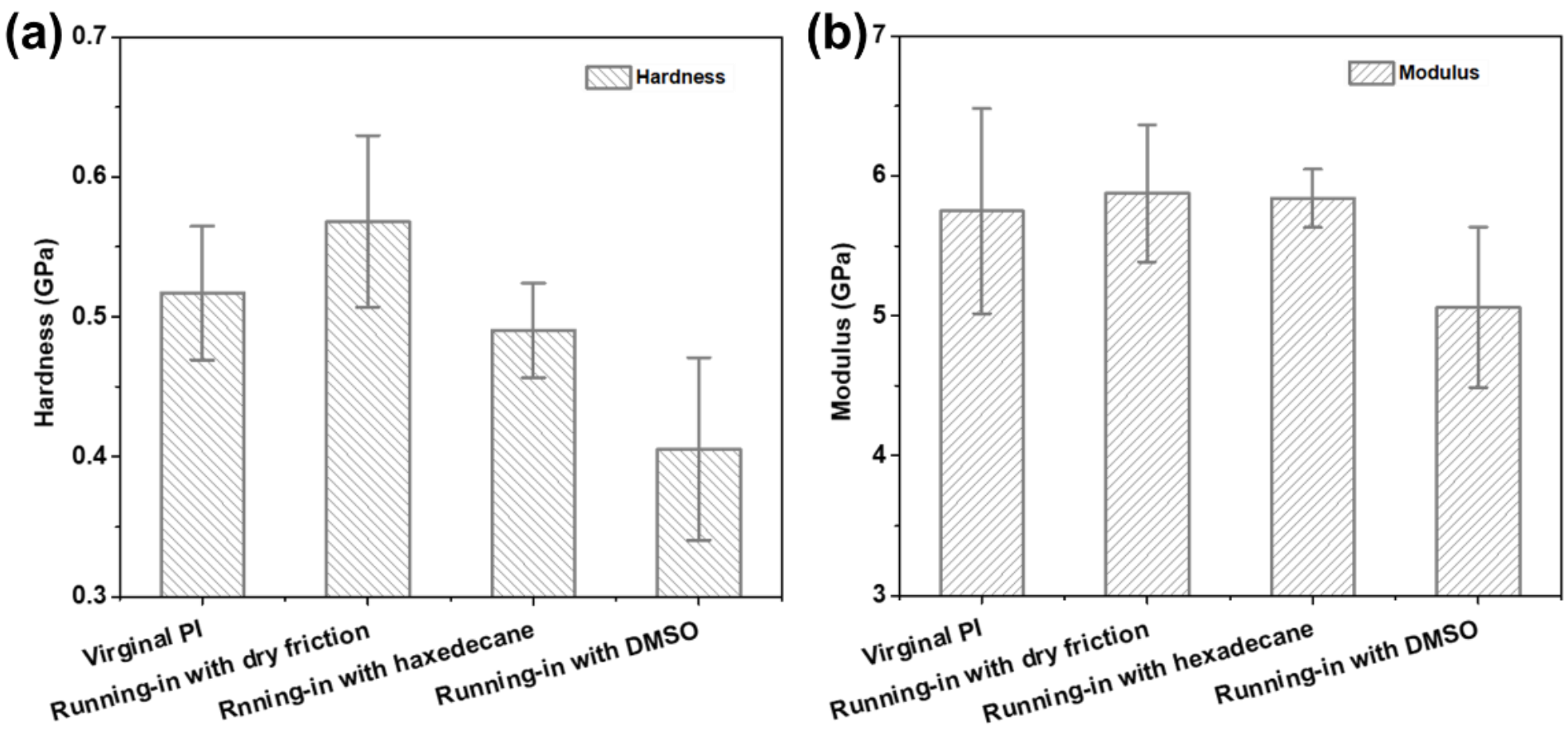

Figure S18 The hardness (a) and modulus (b) of the virgin PI, the PI after running-in with dry friction, hexadecane, and DMSO.

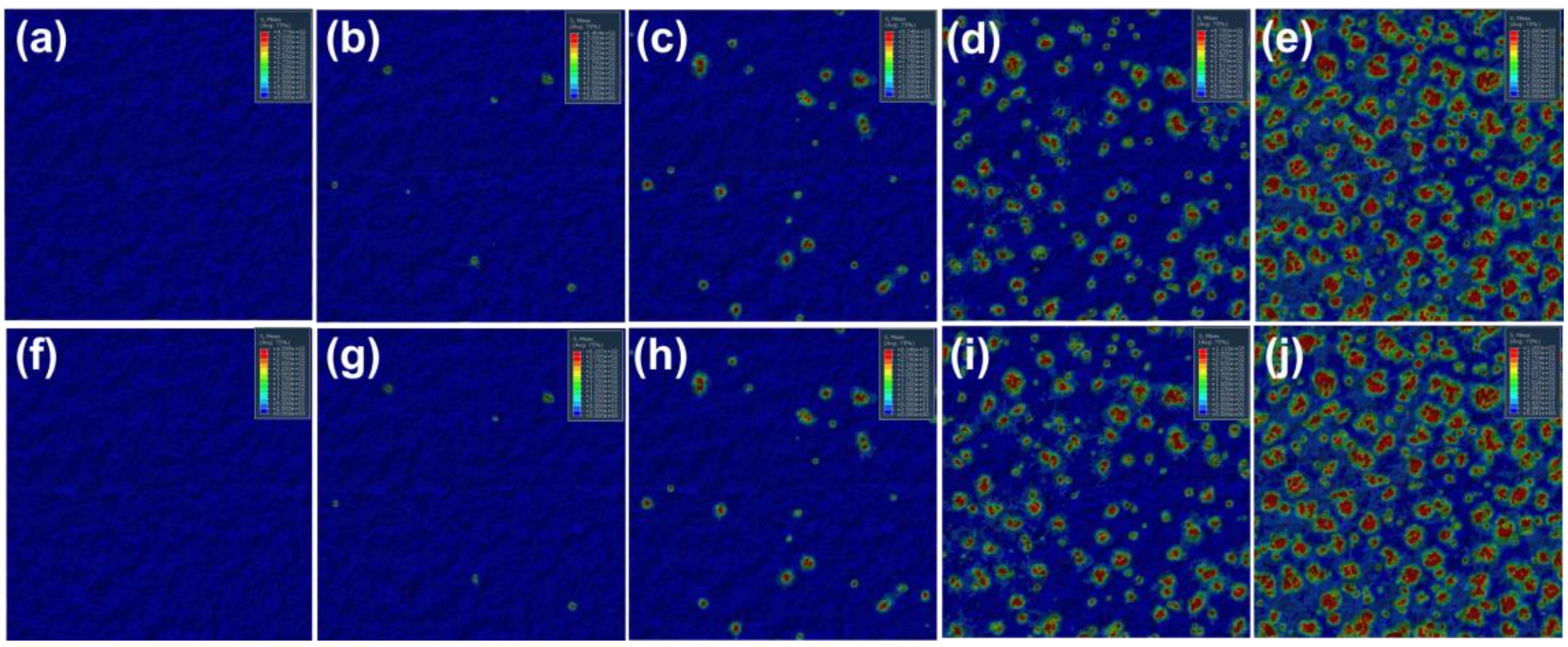

Figure S19 Contact stress distribution of the TENG after running-in with hexadecane under the contact depth of 100 nm (a), 200 (nm) (b), 300 nm (c), 400 nm (d), and 500 nm (e); Contact stress distribution after running-in with DMSO under different contact depth of 100 nm (a), 200 nm (b), 300 nm (c), 400 nm (d), and 500 nm (e).

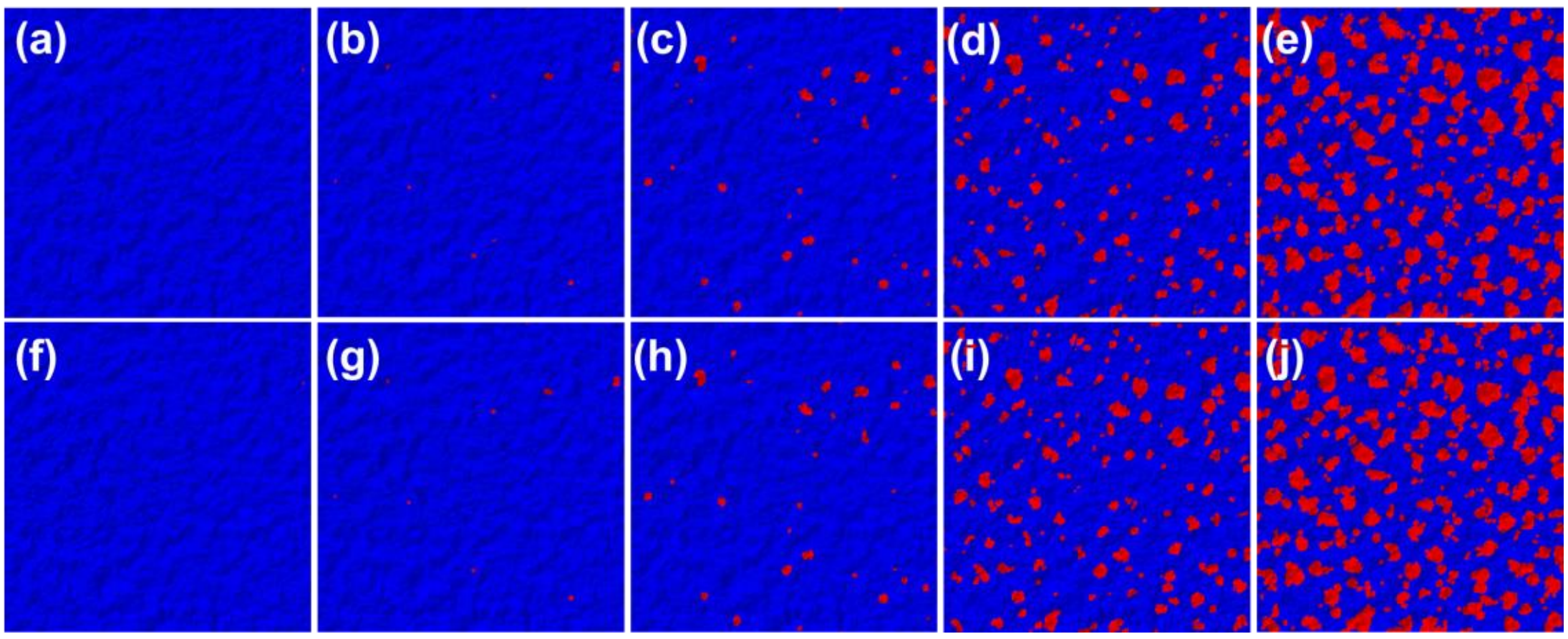

Figure S20 Real contact area (red spots showing the in-of-contact zones) after the running-in with hexadecane under the contact depth of 100 nm (a), 200 (nm) (b), 300 nm (c), 400 nm (d), and 500 nm (e); Real contact area after the running-in with DMSO under the contact depth of 100 nm (a), 200 nm (b), 300 nm (c), 400 nm (d), and 500 nm (e).

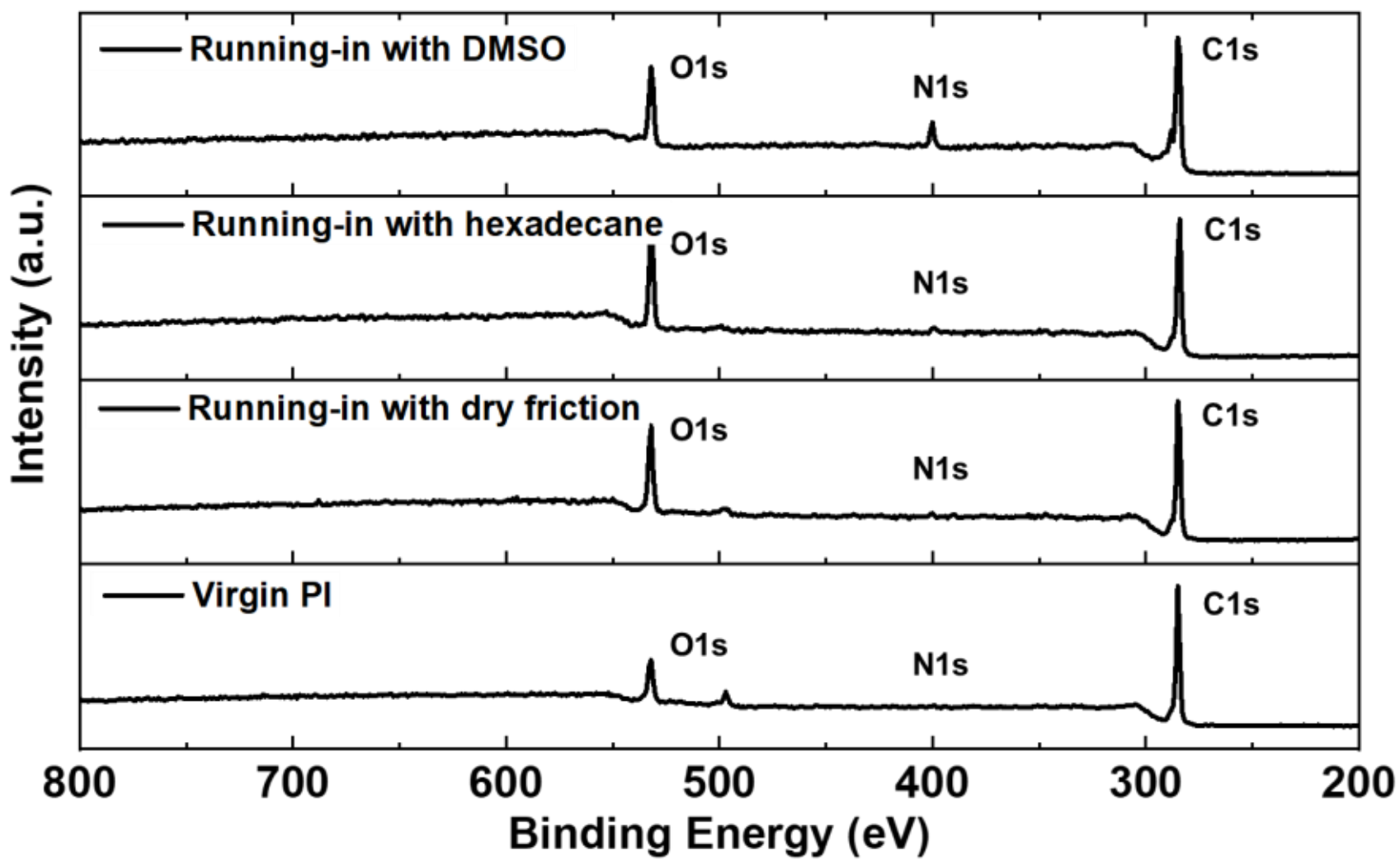

Figure S21 XPS survey spectra of the PI surfaces

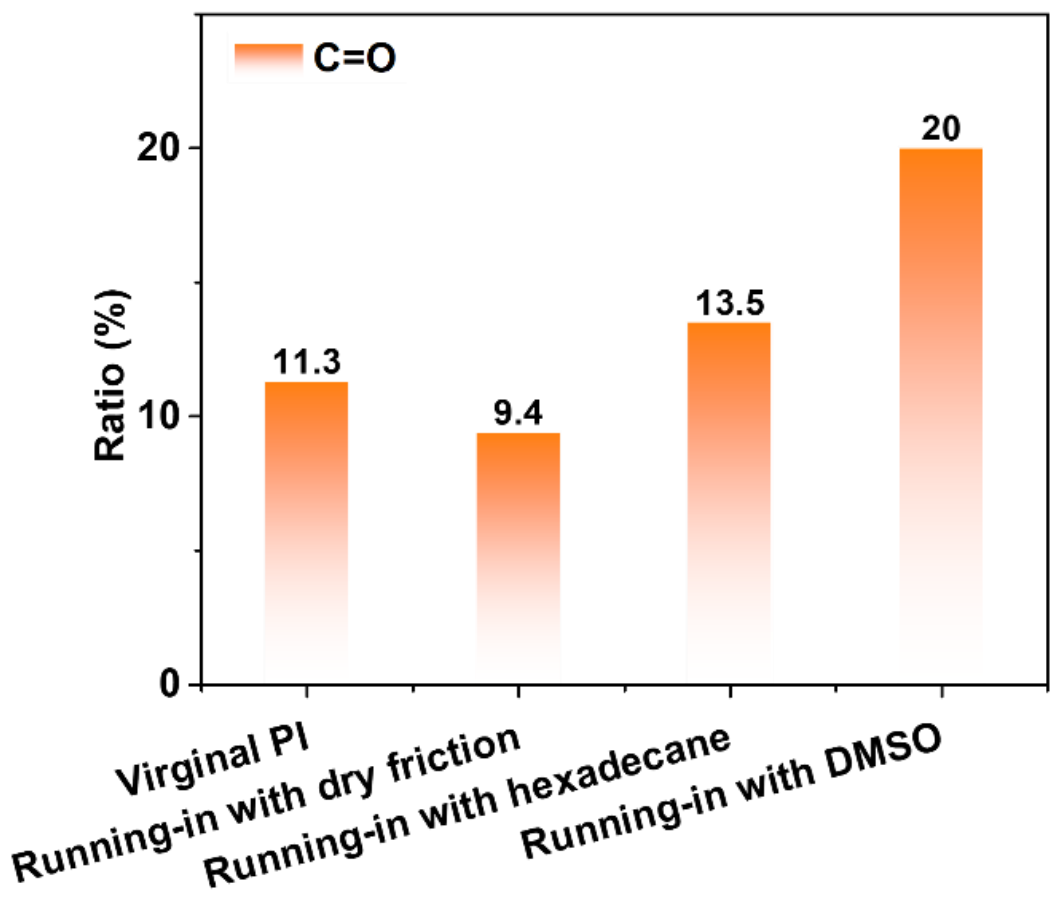

Figure S22 The chemical group ratio of C=O from the PI surface after the running-in period

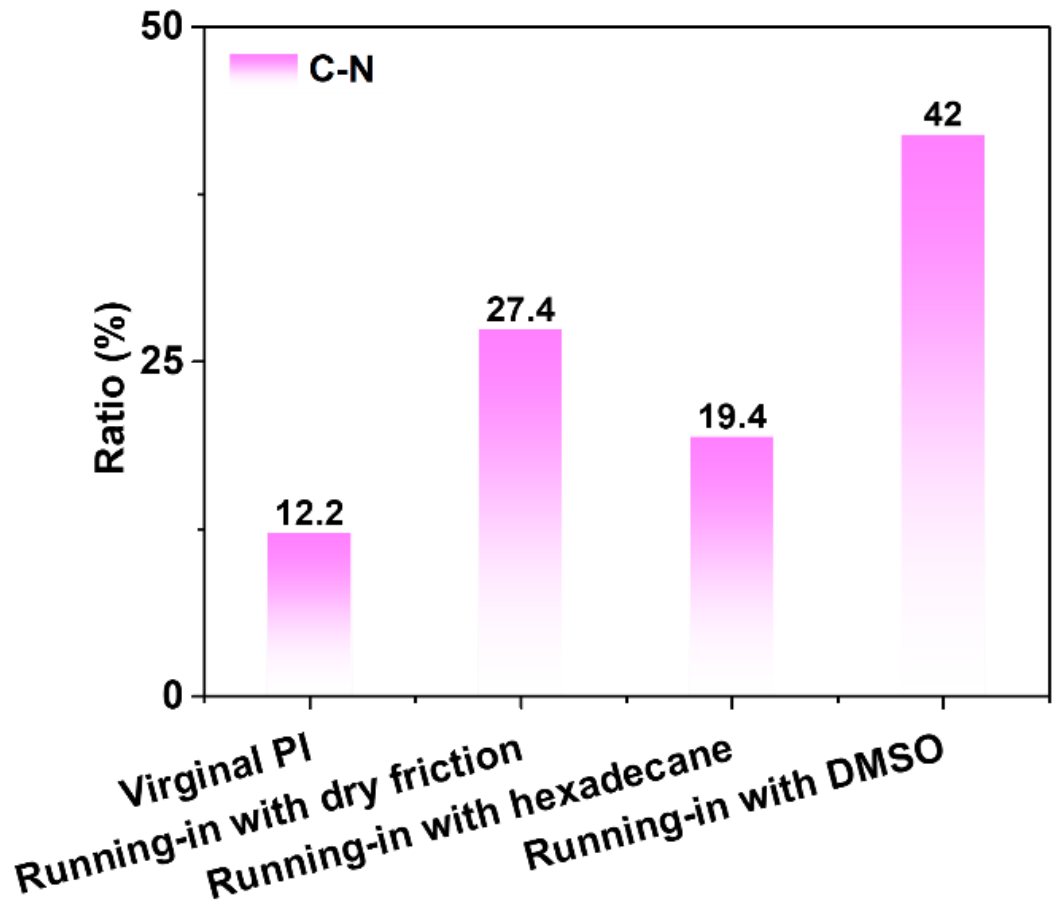

Figure S23 The chemical group ratio of C-N from the PI surface after the running-in period

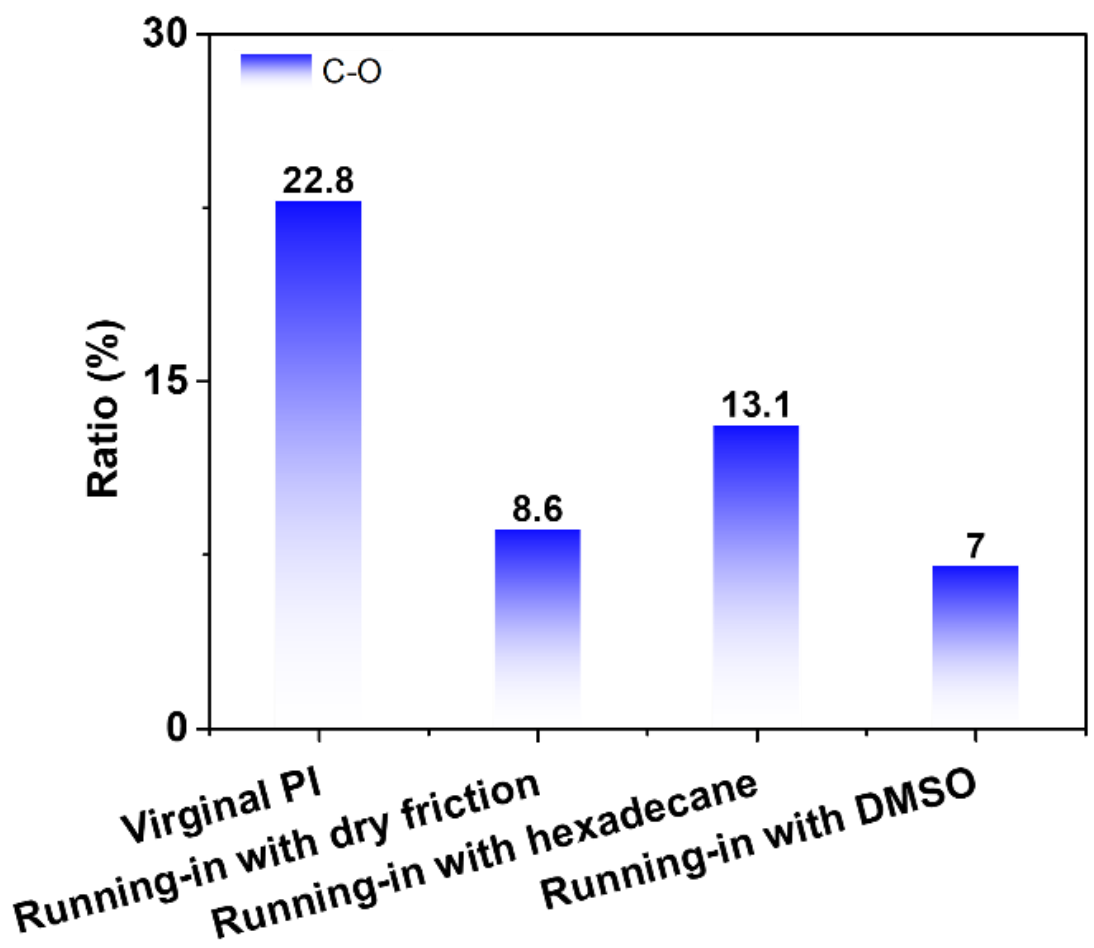

Figure S24 The chemical group ratio of C-O from the PI surface after the running-in period

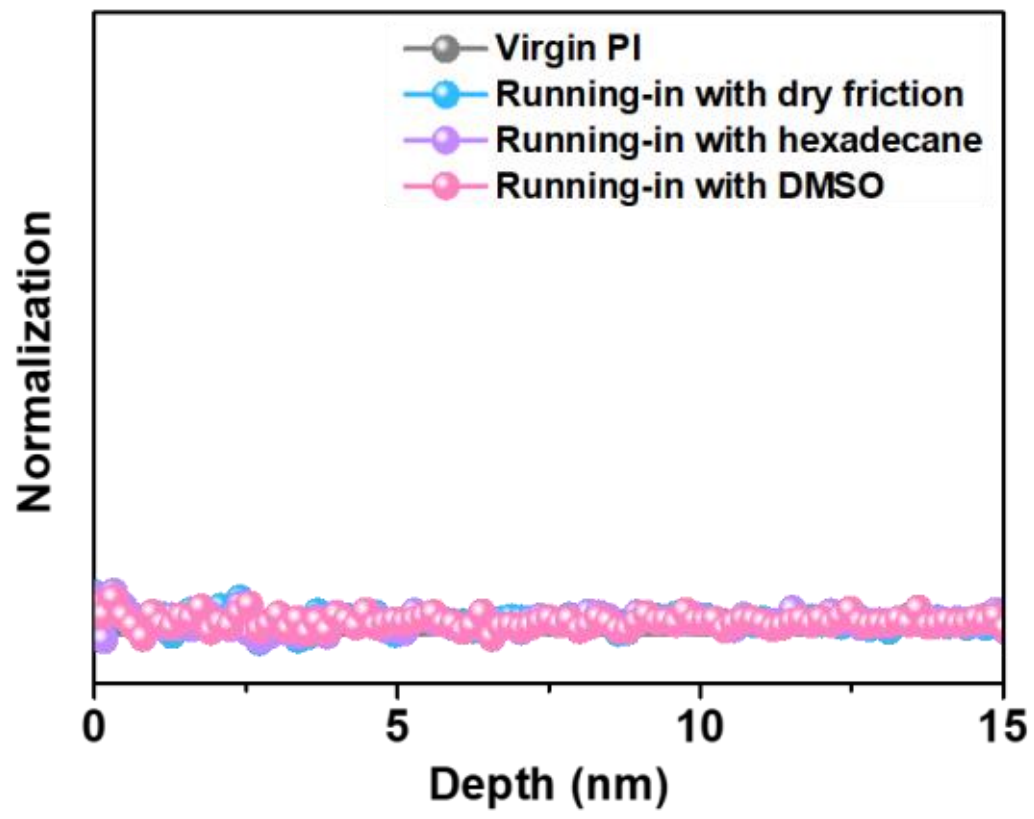

Figure S25 The content changes of the CO^- fragment along the depth direction.

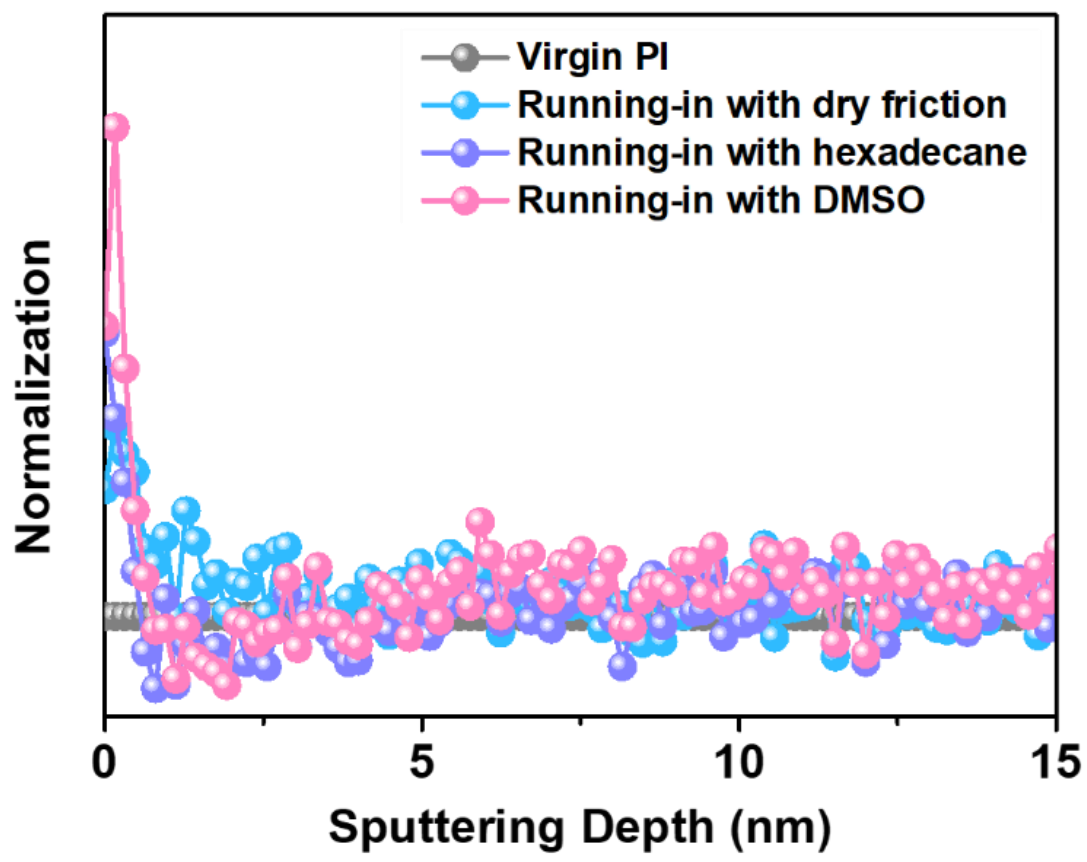

Figure S26 The content changes of the NH^- fragment along the depth direction.

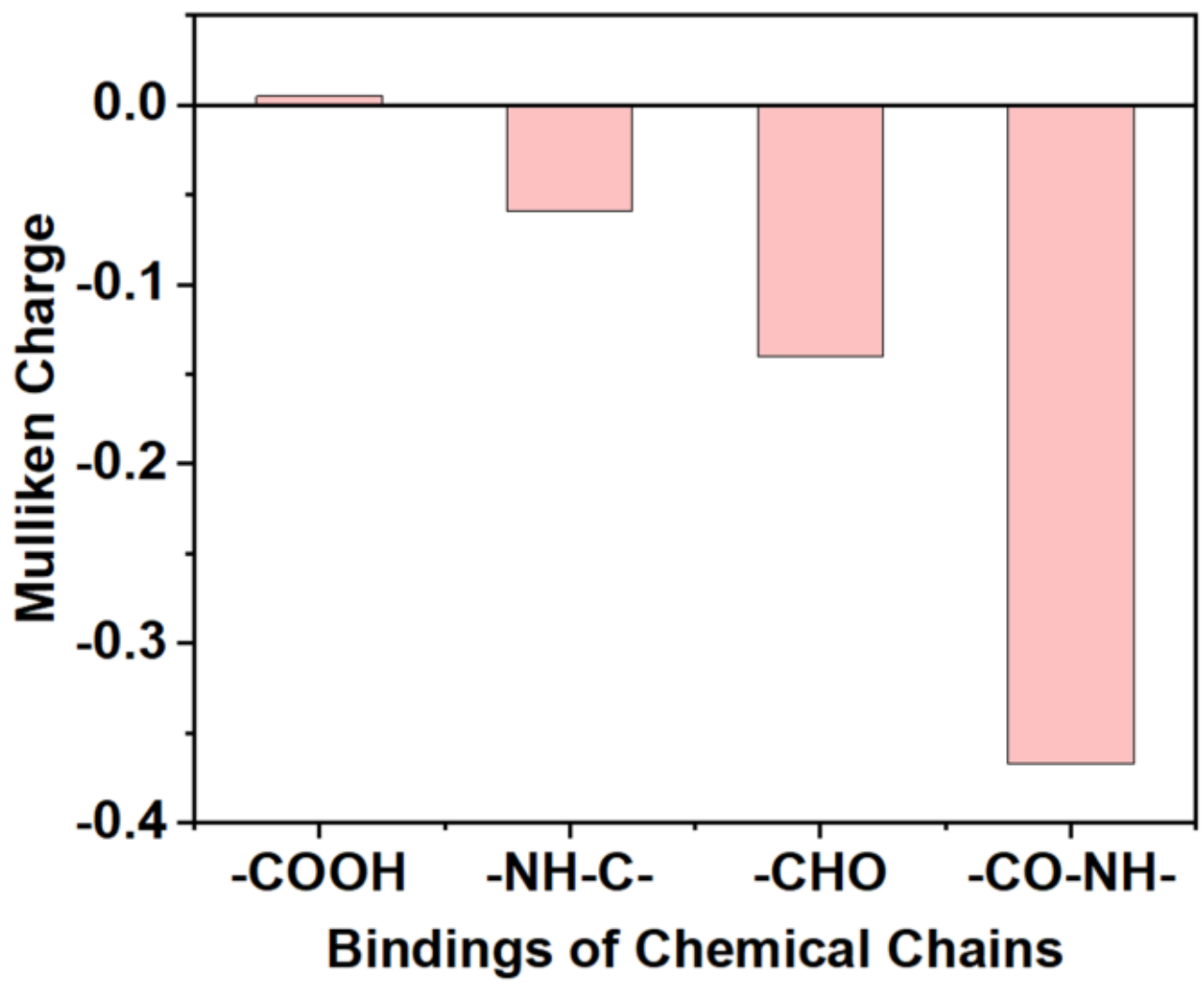

Figure S27 Mulliken charge of the chain scissions in PI induced by the running-in process.

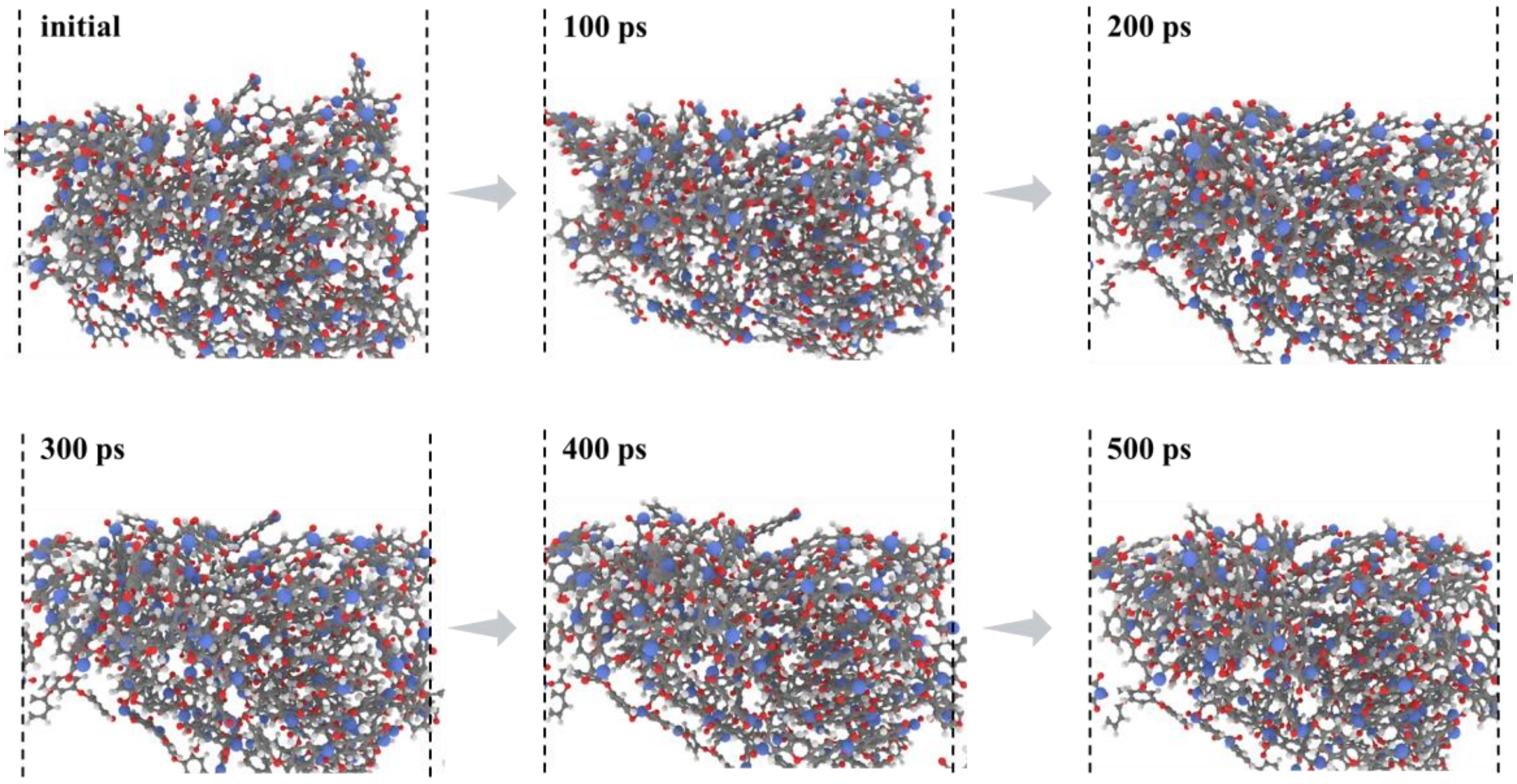

Figure S28 The structural optimization for the PI bulk phase was performed by classical MD for 500 ps to minimize system energy and ensure structural stability.

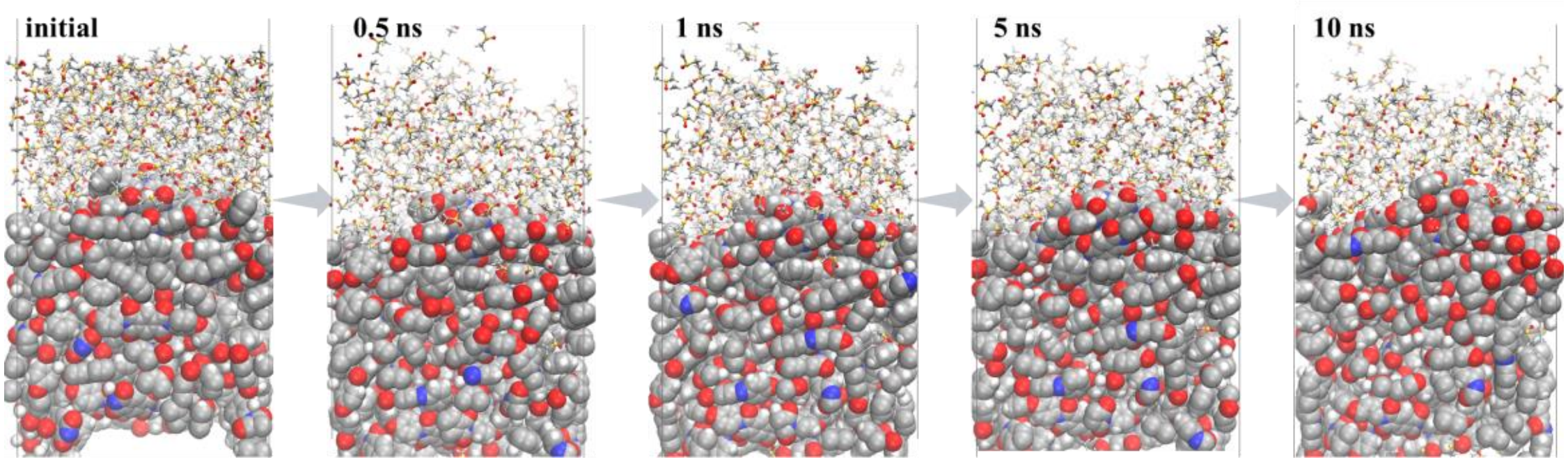

Figure S29 Time-dependent MD analysis of the extraction effect of DMSO molecules virgin PI for comparison.

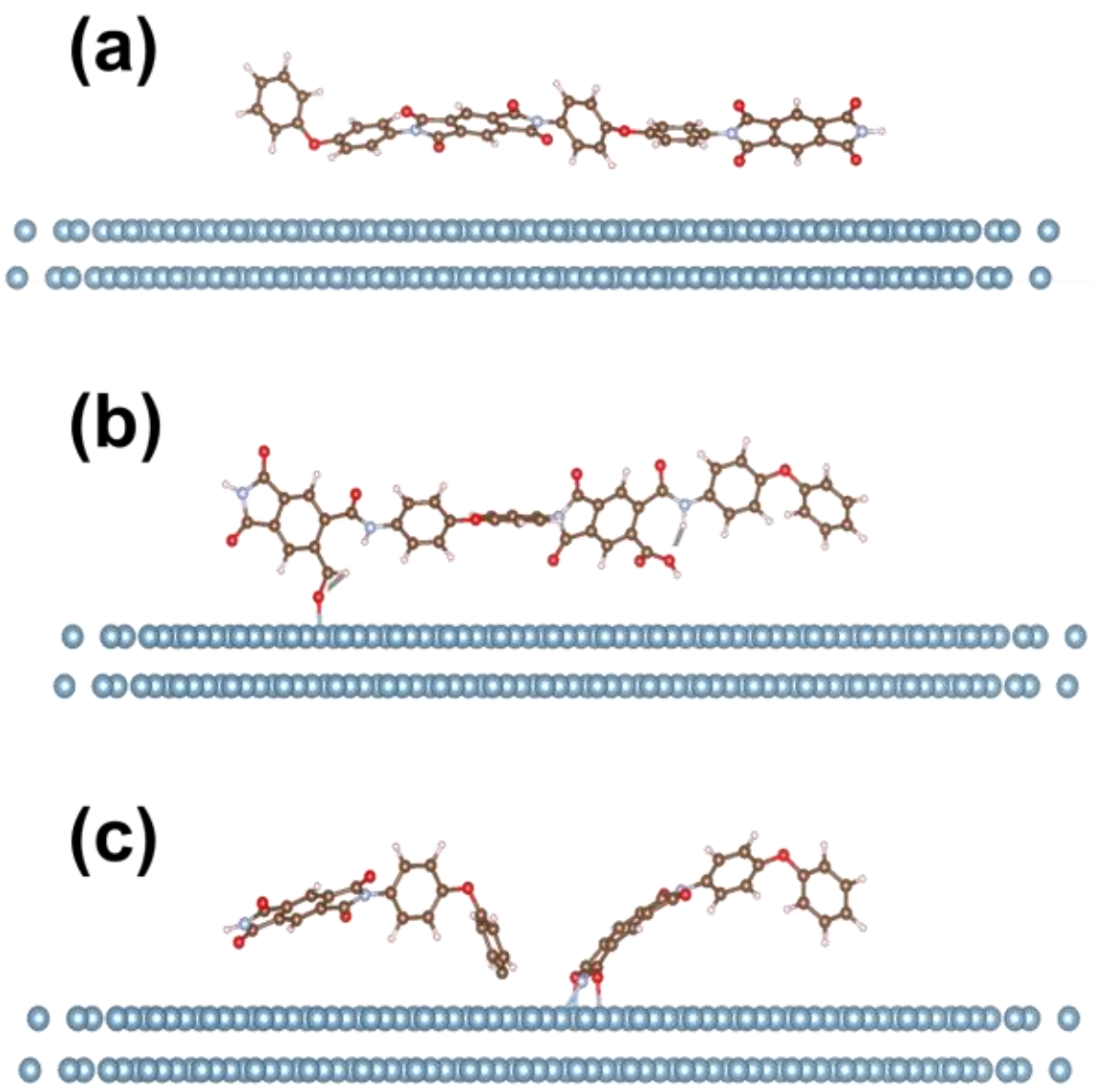

Figure S30 Schematic diagram of contact models of AI-PI with an energy minimum state. The contact model of the Al surface and virgin PI (a). The contact model of the Al surface and the running-in PI with the molecular chain scission at the C-N bond around carbonyl group (b). The contact model of the Al surface and the running-in PI with the molecular chain scission at the C-N bond around benzene group (c).

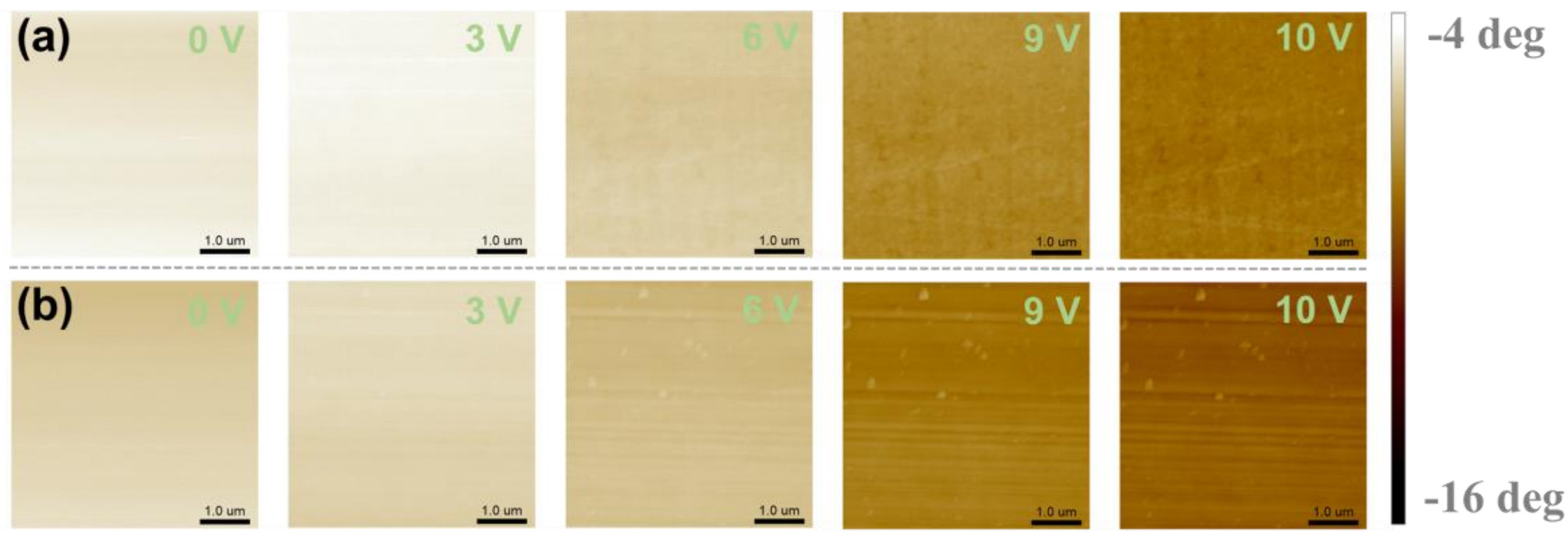

Figure S31 Electrical phase images via EFM for PIs after running-in for 0 cycle (a) and 100 cycles (b).

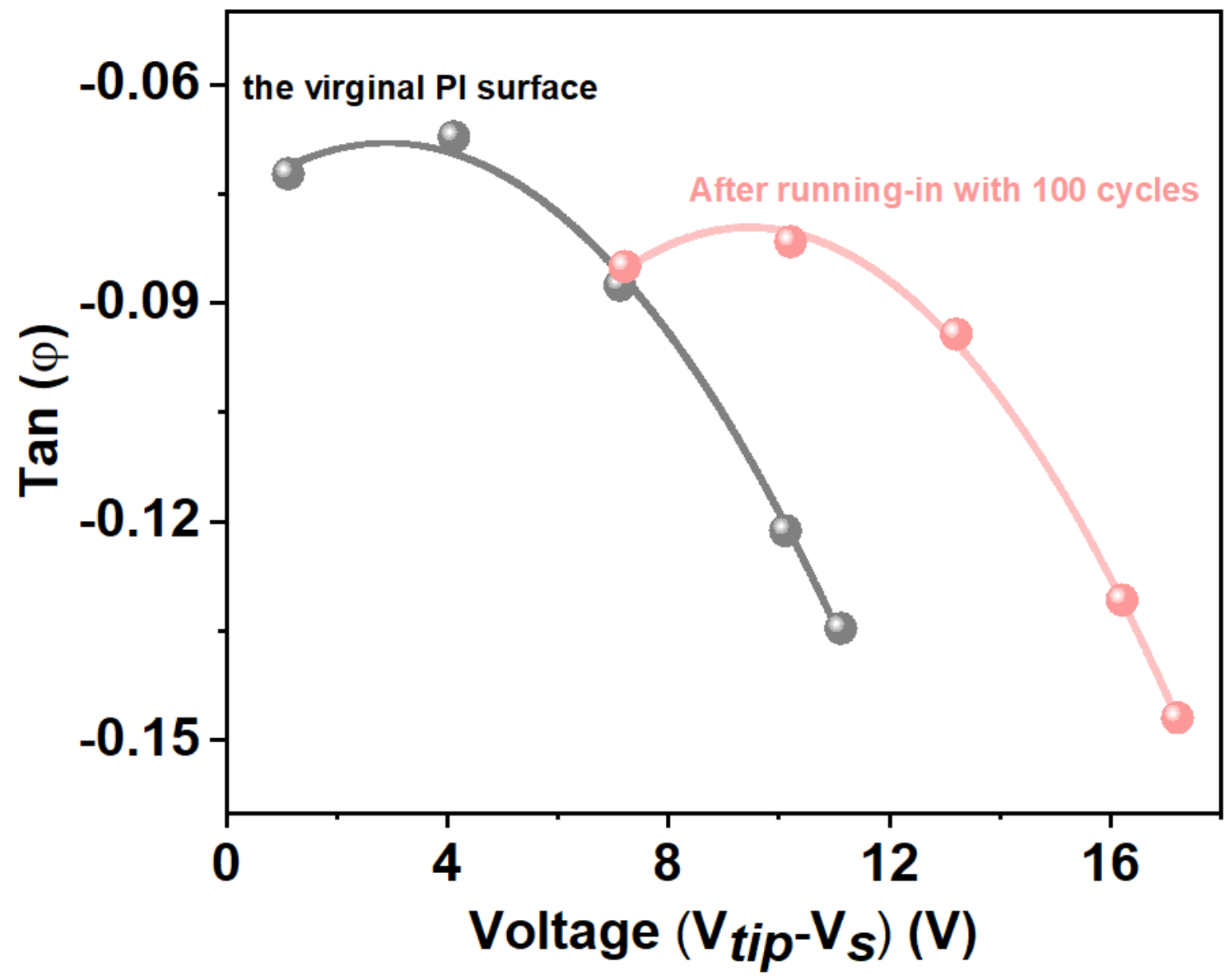

Figure S32 Characteristic curve of the tan φ of the PI as a function of the voltage between the tip and the sample surface ($Vtip$-Vs).

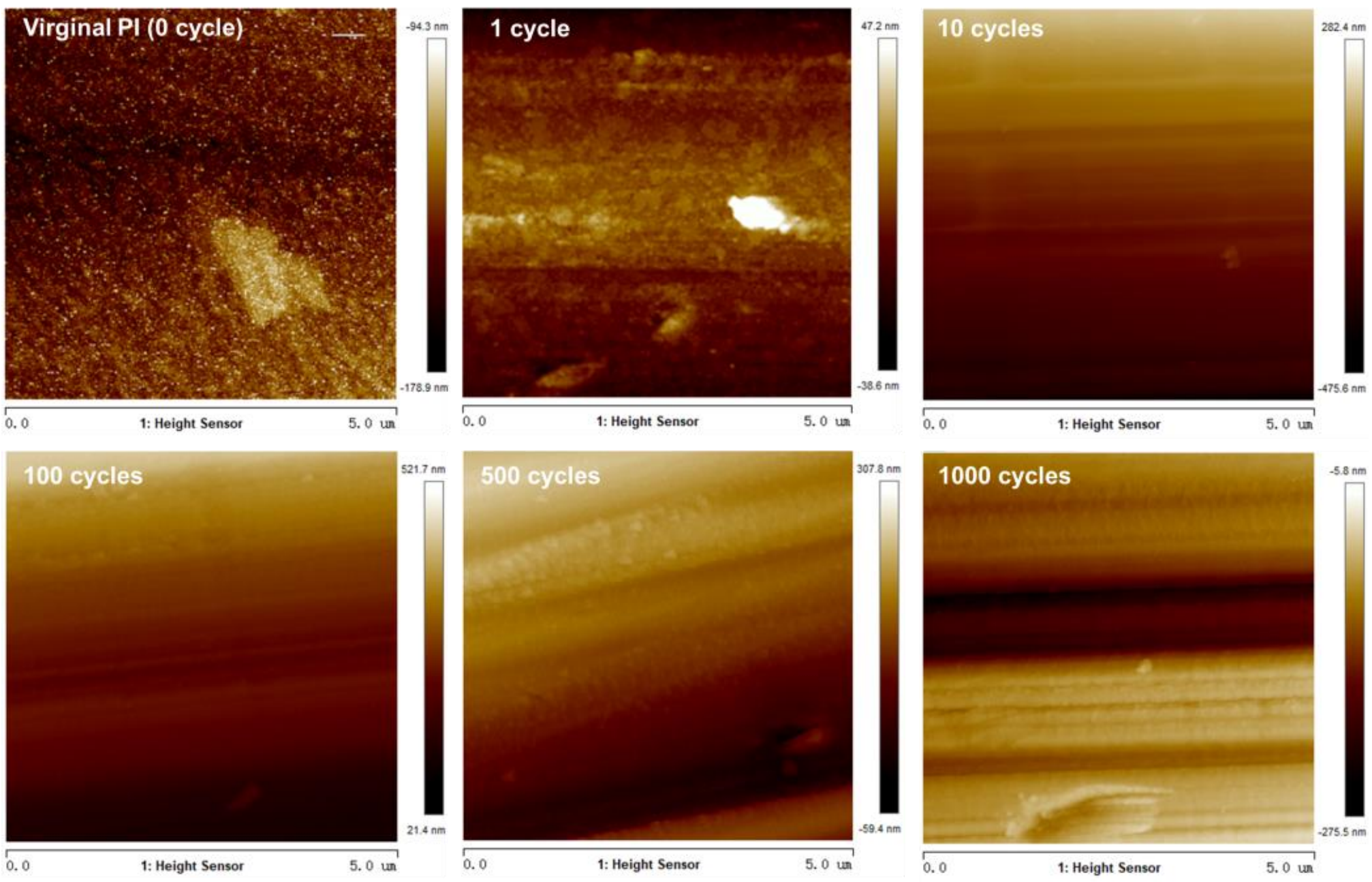

Figure S33 Surface morphology of the PI after running-in with DMSO for different cycles through AFM analysis.

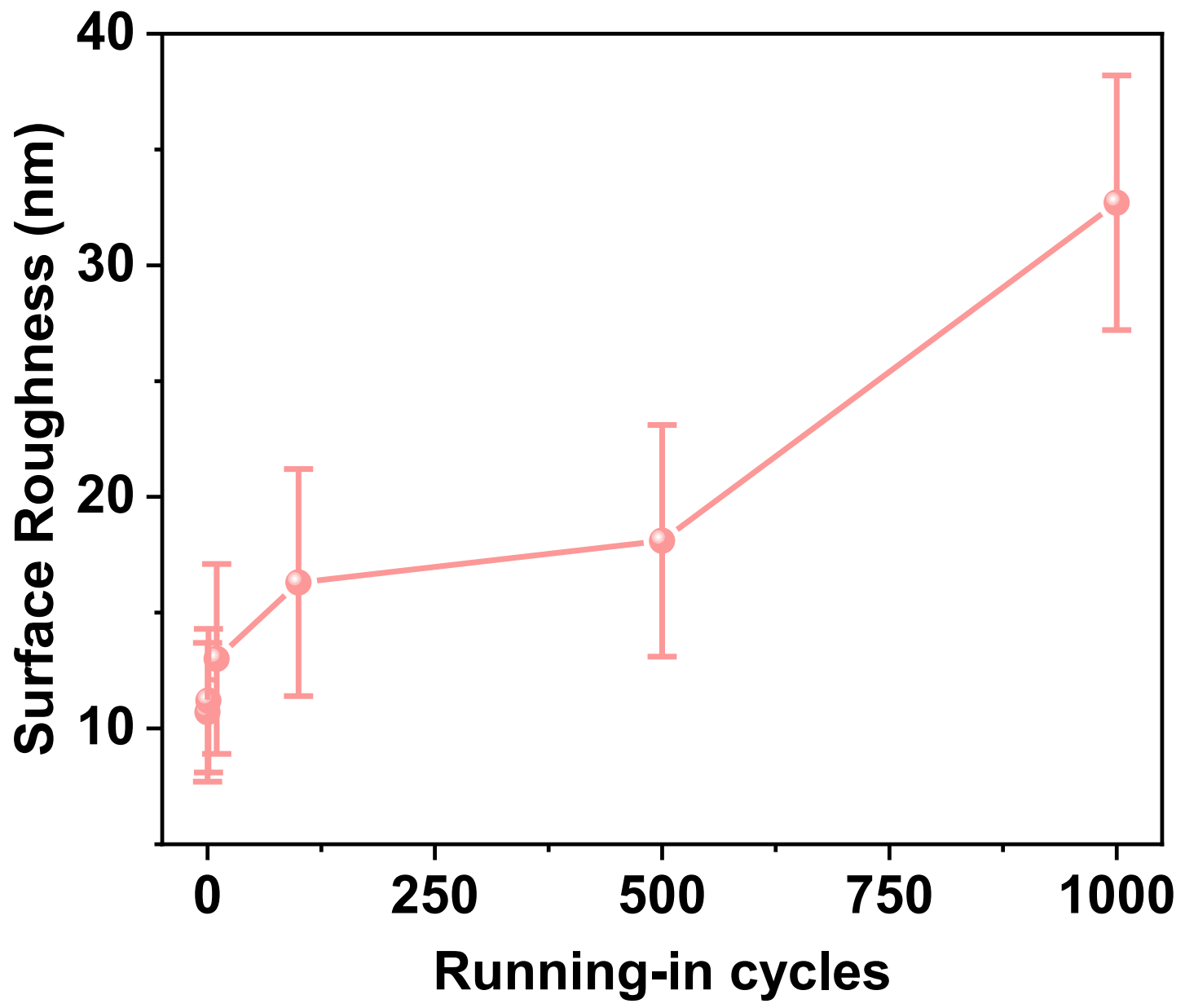

Figure S34 Surface roughness of the PI after running-in with DMSO at different cycles through AFM analysis.

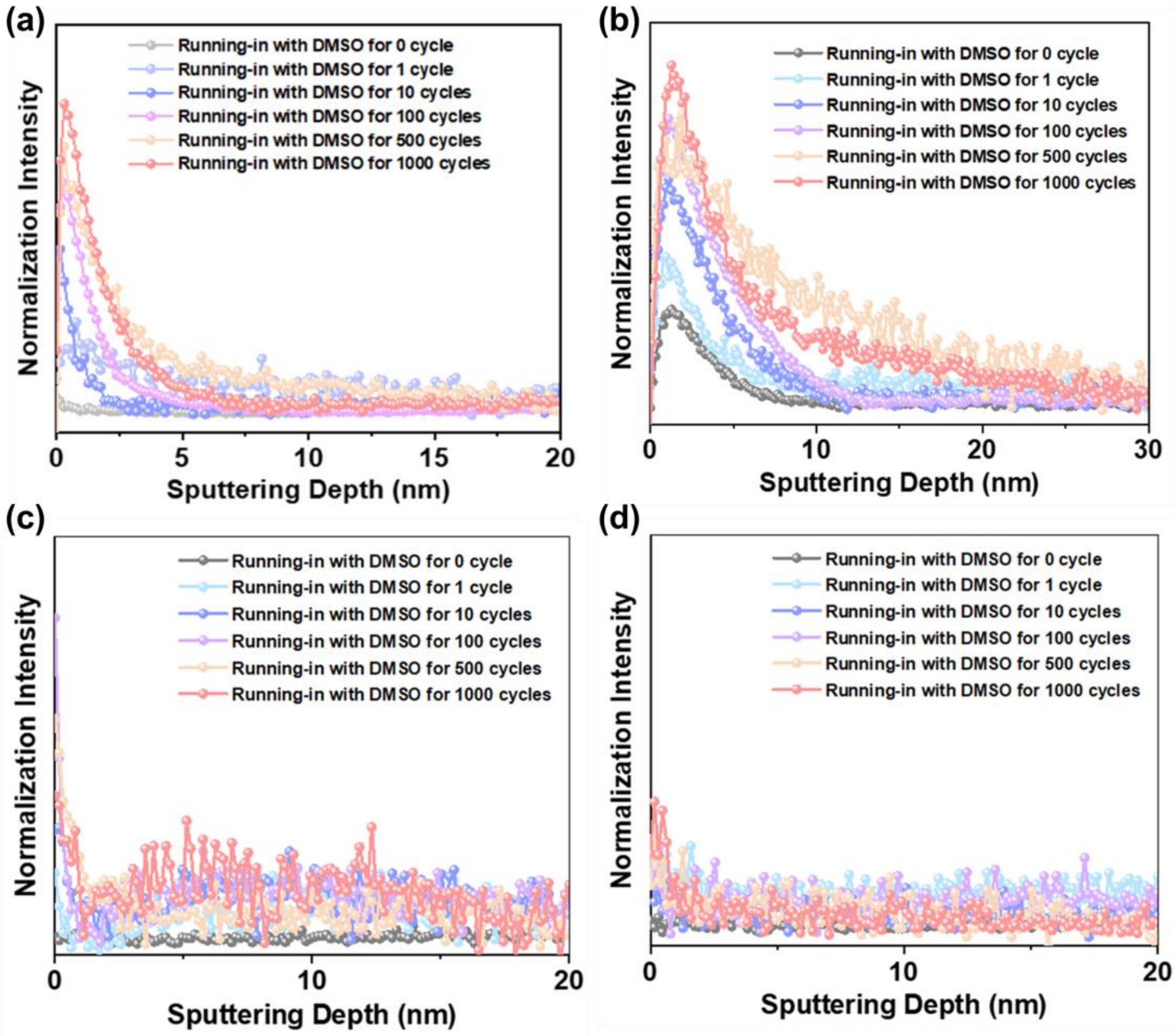

Figure S35 Content changes of CNO^{-}(a), CN^{-} (b), NH^{-} (c) and CO^{-} (d) fragments respectively along the depth direction.

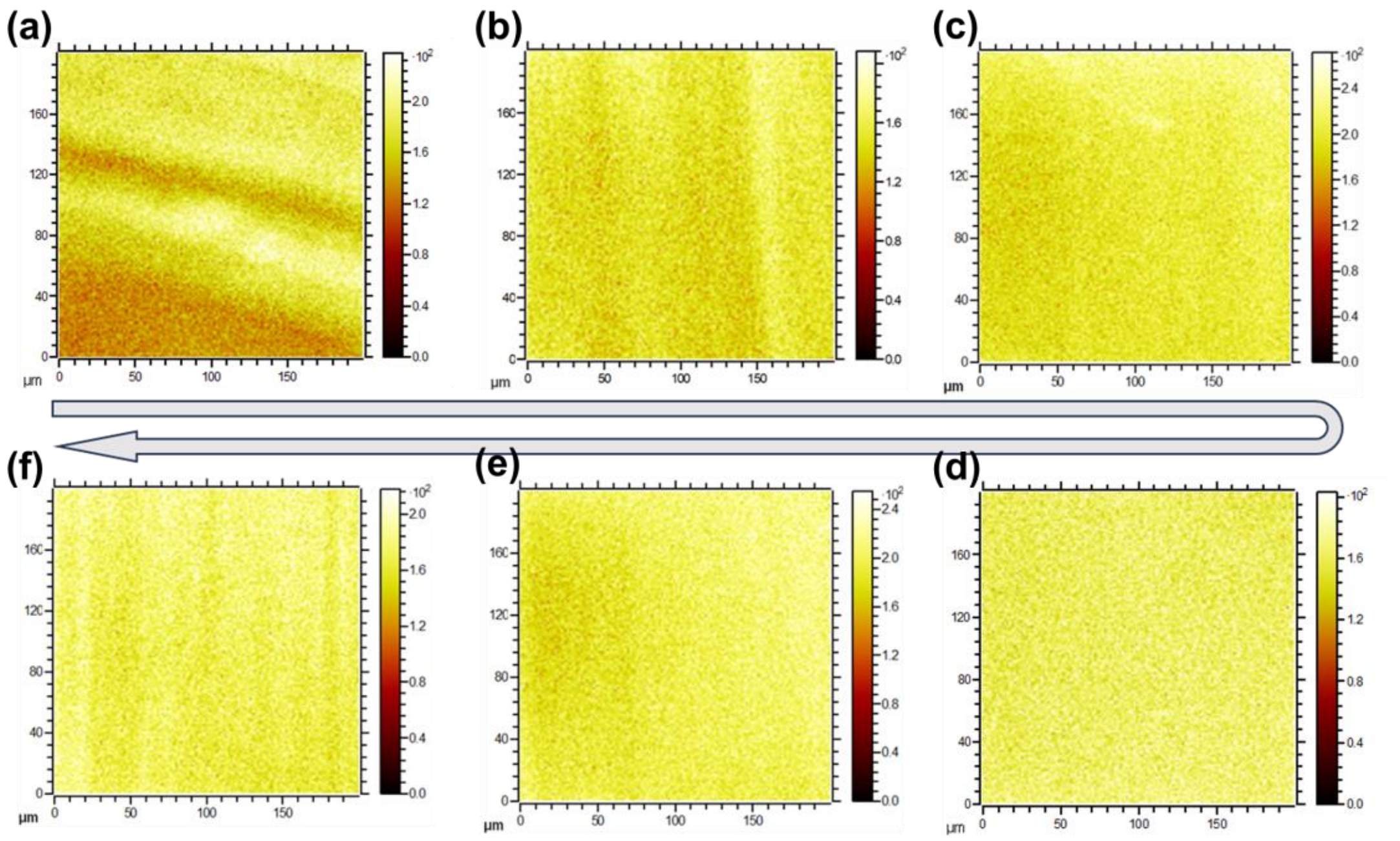

Figure S36 TOF-SIMS images showing the distributions of the chemical fragment of CNO- after running-in with DMSO for different cycles, 0 cycle (a), 1 cycle (b), 10 cycles (c), 100 cycles (d), 500 cycles (e), and 1000 cycles (f).

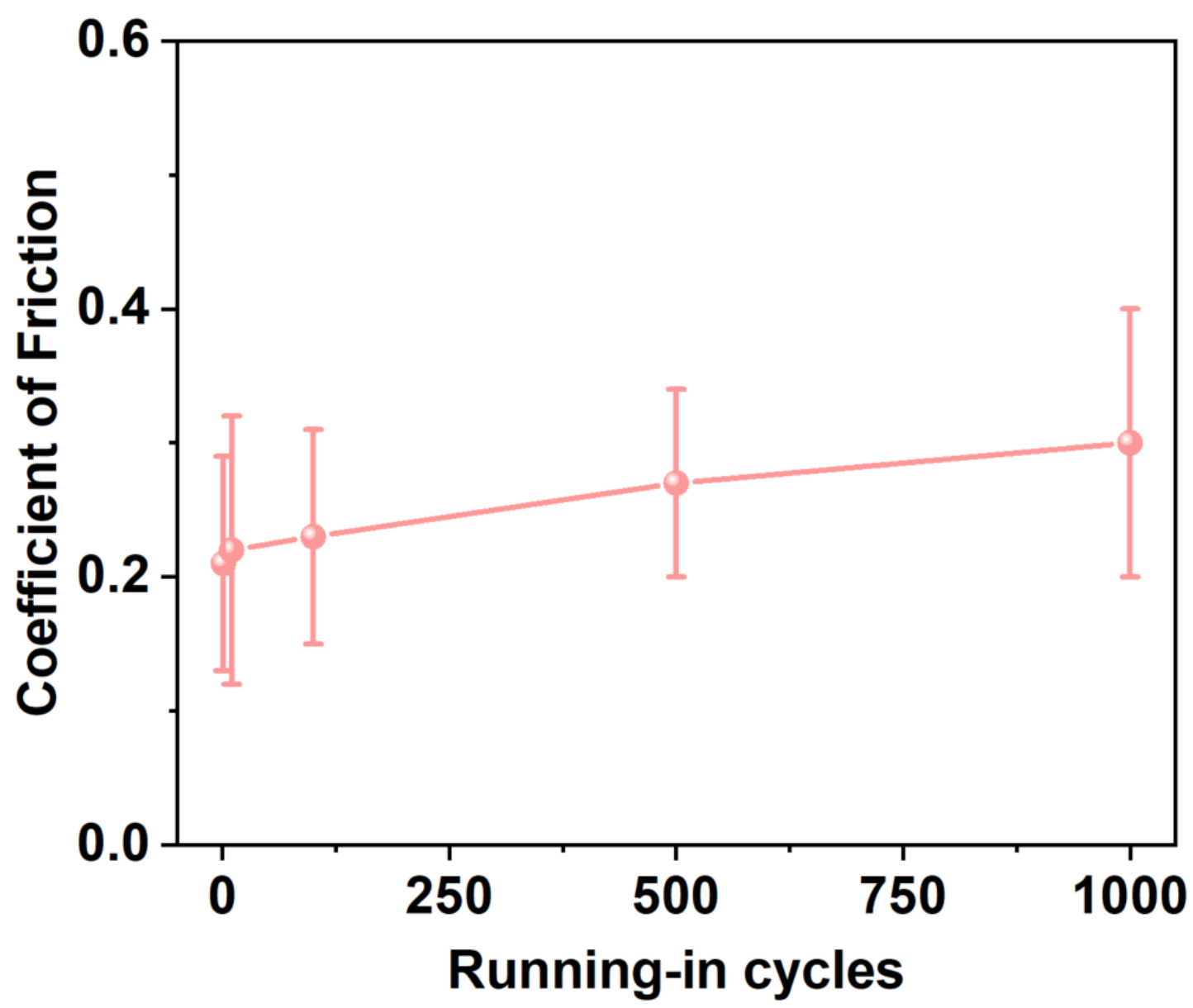

Figure S37 Coefficient of friction of the PI-based TENG as a function of DMSO-assisted running-in cycles.

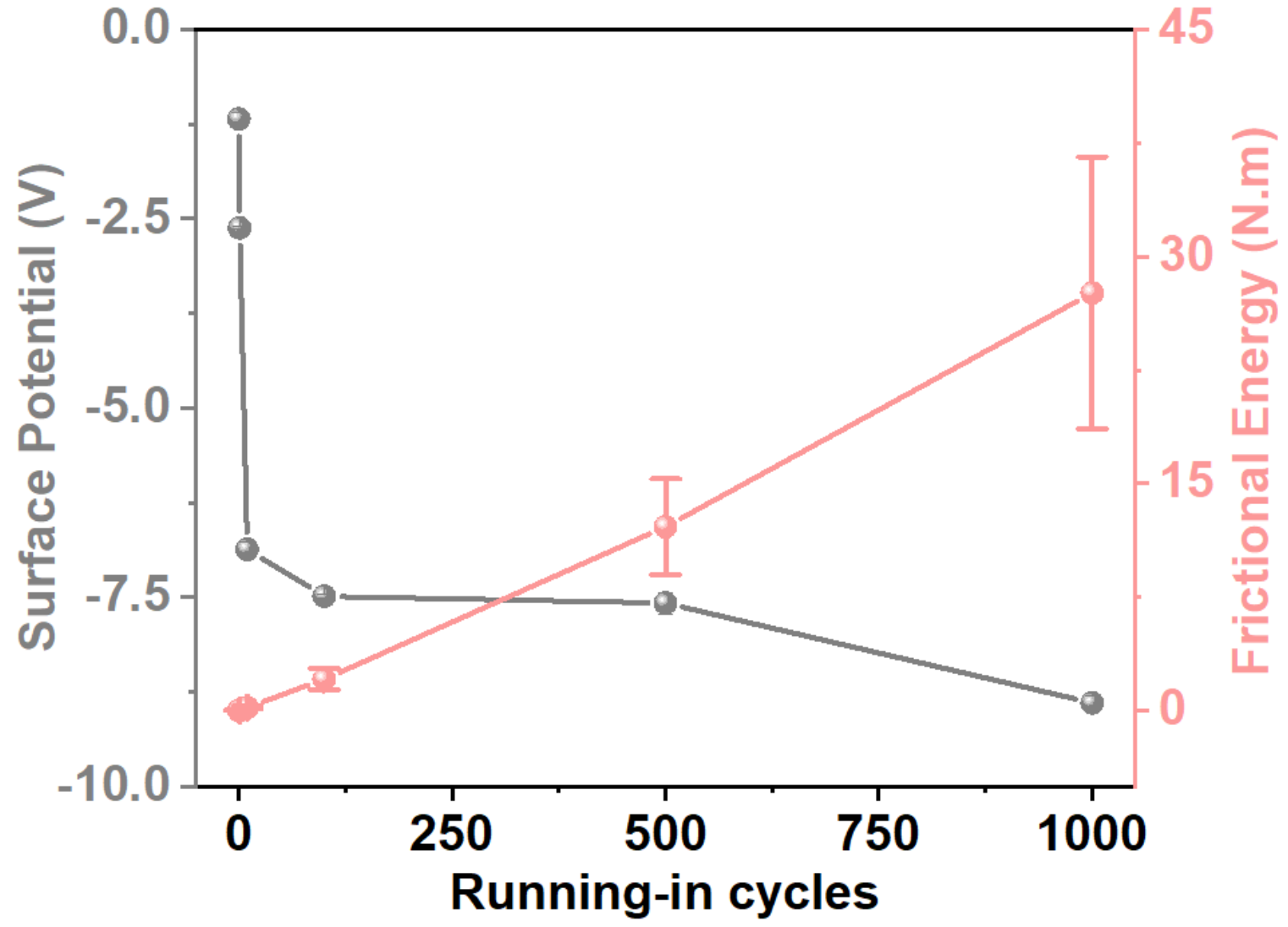

Figure S38 Surface potential and frictional energy consumption based on the work done by friction forces as a function of running-in cycles.

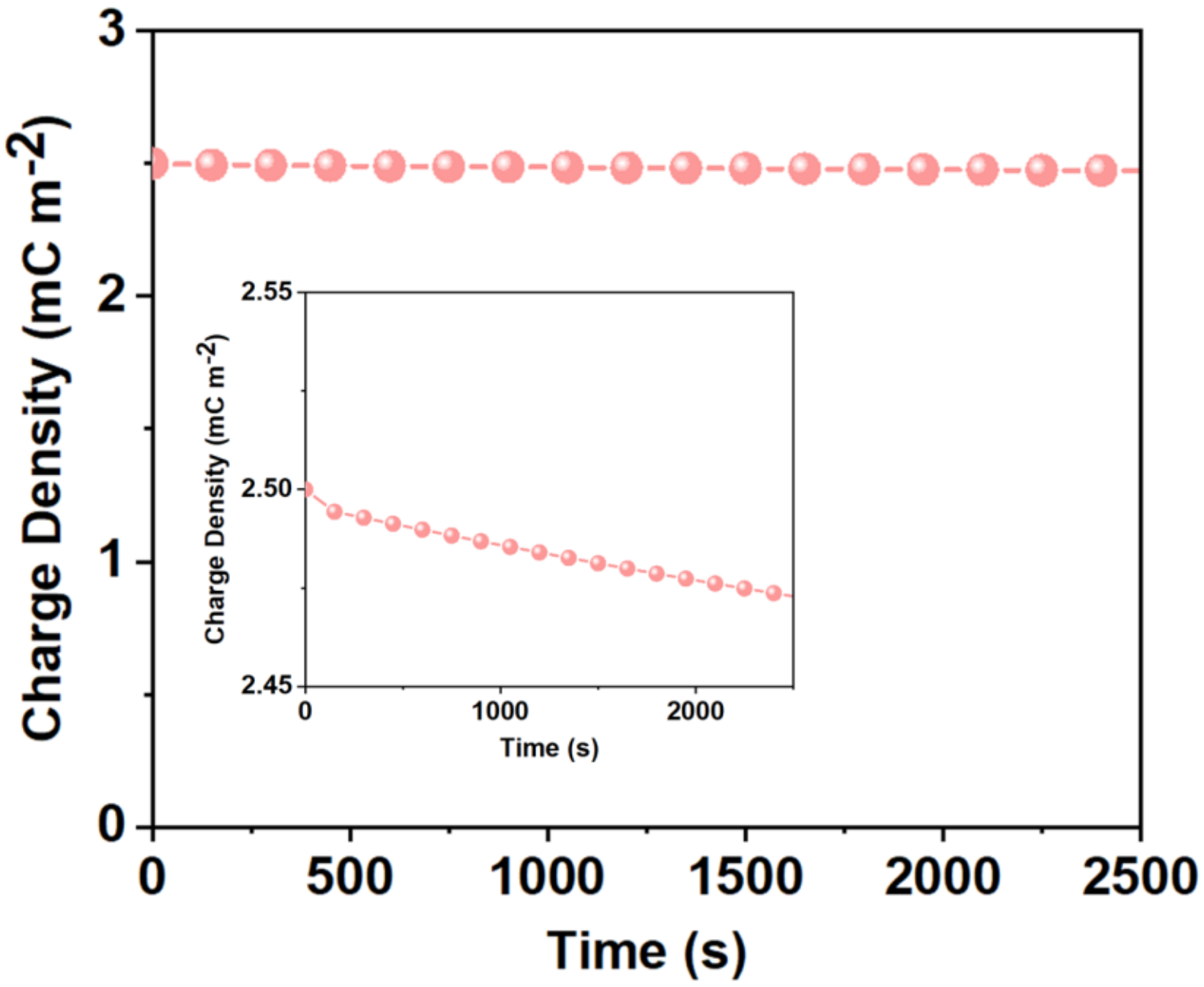

Figure S39 Charge decay of the TENG with the lubrication of hexadecane after running-in with DMSO.

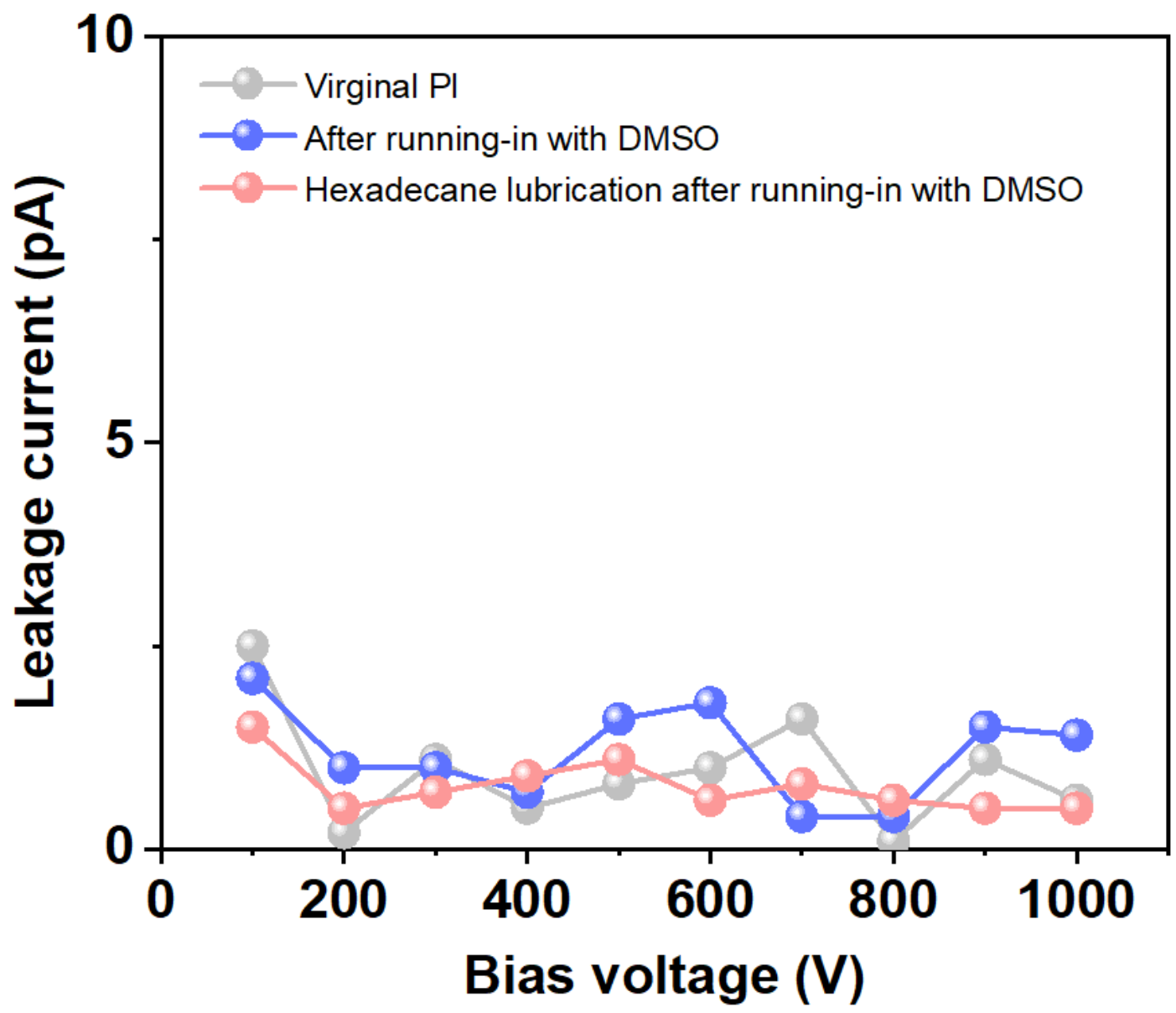

Figure S40 Leakage current of the PIs under different bias voltages

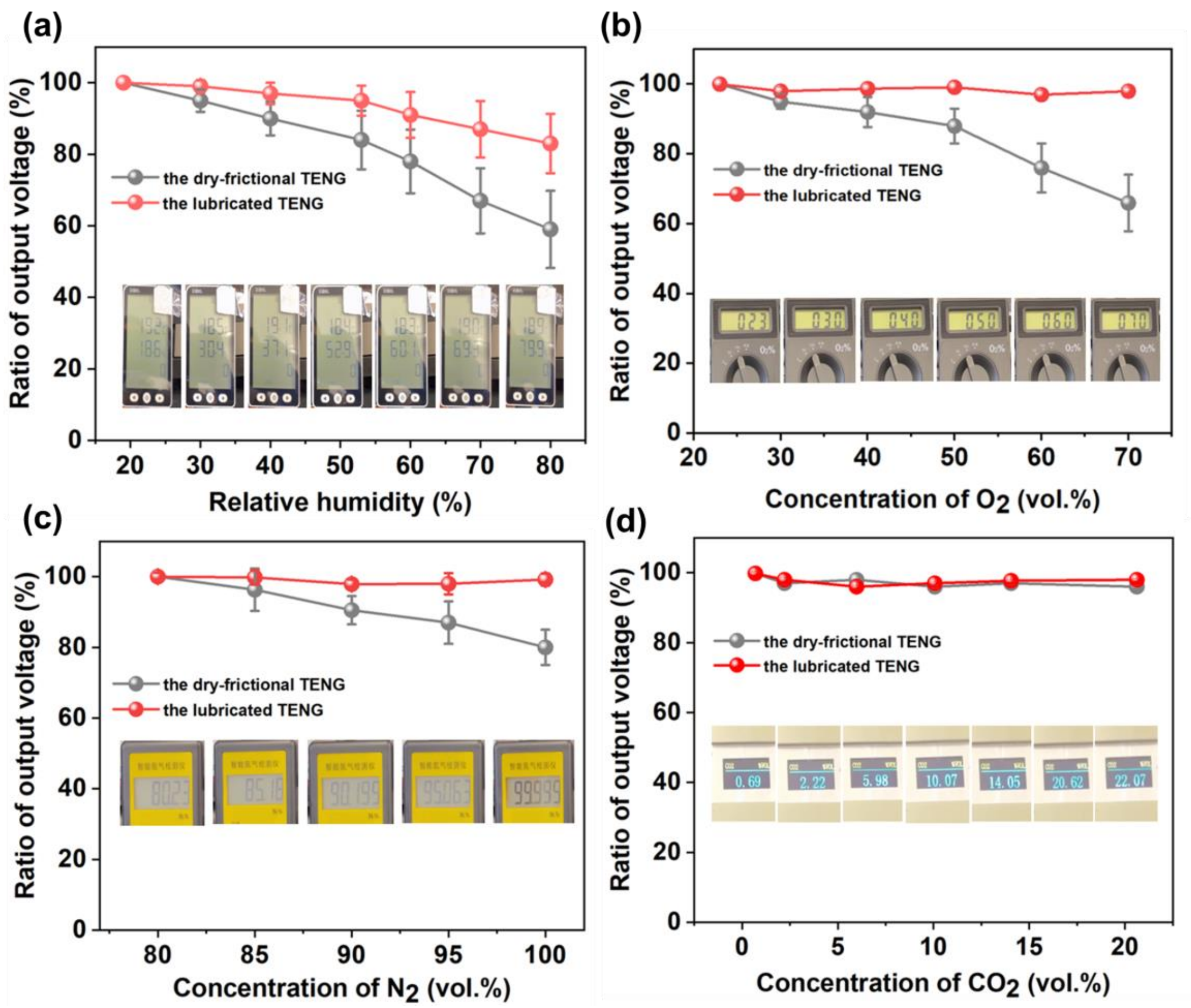

Figure S41 The influence of humidity and gas in air on the electric performance of the TENG with the lubrication of hexadecane after running-in with DMSO in contrast to the TENG under dry friction, relative humidity (a), O_2 (b), N_2 (c), and CO_2 (d).

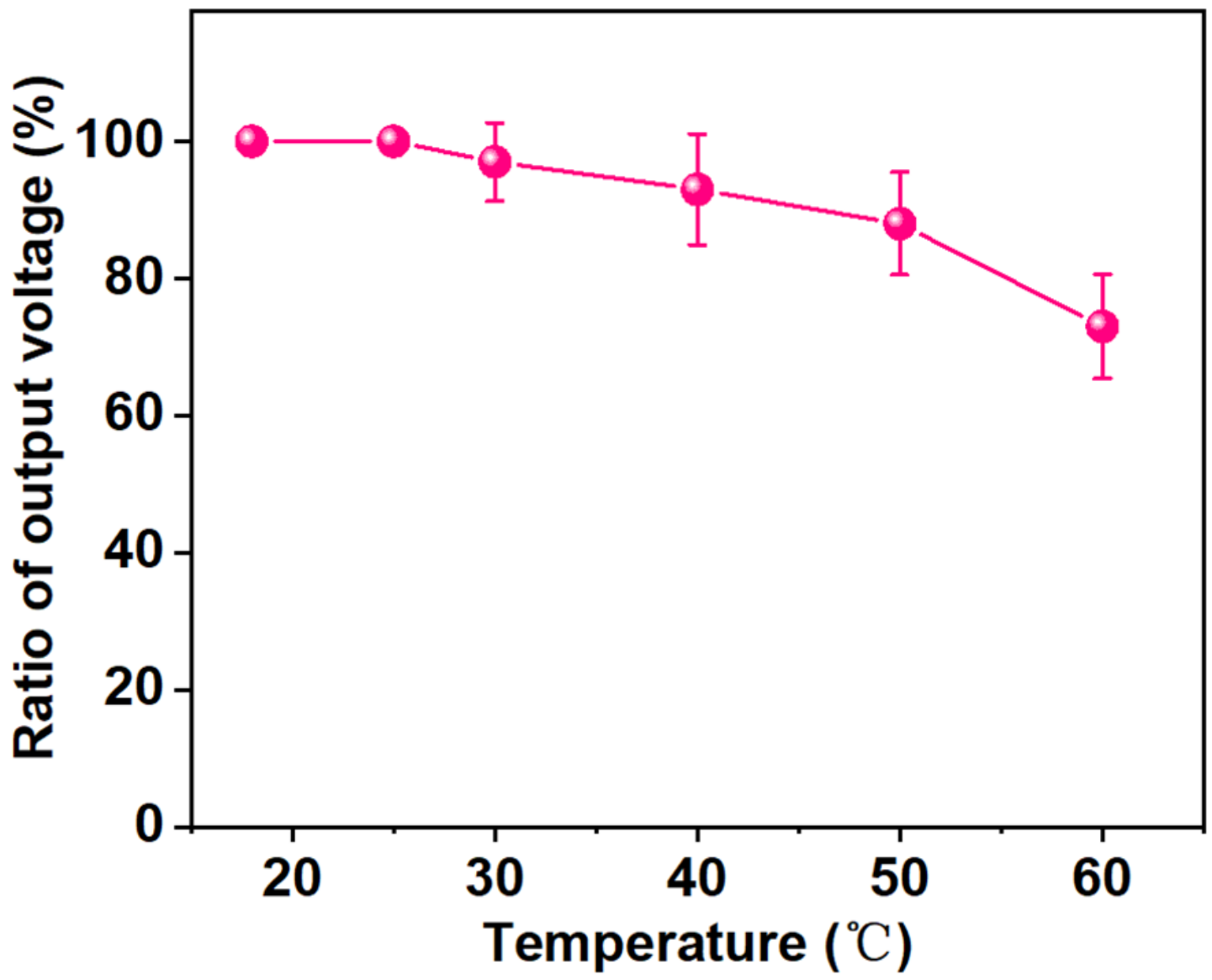

Figure S42 The influence of ambient temperature on the electric performance of the TENG with the lubrication of hexadecane after running-in with DMSO at the atmosphere (relative humidity ~20%RH).

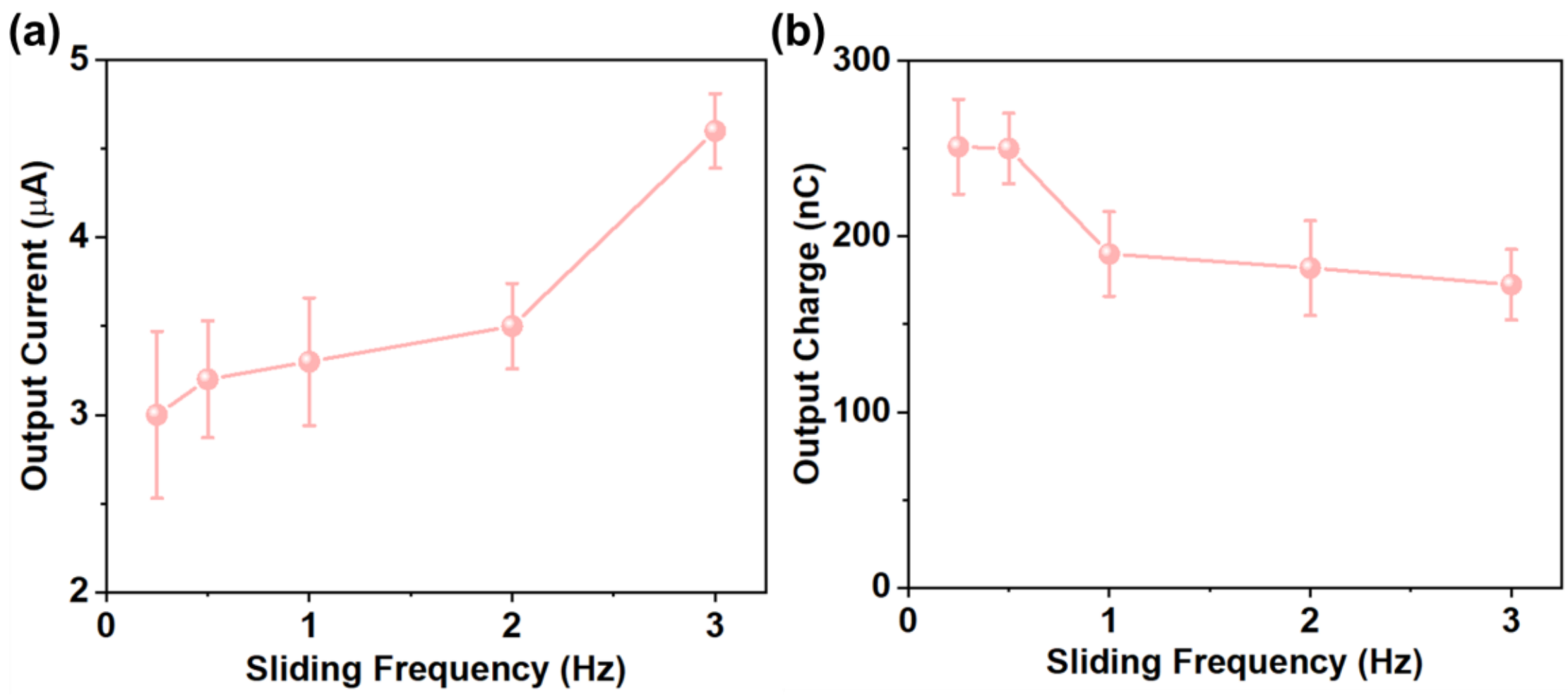

Figure S43 Output current (a) and charge (b) of the TENG lubricated by hexadecane after running-in with DMSO as a function of sliding frequency.

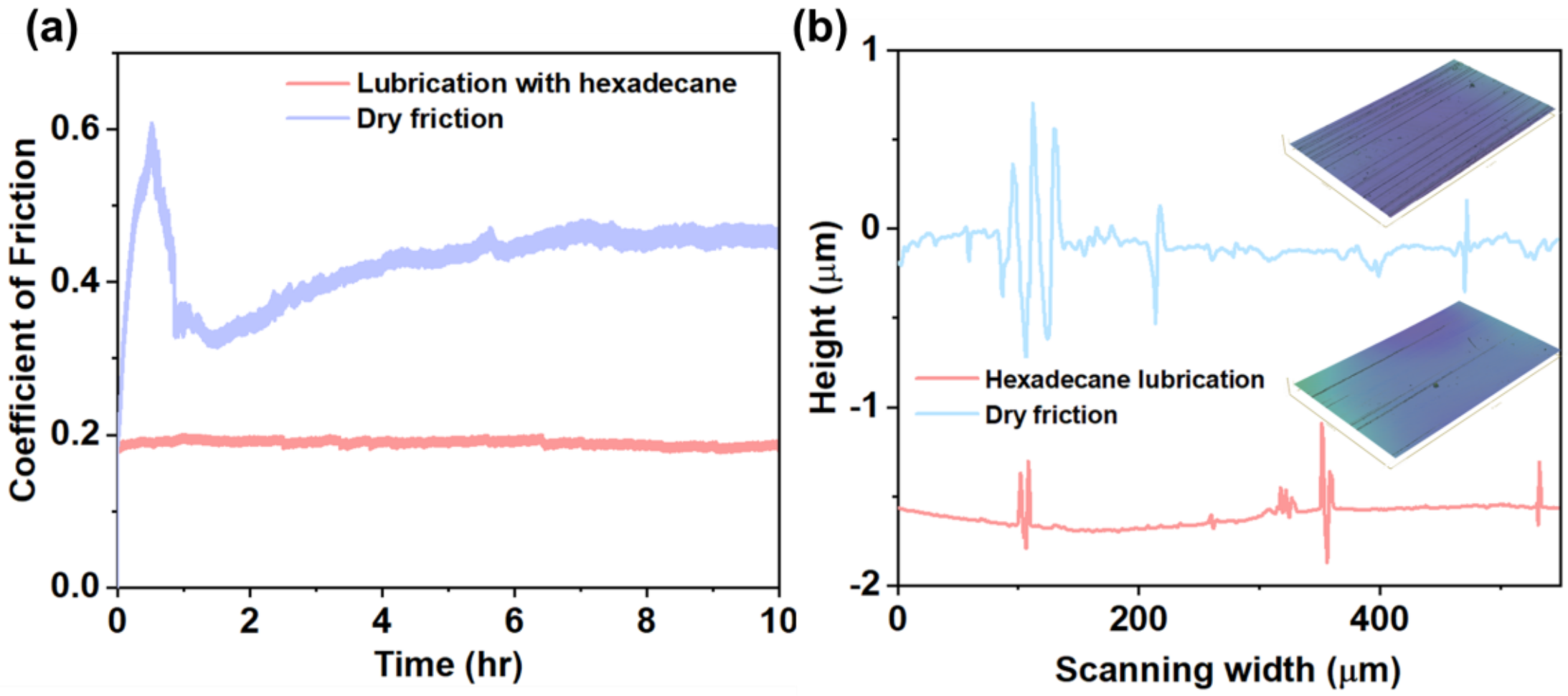

Figure S44 Coefficient of friction (a) and wear depth (b) of the hexadecane-lubricated TENG after running-in with DMSO compared with the dry-friction TENG in the long-term test, the inset in Fig. S44b showing the 3D morphologies after the long-term test.

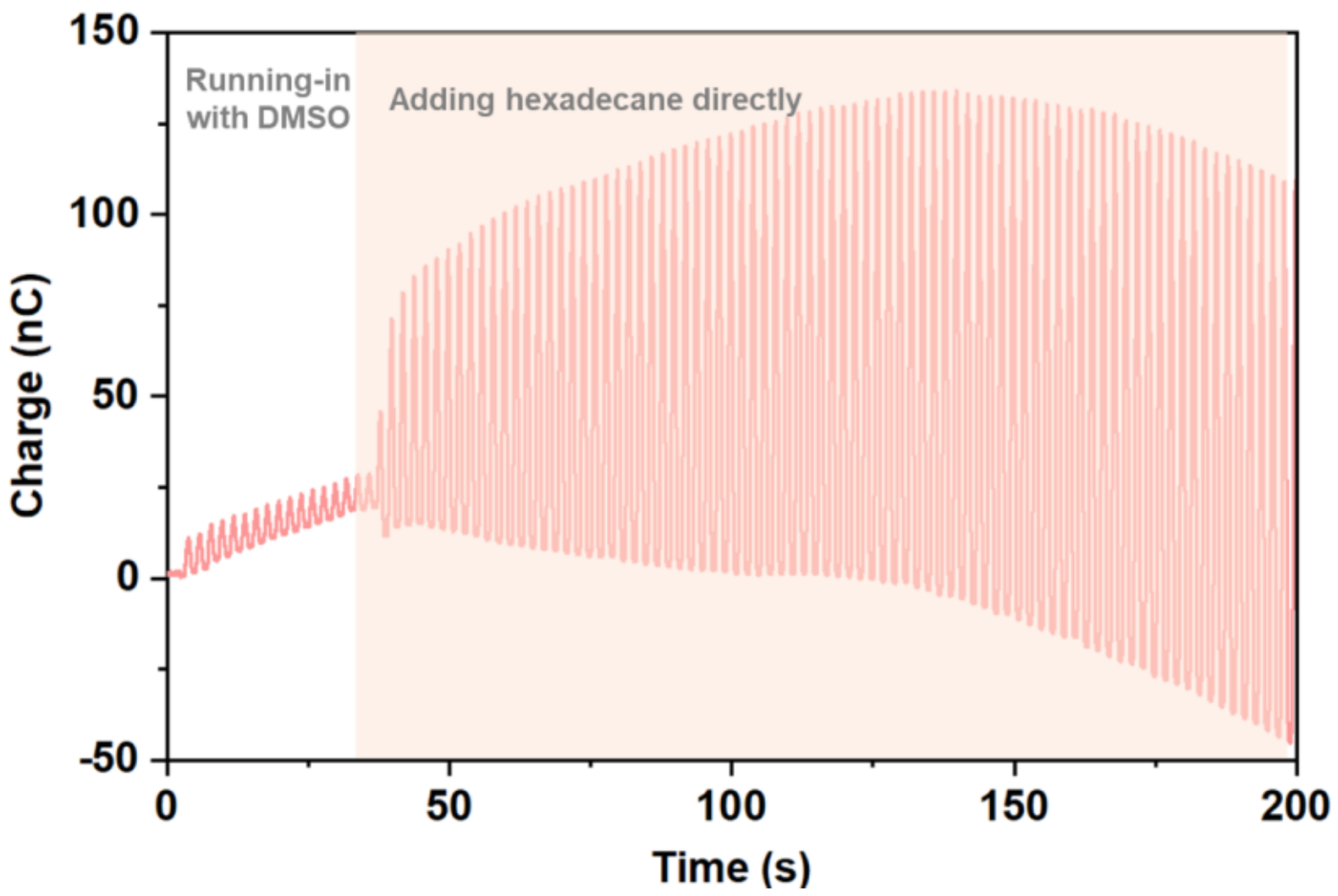

Figure S45 Charge output of the TENG through running-in with DMSO and adding hexadecane.

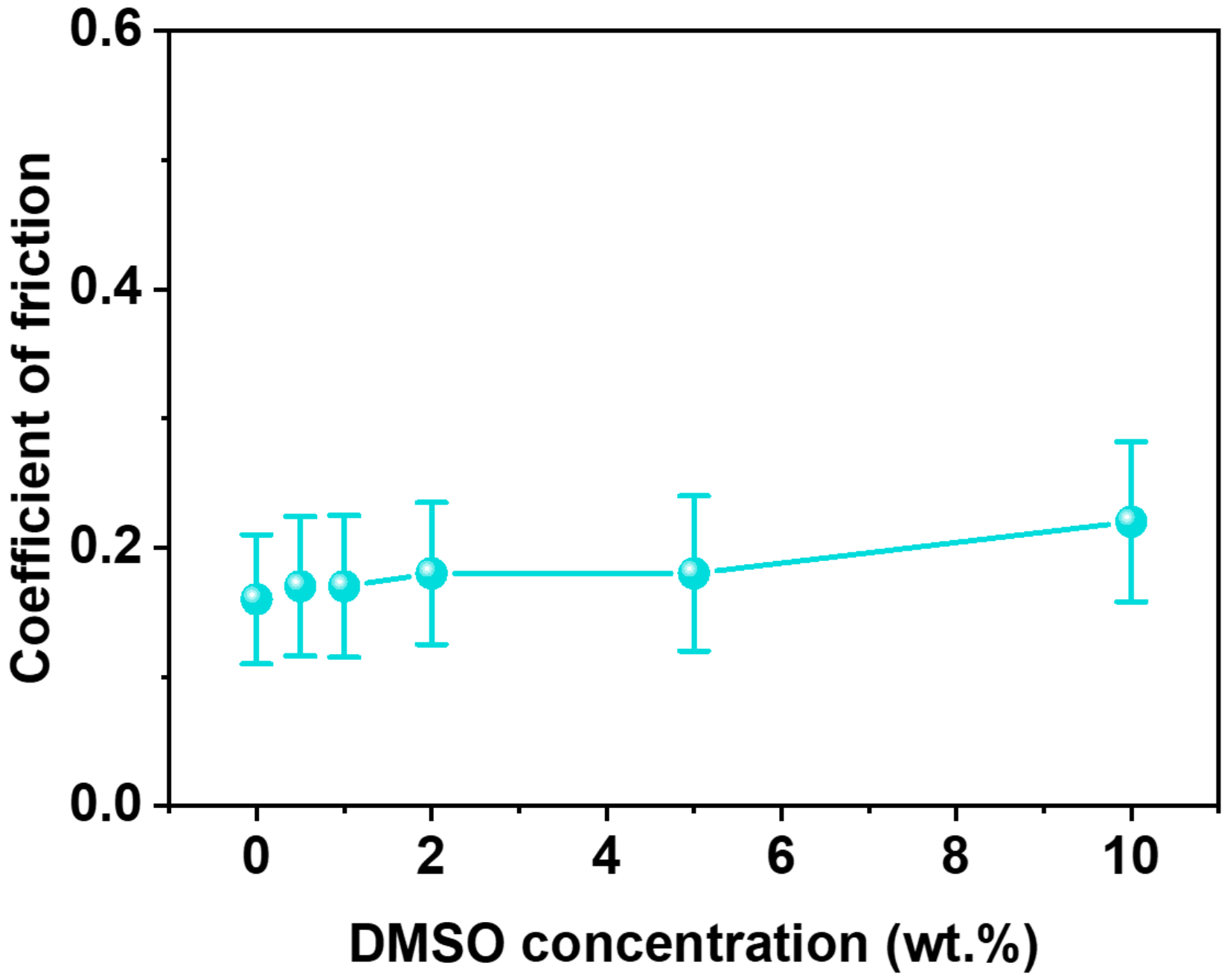

Figure S46 COF of the friction pairs of virgin PI/Al as a function of DMSO concentration mixed in hexadecane.

Table S1 Atomic concentration of the PI surfaces (at.%)

	C1s	N1s	O1s	Al2p	S2p
**Virgin PI**	84.46	1.37	13.96	0.06	0.15
**Runing-in with dry friction**	77.89	1.36	20.41	0.05	0.29
**Running-in with hexadecane**	72.57	2.18	25.08	0.00	0.17
**Running-in with DMSO**	75.82	6.98	17.10	0.00	0.11

References

1. Zhou, Linglin, et al. "Simultaneously enhancing power density and durability of sliding-mode triboelectric nanogenerator via interface liquid lubrication." *Advanced Energy Materials* 10.45 (2020): 2002920.
2. Shi, Lin, et al. "High-performance triboelectric nanogenerator based on electrospun PVDF-graphene nanosheet composite nanofibers for energy harvesting." Nano Energy 80 (2021): 105599.
3. Liu, Lu, et al. "Achieving Ultrahigh Effective Surface Charge Density of Direct-Current

Triboelectric Nanogenerator in High Humidity." *Small* 18.24 (2022): 2201402.

4. Zhang, Zhi, et al. "Semiconductor contact-electrification-dominated tribovoltaic effect for ultrahigh power generation." *Advanced Materials* 34.20 (2022): 2200146.
5. He, Wencong, et al. "Capturing dissipation charge in charge space accumulation area for enhancing output performance of sliding triboelectric nanogenerator." *Advanced Energy Materials* 12.31 (2022): 2201454.
6. Wu, Huiyuan, et al. "A constant current triboelectric nanogenerator achieved by hysteretic and ordered charge migration in dielectric polymers." *Energy & Environmental Science* 16.11 (2023): 5144-5153.
7. Chen, Ai, et al. "A novel hybrid triboelectric nanogenerator based on the mutual boosting effect of electrostatic induction and electrostatic breakdown." Energy & Environmental Science 16.8 (2023): 3486-3496.
8. Li, Qianying, et al. "Overall performance improvement of direct-current triboelectric nanogenerators by charge leakage and ternary dielectric evaluation." Energy & Environmental Science 16.8 (2023): 3514-3525.
9. An, Shanshan, et al. "Boosting output performance of sliding mode triboelectric nanogenerator by shielding layer and shrouded-tribo-area optimized ternary electrification layered architecture." *Small* 19.45 (2023): 2303277.
10. Gao, Yikui, et al. "Achieving high-efficiency triboelectric nanogenerators by suppressing the electrostatic breakdown effect." *Energy & Environmental Science* 16.5 (2023): 2304-2315.
11. Li, Qianying, et al. "A robust constant–voltage DC triboelectric nanogenerator using the ternary dielectric triboelectrification effect." *Advanced Energy Materials* 13.2 (2023): 2202921.
12. Gao, Yikui, et al. "Achieving high performance triboelectric nanogenerators simultaneously with high-voltage and high-charge energy cycle." *Energy & Environmental Science* 17.22 (2024): 8734-8744.
13. Cao, Zeyuan, et al. "Ultrahigh power generation from low-frequency and micro motion by suppressing air breakdown." *Energy & Environmental Science* 17.14 (2024): 5002-5012.
14. Gao, Yikui, et al. "Spontaneously established reverse electric field to enhance the performance of triboelectric nanogenerators via improving Coulombic efficiency." *Nature*

Communications 15.1 (2024): 4167.

15. Sun, Yushan, et al. "Enhancing performance of triboelectric nanogenerator by accelerating the charge transfer strategy." *Nano Energy* 121 (2024): 109194.
16. He, Lixia, et al. "Dynamic interfacial electrostatic energy harvesting via a single wire." *Science Advances* 10.24 (2024): eado5362.
17. Yi, Hongyu, et al. "Efficient Interfacial Electrical Energy Extraction of a Triboelectric Nanogenerator by the Charge Lock-Free Strategy." *Advanced Materials* (2025): 2503598.
18. Hu, Dongyang, et al. "An ultrahigh performance hybrid energy harvester leveraging the induced charge excitation strategy." *Energy & Environmental Science* 18.10 (2025): 4665-4676.
19. Guchait, Aparna, et al. "Tannic Acid Modified Epoxidized Natural Rubber Based High Performing Triboelectric Nanogenerator." *Small* 21.13 (2025): 2411410.
20. Liu, Xiaoru, et al. "Superior charge density of triboelectric nanogenerator via trap engineering." *Advanced Functional Materials* 35.14 (2025): 2416944.
