# Supplementary material for: Solvent-assisted running-in strategy enables improved triboelectric nanogenerator output
Source: Nat Commun. 2026 Jun 11;17:8971. doi: 10.1038/s41467-026-74269-5 (PMC13507328; doi:10.1038/s41467-026-74269-5)
Supplement: Supplementary file 2 [file 41467_2026_74269_MOESM2_ESM.pdf]

Description of Additional Supplementary Files

Supplementary Movie 1: DMSO-assisted extraction of broken PI chains during running-in. Molecular dynamics trajectory showing DMSO molecules infiltrating the running-in PI surface and extracting polar amide-containing chain fragments toward the contact interface

Supplementary Movie 2: Interaction between DMSO and virgin PI. Molecular dynamics trajectory showing that, without running-in-induced bond scission, imide groups remain largely confined within the PI molecular chains and are not extracted toward the contact interface under the simulated conditions.
