# Supplementary material for: Solvent-assisted running-in strategy enables improved triboelectric nanogenerator output
Source: Nat Commun. 2026 Jun 11;17:8971. doi: 10.1038/s41467-026-74269-5 (PMC13507328; doi:10.1038/s41467-026-74269-5)
Supplement: Supplementary file 3 [file 41467_2026_74269_MOESM5_ESM.pdf]

nature portfolio

Peer Review File

Solvent-assisted running-in strategy enables improved triboelectric nanogenerator output

Corresponding Author: Professor Jun Zhao

Parts of this Peer Review File have been redacted as indicated to remove third-party material.

This file contains all reviewer reports in order by version, followed by all author rebuttals in order by version.

Version 0:

Reviewer comments:

Reviewer #1

(Remarks to the Author)
This manuscript titled "Achieving superhigh output of triboelectric nanogenerator on engineering materials via optimizing running-in process" proposed a solvent-assisted running-in strategy to enhance the interface lubrication of triboelectric nanogenerators (TENGs). The authors confirmed that friction force induces the breakage of the relatively unstable N-C bonds in PI during the running-in period, resulting in the freedom releasing of amide groups. The operational mechanism underlying the solvent-treated running process is deeply elucidated comprehensively by advanced molecular dynamics (MD) simulations and density functional theory (DFT) analyses, providing deep insights at the molecular and electronic levels. Please find the specific comments and questions below.

1. While this study demonstrates the effectiveness of solvent-assisted running-in for enhancing TENG performance, it does not provide any discussion on how such a process could be automated.

2. Although rapid improvements have been achieved through the running-in process, long-term stability under repeated cycling or environmental stresses (humidity, temperature, etc.) has not been clearly established.

Reviewer #2

(Remarks to the Author)
Review Comment
In the manuscript entitled "Achieving superhigh output of triboelectric nanogenerator on engineering materials via optimizing running-in process", the authors reported a new method to generate the output from the sliding mode TENG. The authors explained the new device operation for the sliding mode based triboelectrification using solvents and lubricants in running process. To explain the phenomenon, chemical characterizations, molecular simulation and DFT were studied in the manuscript. However, the fundamentals are not properly covered in the present version of the manuscript. The present version of the manuscript is not suitable for publication in Nature Communications. However, the authors should consider the following comments to improve the manuscript for acceptance.
In Figure 2b, the authors explained the proposed sliding mode-based triboelectrification. It contains three steps, running-in period (solvent were used), cleaning interface (removing the solvent) and triboelectric period (applying the lubricants) were used to the measurements. Usually, triboelectrification works on the coupled mechanism of contact electrification and electrostatic induction. Here, active tribolayers Al is making contact with the tribolubricant rather than the active layer PI. In that case, how are the authors claiming the triboelectrification between the Al and PI. And how the electrostatic induction happening between the active layer to the electrodes is not explained by the authors in the manuscript.
The first principle theory of nanogenerator ($J_D \approx \sigma_{(T)} \times dH/dt \times ((d_1 \in_0)/\in_1 + (d_2 \in_0)/\in_2)/((d_1 \in_0)/\in_1 + (d_2 \in_0)/\in_2 + z)^2$) suggest the triboelectrification can be affected by three components first part $\sigma_{(T)}$ is related to the charge density of the triboactive materials, the second part dH/dt is related to the operation of the TENG device, and the third part describes the electrostatic induction. P1 and P3 are related to the material property, and P2 are related to the structural property and operation of the device. The authors claim that there will be a change in the chemical structure of PI (line no.

244 to 278). These chemical changes can affect the surface charge density and dielectric properties of the materials. The authors should study the surface charge density using KPFM analysis and dielectric analysis of all steps to explain triboelectrification in a better way. The change in the device operation of sliding mode can affect the second part of the equation. So, the author should explain the same in the manuscript.
In line no. 271 to 278, the authors explained the Mulliken charge of analysis of the carbon, hydrogen, oxygen and nitrogen of the different bindings (-CO-N-C, -CHO, -COOH, -NH-C). The authors claimed that the high negative Mulliken charge means the electron acceptance behaviour. These changes in charge are due to the change of binding atom to the oxygen, carbon and nitrogen. The electron withdrawal from an external surface is different from this one. To understand the electron-withdrawing behaviour of the active layer, the author should model another tribolayer and study interaction with the different models (includes the bindings -CO-N-C, -CHO, -COOH, -NH-C) by analysing the Mulliken charge before and after interaction. Rather than atomic charge alteration, the authors should focus on the whole binding (-CO-N-C, -CHO, -COOH, -NH-C)
In Figure 5 b, the authors reported the longer cycle run of the device. After the 1800s, there was a gradual decrease in the device's performance. Why decrement in the output observed, the authors should explain in detail. It shows the poor stability of the device, which is usually for the TENG stability.
In supplementary figure S21, the authors reported the peak power density analysis. It shows that peak power density is obtained at 5Gohm. It shows the high impedance of the device. Why is it high, and how to control the phenomenon with the proposed method? And the authors have used the unit W m-2 Hz-1, which formula authors have used to calculate the power and power density.
The authors have provided the charge density analysis of the TENG by optimising the running-in process. The authors should add the output performance in terms of voltage and current in the same way.

Reviewer #3

(Remarks to the Author)
The Authors present a work describing novel strategies to reach high performances in polyimide (PI)/aluminum triboelectric nanogenerators (TENGs), focusing in particular to the “running-in” pre-processing of the device. Treatment with DMSO polar, aprotic, solvent at the interface allowed the removal of the species formed due to friction which could interfere with charge separation. Moreover, both an increase of the effective interaction area, as well as C-N bonds breakage on the PI surface and exposure of electron-withdrawing groups, contribute to obtain a remarkably high charge density at the interface, if compared with current state-of-the-art of TENGs devices.
The results are indeed interesting, also for their technological implications. Moreover, the suggested mechanism is convincingly supported by experimental evidence (SEM analysis of the surfaces, XPS and mass spectrometry characterization of surface groups) and computational simulations (molecular dynamics of PI polymer with DMSO, electronic structure calculations of cleaved PI on Al surface). I have only minor questions before recommending for publication in Nature Communications:
1. Authors state that the obtained charge density is higher than the “intrinsic charge density of PI” (ref. 17). Can Authors describe more in detail what the “intrinsic charge density” refers to?
2. Please make the EDS acronym explicit when first used in the text.
3. The S-N RDF is reported in Fig. 4d (and not Fig. 3d, as stated in the text), while S-O RDF is in Fig. 4c (and not Fig. 4d).
4. In the PI – Al surface modelling through DFT calculations, are two Al layers (as shown in Figure 4 and S17) sufficient? Has the inner surface of the slab been passivated (for instance with (pseudo)hydrogens)?
5. In PI models with broken C-N bonds, it seems that C=O (cleavage in the imide moiety) or NH (cleavage of the N – benzene bond) strongly interact (with coordinate covalent bonds) with Al surface atoms (according to Figures 4 and S17). Since C=O groups in unbroken PI could, in principle, also coordinate Al, but this is not observed in the model, could Authors give more details about possible reasons for this different binding behavior at the interface? Does the higher flexibility of the broken PI contribute to some extent?
6. In the simulation of the friction between PI and Al, Authors say that a “virtual aluminum (Al) substrate” was employed. Can they give more details about this virtual substrate approach?
7. Has the ReaxFF force field employed in MD simulations to allow bond breakage been specifically re-parametrized for the PI system, or have the default parameters been employed?
8. Some sentences in the text are slightly unclear: “The dissolvents have the functionalization of carrying worn free radicals and debris…” (function, pg. 3); To further understand how the DMSO-assisted solvent activation…” (pg. 10); “there is a significant peak of electron loss near the side of Al near the interface of Al/PI.” (pg. 13); “…the nearby PI molecules gain electrons are also obvious…” (electrons gain, pg. 13); “And, the peak current density and average power of the TENG were measured with different resistances.” (pg. 15); “…making friction and wear of tribo-materials reduction.” (pg. 16); “The raking of the running-in PI after wear changes behand to virgin PTFE in the triboelectric series…” (ranking, behind, pg. 17).

Reviewer #4

(Remarks to the Author)
This manuscript reports an optimization running-in process with various solvents in enhancing triboelectric output. In particular, non-polar solvents like DMSO, DMA and DMF are highly recommended in removing the worn radicals and debris from the running-in process. The proposed idea is interesting, particularly with the use of liquid lubricant enabling the efficient charge transferred in a short time and support by the MD simulation. However, the study requires further detailed analysis and discussion to support the conclusions drawn. Specific comments are as follows:

1. The ratio of the real contact to the nominal contact area using the simulation calculation required more detail information to support the quantitative analysis of contact stress analysis after the running in with hexadecane and DMSO as shown in Figure 3b-e. It is required to align the terminology between the evaluated surface roughness and the simulation calculation for clarity and consistency.

2. Are there any depth profiling results of the TOF-SIMS to support the quantitative contribution of physical and chemical effects of triboelectricity of DMSO running-in optimization as claim in the conclusion? It is essential to show the depth profiling results using TOF-SIMs as a function of the number of cycles of running-in to correlate the spatial distribution of different elements and molecules on the surface and determine the composition of the sample layer by layer.

3. In Figure 5d-f, the charge reversal mechanism was demonstrated with the voltage and charge measurement and a triboelectric series for lubricated sliding modes which considered the running-in optimization is proposed. The authors are required to perform the surface potential evaluation and the surface roughness using the KPFM and AFM, respectively to provide more details on the minimum energy barrier and number of running-in cycles in relation to friction efficiency are required for polarization "switching" in each of the sample as claimed. Additionally, the key factors that influence polarization "switching" should be clearly outlined.

4. On simulation part, the flow of the results and discussion is disrupted, not easy to follow. Specifically, the effects of the non-polar and polar solvent on the charge transfer/shielding mechanism are not presented in a clear and intuitive manner. The overall discussion of the MD & DFT simulation (mainly focusing on DMSO only) to support the experimental observation lacks cohesion. The authors should reorganize the narrative to improve the flow and ensure that the progression of ideas is logical and easier to follow.

Reviewer #5

(Remarks to the Author)
The manuscript presents a novel surface modification approach aimed at enhancing the performance of TENGs. The use of dissolvent-assisted running-in processes, particularly involving DMSO and hexadecane, contributes a degree of innovation. However, the overall presentation suffers from a lack of organization, insufficient data analysis, and weak comparisons to existing literature. Several key claims are not adequately substantiated, and multiple aspects of the methodology require clarification and further validation. Below are detailed comments and suggestions for revision before its acceptance for publication.
1. Lack of Experimental Support for Dissolvent Mechanism:
The manuscript asserts that the use of dissolvents facilitates removal of surface radicals and debris while preventing material transfer. However, this claim is not backed by direct experimental evidence. The authors are suggested to provide a comprehensive surface analysis (e.g., XPS, AFM, SEM) and charge transfer characterization across all sample sets, ideally including a calibrated reference sample. The influence of environmental parameters such as humidity and chemical environment must also be addressed.
2.Insufficient Evidence for Superhigh Charge Density and In-Running Modification:
The reported "superhigh" charge densities and in-running modification phenomena require stronger justification. The authors suggest that ion release (e.g., from $BMImBF_4$) may shield the surface, but no direct measurements support this. Detailed electrochemical and spectroscopic evidence (e.g., ion concentration, interfacial ion accumulation) is needed to validate this hypothesis.
3. Surface Potential Analysis Missing:
Since surface potential critically governs TENG output, both bulk and localized surface potential distributions should be analyzed (e.g., KPFM, surface voltmeter mapping). These results would provide direct insight into the impact of surface modification on device performance.
4.Lack of Comparative Performance Metrics:
A detailed comparison of voltage, current, power density, and internal resistance before and after treatment is necessary, especially in relation to standard TENG configurations. Current values appear lower than in comparable literature, despite higher power densities. The authors should clarify whether this discrepancy results from changes in internal resistance or other parameters.
5.Internal Resistance and Calibration Methodology:
The reported intrinsic charge density (2.5 mC m^{-2}) after DMSO-assisted running-in lacks methodological transparency. There is no clear reference to standard calibration techniques (e.g., air breakdown suppression, guard-ring electrode geometries). Without proper calibration, fair comparison with other TENGs becomes unreliable.
6. Device Longevity and Wear:
Long-term stability is not addressed. A durability study (e.g., $>10^4$ cycles) is needed to demonstrate whether performance is stable or degrades over time. It would also be useful to report any physical degradation (e.g., thinning of the plastic film) post-cycling.
7. Limitations of MD System Size:
The molecular dynamics simulations utilize short-chain PI (DP = 4, 25 chains), which likely exaggerates surface mobility and fragmentation. The authors should either increase chain length and system size or include coarse-grained simulations to verify that the results generalize to bulk-like behavior.
8. Charge Accumulation and Interfacial Leakage:
The role of interfacial permittivity and conduction in charge generation must be rigorously examined. High apparent Q values may result from displacement or leakage currents through residual solvent films. Recommended tests include open-circuit decay, leakage I–V, frequency scaling, and blocking capacitor checks to eliminate non-triboelectric contributions.

9. Aluminum Surface Representation:
Both MD and DFT simulations model bare aluminum surfaces, while experiments inevitably involve native Al_2O_3 layers and tribocorrosion. The relevance of pure Al to the actual interfacial chemistry is questionable. The authors are encouraged to simulate Al_2O_3 surfaces or discuss the limitations of neglecting this oxide layer.
10. Solvent–Polymer Interaction Mechanism Unclear:
The proposed mechanism of targeted N–C scission and solvent extraction lacks rigorous evidence. The authors must provide details of the ReaxFF parameterization, validate fragmentation pathways (e.g., energy distribution, scission probability), and compare with DFT-derived reaction barriers. Sensitivity to time step and thermostat parameters should also be reported.
11.Lack of Detail in FEM Modeling:
The finite element model references fractal surfaces and Johnson–Cook plasticity, but no parameter values, mesh independence, or validation (e.g., via profilometry) are provided. A power spectral density (PSD) of the roughness profiles, mechanical parameters (e.g., yield modulus), and uncertainty analysis on contact area are essential for credibility.
12.Electrostatics and Boundary Conditions in MD:
Simulations involving vacuum interfaces and long-range electrostatics (PPPM) should include corrections (e.g., Yeh–Berkowitz, non-periodic z-direction) to prevent spurious dipole interactions. Failure to do so may compromise RDFs and interfacial behavior predictions.
13. DFT: Solvent Effects Not Captured
The DFT modeling assumes vacuum interfaces, despite the key role of DMSO in the hypothesized mechanism. The authors should incorporate solvent effects explicitly (e.g., via VASPsol or explicit DMSO layers) and recompute key parameters such as LUMO localization, charge transfer, and interfacial dipole moments.

Version 1:

Reviewer comments:

Reviewer #1

(Remarks to the Author)
The reviewer recognizes that significant effort has been made to revise the manuscript, and that many aspects have been strengthened through additional experiments, analyses, and clarifications. These revisions have clearly improved the overall quality of the work. Nevertheless, despite these improvements, the manuscript still does not achieve the level of novelty and broad impact expected for publication in Nature Communications. The reported advances, while technically solid, remain incremental and are unlikely to substantially change current understanding or paradigms in the field.

Reviewer #2

(Remarks to the Author)
During the revision, the authors have asked to revise the manuscript with additional experiments and scientific discussions. The authors have addressed all the comments with the point by point response. The present version of the manuscript is suitable for publication.

Reviewer #3

(Remarks to the Author)
I recommend the revised manuscript for publication in Nature Communications.

Reviewer #4

(Remarks to the Author)
The authors have adequately addressed the reviewers' comments with appropriate scientific justification. The manuscript is recommended for publication in Nature Communication.

Open Access This Peer Review File is licensed under a Creative Commons Attribution 4.0 International License, which permits use, sharing, adaptation, distribution and reproduction in any medium or format, as long as you give appropriate credit to the original author(s) and the source, provide a link to the Creative Commons license, and indicate if changes were made.
In cases where reviewers are anonymous, credit should be given to 'Anonymous Referee' and the source.
The images or other third party material in this Peer Review File are included in the article's Creative Commons license, unless indicated otherwise in a credit line to the material. If material is not included in the article's Creative Commons license and your intended use is not permitted by statutory regulation or exceeds the permitted use, you will need to obtain permission directly from the copyright holder.
To view a copy of this license, visit https://creativecommons.org/licenses/by/4.0/

Dear editor, Dear reviewers

We gratefully thank the editor and all reviewers for their time spend making their constructive remarks and useful suggestions, which has significantly raised the quality of the manuscript and has enabled us to improve the manuscript. We have studied comments carefully and have made corrections which we hope meet with approval. Below the comments of the reviewers are response point by point and the revisions are indicated.

The black words are the comments provided by the reviewers.

The blue ones are the responses.

The green ones are the revised part in the manuscript and the supplementary information document.

Reviewer: 1

This manuscript, titled "Achieving superhigh output of triboelectric nanogenerator on engineering materials via optimizing running-in process" proposed a solvent-assisted running-in strategy to enhance the interface lubrication of triboelectric nanogenerators (TENGs). The authors confirmed that friction force induces the breakage of the relatively unstable N-C bonds in PI during the running-in period, resulting in the freedom releasing of amide groups. The operational mechanism underlying the solvent-treated running process is deeply elucidated comprehensively by advanced molecular dynamics (MD) simulations and density functional theory (DFT) analyses, providing deep insights at the molecular and electronic levels. Please find the specific comments and questions below.

1. While this study demonstrates the effectiveness of solvent-assisted running-in for enhancing TENG performance, it does not provide any discussion on how such a

process could be automated.

1. Response: Thanks so much for your valuable comments. The automated running-in with solvent assistant is important for the practical application of TENGs. This study has potential for automated operation, because the promoted solvent-assisted running-in action in this study is easy to operate. At the same time, it is found in Fig. 5a that an ultrahigh charge density of ~2.2 mC m^{-2} is achieved with just one running-in cycle. In addition, the output efficiency of the TENG can also be greatly improved under the lubrication of the hexadecane containing a small amount of DMSO directly (Fig. 5g), i.e., it is not necessary to handle the optimization of the running-in process separately.

To achieve the highest electric output, the contact surfaces are fully cleaned manually to remove the residual DMSO after the running-in process shown in Fig. 1b. Whereas another test is carried out in this study without any cleaning, which also shows a high electric output (~1.2 mC m^{-2}) (Supplementary Fig. S45). It means the solvent-assisted running-in process could be further simplified. Thereby, the promoted solvent-assisted running-in in this study is easy to operate with high potential for automated operation.

Changes as follow:

Page 20

For the above tests, after the running-in process, the contact surfaces are then fully cleaned manually to remove the residual DMSO (Fig. 1b). Another electric test is carried out here without any cleaning action. It is found that there is still a high electric output (~1.2 mC m^{-2}) when the hexadecane lubricant (~1 mL) is directly added after running-in with a low volume of DMSO (~0.2 mL) (Supplementary Fig. S45), meaning the solvent-assisted running-in process could be further simplified.

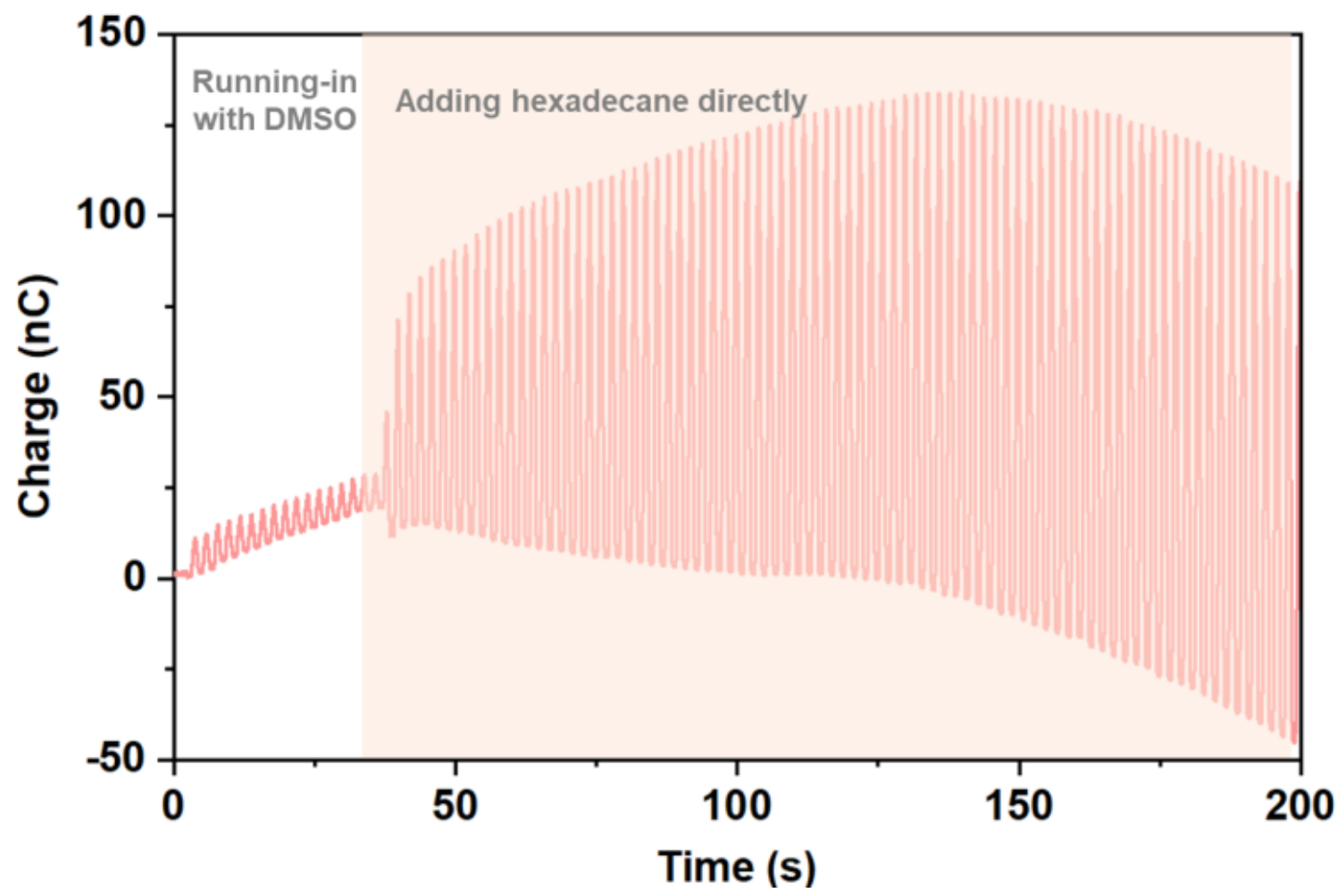

Figure S45 Charge output of the TENG through the running-in with DMSO followed by directly adding hexadecane.

2. Although rapid improvements have been achieved through the running-in process, long-term stability under repeated cycling or environmental stresses (humidity, temperature, etc.) has not been clearly established.

2. Response: Thanks so much for your meaningful comments. The long-term stability of TENGs is very important for the practical application, which has been studied in this work as shown in Fig. 5e and Supplementary Fig. S44. After more than 10 hours (~18 000 cycles), the lubricated TENG exhibits superior electric durability maintaining 86% output (>2 mC m^{-2}) which is much higher than previous works (Fig. 2d). In contrast, the same TENG device retains only 9% electric output under dry friction in air (Fig. 5e).

In addition, the influence of environmental stresses, e.g., gas, humidity, and temperature, on the electric performance is also carried out in the revised manuscript. As shown in Supplementary Fig. S41a, the electric output of the lubricated TENG maintains a relatively high retention rate of 90% under a high humidity of 60%RH. In contrast to the TENG under dry friction, the lubricated TENG has a much better performance of humidity resistance. It is because the electric interface is fully covered with the lubricant so that the water is difficult to diffuse into the Al tribolayer and the PI surface[1, 2]. Further, it is found that the gas atmospheres e.g., O_2, N_2 and CO_2 have

little impact on the electric output of the lubricated TENGs (Supplementary Fig. S41b-d), which is very different from that of the TENG under dry friction. At the beginning of dry friction, the effect of O_2 on the TENGs is not obvious when the concentration is lower than 50% (Supplementary Fig. S41b). Whereas, the ratio of voltage output is reduced by about 40% as the O_2 concentration gradually goes up to 70%. Similarly, the electric output gradually decreases with the increase of N_2 concentration (Supplementary Fig. S41c). Both the lubricated and dry-frictional TENGs keep a high and stable electric value in CO_2 atmosphere[3] (Supplementary Fig. S41d). In addition, the study on the environmental temperature on the electrical performance of TENGs is also critical considering the application of TENGs across diverse regions worldwide, where recorded extreme temperature can be as high as 55 °C[4]. The reduction in the output ratio is less pronounced up to 50 °C as shown in Supplementary Fig. S42.

Changes as follow:

Page 20

In addition, the influence of environmental stresses, e.g., gas, humidity, and temperature, is also carried out for the lubricated TENG after the running-in process, in contrast to the TENG under dry friction. As shown in Supplementary Figure S41, the electric output of the lubricated TENG maintains a relatively high retention rate of 90% at a high humidity of 60%RH, which has a much better performance of humidity resistance than that under dry friction. It is because the electric interface is fully covered with the lubricant so that the water is difficult to diffuse into the Al tribolayer and the PI surface[1, 2]. Further, it is found that the gas atmospheres, e.g., O_2, N_2, and CO_2 have little impact on the electric output of the lubricated TENGs after running-in with DMSO (Supplementary Figure S41b-d), which is very different from that of the TENG under dry friction. At the beginning of dry friction, the effect of O_2 on the TENGs is not obvious when the concentration is lower than 50% (Supplementary Figure S41b). Whereas, the ratio of voltage output is reduced by about 40% as the O_2 concentration gradually exceeds 70% because of the adsorption of O_2 onto the contact surface under

dry friction[3, 5]. Similarly, the electric output of the dry-frictional TENG gradually decreases with the increase of N_2 concentration (Supplementary Figure S41c). Both the lubricated and dry-frictional TENGs keep a high and stable electric value in a CO_2 atmosphere[3] (Supplementary Figure S41d). Furthermore, a systematic investigation of the impact of environmental temperature on the electrical performance of TENGs is essential, considering their application across diverse regions worldwide, where recorded extreme temperature can be as high as 55 °C[4]. Although the electric output decreases dramatically with the increase of temperature, the reduction in the output ratio is less obvious up to 50 °C (Supplementary Fig. S42).

Page 20

The long-term stability of TENGs is very important for the practical application, which was examined and shown in Fig. 5e. After more than 10 hours (~18 000 cycles), the lubricated TENG exhibits superior electric durability and maintains 86% output (>2 mC m^{-2}) which is still much higher than previous works (Fig. 2d). Although there is a little attenuation on the electric output, the charge density of the lubricated TENG significantly increases within a fast running-in process of 100 cycles (Fig. 5f). The high electric output of the TENG is attributed to the regulation of amide-containing chemical chains into "molecular brushes" that extend toward the contact surfaces. These outspread groups could be worn during triboelectrification, probably resulting in a decrease in the electric output. In contrast, the same TENG device retains only 9% electric output under dry friction in air. In addition, the coefficient of friction of the dry-frictional TENG is two times as much as that of the lubricated TENG (Supplementary Fig. S44a). The PI surface suffers severe wear after dry friction in contrast to the PI surface with the hexadecane lubrication (Supplementary Fig. S44b). Thus, the TENG with the hexadecane lubrication has good electric durability and tribological performance.

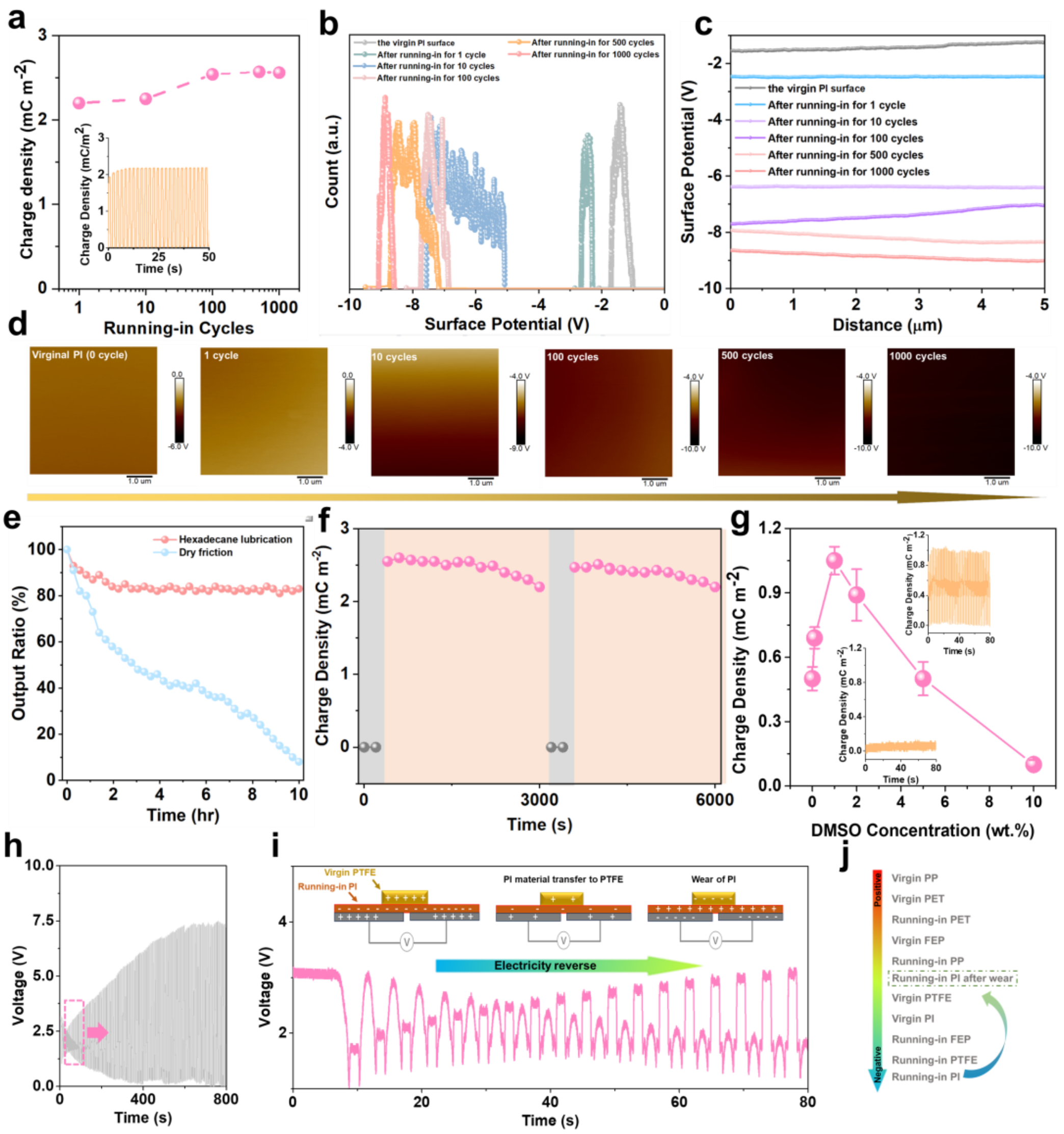

Fig. 5 Super-fast DMSO-assisted running-in strategies towards triboelectric enhancement and reversal effect. a Relationship between the running-in cycle and the triboelectric output. **b** Relative pixel distribution with respect to the surface potential value after running-in with DMSO. **c** Extracted line scan profile with respect to the surface potential value after running-in with DMSO. **d** Surface potential profile of the PI surfaces after running-in with DMSO. **e** Electric outputs of the hexadecane-lubricated TENG after running-in with DMSO compared with the dry-frictional TENG in the long-term test. **f** Charge density of the running-in PI with hexadecane lubrication after intermittent running-in processes. **g** Charge density of the virginal PI lubricated by the mixture of hexadecane and DMSO. **h** and **i** Triboelectric output of the friction

pairs between the running-in PI with virginal PTFE, the inset in Figure 5i demonstrating the electric reversal mechanism of the friction pairs. **j** Triboelectric series of engineering dielectric materials for lubricated sliding modes considering the running-in optimization.

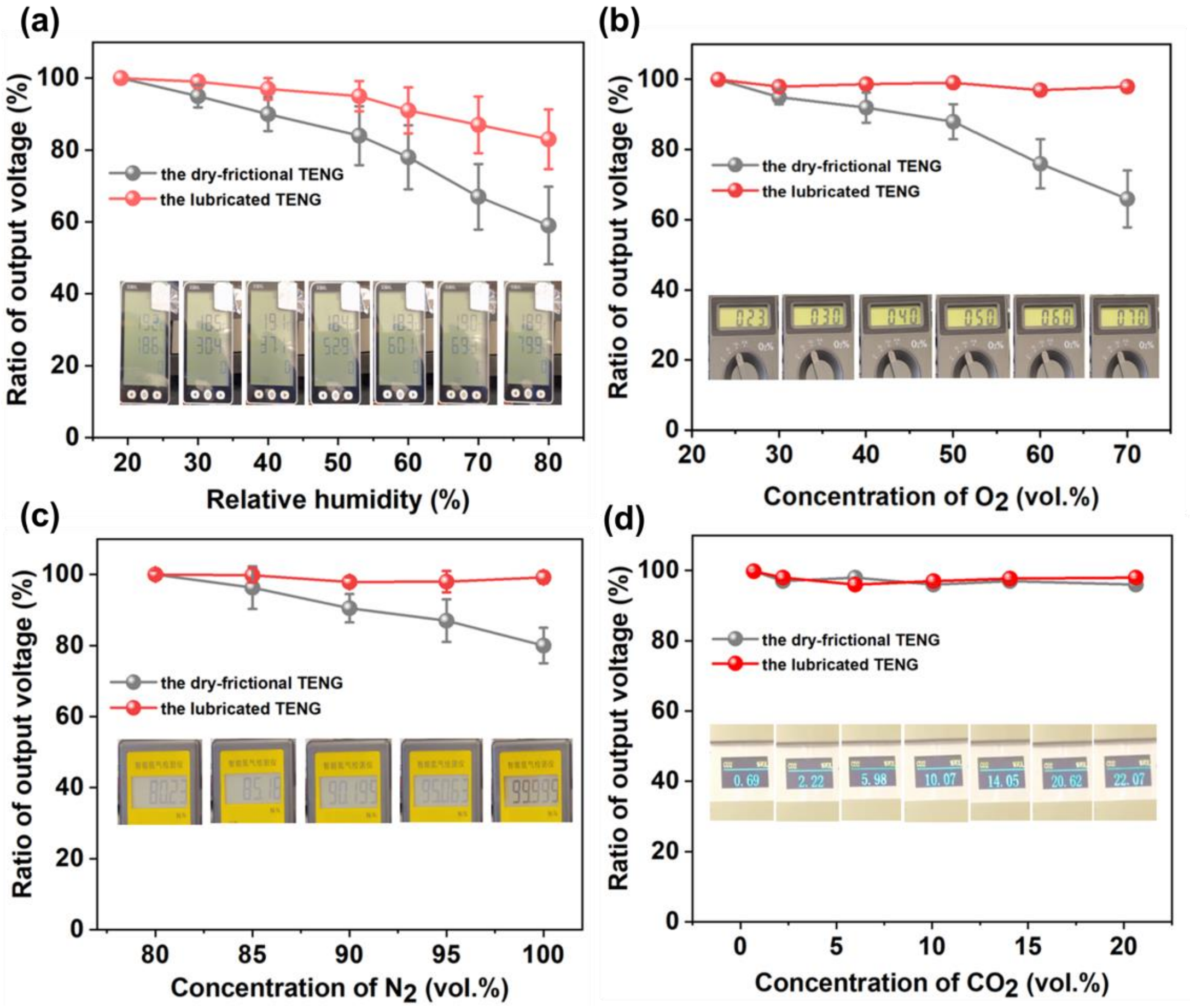

Figure S41 The influence of humidity and gas in air on the electric performance of the TENG with the lubrication of hexadecane after running-in with DMSO in contrast to the TENG under dry friction, relative humidity (a), O_2 (b), N_2 (c), and CO_2 (d).

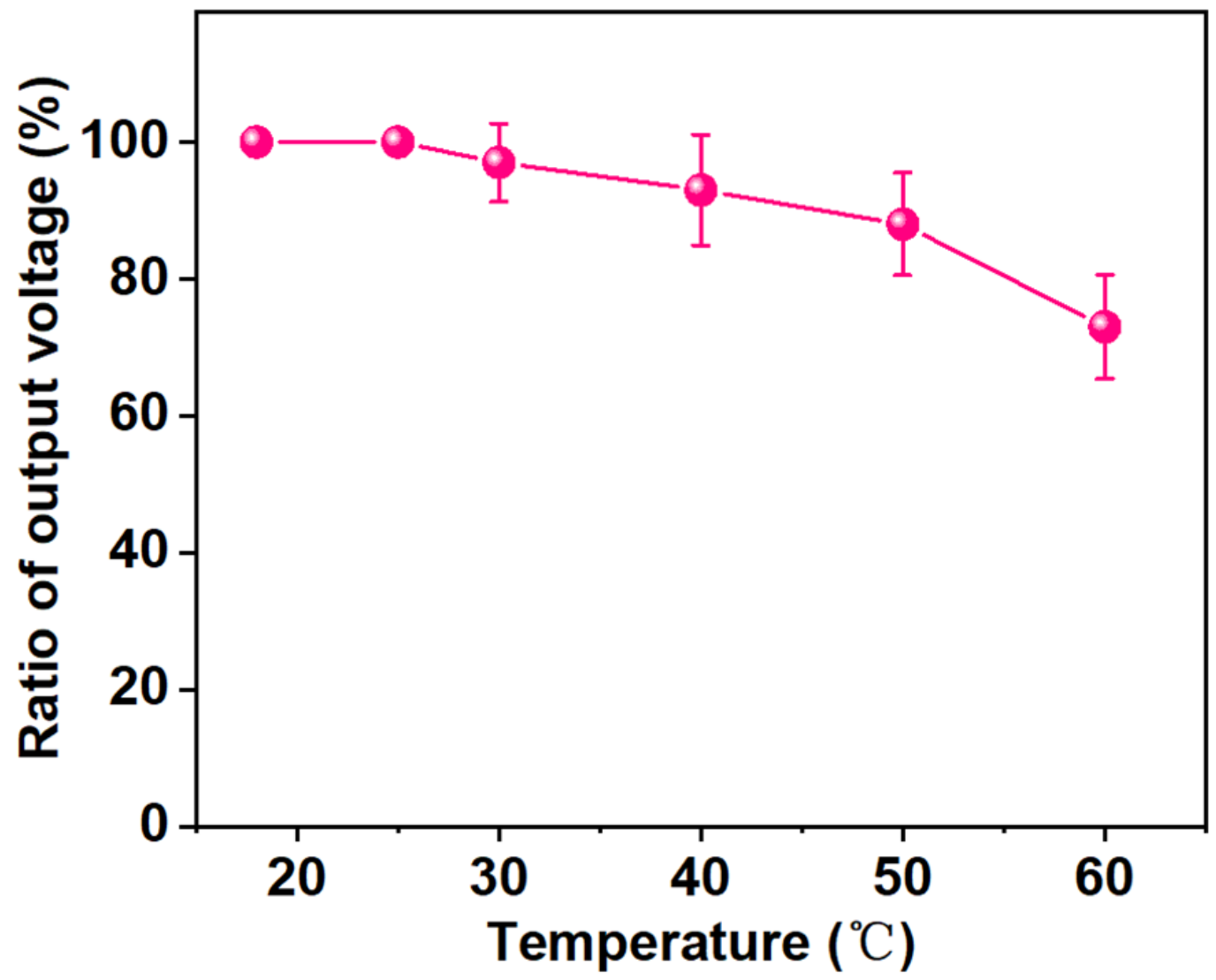

Figure S42 The influence of ambient temperature on the electric performance the TENG with the lubrication of hexadecane after running-in with DMSO at atmosphere (relative humidity ~20%RH).

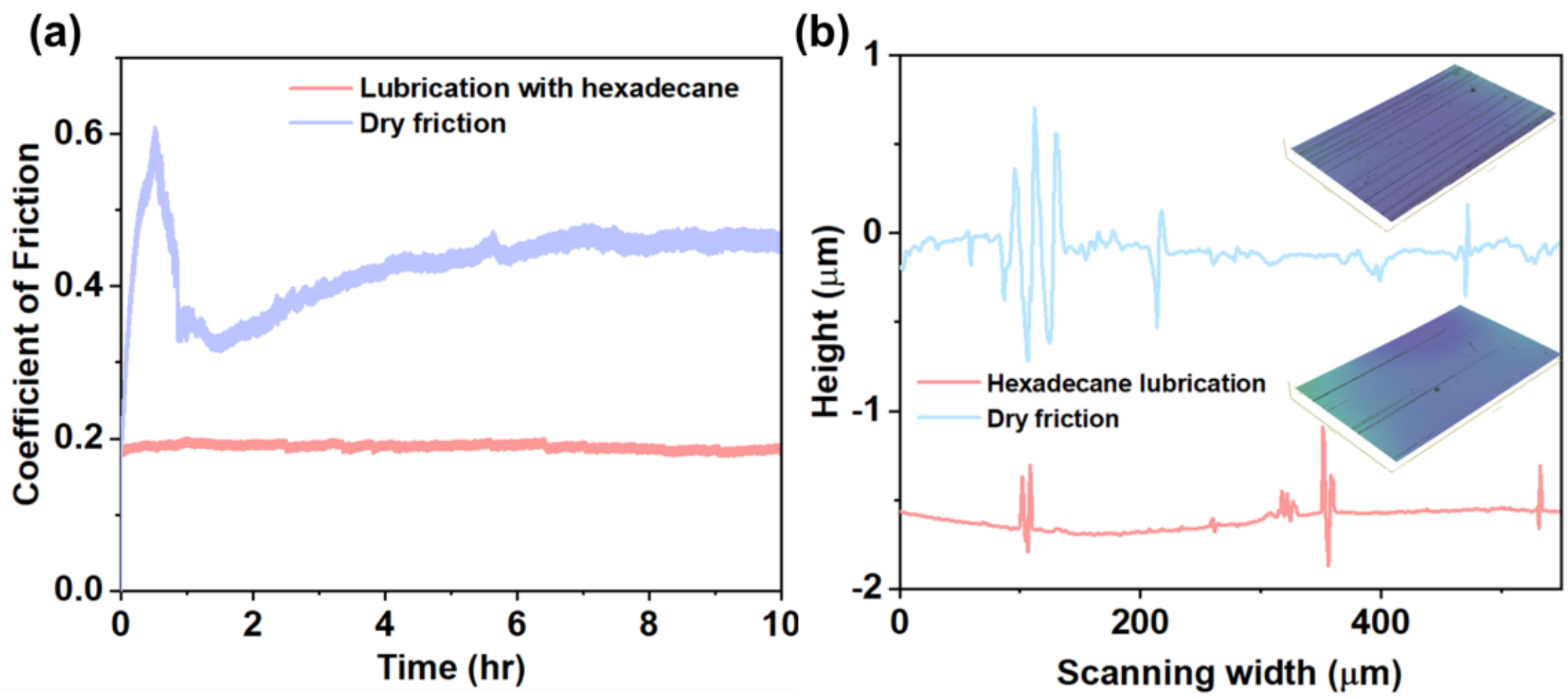

Figure S44 Coefficient of friction (a) and wear depth (b) of the hexadecane-lubricated TENG after running-in with DMSO compared with the dry-frictional TENG in the long-term test, the inset in Fig. S44b showing the 3D morphologies after the long-term test.

References

1. Xiao, K. et al. Improving Triboelectric Nanogenerators Performance Via Interface Tribological Optimization: A Review. *Adv. Funct. Mater.* **34**, 38 (2024).
2. Heo, D. et al. Nano-Oil-Barrier-Based Fluttering Triboelectric Nanogenerator. *Adv. Sci.* **12**, 10 (2025).

3. Li, W.T. et al. Adsorption-induced surface potential modulation and electron transfer of solid-solid triboelectric nanogenerators under gas atmospheres. *Nano Energy* **143**, 12 (2025).
4. Merlone, A. et al. Temperature extreme records: World Meteorological Organization metrological and meteorological evaluation of the 54.0°C observations in Mitribah, Kuwait and Turbat, Pakistan in 2016/2017. *Int. J. Climatol.* **39**, 5154-5169 (2019).
5. Sun, L.L., Lin, S.Q., Tang, W., Chen, X. & Wang, Z.L. Effect of Redox Atmosphere on Contact Electrification of Polymers. *Acs Nano* **14**, 17354-17364 (2020).

Reviewer: 2

In the manuscript entitled "Achieving superhigh output of triboelectric nanogenerator on engineering materials via optimizing running-in process", the authors reported a new method to generate the output from the sliding mode TENG. The authors explained the new device operation for the sliding mode based triboelectrification using solvents and lubricants in running process. To explain the phenomenon, chemical characterizations, molecular simulation and DFT were studied in the manuscript. However, the fundamentals are not properly covered in the present version of the manuscript. The present version of the manuscript is not suitable for publication in Nature Communications. However, the authors should consider the following comments to improve the manuscript for acceptance.

1. In Figure 2b, the authors explained the proposed sliding mode-based triboelectrification. It contains three steps, running-in period (solvent were used), cleaning interface (removing the solvent) and triboelectric period (applying the lubricants) were used to the measurements. Usually, triboelectrification works on the coupled mechanism of contact electrification and electrostatic induction. Here, active tribolayers Al is making contact with the tribolubricant rather than the active layer PI. In that case, how are the authors claiming the triboelectrification between the Al and PI. And how the electrostatic induction happening between the active layer to the electrodes is not explained by the authors in the manuscript.

1. Response: Thanks so much for your valuable comments. Triboelectrification works on the coupled mechanism of contact electrification and electrostatic induction. In dry conditions, electrons are usually transferred from the Al tribolayer to the PI surface during contact electrification. Driven by electrostatic induction, electric outputs will be achieved when the Al tribolayers are sliding against the PI surfaces (Fig. 1a). For the case of lubrication, a thin liquid film (normally less than several tens of nanometers) is formed at the interface[1]. Due to the presence of roughness on contact surfaces, some small liquid pockets are formed. When an external force is applied, the liquid film

ruptures at certain asperities, and solid–solid contact becomes dominant at the electrical interface (at the right of Fig. 1b), which means the contact electrification occurs between the Al tribolayers and the PI surface. Herein, the Al-PI contact is the dominant contributor to the electric outputs for the liquid lubrication conditions. In contrast to dry friction in air (Supplementary Fig. S1), liquid lubricants can significantly suppress interfacial breakdown due to its higher breakdown field strength requirement and lower electrostatic field strength in the microgap between the Al tribolayer and the PI surface. Further, when the charged PI surface is submerged in the lubricant, the surface charge will not be screened by the nonpolar lubricants, e.g., hexadecane, squalane, and paraffin oil, which have a larger Debye length than polar liquids[2]. Because of the electrostatic induction between the charged PI surface and the Al electrode, electrons will flow from the Al electrode to the ground under short-circuit conditions for an electric equilibrium (Fig. 1a).

Changes as follows:

Page 4

Triboelectrification works on the coupled mechanism of contact electrification and electrostatic induction. Under dry conditions, electrons are usually transferred from the Al tribolayer to the PI surface during contact electrification. Electric outputs will be achieved when the Al tribolayer is sliding against the PI surfaces due to electrostatic induction. For the case of lubrication, a thin liquid film (normally less than several tens of nanometers) will be formed at the interface[1]. Due to the presence of roughness on contact surfaces, some small liquid pockets will be generated. When an external force is applied, the liquid film ruptures at certain asperities, and solid–solid contact becomes dominant at the electrical interface (Fig. 1b), which means the contact electrification occurs between the Al tribolayers and the PI surface. In contrast to dry friction in air (Supplementary Fig. S1), liquid lubricants can significantly suppress interfacial breakdown due to its higher breakdown field strength requirement and lower electrostatic field strength in the microgap between the Al tribolayer and the PI surface.

Further, when the charged PI surface is submerged in the lubricant, the surface charge will not be screened by the nonpolar lubricants, which have a larger Debye length than polar liquids[2]. Because of the electrostatic induction between the charged PI surface and the Al electrode, electrons will flow from one Al electrode to another electrode under short-circuit conditions for an electric equilibrium (Fig. 1a).

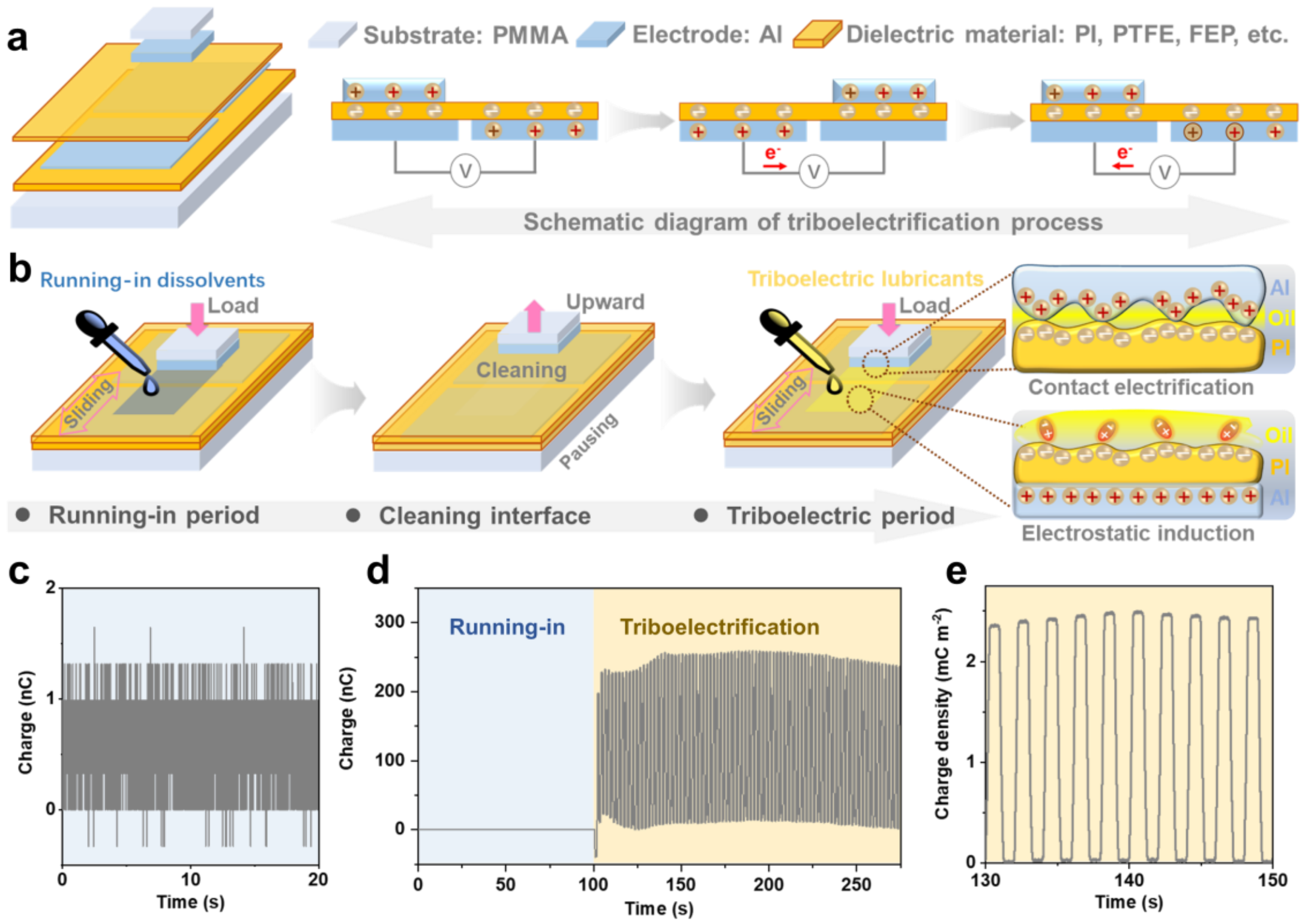

Fig. 1 Demonstration of the TENG via optimizing the running-in process. a Schematic diagram of device structure and triboelectric mechanism. **b** Optimizing process for the running-in and triboelectrification. **c** Electric output during the running-in process. **d** Electric output for the triboelectric period. **e** Charge density during the triboelectric period.

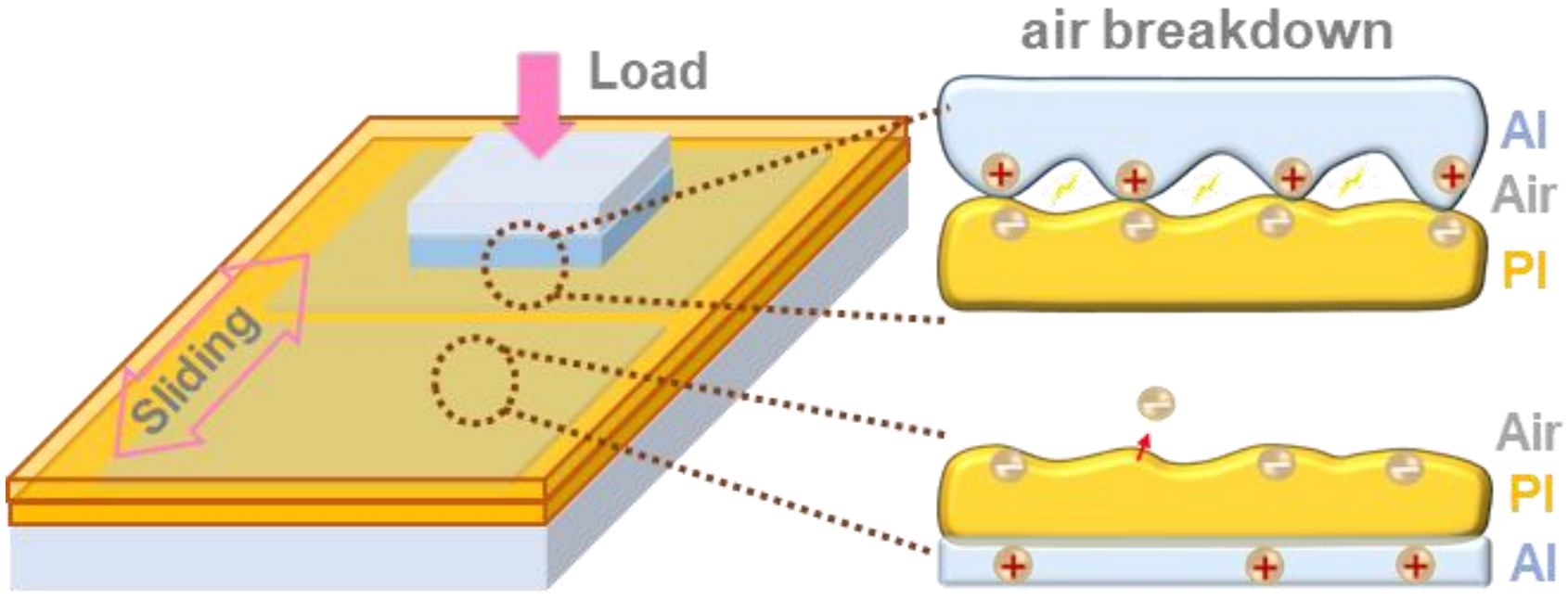

Figure S1 Triboelectrification mechanism diagram of the PI-Al friction pair under dry friction in air.

2. The first principle theory of nanogenerator ($J_D \approx \sigma_{(T)} \times dH/dt \times ((d_1\varepsilon_0)/\varepsilon_1+(d_2\varepsilon_0)/\varepsilon_2)/((d_1\varepsilon_0)/\varepsilon_1+(d_2\varepsilon_0)/\varepsilon_2+z)^2$) suggest the triboelectrification can be affected by three components first part $\sigma_{(T)}$ is related to the charge density of the triboactive materials, the second part dH/dt is related to the operation of the TENG device, and the third part describes the electrostatic induction. P1 and P3 are related to the material property, and P2 are related to the structural property and operation of the device. The authors claim that there will be a change in the chemical structure of PI (line no. 244 to 278). These chemical changes can affect the surface charge density and dielectric properties of the materials. The authors should study the surface charge density using KPFM analysis and dielectric analysis of all steps to explain triboelectrification in a better way. The change in the device operation of sliding mode can affect the second part of the equation. So, the author should explain the same in the manuscript.

2. Response: Thanks so much for your meaningful comments. The electric performance of the TENG is related to material properties, structural properties, and device operation according to the first principle theory of nanogenerators[3]. This study finds high surface charge density of TENGs can be achieved via the DMSO-assisted running-in process that induces surface molecular chain scission and rearrangement, making electron-withdrawing groups toward the triboelectric surface. In addition, the ultrahigh charge density can be achieved with some running-in cycles (Fig. 5a).

Accordingly, the surface potential of the PI surface investigated by KPFM is measured before running-in and after that for different cycles. Shown in Fig. 5b-d, the surface potential changes obviously after the running-in process. From the extracted pixel distribution (Fig. 5b) with respect to the surface potential value, it can be seen that the change occurred immediately after the first cycle, and there is a significant change in the surface potential after running-in for 100 cycles. Thus, the surface potential of the running-in region significantly drops. Accordingly, it is evident from the KPFM measurements that the proposed running-in process leads to a much lower surface potential, which is correlated with a higher output in the TENG. It is because the reduction in surface potential is due to the transfer of charges from the AFM tip to the PI surface[4]. Thus, the more running-in cycles, the more generated charges on the surface, indicating that the running-in PI surface after more cycles has a stronger electron-withdrawing ability. This observation aligns with our findings that the ultrahigh charge density is able to be achieved even with one running-in cycle. After 100 cycles of running-in, the triboelectric polarity of the PI changes to highly negative. It is because the scission and rearrangement of molecular chains occur quickly through the running-in optimization. Accordingly, the surface potential via KPFM measurements can successfully identify the change in the chemical structure of PI surfaces.

The dielectric analysis of the electric materials is also a good way to explain triboelectrification. Scanning force microscopy based on capacitance and electrostatic force is used for measuring the dielectric properties of insulating films at the nanoscale[5-8]. As shown in the following Figure R1, the detection principle involves defining the phase change of the probe under actual vibration state, in contrast to the free vibration phase[9, 10]. In Electric Force Microscopy (EFM), the relationship between the probe vibration phase and the force gradient can be expressed by formula (1) [9, 10].

$$\tan(\varphi) \propto -\frac{Q}{K}F' \quad (1)$$

where φ is the phase of the probe vibration, K is the spring constant of the probe, Q is the quality factor, and F' is the force gradient acting on the probe. Under identical

testing conditions and with the same probe, K and Q can be regarded as constants. The long-range force acting on the probe is dominated by the capacitive force between the probe and the sample, which can be expressed by formulas (2) and (3) [11].

$$F = \frac{1}{2}\frac{\partial C}{\partial Z}\left(V_{tip} - V_S\right)^2 \quad (2)$$

$$F' = \frac{1}{2}\frac{\partial^2 C}{\partial Z^2}\left(V_{tip} - V_S\right)^2 \quad (3)$$

where V_{tip} is the probe voltage, V_S is the surface potential of the sample, Z is a lifting height, the distance between the probe and the sample. C is the system capacitance composed of the probe and sample. In addition to voltage factors, the force gradient F' is mainly determined by the probe sample capacitance structure and the sample dielectric constant. The relationship between the vibration phase of probe and voltage in EFM mode can be expressed as formula (4):

$$\tan\varphi \propto A\left(V_{tip} - V_S\right)^2, A = -\frac{Q}{K}\frac{1}{2}\frac{\partial^2 C}{\partial Z^2} \quad (4)$$

It means that there is a quadratic function relationship between tanφ and the electric potential difference (V_{tip}-V_S), in which the factor A is the coefficient of the quadratic term. The factor A is related to the capacitance between the probe and the sample, according to the formula (4). According to the results of various probe-sample capacitance models[9, 10], the probe-sample capacitance increases with the increase of the dielectric constant ε. Therefore, the absolute value of the factor A has a positive relationship with the dielectric constant ε of the sample. With the remaining parameters basically fixed, the local dielectric properties can be obtained by comparing the slope of the phase change by regulating the bias voltage applied to the probe.

Therefore, EFM can be used to detect the changes in local dielectric properties of PIs in this study. For example, the phase evolution ~ φ of the running-in PI under different tip voltages compared with the virginal PI is shown in Supplementary Fig. S31. The degree of phase change increases with the increase of the tip voltage, in which the phase change of the running-in PI occurs to a greater extent than that of the virgin PI. Furthermore, the surface potential of the PI sample is obtained through the KPFM analysis (Fig. 5b-c). According to the formula (4), the factor A can be thus obtained through establishing the quadratic function relationship between the tan φ and the

electric potential difference (*Vtip*-*Vs*) shown in Supplementary Fig. S32. Accordingly, the running-in PI has a larger absolute value of the factor *A* (~0.00112) than that of the virginal PI (~0.00101), so the local dielectric property of the PI after running-in is enhanced. The analysis of the dielectric property is based on a qualitative method, and more quantitative analysis will be carried out in a future study.

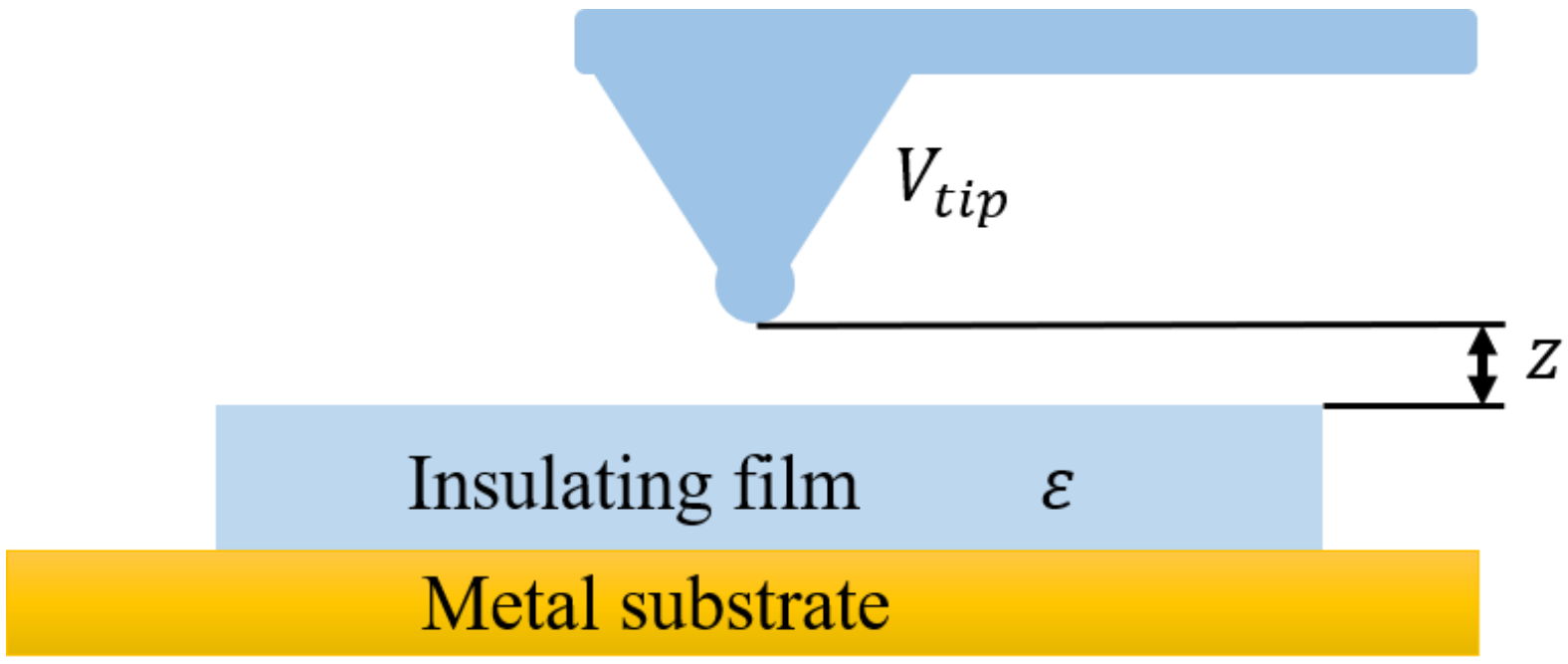

Figure R1 Schematic diagram of dielectric analysis EFM detection.

In addition, the change in the device operation, i.e., sliding frequency, can directly affect the electric output of TENGs according to the second part of the equation from the first principle theory[3]. As shown in Supplementary Fig. S43a, with the increase of sliding frequency, the output current increases obviously, which means the value of the output current is proportional to the frequency, aligning with previous works[12, 13]. The electric interface between the Al tribolayer and the PI surface will be further separated by the lubricant because of the hydrodynamic effect in terms of liquid lubrication theory[14]. Thus, the output charge decreases slightly at a high sliding frequency (Supplementary Fig. S43b).

Changes as follows:

Page 17

Accordingly, the surface potential of the PI surface using Kelvin Probe Force Microscopy (KPFM) is carried out (Fig. 5b-d), and it is obtained that the surface potential changes obviously after the running-in process. From the extracted pixel distribution (Fig. 5b) with respect to the surface potential value, the change occurs after

the first cycle, and there is a significant change in the surface potential after running-in for 100 cycles (Fig. 5c). It is evident from the KPFM results that the proposed running-in process leads to a much lower surface potential, which is correlated with a higher output in the TENG. A lower surface potential, implying a more negative potential, indicates a stronger electron-withdrawing ability[15, 16]. This observation aligns with our findings that the ultrahigh charge density is able to be achieved with one running-in cycle (Fig. 5d). After 100 cycles of running-in, the triboelectric polarity of the PI changes to much more negative (surface potential ~ -6.98 V). It is because the scission and rearrangement of molecular chains occur quickly through the running-in optimization. Accordingly, the surface potential via KPFM can successfully identify the change in the chemical structure of PI surfaces.

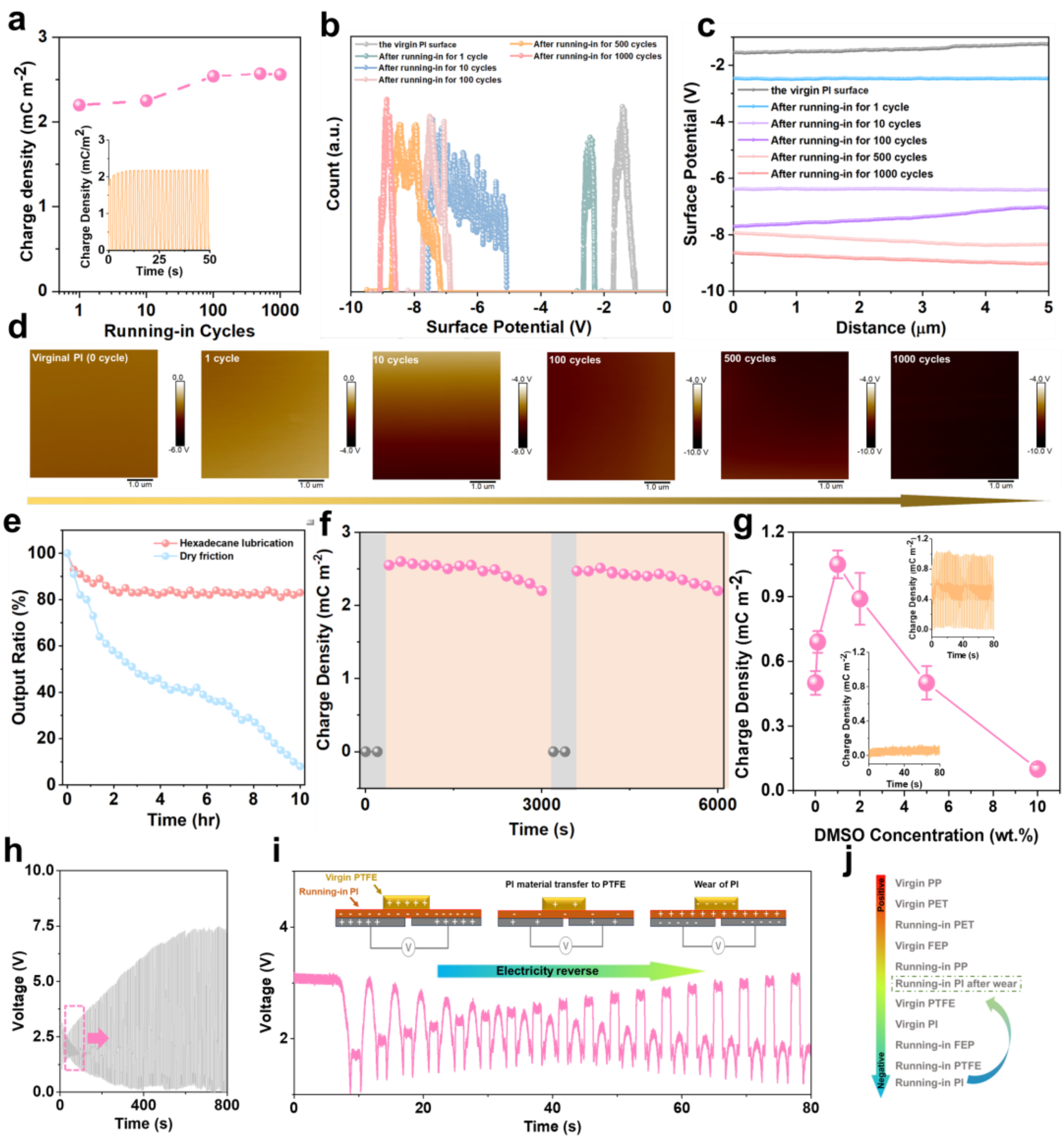

Fig. 5 Super-fast DMSO-assisted running-in strategies towards triboelectric enhancement and reversal effect. a Relationship between the running-in cycle and the triboelectric output. **b** Relative pixel distribution with respect to the surface potential value after running-in with DMSO. **c** Extracted line scan profile with respect to the surface potential value after running-in with DMSO. **d** Surface potential profile of the PI surfaces after running-in with DMSO. **e** Electric outputs of the hexadecane-lubricated TENG after running-in with DMSO compared with the dry-frictional TENG in the long-term test. **f** Charge density of the running-in PI with hexadecane lubrication after intermittent running-in processes. **g** Charge density of the virgin PI lubricated by the mixture of hexadecane and DMSO. **h** and **i** Triboelectric output of the friction pairs between the running-in PI with virgin PTFE, the inset in Figure 5i demonstrating the electric reversal mechanism of the friction pairs. **j** Triboelectric series of engineering dielectric materials for lubricated sliding modes considering the running-in optimization.

Page 18

In addition, the dielectric analysis of the electric materials is also a good way to explain triboelectrification. Scanning force microscopy based on capacitance and electrostatic force is used for measuring the dielectric properties of insulating films at the nanoscale[5-8]. The detection principle involves defining the phase change of the probe under actual vibration state, in contrast to the free vibration phase in Electric Force Microscopy (EFM)[9, 10]. The relationship between the phase of probe vibration and voltage in EFM mode can be expressed as Eq. 4 (more details in **Methods**):

$$\tan\varphi \propto A\left(V_{tip}-V_S\right)^2,\ A=-\frac{Q}{K}\frac{1}{2}\frac{\partial^2 C}{\partial Z^2} \quad (4)$$

where φ is the phase of probe vibration, V_{tip} is the probe voltage, V_S is the surface potential of the sample, Z is a lifting height, the distance between the probe and the sample, C is the system capacitance composed of the probe and the sample. It means that there is a quadratic function relationship between tan φ and the electric potential difference (V_{tip}-V_S), in which the factor A is the coefficient of the quadratic term. The

factor *A* is related to the capacitance between the probe and the sample according to the Eq. 4. According to the results of various probe-sample capacitance models[9, 10], the probe-sample capacitance increases with the increase of the dielectric constant *ε*, so the absolute value of the factor *A* has a positive relationship with the dielectric constant *ε* of the sample. Therefore, EFM can be used to detect the changes in local dielectric properties of PIs. For example, the phase evolution ~ *φ* of the running-in PI under different tip voltages compared with the virginal PI is shown in Supplementary Fig. S31. Through establishing the quadratic function relationship between the tanφ and the electric potential difference (*Vtip*-*Vs*) (Supplementary Fig. S32), the running-in PI has a larger absolute value of the factor *A* (~0.00112) than that of the virginal PI (~0.00101), so the local dielectric property of the PI is enhanced after running-in process.

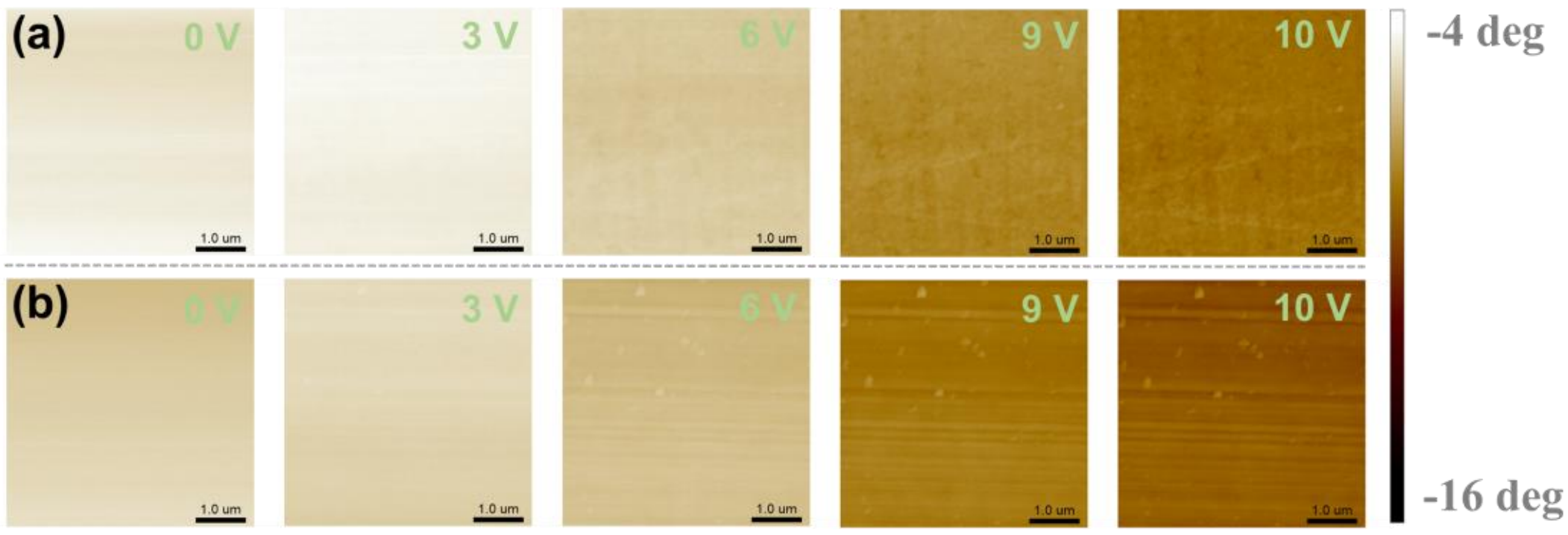

Figure S31 Electrical phase images via EFM for PIs after running-in for 0 cycle (a) and 100 cycles (b).

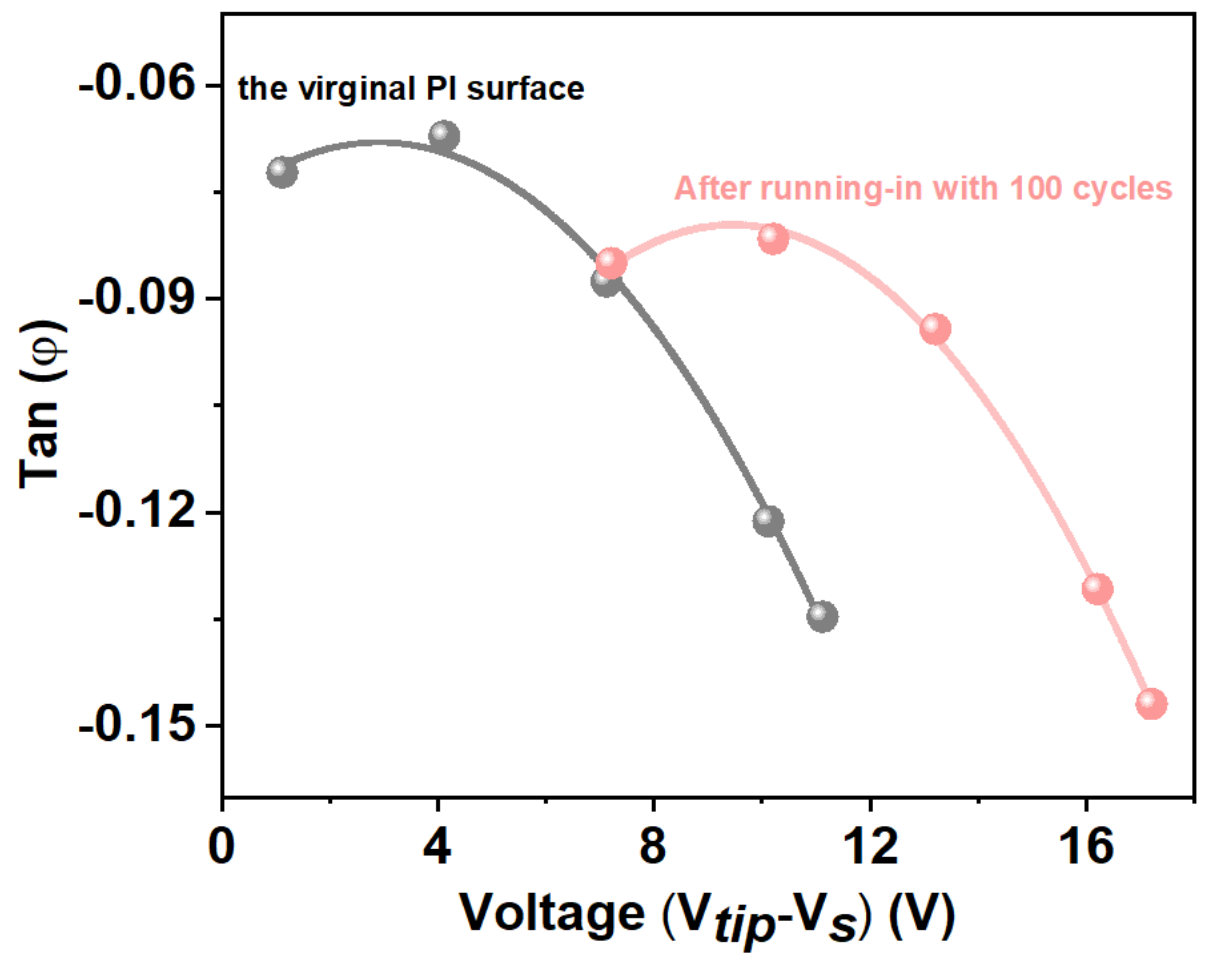

Figure S32 Characteristic curve of the tan *φ* of the PI as a function of the voltage between the tip and the sample surface (*Vtip*-*Vs*).

On the other hand, the change in the device operation, i.e., sliding frequency, can directly affect the electric output of TENGs according to the second part of the equation from the first principle theory[3]. With the increase of sliding frequency, the output current increases obviously (Supplementary Fig. S43a), meaning the value of output current is proportional to the frequency, which aligns with previous works[12, 13]. However, the electric interface between the Al tribolayer and the PI surface will be further separated by the lubricant because of the hydrodynamic effect according to liquid lubrication theory[14]. Thus, the output charge decreases slightly at a high sliding frequency (Supplementary Fig. S43b).

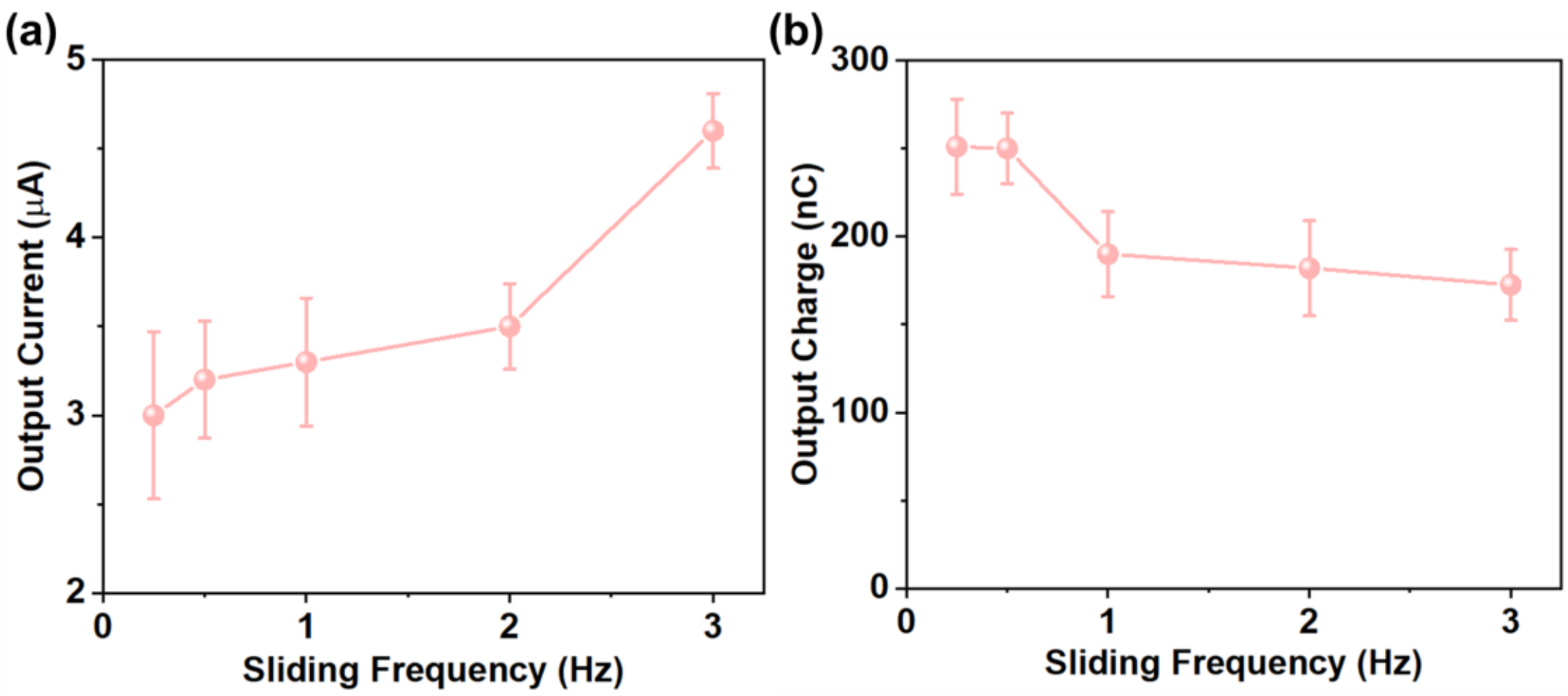

Figure S43 Output current (a) and charge (b) of the TENG lubricated by hexadecane after running-in with DMSO as a function of sliding frequency.

3. In line no. 271 to 278, the authors explained the Mulliken charge of analysis of the carbon, hydrogen, oxygen and nitrogen of the different bindings (-CO-N-C, -CHO, -COOH, -NH-C). The authors claimed that the high negative Mulliken charge means the electron acceptance behaviour. These changes in charge are due to the change of binding atom to the oxygen, carbon and nitrogen. The electron withdrawal from an external surface is different from this one. To understand the electron-withdrawing behaviour of the active layer, the author should model another tribolayer and study interaction with the different models (includes the bindings -CO-N-C, -CHO, -COOH,

-NH-C) by analysing the Mulliken charge before and after interaction. Rather than atomic charge alteration, the authors should focus on the whole binding (-CO-N-C, -CHO, -COOH, -NH-C).

3. Response: Thanks so much for your valuable comments. The whole binding of electric groups should be focused on, as suggested, rather than atomic charge alteration. It is believed that complex physical and chemical reactions occur during the running-in process with DMSO. The C-N bonds in the PI molecule chains are mainly broken around both carbonyl groups. Obtained dangling bonds thus react with H_2O and O_2 so that some tribo-chemical products are achieved, such as the chain bindings (-CO-NH-, -CHO, -COOH, -NH-C). It is known that the C=O bonds in the imide group have strong electron-withdrawing capability, of which the electron-gain abilities are even stronger than the tribo-materials with strong electronegativity atoms (F, Cl, etc.[17, 18]). It is found that the binding of -CO-NH- has much higher electron affinity, which is confirmed by the highly negative Mulliken charge (Supplementary Fig. S27). The positive Mulliken charge of the -COOH binding is probably because the acceptance abilities of the binding could be shielded by the outward-facing hydrogen atom.

Changes as follows:

Page 11

Accordingly, complex physical and chemical reactions occur during the running-in process with DMSO. The C-N bonds in the molecular chains are mainly broken around carbonyl groups and benzenes. Obtained dangling bonds thus react with H_2O and O_2 so that some tribo-chemical products are achieved, such as the chain bindings (-CO-NH-, -CHO, -COOH, -NH-C). It is known that the C=O bonds in the imide group have strong electron-withdrawing capability, of which the electron-gain abilities are even stronger than the tribo-materials with strong electronegativity atoms (F, Cl, etc.[17, 18]). It is found that the binding of -CO-NH- has much higher electron affinity than others, which is confirmed by the highly negative Mulliken charge (Supplementary Fig. S27).

The positive Mulliken charge of the -COOH binding is probably because the acceptance abilities of the binding could be shielded by the outward-facing hydrogen atom.

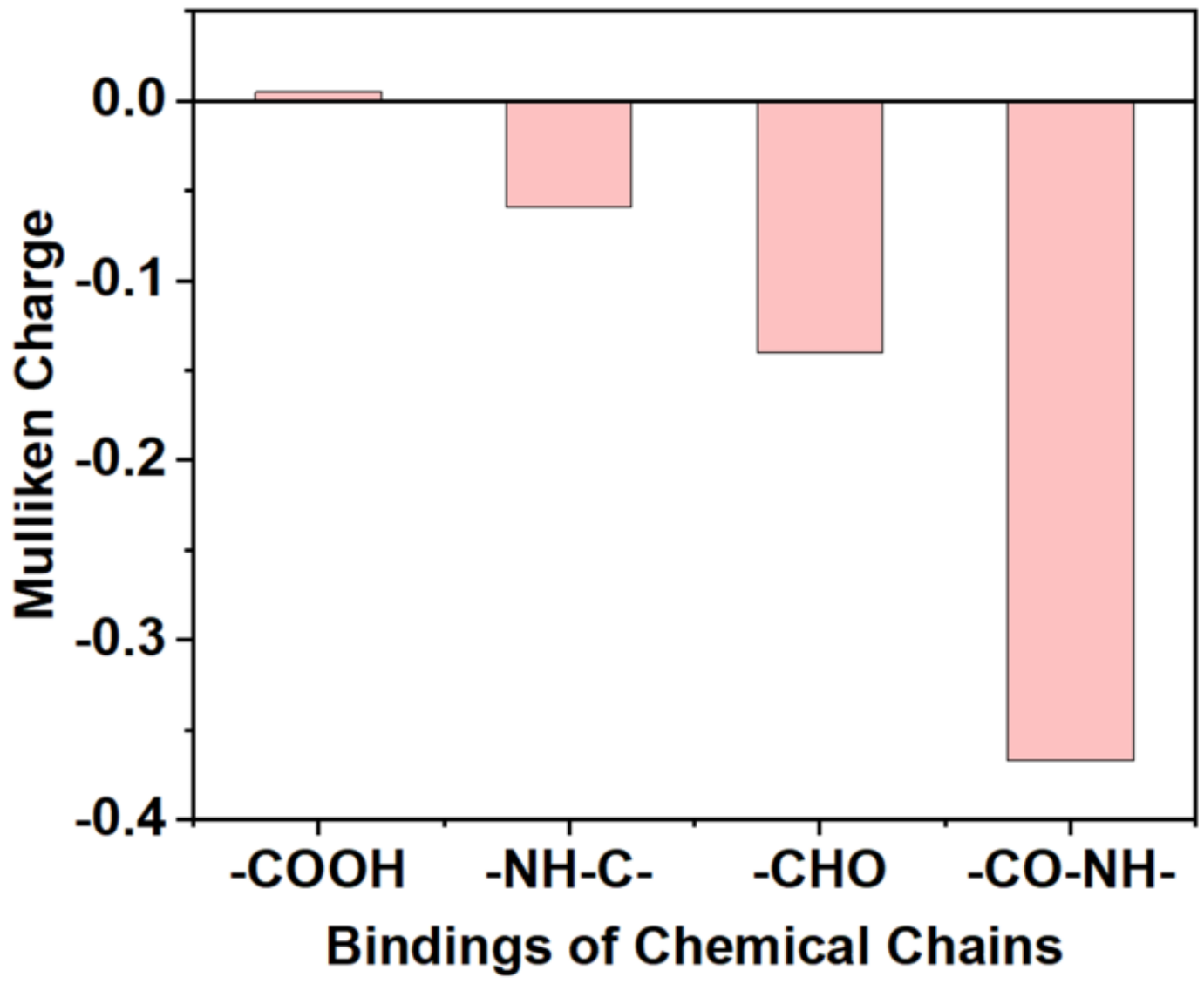

Figure S27 Mulliken charge of the chain scissions in PI induced by running-in process.

4. In Figure 5b, the authors reported the longer cycle run of the device. After the 1800s, there was a gradual decrease in the device's performance. Why decrement in the output observed, the authors should explain in detail. It shows the poor stability of the device, which is usually for the TENG stability.

4. Response: Thanks so much for your valuable comments. The long-term stability of TENGs is very important for practical applications. The high electric output of the TENG is attributed to the regulation of amide-containing chemical chains into "molecular brushes" that extend toward the contact surfaces. These outspread groups could be worn during triboelectrification, and the worn fragments are dissolved in the lubricants. The electric output can keep a high and stable state when the proper lubricants are added continuously[1]. The long-term durability of the TENG was examined in the revised Manuscript. After more than 10 hours (~18 000 cycles), the lubricated TENG exhibits superior electric durability, and maintains 86% output (>2 mC m^{-2}), which is much higher than previous works (Fig. 2d). In contrast, the same TENG device retains only 9% electric output under dry friction in air (Fig. 5e). In

addition, the coefficient of friction of the dry-frictional TENG is two times as much as that of the lubricated TENG (Supplementary Fig. S44a). The PI surface suffers severe wear after dry friction in contrast to the PI surface with the hexadecane lubrication (Supplementary Fig. S44b). This indicates that the DMSO-assisted running-in process has great advantages for boosting the triboelectric output and durability of TENGs. Thus, the TENG with the hexadecane lubrication has good durability.

Changes as follow:

Page 20

The long-term stability of TENGs is very important for the practical application, which is examined as shown in Fig. 5e. After more than 10 hours (~18 000 cycles), the lubricated TENG exhibits superior electric durability and maintains 86% output (>2 mC m^{-2}) which is still much higher than previous works (Fig. 2d). Although there is a little attenuation on the electric output, the charge density of the lubricated TENG significantly increases within a fast running-in process of 100 cycles (Fig. 5f). The high electric output of the TENG is attributed to the regulation of amide-containing chemical chains into "molecular brushes" that extend toward the contact surfaces. These outspread groups could be worn during triboelectrification, probably resulting in a decrease in the electric output. In contrast, the same TENG device retains only 9% electric output under dry friction in air. In addition, the coefficient of friction of the dry-frictional TENG is two times as much as that of the lubricated TENG (Supplementary Fig. S44a). The PI surface suffers severe wear after dry friction in contrast to the PI surface with the hexadecane lubrication (Supplementary Fig. S44b). Thus, the TENG with the hexadecane lubrication has good electric durability and tribological performance.

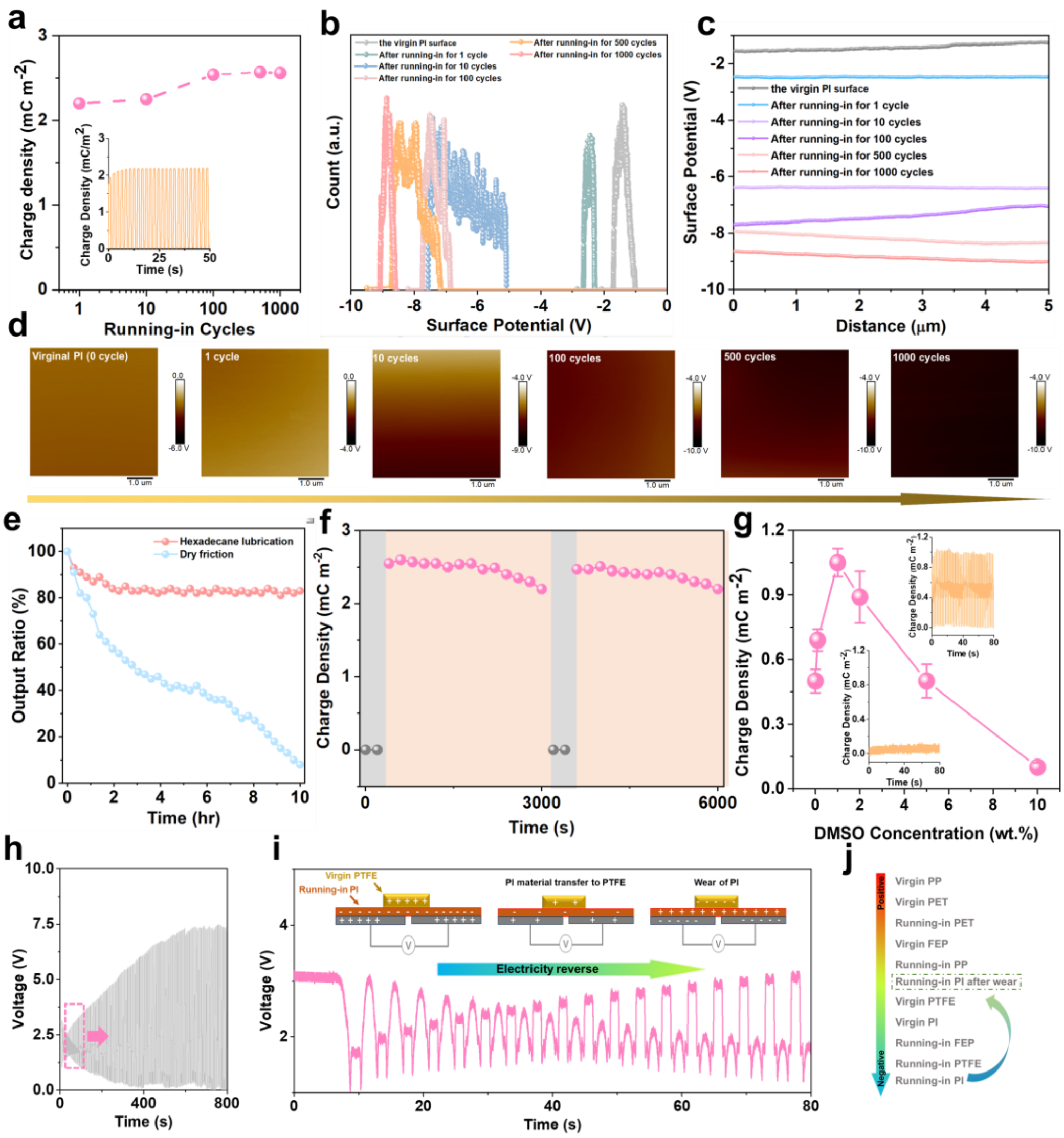

Fig. 5 Super-fast DMSO-assisted running-in strategies towards triboelectric enhancement and reversal effect. a Relationship between the running-in cycle and the triboelectric output. **b** Relative pixel distribution with respect to the surface potential value after running-in with DMSO. **c** Extracted line scan profile with respect to the surface potential value after running-in with DMSO. **d** Surface potential profile of the PI surfaces after running-in with DMSO. **e** Electric outputs of the hexadecane-lubricated TENG after running-in with DMSO compared with the dry-frictional TENG in the long-term test. **f** Charge density of the running-in PI with hexadecane lubrication after intermittent running-in processes. **g** Charge density of the virginal PI lubricated by the mixture of hexadecane and DMSO. **h** and **i** Triboelectric output of the friction

pairs between the running-in PI with virginal PTFE, the inset in Fig. 5i demonstrating the electric reversal mechanism of the friction pairs. **j** Triboelectric series of engineering dielectric materials for lubricated sliding modes considering the running-in optimization.

5. In supplementary figure S21, the authors reported the peak power density analysis. It shows that peak power density is obtained at 5Gohm. It shows the high impedance of the device. Why is it high, and how to control the phenomenon with the proposed method? And the authors have used the unit W m^{-2} Hz^{-1}, which formula authors have used to calculate the power and power density. The authors have provided the charge density analysis of the TENG by optimising the running-in process. The authors should add the output performance in terms of voltage and current in the same way.

5. Response: Thanks so much for your valuable comments. The main reasons for high internal resistance are related to the dielectric properties of the triboelectric materials and the capacitance limitations of the device. The impedance of the TENG (Z) is expressed as $Z = \frac{1}{2\pi f C_s}$. Among them, Cs and f are the inherent capacitance and operation frequency of TENGs, respectively. The TENG commonly uses typical dielectric PIs with relatively low dielectric constants. The PI-based TENGs have a very low capacitance, ~ Cs, which ultimately leads to the extremely high internal resistance of the TENG[19]. Further, the internal resistance of the lubricated TENGs is higher than that of the dry-frictional TENG, which is because the hexadecane lubricant is nonpolar and insulating Thus, it shows a high impedance of the device and is similar to others' work[20].

It is important to address the issue of high impedance of the TENG. Various approaches have been explored to reduce the impedance of the TENGs, e.g., using external power management circuits. For example, optimizing inherent features by coupling TENGs to a capacitor auto-conversion circuit can effectively reduce external load impedance[21]. This study mainly works on optimizing the running-in process to

boost the charge density of TENGs. It is of course, a valuable idea to address the issue via power management circuits, which will be further studied in future studies.

The formula of peak output power used in this study is $P_{max} = I_{max}^2 R$ [22], where I_{max} represents the peak current, and R is the resistance. The unit W m^{-2} Hz^{-1} represents the normalized peak power density as an essential metric, i.e., $P_{norm} = \frac{P_{max}}{A \cdot f}$, in which A and f represent the contact area and frequency, respectively. These formulas and the unit of W m^{-2} Hz^{-1} are used in recent works[13, 22-24]. The related references are cited in the revised manuscript.

The output voltage and current of the TENGs are also added in the revised Supplementary Information. It is found that the output voltage and current of the TENG lubricated by hexadecane after running-in with DMSO are about 680 V and 3.3 μA, respectively (Supplementary Fig. S2). In addition, the PI-based TENG has much higher voltage and current than those without running-in treatment (Supplementary Fig. S2c-d). The influence of the dissolvents on output voltage and current is also similar to the trends of the charge density (Supplementary Fig. S2e-f).

Changes as follow:

Page 5

In addition, the PI-based TENG has much higher voltage and current than those without running-in treatment (Supplementary Fig. S2). The output voltage and current of the TENG lubricated by hexadecane after running-in with DMSO are about 680 V and 3.3 μA, respectively. The influence of lubricants during the triboelectric period is shown in Fig. 2b and Supplementary Fig. S2c-d.

Page 6

It is found that dissolvents, such as DMSO, DMA, DMF, etc., during the running-in process can boost the electric output of PI (Fig. 2c and Supplementary Fig. S2e-f)

Further, the peak current density and power of the TENG were measured with different resistances (Supplementary Fig. S9 and Fig. S10). According to the normalized peak power density as an essential metric[13, 22-24], the peak output power density of the TENG reaches a maximum of 18.5 W m^{-2} Hz^{-1} at an external circuit load of 5 GΩ, which is also higher than other works (Supplementary Fig. S11), representing the strong advantage of the running-in optimization strategy in achieving high-performance TENGs. In contrast, the dry-frictional TENG as the standard sample has the lowest power density of 0.2 W m^{-2} Hz^{-1} at the external circuit load of 2 GΩ (Supplementary Fig. S10). It is found that the internal resistance of the TENG is high, which is similar to other work[20]. The main reason is that PI-based TENGs have a very low capacitance, which ultimately leads to a high internal resistance of the TENG[19]. Further, the internal resistance of the lubricated TENG is higher than that the dry-frictional TENG because the hexadecane lubricant is nonpolar and insulating[1].

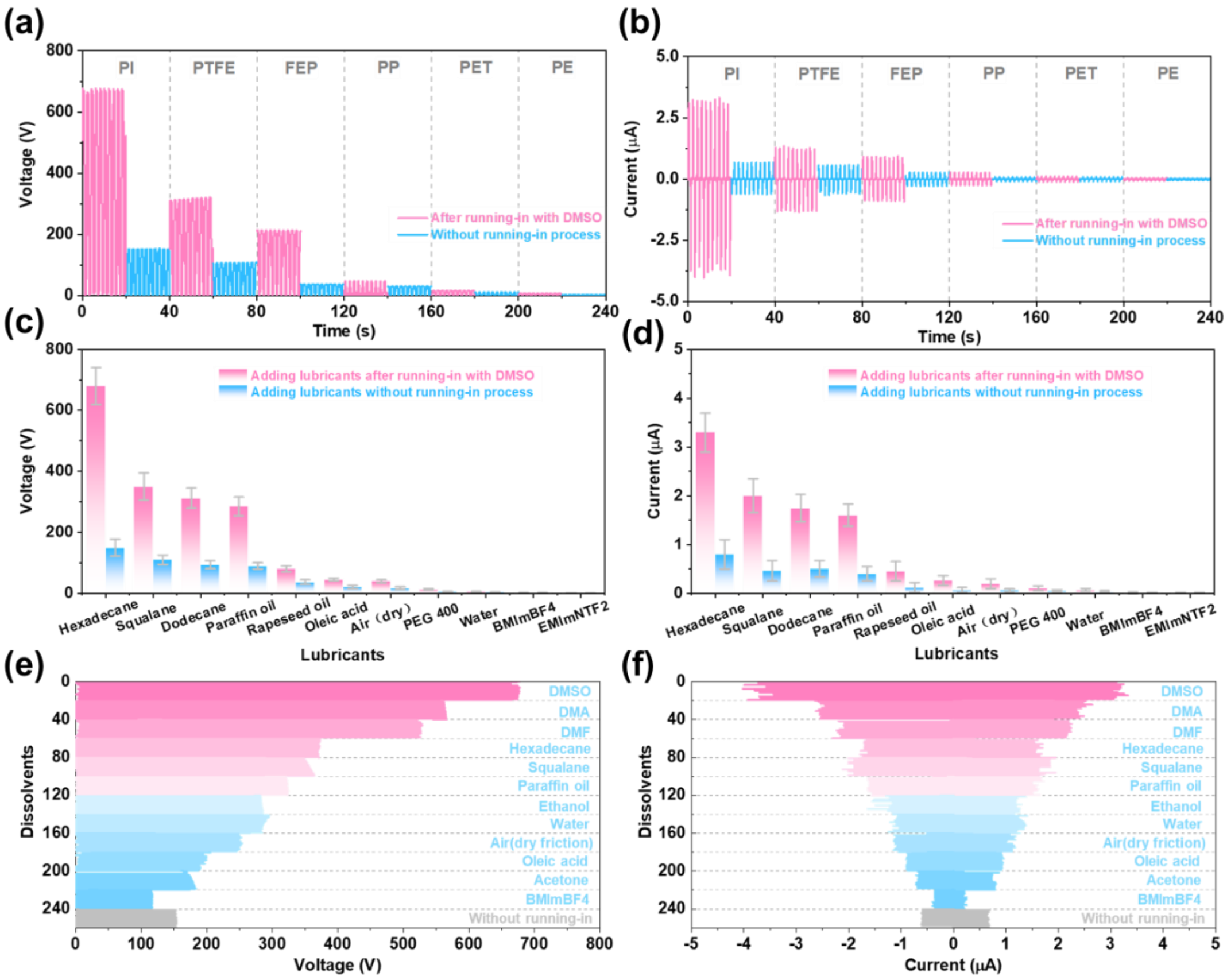

Figure S2 Output voltage (a) and current (b) of different engineering tribo-materials lubricated by hexadecane after running-in with DMSO. Output voltage (c) and current (d) of the engineering PI lubricated by different lubricants after running-in with DMSO. Output voltage (e) and current (f) of the engineering PI lubricated by hexadecane after running-in with different dissolvents.

References

1. Wu, J., Xi, Y.H. & Shi, Y.J. Toward wear-resistive, highly durable and high performance triboelectric nanogenerator through interface liquid lubrication. *Nano Energy* **72**, 9 (2020).
2. Chung, S.H. et al. Nonpolar Liquid Lubricant Submerged Triboelectric Nanogenerator for Current Amplification via Direct Electron Flow. *Adv. Energy Mater.* **11**, 8 (2021).
3. Wang, Z.L. On the first principle theory of nanogenerators from Maxwell's equations. *Nano Energy* **68**, 10 (2020).
4. Busolo, T. et al. Surface potential tailoring of PMMA fibers by electrospinning for enhanced triboelectric performance. *Nano Energy* **57**, 500-506 (2019).
5. Fumagalli, L., Gramse, G., Esteban-Ferrer, D., Edwards, M.A. & Gomila, G. Quantifying the dielectric constant of thick insulators using electrostatic force microscopy. *Appl. Phys. Lett.* **96**, 3 (2010).
6. Fumagalli, L., Ferrari, G., Sampietro, M. & Gomila, G. Quantitative Nanoscale Dielectric Microscopy of Single-Layer Supported Biomembranes. *Nano Lett.* **9**, 1604-1608 (2009).
7. Fumagalli, L., Ferrari, G., Sampietro, M. & Gomila, G. Dielectric-constant measurement of thin insulating films at low frequency by nanoscale capacitance microscopy. *Appl. Phys. Lett.* **91**, 3 (2007).
8. Houssat, M., Villeneuve-Faure, C., Dignat, N.L. & Cambronne, J.P. Nanoscale mechanical and electrical characterization of the interphase in polyimide/silicon nitride nanocomposites. *Nanotechnology* **32**, 13 (2021).

9. Qi, G.C. et al. Quantifying Surface Charge Density by Using an Electric Force Microscope with a Referential Structure. *J. Phys. Chem. C* **113**, 204-207 (2009).
10. Qi, G.C. et al. Characteristic capacitance in an electric force microscope determined by using sample surface bias effect. *J. Appl. Phys.* **103**, 4 (2008).
11. Li, D.A., Wang, X., Mo, X.Y., Tse, E.C.M. & Cui, X.D. Electronic gap characterization at mesoscopic scale via scanning probe microscopy under ambient conditions. *Nat. Commun.* **13**, 7 (2022).
12. Zi, Y.L. et al. Harvesting Low-Frequency (<5 Hz) Irregular Mechanical Energy: A Possible Killer Application of Triboelectric Nanogenerator. *Acs Nano* **10**, 4797-4805 (2016).
13. He, W.C. et al. Boosting output performance of sliding mode triboelectric nanogenerator by charge space-accumulation effect. *Nat. Commun.* **11**, 8 (2020).
14. Gohar, R. & Cameron, A. Optical Measurement of Oil Film Thickness under Elasto-Hydrodynamic Lubrication. *Nature* **200**, 458-& (1963).
15. Li, Y. et al. Advanced Dielectric Materials for Triboelectric Nanogenerators: Principles, Methods, and Applications. *Adv. Mater.* **36**, 53 (2024).
16. Zhao, K. et al. Layer-Engineered Hybrid MoS2 Nanosheets for Synergistic Charge Trapping and Superlubrication toward High-Output Ultra-Durable Triboelectric Nanogenerators. *Adv. Funct. Mater.*, 16 (2025).
17. Wu, J., Wang, X.L., Li, H.Q., Wang, F. & Hu, Y.Q. First-principles investigations on the contact electrification mechanism between metal and amorphous polymers for triboelectric nanogenerators. *Nano Energy* **63**, 7 (2019).
18. Zou, H.Y. et al. Quantifying the triboelectric series. *Nat. Commun.* **10**, 9 (2019).
19. Wang, H.L., Guo, Z.H., Zhu, G., Pu, X. & Wang, Z.L. Boosting the Power and Lowering the Impedance of Triboelectric Nanogenerators through Manipulating the Permittivity for Wearable Energy Harvesting. *Acs Nano* **15**, 7513-7521 (2021).
20. Yang, P. et al. Radical anion transfer during contact electrification and its compensation for charge loss in triboelectric nanogenerator. *Matter* **6**, 18 (2023).

21. Ren, D.H. et al. Ultra-High DC and Low Impedance Output for Free-Standing Triboelectric Nanogenerator. *Adv. Energy Mater.* **13**, 10 (2023).
22. Niu, S.M. et al. Theoretical study of contact-mode triboelectric nanogenerators as an effective power source. *Energy Environ. Sci.* **6**, 3576-3583 (2013).
23. Luo, Y.J. et al. Advanced energy harvesting from low-frequency ocean waves for lithium-ion battery applications. *Energy Environ. Sci.* **18**, 4821-4832 (2025).
24. Gao, Y.K. et al. Spontaneously established reverse electric field to enhance the performance of triboelectric nanogenerators via improving Coulombic efficiency. *Nat. Commun.* **15**, 11 (2024).

Reviewer: 3

The Authors present a work describing novel strategies to reach high performances in polyimide (PI)/aluminum triboelectric nanogenerators (TENGs), focusing in particular to the "running-in" pre-processing of the device. Treatment with DMSO polar, aprotic, solvent at the interface allowed the removal of the species formed due to friction which could interfere with charge separation. Moreover, both an increase of the effective interaction area, as well as C-N bonds breakage on the PI surface and exposure of electron-withdrawing groups, contribute to obtain a remarkably high charge density at the interface, if compared with current state-of-the-art of TENGs devices.

The results are indeed interesting, also for their technological implications. Moreover, the suggested mechanism is convincingly supported by experimental evidence (SEM analysis of the surfaces, XPS and mass spectrometry characterization of surface groups) and computational simulations (molecular dynamics of PI polymer with DMSO, electronic structure calculations of cleaved PI on Al surface). I have only minor questions before recommending for publication in Nature Communications:

1. Authors state that the obtained charge density is higher than the "intrinsic charge density of PI" (ref. 17). Can authors describe more in detail what the "intrinsic charge density" refers to?

1. Response: Thanks so much for your valuable comments. In most practical situations, TENGs operate in the air atmosphere, where the maximum triboelectric charge density has been restricted because of the air breakdown effect[1, 2]. Accordingly, it is worth noting that vacuum triboelectric charge density has the advantages: (1) as a reference for determining the presence of air breakdown in an atmosphere condition; (2) revealing the relationship between triboelectric charge density and material's intrinsic properties[3]. Thus, the intrinsic charge density represents the maximum triboelectric charge of the TENG operating in a vacuum environment[1, 3].

Changes as follows:

Page 4

It is reported that, due to the limitation of triboelectric properties, the charge density can only be realized to 1.67 mC m^{-2} on the engineering tribo-materials. Thus, in this study, a new record charge density is successfully achieved.

Page 7

Remarkably, the running-in optimization method developed in this study delivers a record-high charge density of 2.5 mC m^{-2}, exceeding the previous maximum achieved by the space-accumulation effect[5] by a factor of 1.53. The previous space-accumulation work also breaks the limit of the intrinsic charge density that defines the maximum charge density of triboelectric materials under vacuum conditions[1, 3].

2. Please make the EDS acronym explicit when first used in the text.

2. Response: Thanks so much for your valuable suggestion. The EDS acronym, explicit, i.e., Energy Dispersive Spectrometer, when first used, has been added in the revised manuscript.

Changes as follows:

Page 8

According to the Energy Dispersive Spectrometer (EDS) analysis, it also finds that many abrasive grains are transferred from the Al tribolayer to the dry-frictional PI surface (Supplementary Fig. S15).

3. The S-N RDF is reported in Fig. 4d (and not Fig. 3d, as stated in the text), while S-O RDF is in Fig. 4c (and not Fig. 4d).

3. Response: Thanks so much for your valuable suggestions. We are very sorry for these errors. Fig. 4c and Fig. 4d have been swapped in position for each other. That has been corrected in the revised manuscript.

Changes as follows:

Page 12

Furthermore, the variations of the radius distribution function (RDF) are introduced here to identify the interaction between DMSO and PI, shown in Fig. 4c-d. The value of RDF between the S atoms in DMSO and the N atoms in the running-in PI increases gradually at a distance ranging from 3.3 Å to 9.9 Å with the time relaxation (Fig. 4c), in which the peaks of the RDF values are mainly distributed around 6 Å. It means the DMSO molecule effectively infiltrates into the molecular chains of PI, which is also confirmed by the result of RDF between the S atoms in DMSO and the O atoms in the running-in PI shown in Fig. 4d.

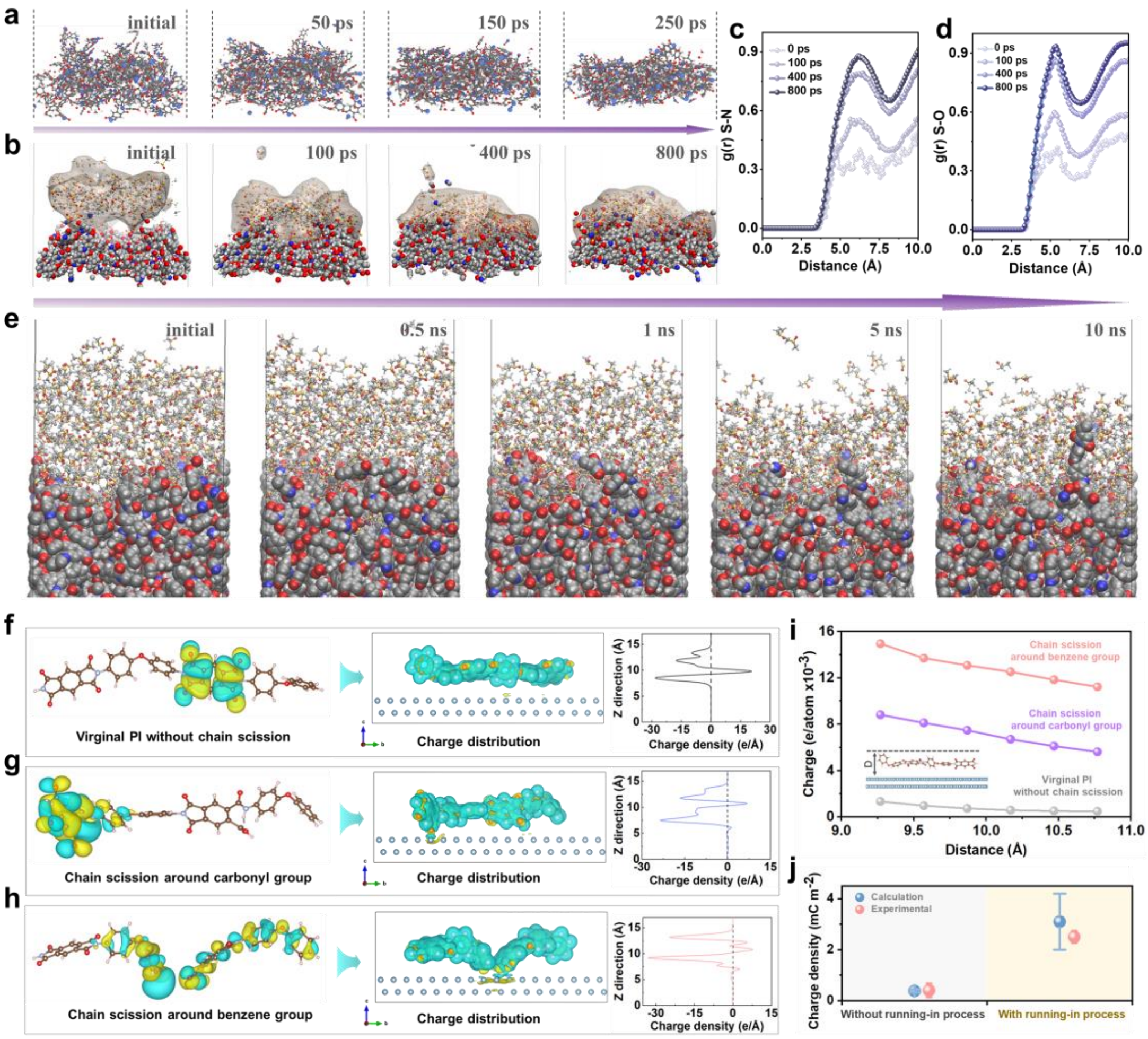

Fig. 4 The triboelectric mechanism of PI with DMSO-assisted running-in process achieved via MD and DFT simulation. a Relaxation process of the PI bulk phase after friction with Al performed by classical MD. **b** Time-dependent dynamics of DMSO molecules infiltrating into the running-in PI. **c** and **d** Radial distribution function between the S atom of DMSO and the N, O atom in the running-in PI. **e** Time-dependent MD analysis of the extraction effect of DMSO molecules on the broken chemical chains, e.g., amide groups, on the surface of the running-in PI. **f**, **g**, and **h** Lowest unoccupied molecular orbital (LUMO), charge distribution, and 2D charge density difference of PIs. **i** Average number of electrons obtained by each atom of PIs. Inset demonstrating the distance between the top of the molecule and the contact interface. **j** Charge densities from the calculations according to Figure 4i and from the experimental through lubrication without running-in process or lubrication after running-in with DMSO according to Figure 2b.

4. In the PI-Al surface modelling through DFT calculations, are two Al layers (as shown in Figure 4 and S17) sufficient? Has the inner surface of the slab been passivated (for instance with (pseudo)hydrogens)?

4. Response: Thanks so much for the valuable comment. Surface calculations were performed using slab models. The Al (111) surface was modeled by cleaving a 2-layer slab from the bulk FCC aluminum (a = 4.038 Å). A vacuum spacing of 15 Å was introduced normal to the surface to decouple periodic slabs. During geometry optimization, the entire Al (111) slab was held fixed to simulate a rigid surface, and only the adsorbed molecule was allowed to relax. Two layers are sufficient, as both layers are fixed in calculation, so the number of layers has no impact on DFT calculations.

In the current preliminary calculations, we have simplified the model by not explicitly passivating the aluminum surface, which is based on the core scientific objective of this stage of research: to clarify the charge transfer mechanism between specific functional groups of molecular chains and the pure aluminum surface at the atomic scale, at the moment of bond breaking. Our calculations aim to reveal the intrinsic electronic behavior of this initial reaction interface without uncertainties, e.g., a passivation layer in structure, thickness, and defect density. The complex electronic states of the passivation layer itself will strongly couple with the states of PI, making it difficult for us to clearly distinguish and attribute the source of transferred charges transfer. Incorporation of explicit passivation and/or oxide layers will be considered in future work.

5. In PI models with broken C-N bonds, it seems that C=O (cleavage in the imide moiety) or NH (cleavage of the N-benzene bond) strongly interact (with coordinate covalent bonds) with Al surface atoms (according to Figures 4 and S17). Since C=O groups in unbroken PI could, in principle, also coordinate Al, but this is not observed in the model, could Authors give more details about possible reasons for this different

binding behavior at the interface? Does the higher flexibility of the broken PI contribute to some extent?

5. Response: Thanks so much for the valuable comment. Running-in friction induces the breakage of the relatively unstable N-C bonds in PI, resulting in the freedom of releasing electron-acceptor groups, which means the broken PI has higher flexibility in contrast to the unbroken PI. Together with the function of DMSO-driven extraction, the chemical chains containing amide groups are regulated to be "molecular brushes" outspread on contact surfaces. In addition, there is a significant peak of electron loss at the interface of Al/PI (5Å) for the case of broken PI (Fig. 4h). At the same time, the electron gain in the nearby PI molecules is also obvious, which identifies that there are many charges exchanging between the Al and the broken PI, meaning the strongly interact between them through additional electrostatic interaction. Thus, the distance between the C=O groups and Al surfaces is significantly decreased for the broken PI. In contrast, the peak of electron gain is weaker for the case of the unbroken PI (Fig. 4f).

Changes as follow:

Page 15

For the case of running-in PI with the N-related chain scission around benzene (Fig. 4h), there is a significant peak of electron loss at the interface of Al/PI (5Å). At the same time, the electron gain in the nearby PI molecules is also obvious, which identifies that the electrons are transferred from the Al to the running-in PI.

Page 15

In addition, running-in friction induces the breakage of the relatively unstable N-C bonds in PI, resulting in the freedom of releasing electron-acceptor groups, which means the running-in PI has higher flexibility. Together with the function of DMSO-driven extraction, the chemical chains containing amide groups are regulated to be

"molecular brushes" outspread on contact surfaces. Thus, the distance between the C=O groups and the Al surface is significantly decreased for the broken PI.

6. In the simulation of the friction between PI and Al, Authors say that a "virtual aluminum (Al) substrate" was employed. Can they give more details about this virtual substrate approach?

6. Response: Thanks so for the valuable comment. Surface calculations were performed using slab models. The Al (111) surface was modeled by cleaving a 2-layer slab from the bulk FCC aluminum (a = 4.038 Å). A vacuum spacing of 15 Å was introduced normal to the surface to decouple periodic slabs. During geometry optimization, the entire Al (111) slab was held fixed to simulate a rigid surface, and only the adsorbed molecule was allowed to relax.

Changes as follows:

Page 26

The Al (111) surface is modeled by cleaving a 2-layer slab from the bulk FCC aluminum (a = 4.038 Å). A vacuum spacing of 15 Å is introduced normal to the surface to decouple periodic slabs. During geometry optimization, the entire Al (111) slab is held fixed to simulate a rigid surface, and only the adsorbed molecule was allowed to relax.

7. Has the ReaxFF force field employed in MD simulations to allow bond breakage been specifically re-parametrized for the PI system, or have the default parameters been employed?

7. Response: Thanks so much for the valuable comment. In our reactive molecular dynamics simulations, we employed the standard ReaxFF parameter set containing C, H, O, and N elements-the same parameterization used by Wang et al.[4] in their study of the RDX–Al interface. This well-established parameter set has been widely applied to

organic systems, with the bonding characteristics being similar to the polyimide (PI) system. Although the original ReaxFF parameters were not explicitly parametrized for PI, we ensured the structural stability of the initial model through preliminary classical MD relaxation and energy minimization using the PCFF force field. Moreover, since ReaxFF inherently incorporates bond-order–dependent interactions, it can naturally describe the induced N–C bond scission in PI chains[5-8].

8. Some sentences in the text are slightly unclear: "The dissolvents have the functionalization of carrying worn free radicals and debris…" (function, pg. 3); To further understand how the DMSO-assisted solvent activation…" (pg. 10); "there is a significant peak of electron loss near the side of Al near the interface of Al/PI." (pg. 13); "…the nearby PI molecules gain electrons are also obvious…" (electrons gain, pg. 13); "And, the peak current density and average power of the TENG were measured with different resistances." (pg. 15); "…making friction and wear of tribo-materials reduction." (pg. 16); "The raking of the running-in PI after wear changes behand to virgin PTFE in the triboelectric series…" (ranking, behind, pg. 17).

8. Response: Thanks so much for the valuable and kind comments. These errors have been corrected in the revised manuscript.

"The dissolvents have the functionalization of carrying worn free radicals and debris…" has changed to "The dissolvents have the function of carrying worn free radicals and debris…".

"To further understand how the DMSO-assisted solvent activation…" has changed to "To further understand the DMSO-assisted solvent activation…".

"there is a significant peak of electron loss near the side of Al near the interface of Al/PI." has changed to "there is a significant peak of electron loss at the interface of Al/PI.".

"…the nearby PI molecules gain electrons is also obvious…" has changed to "the electron gain in the nearby PI molecules is also obvious".

“And, the peak current density and average power of the TENG were measured with different resistances.” has changed to “The peak current and peak power density of the TENG are obtained under different external load resistance.”

“because the hexadecane fluid has the capability of separating the two parts of contact surfaces, making friction and wear of tribo-materials reduction.” has changed to “because the hexadecane fluid has the capability of separating the two parts of contact surfaces, making friction and wear reduction”

“The raking of the running-in PI after wear changes behand to virgin PTFE in the triboelectric series…” has changed to “The ranking of the running-in PI after wear changes behind to virgin PTFE in the triboelectric series…”

Changes as follow:

Page 3 and Page 6

The dissolvents have the function of carrying worn free radicals and debris.

Page 12

To further understand the DMSO-assisted solvent activation, the molecular models of the PI sliding against a virtual Al within DMSO were developed by a ReaxFF-based reactive molecular dynamics (MD) simulation and a polymer consistent force field (PCFF)-based classical MD simulation (Methods).

Page 15

For the case of running-in PI with the N-related chain scission around benzene (Fig. 4h), there is a significant peak of electron loss at the interface of Al/PI (5Å). At the same time, the electron gain in the nearby PI molecules is also obvious, which identifies that the electrons are transferred from the Al to the running-in PI.

Page 8

The peak current and peak power density of the TENG are obtained under different external load resistance.

Page 21

In contrast to the dry friction, the COF is much lower for the case of hexadecane lubrication (Supplementary Fig. S14) because the hexadecane fluid has the capability of separating the two parts of the contact surfaces, resulting in friction and wear reduction.

Page 21

The ranking of the running-in PI after wear changes behind virgin PTFE in the triboelectric series shown in Fig. 5j.

References

1. Zhang, C.L. et al. Surface charge density of triboelectric nanogenerators: Theoretical boundary and optimization methodology. *Appl. Mater. Today* **18**, 8 (2020).
2. He, W.C. et al. Boosting output performance of sliding mode triboelectric nanogenerator by charge space-accumulation effect. *Nat. Commun.* **11**, 8 (2020).
3. Liu, D. et al. Standardized measurement of dielectric materials' intrinsic triboelectric charge density through the suppression of air breakdown. *Nat. Commun.* **13**, 10 (2022).
4. Wang, N. et al. Thermodynamic Simulation of the RDX-Aluminum Interface Using ReaxFF Molecular Dynamics. *J. Phys. Chem. C* **121**, 14597-14610 (2017).
5. Chen, M.H. et al. Recent ReaxFF MD studies on pyrolysis and combustion mechanisms of aviation/aerospace fuels and energetic additives. *Energy Adv.* **2**, 54-72 (2023).

6. Chenoweth, K., Cheung, S., van Duin, A.C.T., Goddard, W.A., III & Kober, E.M. Simulations on the thermal decomposition of a poly(dimethylsiloxane) polymer using the ReaxFF reactive force field. *J. Am. Chem. Soc.* **127**, 7192-7202 (2005).
7. Du, Y.Z., Li, J., Wen, N., Zhang, Z. & Song, D. The pyrolysis of polyimide and epoxy resin by the ReaxFF molecular dynamics simulation. *J. Mol. Model.* **31**, 14 (2025).
8. Wang, X.S., Zhao, T., Wang, Y.H., Zhang, L. & Zou, L. Microscopic Pyrolytic and Electric Decomposition Mechanism of Insulating Polyimide/Boron Nitride Nanosheet Composites based on ReaxFF. *Polymers* **14**, 20 (2022).

Reviewer: 4

This manuscript reports an optimization running-in process with various solvents in enhancing triboelectric output. In particular, non-polar solvents like DMSO, DMA and DMF are highly recommended in removing the worn radicals and debris from the running-in process. The proposed idea is interesting, particularly with the use of liquid lubricant enabling the efficient charge transferred in a short time and support by the MD simulation. However, the study requires further detailed analysis and discussion to support the conclusions drawn. Specific comments are as follows:

1. The ratio of the real contact to the nominal contact area using the simulation calculation required more detail information to support the quantitative analysis of contact stress analysis after the running in with hexadecane and DMSO as shown in Figure 3b-e. It is required to align the terminology between the evaluated surface roughness and the simulation calculation for clarity and consistency.

1. Response: Thanks so much for your valuable comments. As shown in Fig. 3b, the ratio of the real contact area to the nominal contact area has a positive relationship with the contact (or wear) depth according to the simulation calculation (details can be found in Materials Part). The contact stress distribution and contact area for the PI surface after running-in with hexadecane and DMSO are added in the revised Supplementary Information. The contact stress and contact area increase gradually with the increase of the depth (Supplementary Fig. S19-20). Especially, the maximum contact stresses of the surfaces after running-in with hexadecane and DMSO are respectively, about 0.98 GPa and 11.11 GPa at the depth of 400 nm (Fig. 3c and Supplementary Fig. S19). In addition, the numbers of red spots demonstrate the contact state of the friction pairs (Fig. 3c-e and Supplementary Fig. S20). In this regard, the ratio value is 11.9% for the case of the PI after running-in with DMSO, whereas after running-in with hexadecane, the ratio value is about 8.9%. It thus means the real contact area size is increased by 33.7%, which is attributed to the lower mechanical properties of the PI after the running-in process. In addition, we carefully align the terminology between the

evaluated surface roughness and the simulation calculation for clarity and consistency.

Changes as follows:

Page 9

Accordingly, the contact stress and contact area increase gradually with the increase of the depth (Fig. 3b and Supplementary Fig. S19-20) (details shown in **Materials** Part). Especially, the maximum contact stresses of the surfaces after running-in with hexadecane and DMSO are respectively, about 0.98 GPa and 11.11 GPa at the depth of 400 nm (Fig. 3c). In addition, the numbers of red spots demonstrate the contact state of the friction pairs (Fig. 3c-e and Supplementary Fig. S20). In this regard, the ratio value is 11.9% for the case of the PI after running-in with DMSO, whereas after running-in with hexadecane, the ratio value is about 8.9%. It thus means the real contact area size is increased by 33.7%, which is attributed to the lower mechanical properties of the PI after the running-in process.

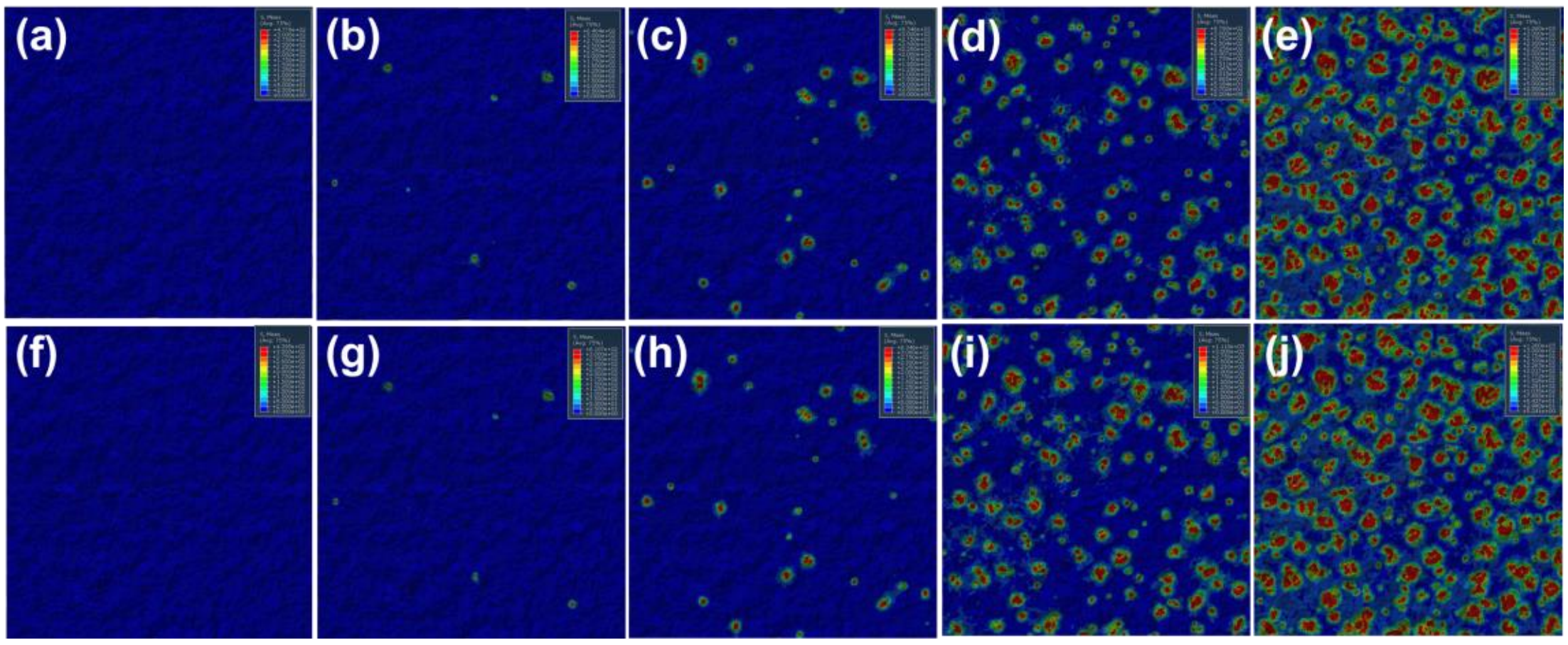

Figure S19 Contact stress distribution of the TENG after running-in with hexadecane under the contact depth of 100 nm (a), 200 (nm) (b), 300 nm (c), 400 nm (d) and 500 nm (e); Contact stress distribution after running-in with DMSO under different contact depth of 100 nm (a), 200 nm (b), 300 nm (c), 400 nm (d), and 500 nm (e).

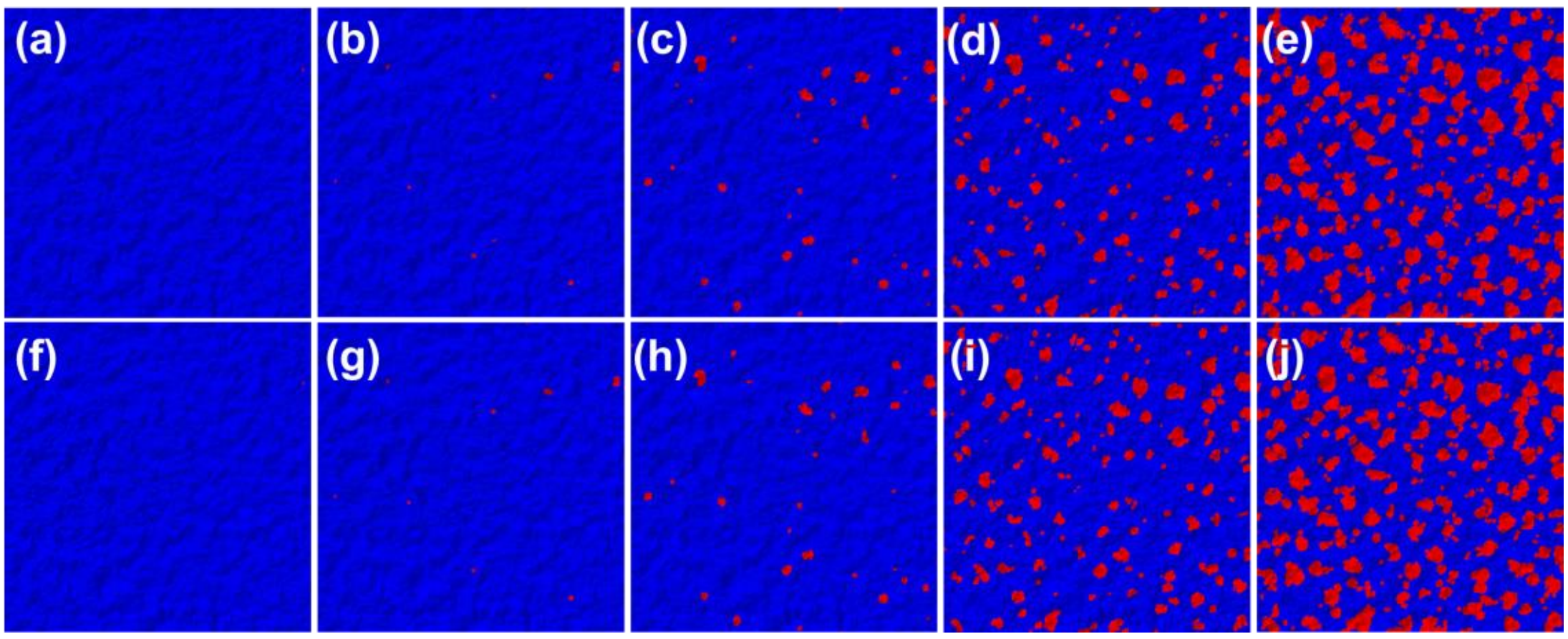

Figure S20 Real contact area (red spots showing the in-of-contact zones) after the running-in with hexadecane under the contact depth of 100 nm (a), 200 (nm) (b), 300 nm (c), 400 nm (d) and 500 nm (e); Real contact area after the running-in with DMSO under the contact depth of 100 nm (a), 200 nm (b), 300 nm (c), 400 nm (d), and 500 nm (e).

2. Are there any depth profiling results of the TOF-SIMS to support the quantitative contribution of physical and chemical effects of triboelectricity of DMSO running-in optimization as claim in the conclusion? It is essential to show the depth profiling results using TOF-SIMs as a function of the number of cycles of running-in to correlate the spatial distribution of different elements and molecules on the surface and determine the composition of the sample layer by layer.

2. Response: Thanks so much for your valuable comments. The high surface charge density of TENGs can be achieved via the DMSO-assisted running-in process that induces surface molecular chain scission and rearrangement, making electron-withdrawing groups toward the triboelectric surface. It is also found in Fig. 5a that the ultrahigh charge density can be achieved with different running-in cycles. The surface chemical properties after different running-in cycles can be identified via TOF-SIMS, which provides comprehensive mass spectrometric information on chemical surface composition and the in-depth structure of the material's surface.

Accordingly, the in-depth profiles of the molecular fragments are carried out on

the running-in treated surface via TOF-SIMS in the revised Supplementary Information. As shown in Supplementary Fig. S35, the intensity of CNO^- obviously increases with the increase of running-in cycles. The detected intensity of CNO^- in the 2D distribution is much higher on the running-in PI after more than 10 cycles (Supplementary Fig. S36). Further, it should be noted that after running-in for 100 cycles, the change trends of the intensity are not obvious, e.g., for CN^- and NH^- fragments (Supplementary Fig. S35b-c). After 1000 cycles, there is an available signal of TOF-SIMS on the CO^- fragment probably due to surface oxidation during running-in friction (Supplementary Fig. S35d). Thus, with the DMSO-driven extraction effect, the polar chemical chains are regulated to be "molecular brushes" outspread on surfaces, which could be strongly enhanced by the running-in cycles.

Changes as follow:

Page 18

Further, the in-depth profiles of the molecule fragments are carried out after running-in at different cycles via TOF-SIMS (Supplementary Fig. S35 and Fig. S36). It is found that the intensity of CNO^- obviously increases with the increase of running-in cycles. The detected intensity of CNO^- in the 2D distribution is much higher on the running-in PI after more than 10 cycles (Supplementary Fig. S35a and Fig. S36). It is also noted that after running-in for 100 cycles, the change trends of the intensity are not obvious, e.g., for CN^- and NH^- fragments (Supplementary Fig. S35b-c). After 1000 cycles, there is an available signal of TOF-SIMS on the CO^- fragment probably due to surface oxidation during running-in friction (Supplementary Fig. S35d). Thus, with the DMSO-driven extraction effect, the polar chemical chains are regulated to be "molecular brushes" outspread on surfaces, which could be strongly enhanced by the cycles of the running-in process.

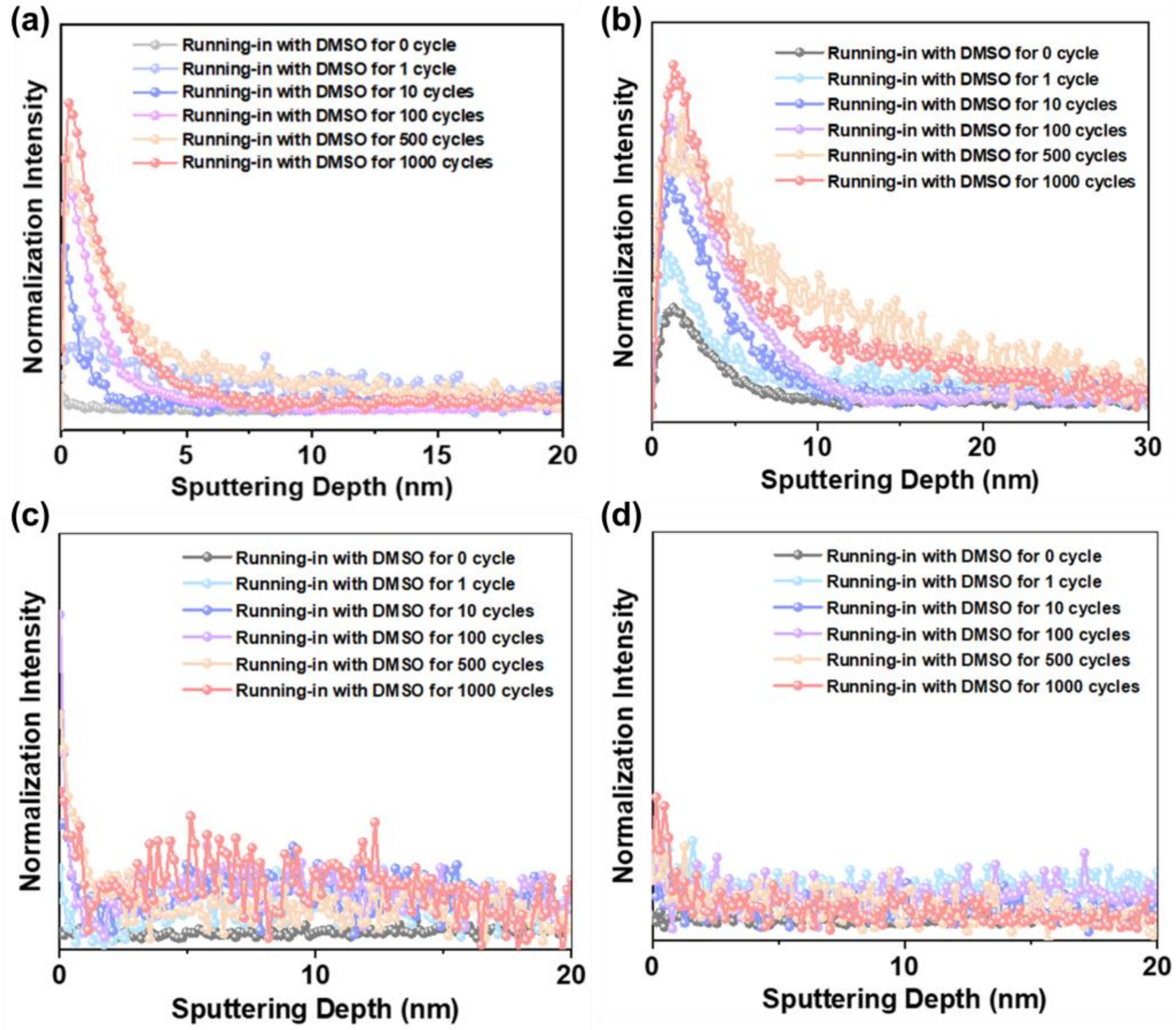

Figure S35 Content changes of CNO^-(a), CN^- (b), NH^- (c), and CO^- (d) fragments respectively along the depth direction.

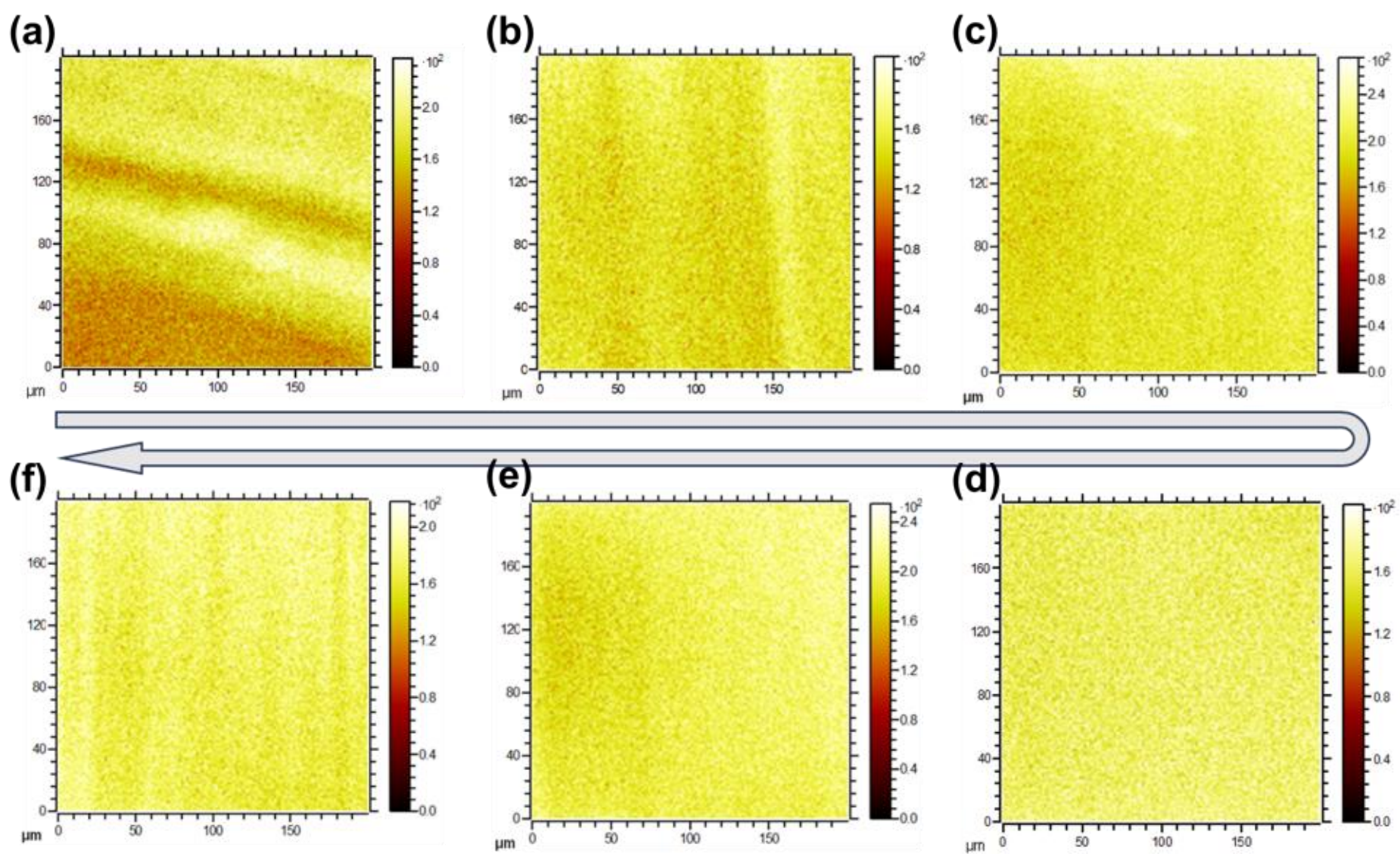

Figure S36 TOF-SIMS images showing the distributions of the chemical fragment of CNO- after running-in with DMSO for different cycles, 0 cycle (a), 1 cycle (b), 10 cycles (c), 100 cycles (d), 500 cycles (e), and 1000 cycles (f).

3. In Figure 5d-f, the charge reversal mechanism was demonstrated with the voltage and charge measurement and a triboelectric series for lubricated sliding modes which considered the running-in optimization is proposed. The authors are required to perform the surface potential evaluation and the surface roughness using the KPFM and AFM, respectively to provide more details on the minimum energy barrier and number of running-in cycles in relation to friction efficiency are required for polarization "switching" in each of the sample as claimed. Additionally, the key factors that influence polarization "switching" should be clearly outlined.

3. Response: Thanks so much for your valuable comments. It is found in Fig. 5a that the ultrahigh charge density is able to be achieved with the increase of running-in cycles. It means the scission and rearrangement of molecular chains occur quickly under the friction-induced DMSO-solvation. After 100 cycles of running-in, the electric output increases significantly, meaning the triboelectric polarity of the PI changes to much more negative through the running-in optimization, which results in the re-ranking of triboelectric series (Fig. 5h-j) according to the electric outputs (Fig. 2). Accordingly, the surface potential of the PI surface using Kelvin Probe Force Microscopy (KPFM) are carried out (Fig. 5b-d), and it is obtained that the surface potential changes obviously after the running-in process. From the extracted pixel distribution (Fig. 5b) with respect to the surface potential value, the change occurs after the first cycle, and there is a significant change in the surface potential after running-in for 100 cycles (Fig. 5c). It is evident from the KPFM results that the proposed running-in process leads to a much lower surface potential, which is correlated with a higher output in the TENG. A lower surface potential, implying a more negative potential, indicates a stronger electron-withdrawing ability[1, 2]. In contrast, the virgin PI surface exhibits a relatively high potential on the surface. This observation aligns with our findings that the ultrahigh charge density is able to be achieved with one running-in cycle (Fig. 5a). After 100 cycles of running-in, the triboelectric polarity of the PI changes to much more negative (surface potential ~ -6.98 V). It is because the scission and rearrangement of molecular chains occur quickly through the running-in optimization. Accordingly, the

surface potential via KPFM can successfully identify the change in the chemical structure of PI surfaces.

In addition, the surface roughness of the running-in PI is determined from AFM surface topography measurements. The surface roughness of the running-in PIs increases with the increase of the running-in cycle, whereas the difference in the local surface roughness is not notable (≥500 cycles) (Supplementary Fig. S33 and Fig. S34). Further, the in-depth profiles of the molecule fragments are carried out after running-in at different cycles via TOF-SIMS (Supplementary Fig. S35 and Fig. S36). It is found that the intensity of CNO^- obviously increases with the increase of running-in cycles. The detected intensity of CNO^- in the 2D distribution is much higher on the running-in PI after more than 10 cycles (Supplementary Fig. S35a and Fig. S36). It is also noted that after running-in for 100 cycles, the change trends of the intensity are not obvious, e.g., for CN^- and NH^- fragments (Supplementary Fig. S35b-c). Thus, with the DMSO-driven extraction effect, the polar chemical chains are regulated to be "molecular brushes" outspread on surfaces, which could be strongly enhanced by the cycles of the running-in process.

The frictional energy consumption based on the work done of friction forces is obtained at different running-in cycles according to the coefficient of friction (Supplementary Fig. S37) and sliding length[3]. In this regard, the surface potential decreases dramatically at an energy consumption of ~0.2 N·m (10 cycles) (Supplementary Fig. S38). Over the value of the energy consumption, the detected TOF-SIMS intensity of CNO^- in the 2D distribution is much higher (Supplementary Fig. S35a and Fig. S36). Further, it should be noted that within a frictional energy consumption of ~2.07 N·m (100 cycles), the differences of the TOF-SIMS intensity are not obvious, e.g., for CNO^-, CN^-, and NH^- (Supplementary Fig. S35a-c). At the same time, the difference in surface potential is relatively low over the frictional energy consumption, which means the friction-consumed energy ~2.07 N·m is required for achieving reliable structural evolution and regenerated triboelectric series of PIs (100 cycles) ((Fig. 5h-j and Supplementary Fig. S38).

According to triboelectric mechanism analysis (Fig. 4 and Fig. 5), the key factors that influence the triboelectric output and series re-ranking are summarized according to triboelectric mechanism analysis (Fig. 4 and Fig. 5). The chemical chains containing amide groups outspread towards contact surfaces through running-in optimization. Thus, the key factors mainly originate from the running-in period. Firstly, the running-in cycles directly influence the structure evolution of PIs. Especially, after 100 cycles of running-in, the triboelectric polarity of the PI changes to much more negative through the running-in optimization (Fig. 5b). Running-in friction can induce the breakage of the relatively unstable N-C bonds in PI, resulting in the freedom to release amide groups. In addition, although the electric output can be enhanced after running-in under the dry condition (Fig. 2c), the running-in dissolvent is necessary to regulate the chemical chains containing amide groups outspread towards contact surfaces, among which the C=O bonds with strong electron-withdrawing capability are thus exposed and capture electrons from the contacting electrode. Further, it is known that the triboelectric performances will be significantly boosted via introducing nonpolar liquids in the electric interface, whereas the ionization property of dissolvents should be focused on for the running-in process, rather than the solvent polarities. Non-proton dissolvents, e.g., DMSO, DMA, and DMF, have the key contributions to the triboelectricity in contrast to the proton and/or polar dissolvents (Fig. 2c). It is because the released ions from proton and/or polar dissolvents could adsorb on the electric surface and shield the electrostatic effect (Supplementary Fig. S7 and Fig. S8).

Changes as follows:

Page 17

Accordingly, the surface potential of the PI surface using Kelvin Probe Force Microscopy (KPFM) is carried out (Fig. 5b-d), and it is obtained that the surface potential changes obviously after the running-in process. From the extracted pixel distribution (Fig. 5b) with respect to the surface potential value, the change occurs after the first cycle, and there is a significant change in the surface potential after running-in

for 100 cycles (Fig. 5c). It is evident from the KPFM results that the proposed running-in process leads to a much lower surface potential, which is correlated with a higher output in the TENG. A lower surface potential, implying a more negative potential, indicates a stronger electron-withdrawing ability[15, 16]. This observation aligns with our findings that the ultrahigh charge density is able to be achieved with one running-in cycle (Fig. 5d). After 100 cycles of running-in, the triboelectric polarity of the PI changes to much more negative (surface potential ~ -6.98 V). It is because the scission and rearrangement of molecular chains occur quickly through the running-in optimization. Accordingly, the surface potential via KPFM can successfully identify the change in the chemical structure of PI surfaces.

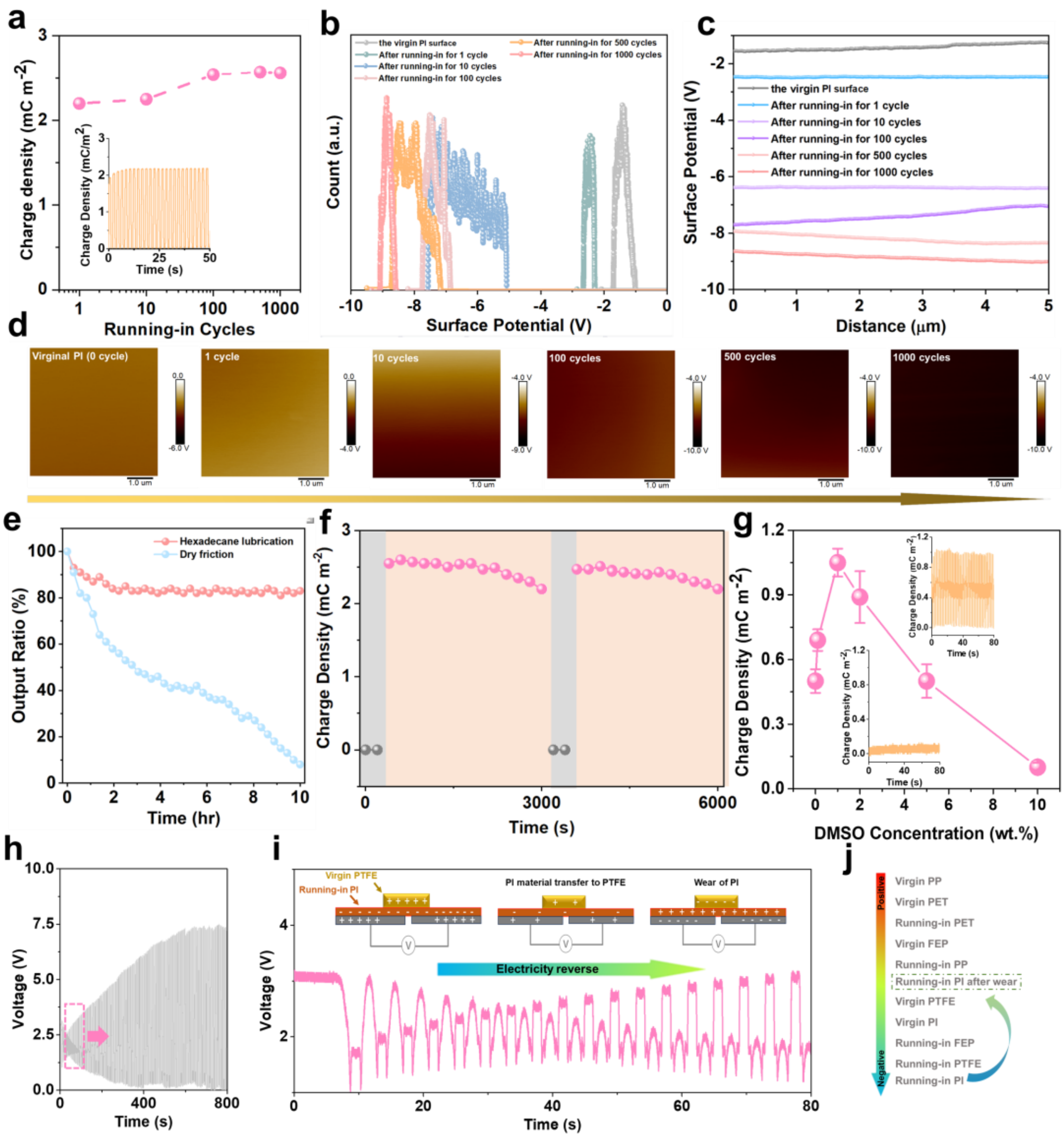

Fig. 5 Super-fast DMSO-assisted running-in strategies towards triboelectric

enhancement and reversal effect. a Relationship between the running-in cycle and the triboelectric output. **b** Relative pixel distribution with respect to the surface potential value after running-in with DMSO. **c** Extracted line scan profile with respect to the surface potential value after running-in with DMSO. **d** Surface potential profile of the PI surfaces after running-in with DMSO. **e** Electric outputs of the hexadecane-lubricated TENG after running-in with DMSO compared with the dry-friction TENG in the long-term test. **f** Charge density of the running-in PI with hexadecane lubrication after intermittent running-in processes. **g** Charge density of the virgin PI lubricated by the mixture of hexadecane and DMSO. **h** and **i** Triboelectric output of the friction pairs between the running-in PI with virgin PTFE, the inset in Figure 5i demonstrating the electric reversal mechanism of the friction pairs. **j** Triboelectric series of engineering dielectric materials for lubricated sliding modes considering the running-in optimization.

Page 18

After that, the surface roughness of the running-in PI is determined from AFM surface topography measurements. The surface roughness of the running-in PIs increases with the increase of the running-in cycle, whereas the difference in the local surface roughness is not notable (≥500 cycles) (Supplementary Fig. S33 and Fig. S34). Further, the in-depth profiles of the molecule fragments are carried out after running-in at different cycles via TOF-SIMS (Supplementary Fig. S35 and Fig. S36). It is found that the intensity of CNO^- obviously increases with the increase of running-in cycles. The detected intensity of CNO^- in the 2D distribution is much higher on the running-in PI after more than 10 cycles (Supplementary Fig. S35a and Fig. S36). It is also noted that after running-in for 100 cycles, the change trends of the intensity are not obvious, e.g., for CN^- and NH^- fragments (Supplementary Fig. S35b-c). After 1000 cycles, there is an available signal of TOF-SIMS on the CO^- fragment probably due to surface oxidation during running-in friction (Supplementary Fig. S35d). Thus, with the DMSO-driven extraction effect, the polar chemical chains are regulated to be "molecular

brushes" outspread on surfaces, which could be strongly enhanced by the cycles of the running-in process. The frictional energy consumption based on the work done of friction forces is obtained at different running-in cycles according to the coefficient of friction (Supplementary Fig. S37) and sliding length[3]. In this regard, the surface potential decreases dramatically at an energy consumption of ~0.2 N·m (10 cycles) (Supplementary Fig. S38). Over the value of the energy consumption, the detected TOF-SIMS intensity of CNO^- in the 2D distribution is much higher (Supplementary Fig. S35a and Fig. S36). Further, it should be noted that within a frictional energy consumption of ~2.07 N·m (100 cycles), the differences of the TOF-SIMS intensity are not obvious, e.g., for CNO^-, CN^-, and NH^- (Supplementary Fig. S35a-c). At the same time, the difference in surface potential is relatively low over the frictional energy consumption, which means the friction-consumed energy ~2.07 N·m is required for achieving reliable structural evolution and high electric outputs of PIs (100 cycles) (Supplementary Fig. S38).

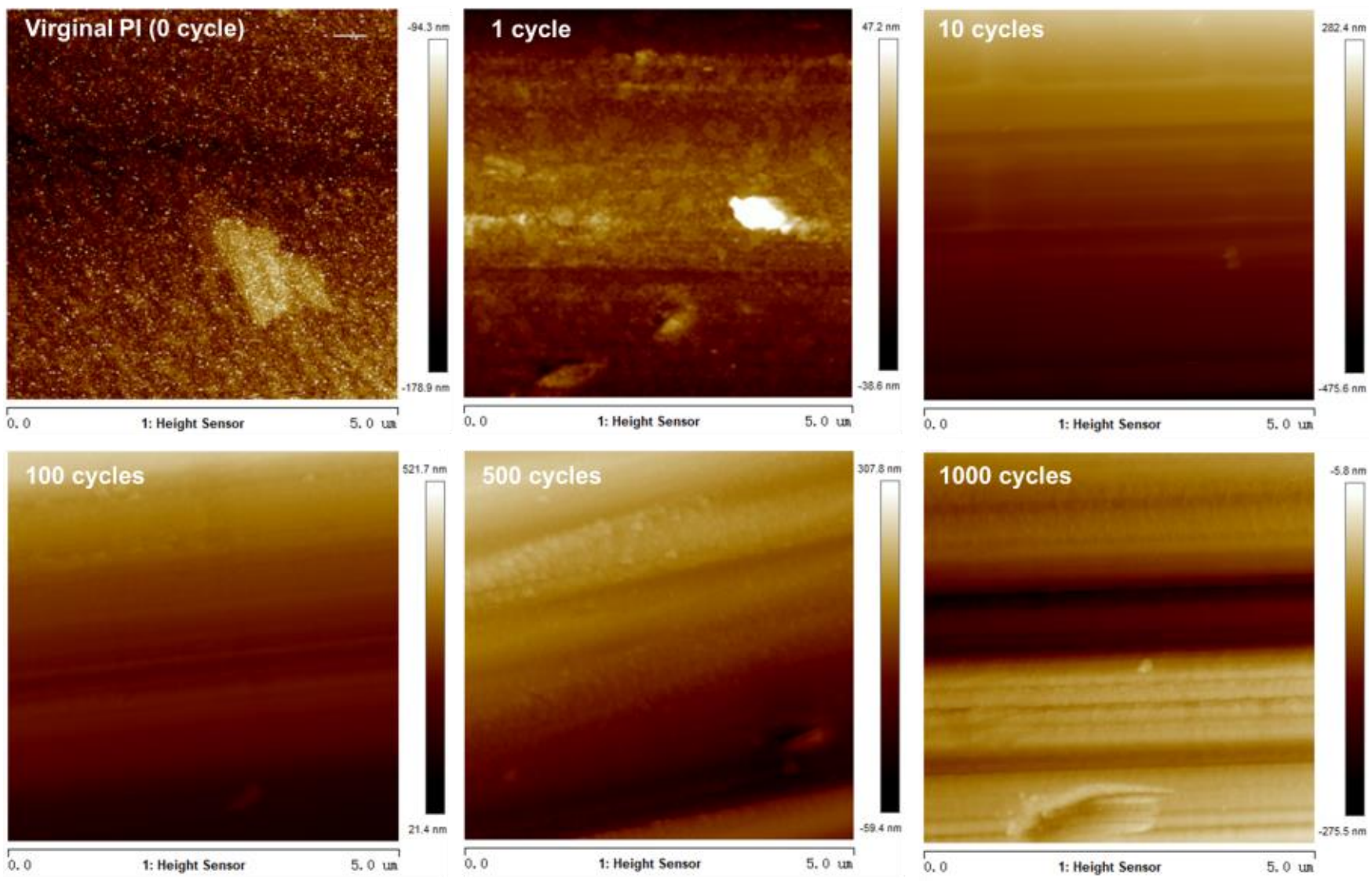

Figure S33 Surface morphology of the PI after running-in with DMSO for different cycles through AFM analysis.

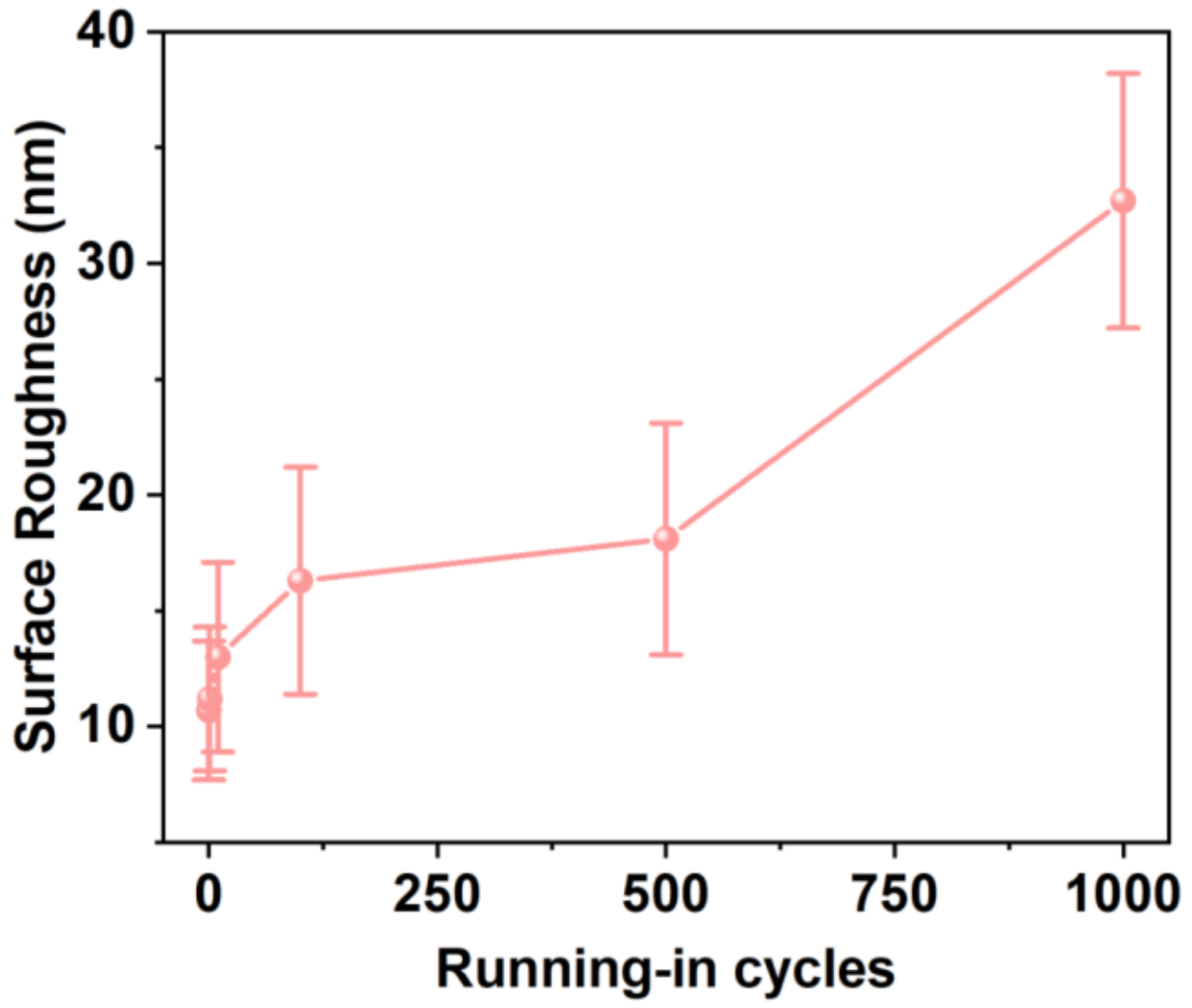

Figure S34 Surface roughness of the PI after running-in with DMSO at different cycles through AFM analysis.

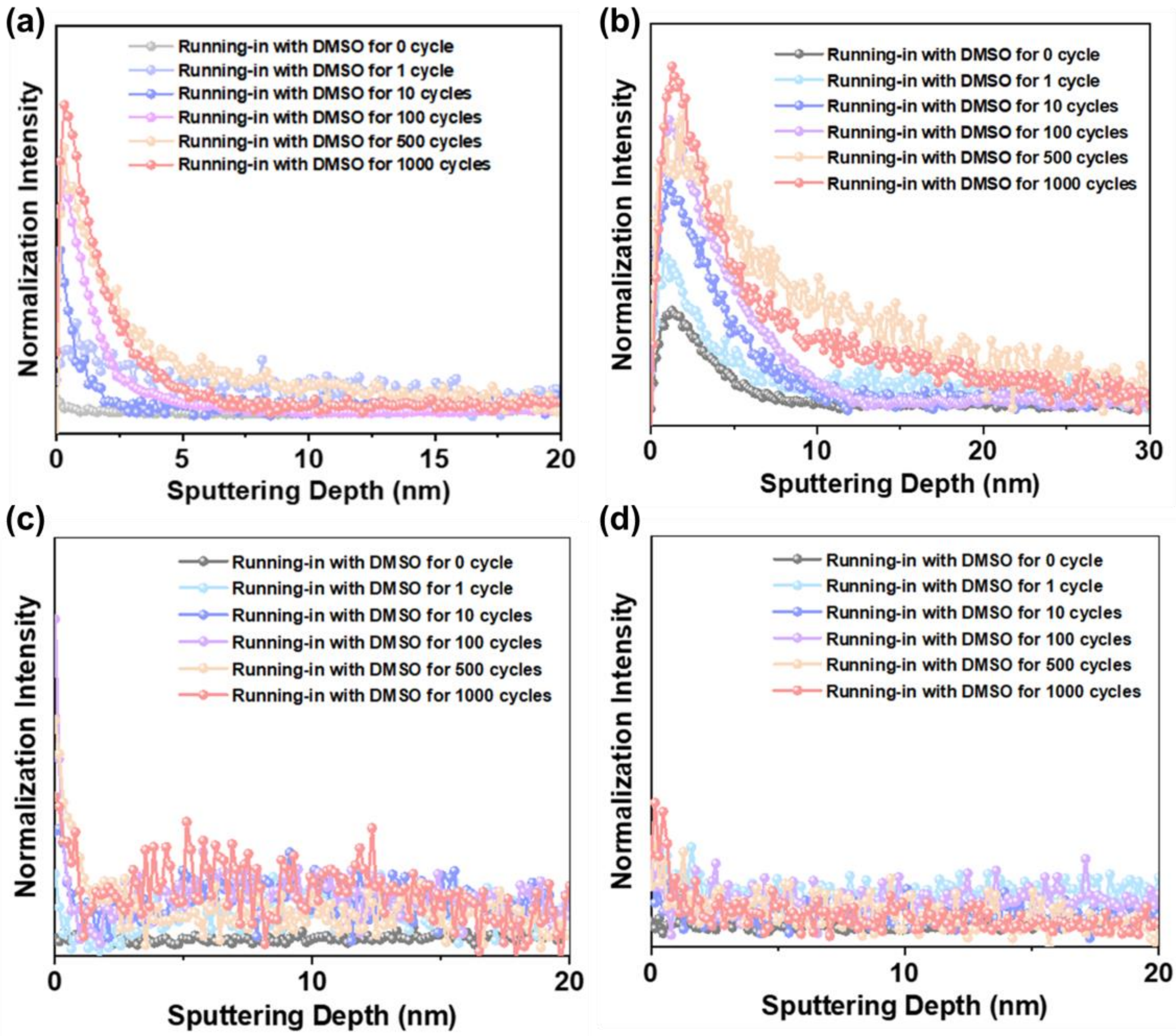

Figure S35 Content changes of CNO^-(a), CN^- (b), NH^- (c), and CO^- (d) fragments respectively along the depth direction.

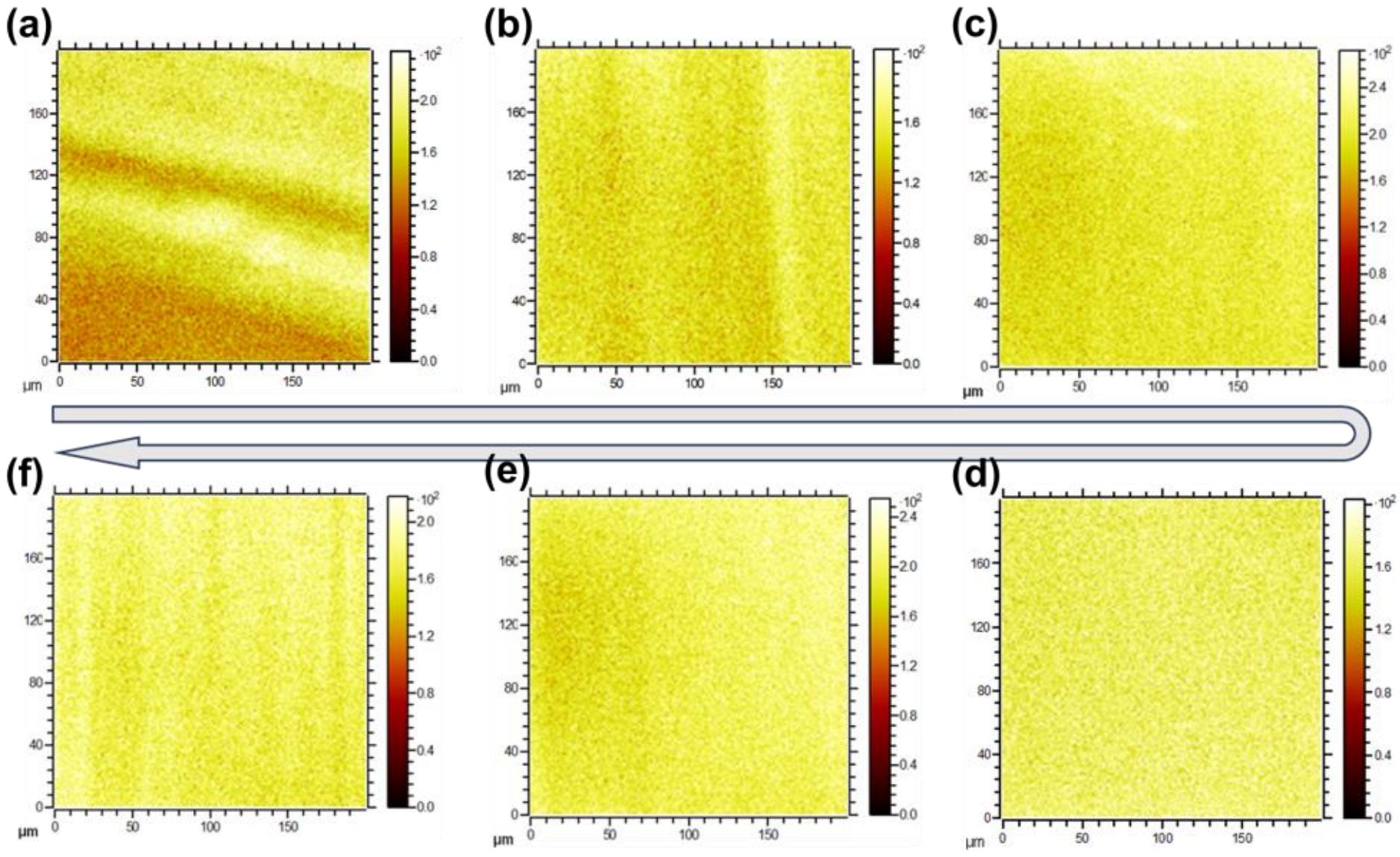

Figure S36 TOF-SIMS images showing the distributions of the chemical fragment of CNO- after running-in with DMSO for different cycles, 0 cycle (a), 1 cycle (b), 10 cycles (c), 100 cycles (d), 500 cycles (e), and 1000 cycles (f).

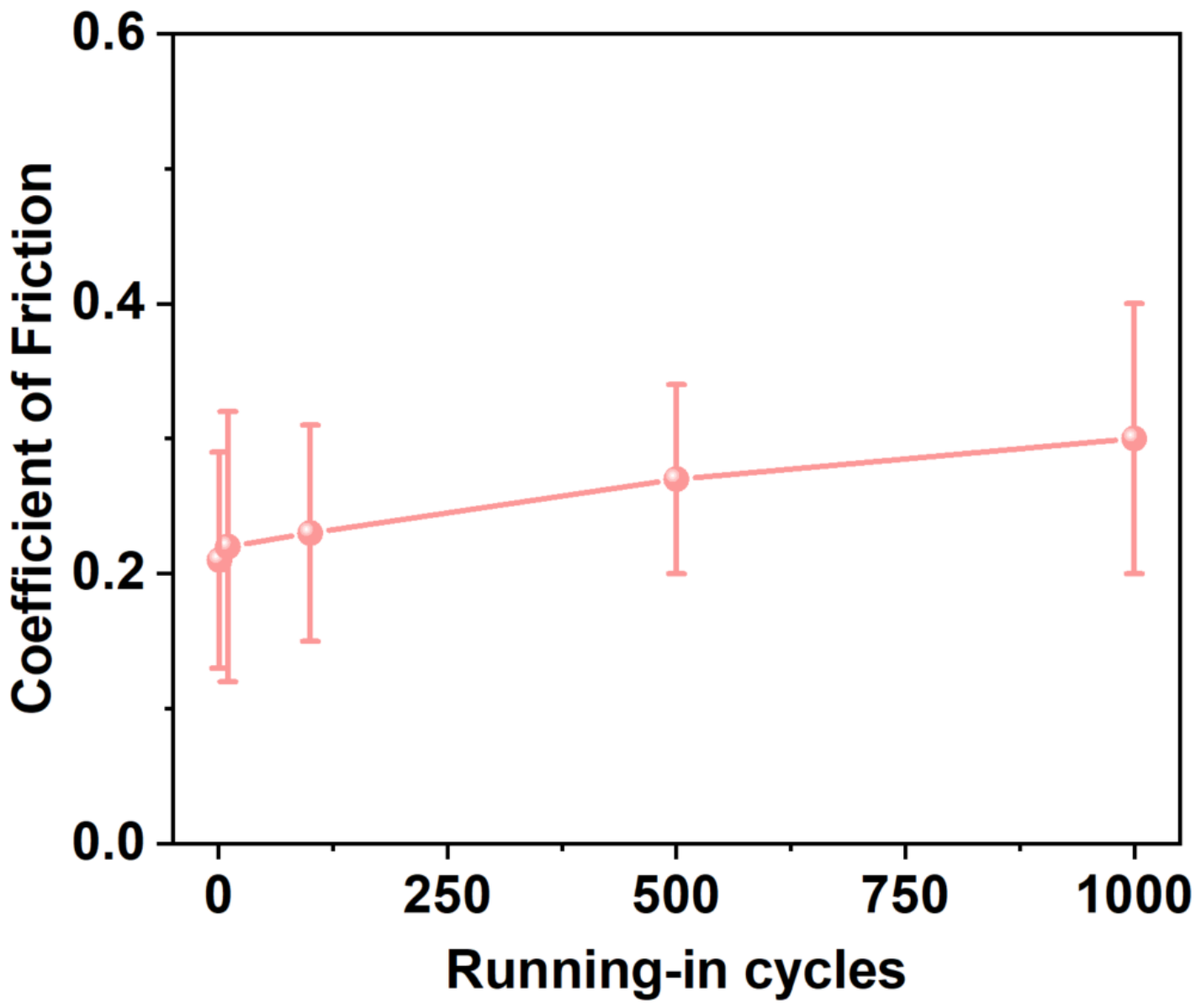

Figure S37 Coefficient of friction of the PI-based TENG as a function of DMSO-assisted running-in cycles.

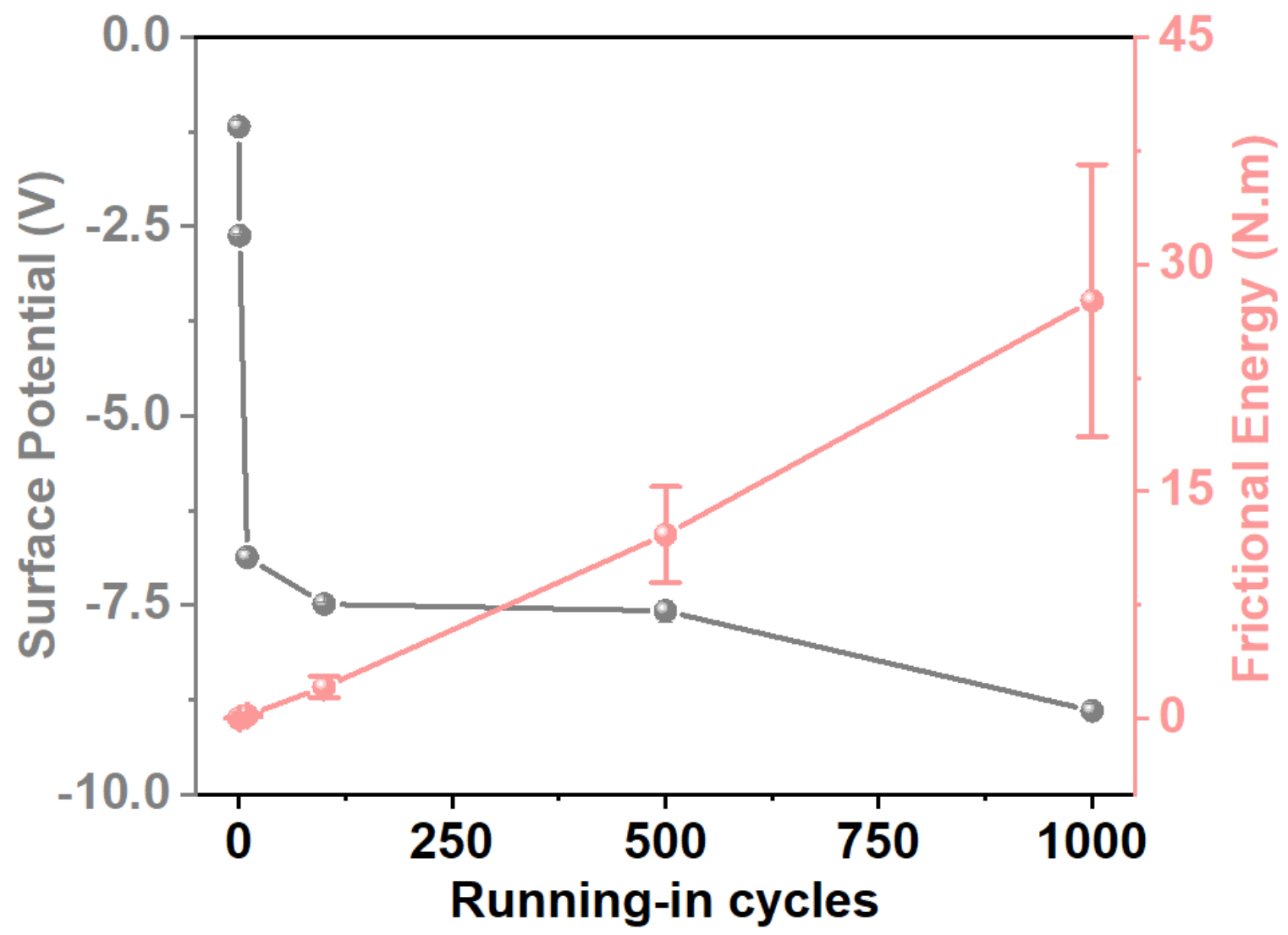

Figure S38 Surface potential and frictional energy consumption based on the work done of friction forces as a function of running-in cycles.

Page 22

The key factors that influence the triboelectricity of TENGs are summarized according to triboelectric mechanism analysis (Fig. 4 and Fig. 5). The chemical chains containing amide groups outspread towards contact surfaces through running-in optimization. Firstly, the running-in cycles directly influence the structure evolution of PIs. Especially, after 100 cycles of running-in, the triboelectric polarity of the PI changes to much more negative through the running-in optimization (Fig. 5b). Running-in friction can induce the breakage of the relatively unstable N-C bonds in PI, resulting in the freedom to release amide groups. In addition, although the electric output can be enhanced after running-in under the dry condition (Fig. 2c), the running-in dissolvent is necessary to regulate the chemical chains containing amide groups outspread towards contact surfaces, among which the C=O bonds with strong electron-withdrawing capability are thus exposed and capture electrons from the contacting tribolayer. Further, it is known that the triboelectric performances will be significantly boosted via introducing nonpolar liquids in the electric interface, whereas the ionization property of dissolvents should be focused on for the running-in process, rather than the solvent polarities. Non-proton dissolvents, e.g., DMSO, DMA, and DMF, have the key

contributions to the triboelectricity in contrast to the proton and/or polar dissolvents (Fig. 2c). It is because the released ions from proton and/or polar dissolvents could adsorb on the electric surface and shield the electrostatic effect (Supplementary Fig. S7 and Fig. S8).

4. On simulation part, the flow of the results and discussion is disrupted, not easy to follow. Specifically, the effects of the non-polar and polar solvent on the charge transfer/shielding mechanism are not presented in a clear and intuitive manner. The overall discussion of the MD & DFT simulation (mainly focusing on DMSO only) to support the experimental observation lacks cohesion. The authors should reorganize the narrative to improve the flow and ensure that the progression of ideas is logical and easier to follow.

4. Response: Thanks so much for your valuable comments. Firstly, the schematic diagram of the device and triboelectric mechanism are added in Fig. 1b in the revised manuscript in a clear and intuitive manner. Triboelectrification works on the coupled mechanism of contact electrification and electrostatic induction. In the dry condition, electrons are usually transferred from the Al tribolayer to the PI surface during contact electrification. Electric outputs will be achieved when the Al tribolayers are sliding against the PI surfaces because of electrostatic induction (Fig. 1a). For the case of lubrication, a thin liquid film (normally less than several tens of nanometers) will be formed at the interface[4]. Due to the presence of roughness for contact surfaces, some small liquid pockets will be generated. When the external force is applied, the liquid film is ruptured for some asperities and solid-solid contact is dominated at the electric interface (at the right of Fig. 1b), which means the contact electrification occurs between the Al tribolayers and the PI surface. In contrast to dry friction in air (Supplementary Fig. S1), liquid lubricants can significantly suppress interfacial breakdown due to higher breakdown field strength requirement and lower electrostatic field strength in the microgap between the Al tribolayer and the PI surface. Further, when the charged PI surface is submerged in the lubricant, the surface charge cannot be screened by the nonpolar lubricants, e.g., hexadecane, squalane, and paraffin oil,

which have a larger Debye length than polar liquids[5]. Because of the electrostatic induction between the charged PI surface and the Al electrode, electrons will flow from the Al electrode to the ground under short-circuit conditions for an electric equilibrium (Fig. 1a).

Accordingly, there are two motivations of the simulation: one is to reveal the evolution of the molecular structure of PI activated by the DMSO dissolvent through MD simulation; another is to identify the triboelectric properties of the activated molecular groups of PI in contact with the Al tribolayer lubricated by the hexadecane liquid via DFT simulation. In this case, the results of the DFT simulation are obtained under the condition of a hexadecane environment in the revised manuscript, which is more practical and accurate (the DFT simulation in the previous manuscript is based on a vacuum state).

In the part of the MD simulation, it is found that the value of RDF between the S atoms in DMSO and the N atoms in the running-in PI increases gradually at a distance ranging from 3.3 Å to 9.9 Å with the time relaxation (Fig. 4c), in which the peaks of the RDF values are mainly distributed around 6 Å. It means the DMSO molecule effectively infiltrates into the molecular chains of PI, which is also confirmed by the result of RDF between the S atoms in DMSO and the O atoms in the running-in PI shown in Fig. 4d

After running-in with DMSO, the contact surfaces are then fully cleaned, and a lubricant, e.g., hexadecane, is added to the sliding interface (Fig. 1b). The DFT simulation is thus introduced to demonstrate the atom-level triboelectricity involving the activated groups of PI from the MD simulation part under the lubrication of hexadecane.

To further improve the cohesion of the MD & DFT simulation correlated to the experimental, more demonstrations have been revised in the revised manuscript. In addition, we also carefully reorganize the narrative to improve the flow and ensure that the progression of ideas is more logical and easier to follow, which are shown as follows.

Changes as follows:

Triboelectrification works on the coupled mechanism of contact electrification and electrostatic induction. Under the dry conditions, electrons are usually transferred from the Al tribolayer to the PI surface during contact electrification. Electric outputs will be achieved when the Al tribolayers are sliding against the PI surfaces due to electrostatic induction. For the case of lubrication, a thin liquid film (normally less than several tens of nanometers) will be formed at the interface[4]. Due to the presence of roughness on contact surfaces, some small liquid pockets will be generated. When an external force is applied, the liquid film ruptures at certain asperities, and solid–solid contact becomes dominant at the electrical interface (Fig. 1b), which means the contact electrification occurs between the Al tribolayers and the PI surface. In contrast to dry friction in air (Supplementary Fig. S1), liquid lubricant can significantly suppress interfacial breakdown due to its higher breakdown field strength requirement and lower electrostatic field strength in the microgap between the Al tribolayer and the PI surface. Further, when the charged PI surface is submerged in the lubricant, the surface charge will not be screened by the nonpolar lubricants, which have a larger Debye length than polar liquids[5]. Because of the electrostatic induction between the charged PI surface and the Al electrode, electrons will flow from one Al electrode to another electrode under short-circuit conditions for an electric equilibrium (Fig. 1a).

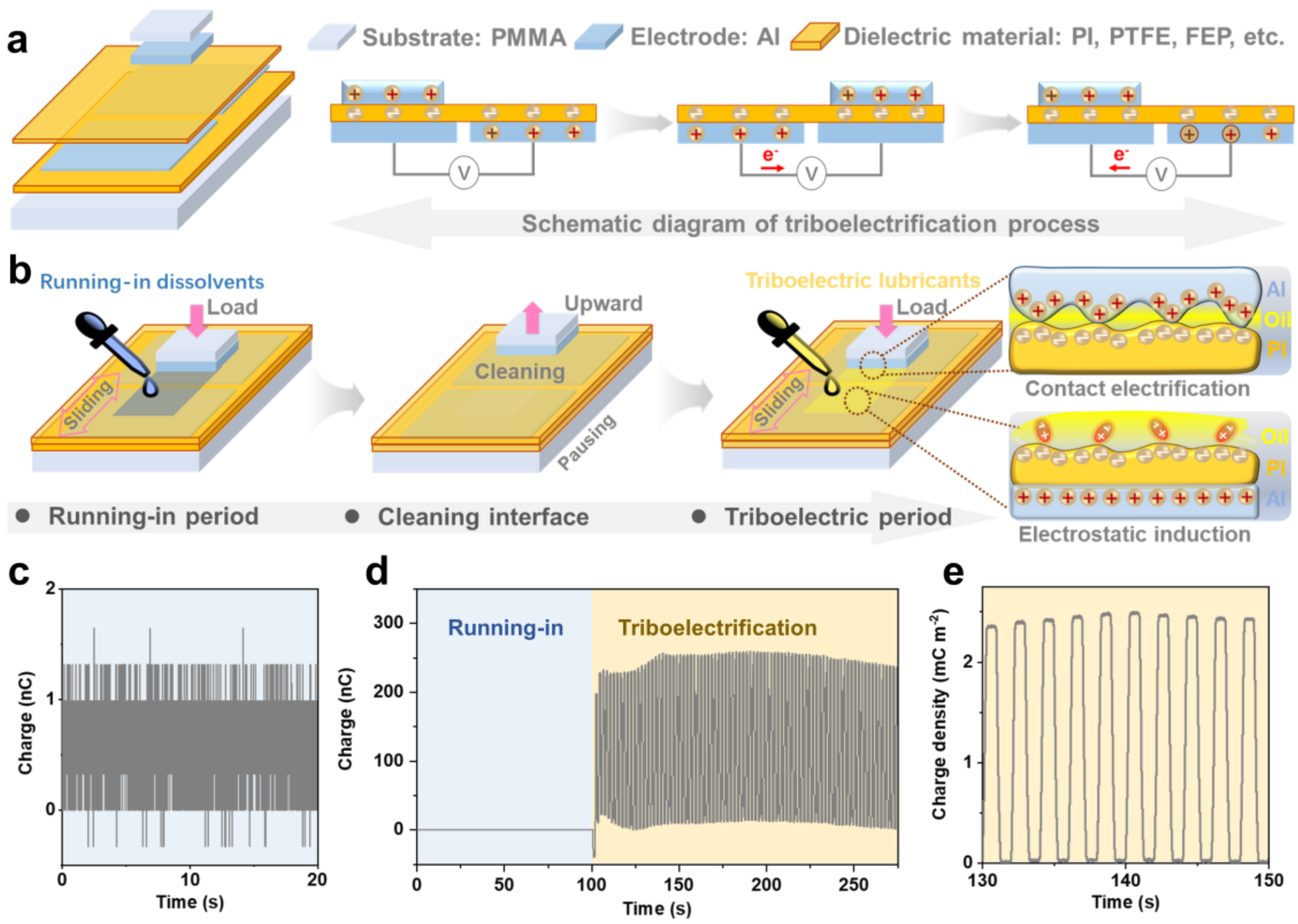

Fig. 1 Demonstration of the TENG via optimizing the running-in process. a Schematic diagram of device structure and triboelectric mechanism. **b** Optimizing process for the running-in and triboelectrification. **c** Electric output during the running-in process. **d** Electric output for the triboelectric period. **e** Charge density during the triboelectric period.

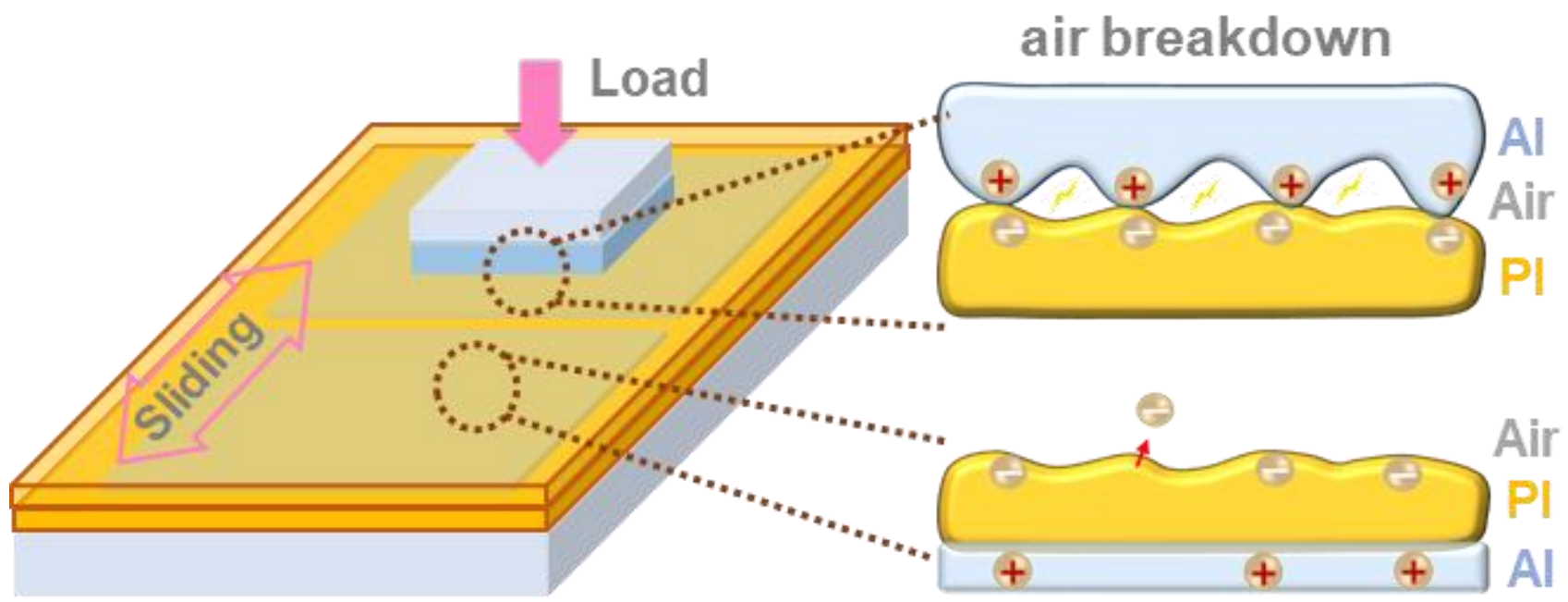

Figure S1 Triboelectrification mechanism diagram of the PI-Al friction pair under dry friction in air.

Molecular origin of the running-in process boosting the charge density. As shown in Fig. 1, superhigh charge density of TENGs can be achieved through two parts, i.e., the running-in process with the assistance of DMSO and the triboelectric period with the lubrication of hexadecane. Firstly, to further understand the DMSO-assisted solvent activation, the molecular models of the PI sliding against a virtual Al within DMSO lubrication were developed by a ReaxFF-based reactive molecular dynamics (MD) simulation and a polymer consistent force field (PCFF)-based classical MD simulation (**Methods**).

Page 12

The value of RDF between the S atoms in DMSO and the N atoms in the running-in PI increases gradually at a distance ranging from 3.3 Å to 9.9 Å with the time relaxation (Fig. 4c), in which the peaks of the RDF values are mainly distributed around 6 Å. It means the DMSO molecule effectively infiltrates into the molecular chains of PI, which is also confirmed by the result of RDF between the S atoms in DMSO and the O atoms in the running-in PI shown in Fig. 4d.

Page 13

After running-in with DMSO, the contact surfaces are then fully cleaned, and a lubricant, e.g., hexadecane, is added to the sliding interface (Fig. 1b). The density functional theory (DFT) simulation is thus introduced to demonstrate the atom-level triboelectricity involving the activated groups of PI from the results of the MD simulation under the lubrication of hexadecane. It is found from the MD simulation that the N-related bonds in PI are broken, resulting in molecular chain scission at the C-N bonds during the running-in with DMSO (Fig. 4e).

References

1. Li, Y. et al. Advanced Dielectric Materials for Triboelectric Nanogenerators: Principles, Methods, and Applications. *Adv. Mater.* **36**, 53 (2024).

2. Zhao, K. et al. Layer-Engineered Hybrid MoS2 Nanosheets for Synergistic Charge Trapping and Superlubrication toward High-Output Ultra-Durable Triboelectric Nanogenerators. *Adv. Funct. Mater.*, 16 (2025).
3. Sherwood, B.A. & Bernard, W.H. Work and Heat-Transfer in the Presence of Sliding Friction. *Am. J. Phys.* **52**, 1001-1007 (1984).
4. Wu, J., Xi, Y.H. & Shi, Y.J. Toward wear-resistive, highly durable and high performance triboelectric nanogenerator through interface liquid lubrication. *Nano Energy* **72**, 9 (2020).
5. Chung, S.H. et al. Nonpolar Liquid Lubricant Submerged Triboelectric Nanogenerator for Current Amplification via Direct Electron Flow. *Adv. Energy Mater.* **11**, 8 (2021).

Reviewer: 5

The manuscript presents a novel surface modification approach aimed at enhancing the performance of TENGs. The use of dissolvent-assisted running-in processes, particularly involving DMSO and hexadecane, contributes a degree of innovation. However, the overall presentation suffers from a lack of organization, insufficient data analysis, and weak comparisons to existing literature. Several key claims are not adequately substantiated, and multiple aspects of the methodology require clarification and further validation. Below are detailed comments and suggestions for revision before its acceptance for publication.

1. Lack of Experimental Support for Dissolvent Mechanism:

The manuscript asserts that the use of dissolvents facilitates removal of surface radicals and debris while preventing material transfer. However, this claim is not backed by direct experimental evidence. The authors are suggested to provide a comprehensive surface analysis (e.g., XPS, AFM, SEM) and charge transfer characterization across all sample sets, ideally including a calibrated reference sample. The influence of environmental parameters such as humidity and chemical environment must also be addressed.

1. Response: Thanks so much for your valuable comments. During friction, the wear debris and radicals from the rubbing interface are easily accommodated in the lubricant, which has been widely found in previous works[1, 2]. In this study, the use of dissolvents, e.g., DMSO, is able to accommodate surface radicals and debris and to facilitate their removal from the electric interface. The direct experimental evidence has been provided in the revised manuscript. As shown in Supplementary Fig. S3, the color of the used DMSO dissolvent after running-in changes to yellow in contrast to the virgin DMSO, which is because the worn debris of the PI dissolved into the DMSO during friction. According to the Transmission electron microscopy (TEM) analysis, the PI debris has a strip-like shape with a size of several hundred nanometers (Supplementary Fig. S4).

The use of dissolvents can also prevent material transfer of the electric tribolayers, so that the shielding effect can be weakened greatly, which is identified in our early work and others[3, 4]. In this study, the contribution of dissolvents to preventing material transfer is also discussed. During friction, added liquids, e.g., hexadecane and DMSO, can separate the contact asperities and prevent them from adsorbing onto the PI tribolayer. According to the SEM morphology and EDS analysis (Supplementary Fig. S16), there are few Al elements transferred on the PI surface. In contrast, many abrasive grains are transferred from the Al electrode to the dry-frictional PI surface (Supplementary Fig. S15). At the same time, the worn surface of the PI treated with DMSO is much rougher (Supplementary Fig. S17). It is believed that the DMSO-assisted running-in process has a surface grinding effect, so that the worn-out and transferred materials are difficult to adhere to the PI surface.

This study found that the running-in cycle has a significant influence on the electric output (Fig. 5a). To characterize the charge transfer properties of materials, KPFM is introduced to study the surface potential of the electric surfaces. Accordingly, the surface potential of the PI surface using KPFM is carried out before running-in and after that for different cycles in the revised Manuscript. Shown in Fig. 5b-d, the surface potential changes obviously after the running-in process. From the extracted pixel distribution (Fig. 5b) with respect to the surface potential value, the change occurs after the first cycle, and there is a significant change in the surface potential after running-in for 100 cycles. It is evident from the KPFM measurements that the proposed running-in process leads to a much lower surface potential, which is correlated with a higher output in the TENG. A lower surface potential, implying a more negative potential, indicates a stronger electron-withdrawing ability[5, 6]. In contrast, the virgin PI surface exhibits a relatively high potential on the surface. This observation aligns with our findings that the ultrahigh charge density is able to be achieved with one running-in cycle. After 100 cycles of running-in, the triboelectric polarity of the PI changes to much more negative. It is because the scission and rearrangement of molecular chains occur quickly through the running-in optimization. Accordingly, the surface potential

via KPFM measurements can successfully identify the charge transfer properties of PI surfaces.

It is believed that the environmental parameters, such as humidity and chemical environment, also have a great influence on the electric output. Therefore, the influence of environmental stresses, e.g., gas, humidity, and temperature, on the electric performance is also carried out in the revised manuscript. As shown in Supplementary Fig. S41a, the electric output of the lubricated TENG maintains a relatively high retention rate of 90% at a high humidity of 60%RH. In contrast to the TENG under dry friction, the lubricated TENG has a much better performance of humidity resistance. It is because the electric interface is fully packaged with the lubricant so that the water is difficult to diffuse into the Al tribolayer and the PI surface[7, 8]. Further, it is found that the gas atmospheres, e.g., O_2, N_2, and CO_2, have little impact on the electric output of the lubricated TENGs (Supplementary Fig. S41b-d), which is very different from that of the TENG under dry friction. At the beginning of dry friction, the effect of O_2 on the TENGs is not obvious when the concentration is lower than 50% (Supplementary Fig. S41b). Whereas, the ratio of voltage output is reduced by about 40% as the O_2 concentration gradually exceeds 70% because of the adsorption of O_2 onto the contact surface under dry friction[9, 10]. Similarly, the electric output of the dry-frictional TENG gradually decreases with the increase of N_2 concentration (Supplementary Figure S41c). Both the lubricated and dry-frictional TENGs keep a high and stable electric value in the CO_2 atmosphere[10] (Supplementary Fig. S41d). Furthermore, a systematic investigation of the impact of environmental temperature on the electrical performance of TENGs is essential, considering their application across diverse regions worldwide, where recorded extreme temperature can be as high as 55 °C[11]. Although the electric output of the TENG decreases with the increase of temperature, the reduction in the output ratio is not obvious up to 50 °C (Supplementary Fig. S42).

Changes as follows:

Page 6

The used dissolvents have the function of carrying worn radicals and debris, which is confirmed by the collected DMSO after the running-in process (Supplementary Fig. S3). The used DMSO is found to change color to yellow as a result of dissolving worn components, which displays a strip-like shape with dimensions of several hundred nanometers (Supplementary Fig. S4).

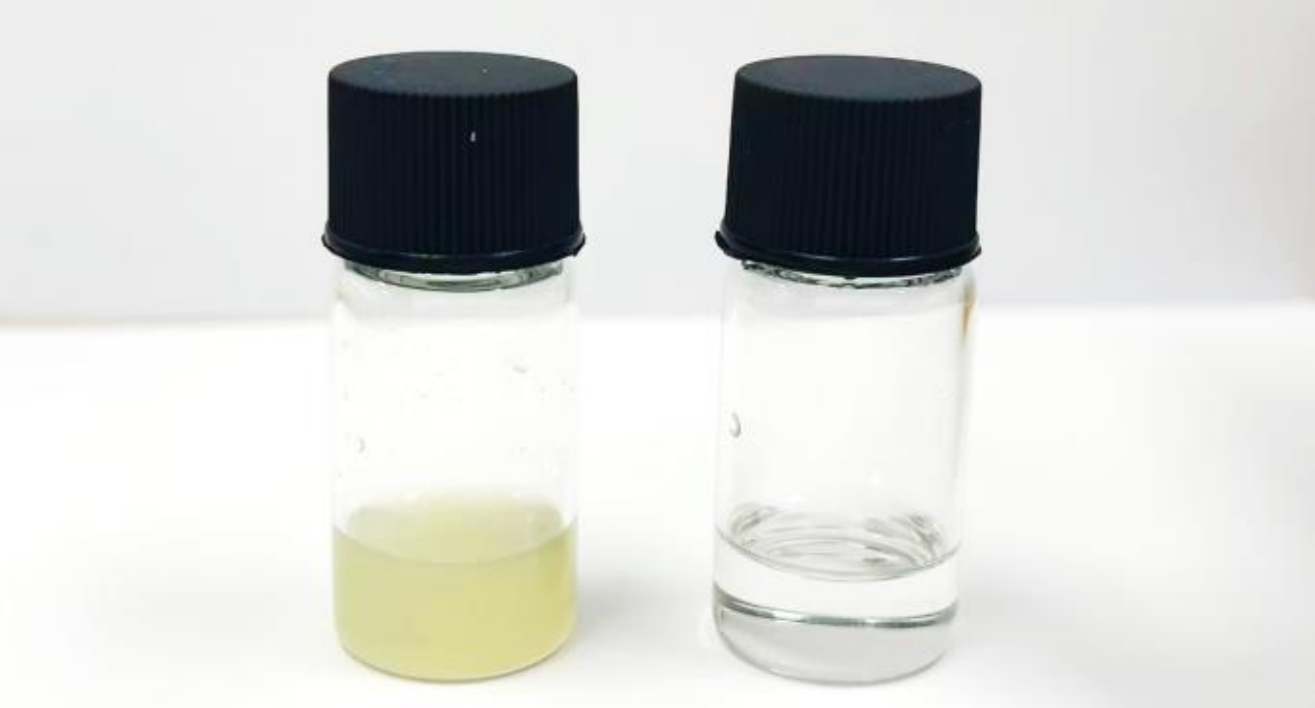

Figure S3 Pictures of the collected DMSO from the electric surface after running-in for 1000 cycles (at the left) compared with the virgin DMSO (at the right)

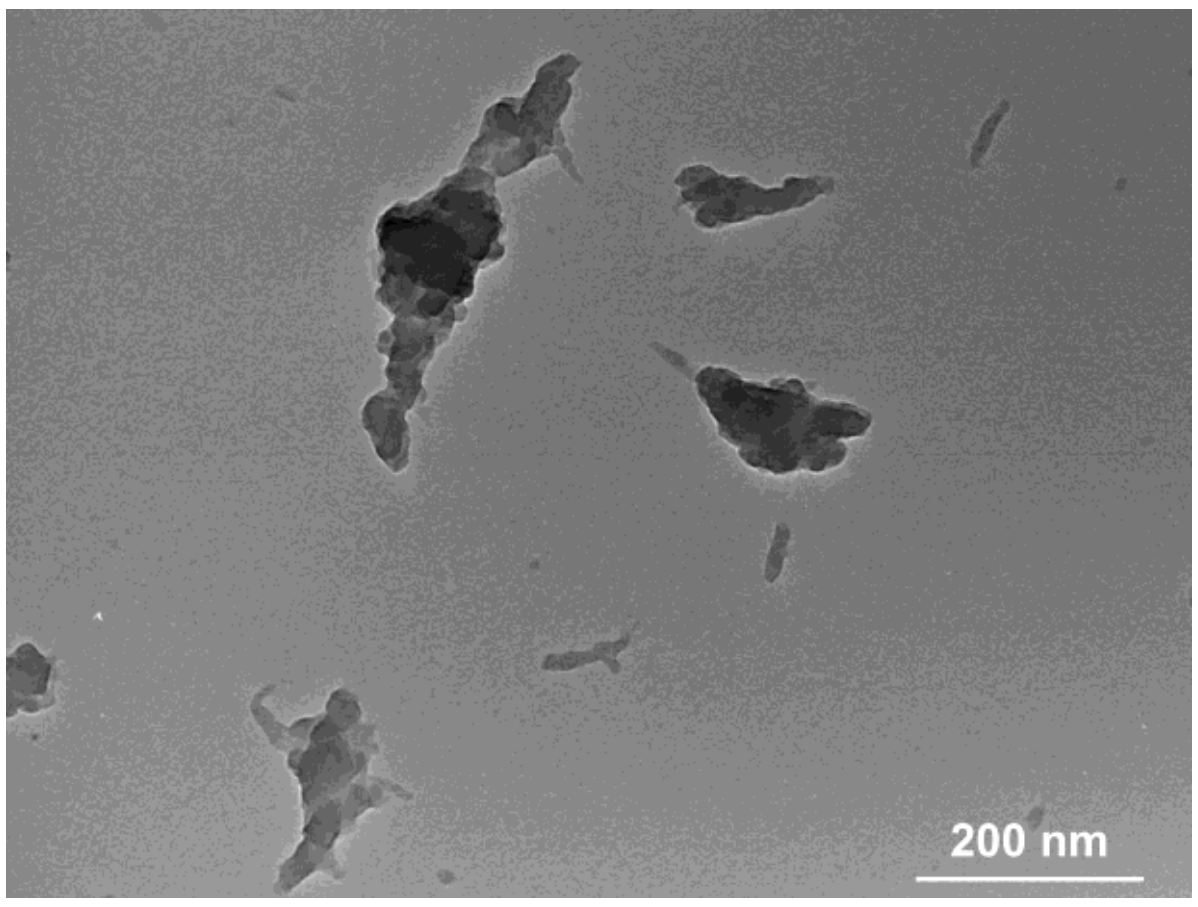

Figure S4 Transmission electron microscopy (TEM) image of the worn debris collected from the electric surface (from Figure S3)

Page 17

Accordingly, the surface potential of the PI surface using Kelvin Probe Force Microscopy (KPFM) is carried out (Fig. 5b-d), and it is obtained that the surface potential changes obviously after the running-in process. From the extracted pixel distribution (Fig. 5b) with respect to the surface potential value, the change occurs after the first cycle, and there is a significant change in the surface potential after running-in

for 100 cycles (Fig. 5c). It is evident from the KPFM results that the proposed running-in process leads to a much lower surface potential, which is correlated with a higher output in the TENG. A lower surface potential, implying a more negative potential, indicates a stronger electron-withdrawing ability[15, 16]. This observation aligns with our findings that the ultrahigh charge density is able to be achieved with one running-in cycle (Fig. 5d). After 100 cycles of running-in, the triboelectric polarity of the PI changes to much more negative (surface potential ~ -6.98 V). It is because the scission and rearrangement of molecular chains occur quickly through the running-in optimization. Accordingly, the surface potential via KPFM can successfully identify the change in the chemical structure of PI surfaces.

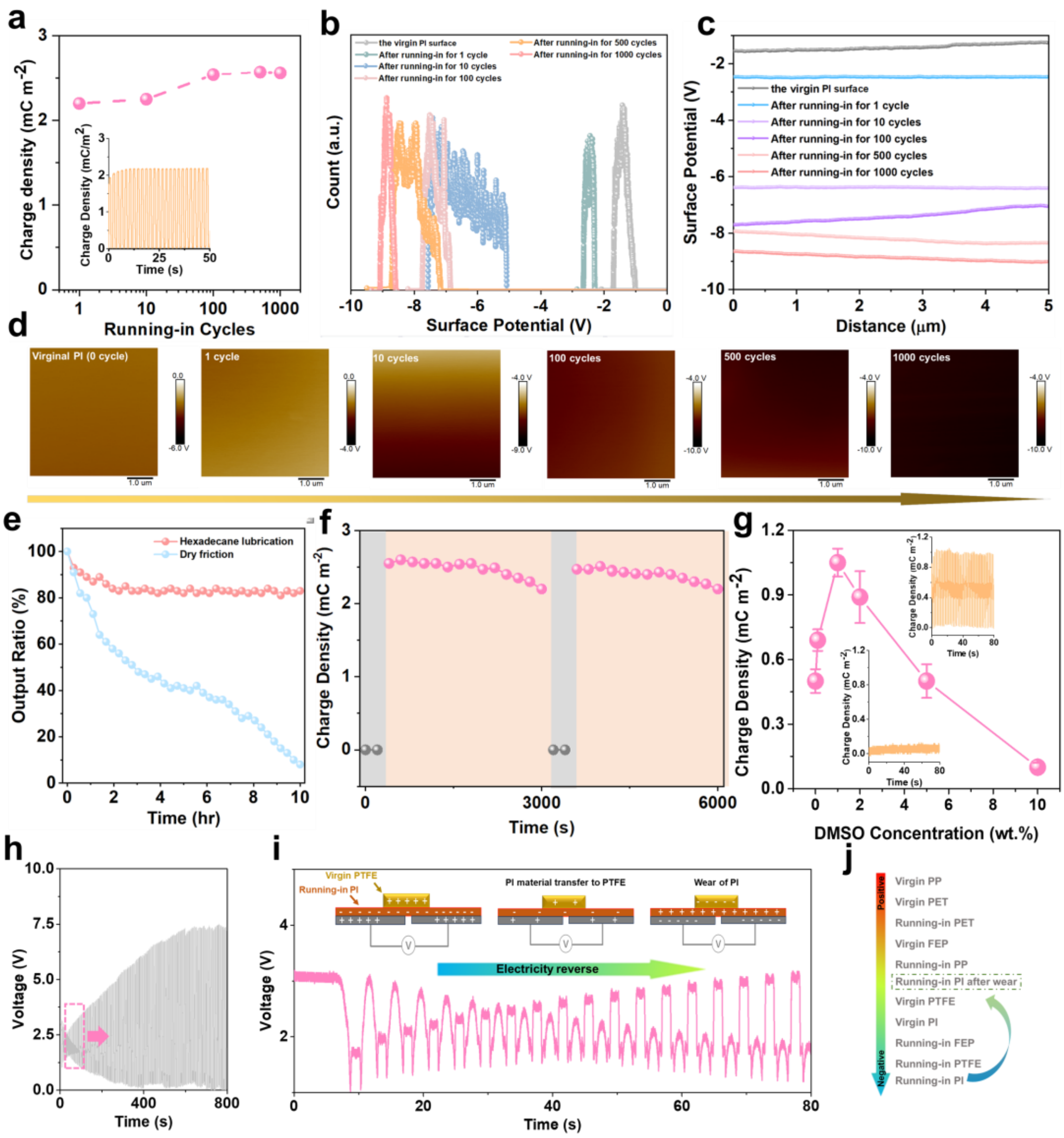

Fig. 5 Super-fast DMSO-assisted running-in strategies towards triboelectric

enhancement and reversal effect. **a** Relationship between the running-in cycle and the triboelectric output. **b** Relative pixel distribution with respect to the surface potential value after running-in with DMSO. **c** Extracted line scan profile with respect to the surface potential value after running-in with DMSO. **d** Surface potential profile of the PI surfaces after running-in with DMSO. **e** Electric outputs of the hexadecane-lubricated TENG after running-in with DMSO compared with the dry-friction TENG in the long-term test. **f** Charge density of the running-in PI with hexadecane lubrication after intermittent running-in processes. **g** Charge density of the virgin PI lubricated by the mixture of hexadecane and DMSO. **h** and **i** Triboelectric output of the friction pairs between the running-in PI with virgin PTFE, the inset in Figure 5i demonstrating the electric reversal mechanism of the friction pairs. **j** Triboelectric series of engineering dielectric materials for lubricated sliding modes considering the running-in optimization.

Page 20

In addition, the influence of environmental stresses, e.g., gas, humidity, and temperature, is also carried out for the lubricated TENG after the running-in process, in contrast to the TENG under dry friction. As shown in Supplementary Figure S41, the electric output of the lubricated TENG maintains a relatively high retention rate of 90% at a high humidity of 60%RH, which has a much better performance of humidity resistance than that under dry friction. It is because the electric interface is fully covered with the lubricant so that the water is difficult to diffuse into the Al tribolayer and the PI surface[1, 2]. Further, it is found that the gas atmospheres, e.g., O_2, N_2, and CO_2 have little impact on the electric output of the lubricated TENGs after running-in with DMSO (Supplementary Figure S41b-d), which is very different from that of the TENG under dry friction. At the beginning of dry friction, the effect of O_2 on the TENGs is not obvious when the concentration is lower than 50% (Supplementary Figure S41b). Whereas, the ratio of voltage output is reduced by about 40% as the O_2 concentration gradually exceeds 70% because of the adsorption of O_2 onto the contact surface under

dry friction[3, 5]. Similarly, the electric output of the dry-frictional TENG gradually decreases with the increase of N_2 concentration (Supplementary Figure S41c). Both the lubricated and dry-frictional TENGs keep a high and stable electric value in a CO_2 atmosphere[3] (Supplementary Figure S41d). Furthermore, a systematic investigation of the impact of environmental temperature on the electrical performance of TENGs is essential, considering their application across diverse regions worldwide, where recorded extreme temperature can be as high as 55 °C[4]. Although the electric output decreases dramatically with the increase of temperature, the reduction in the output ratio is less obvious up to 50 °C (Supplementary Figure S42).

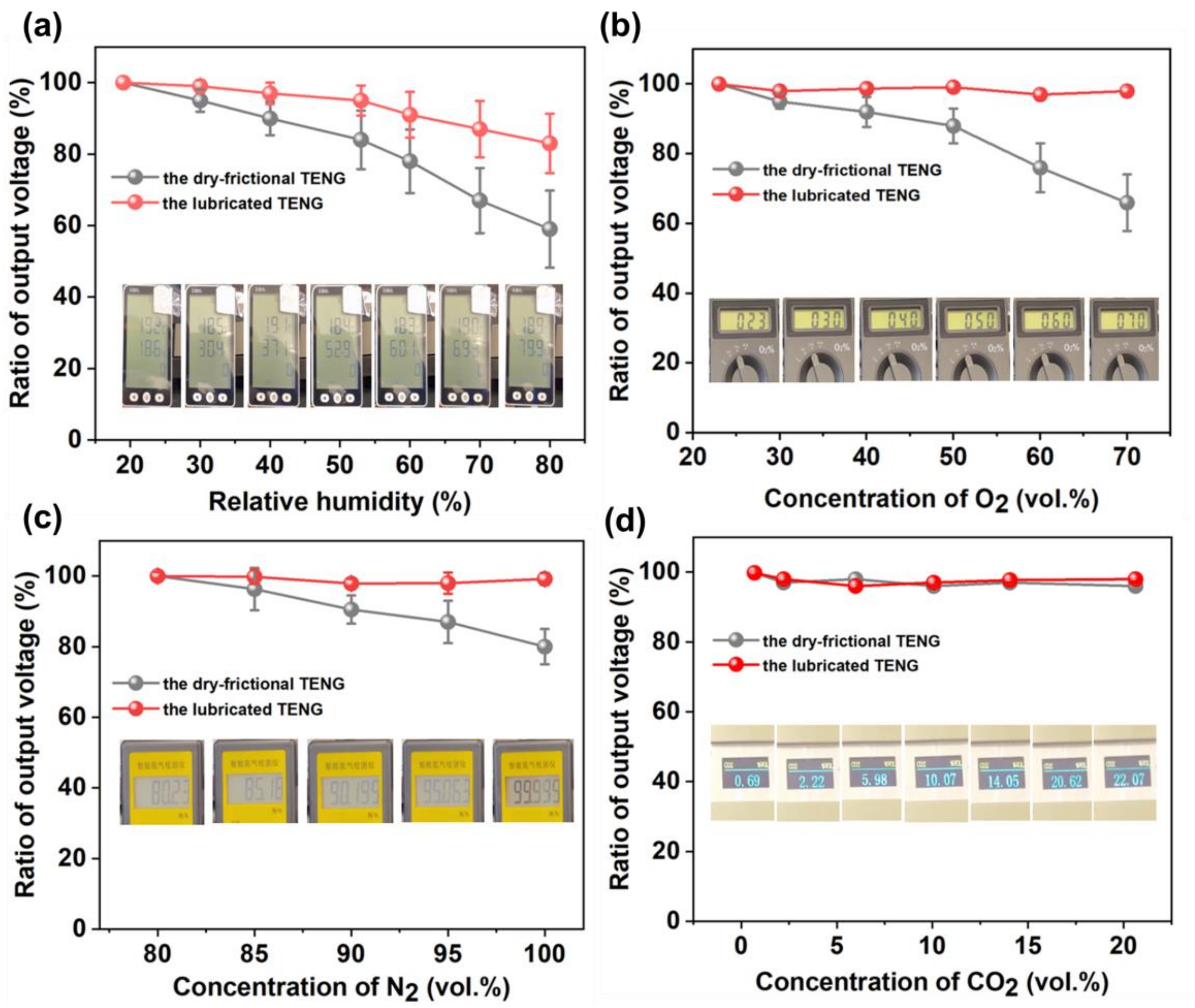

Figure S41 The influence of humidity and gas in air on the electric performance of the TENG with the lubrication of hexadecane after running-in with DMSO in contrast to the TENG under dry friction, relative humidity (a), O_2 (b), N_2 (c), and CO_2 (d).

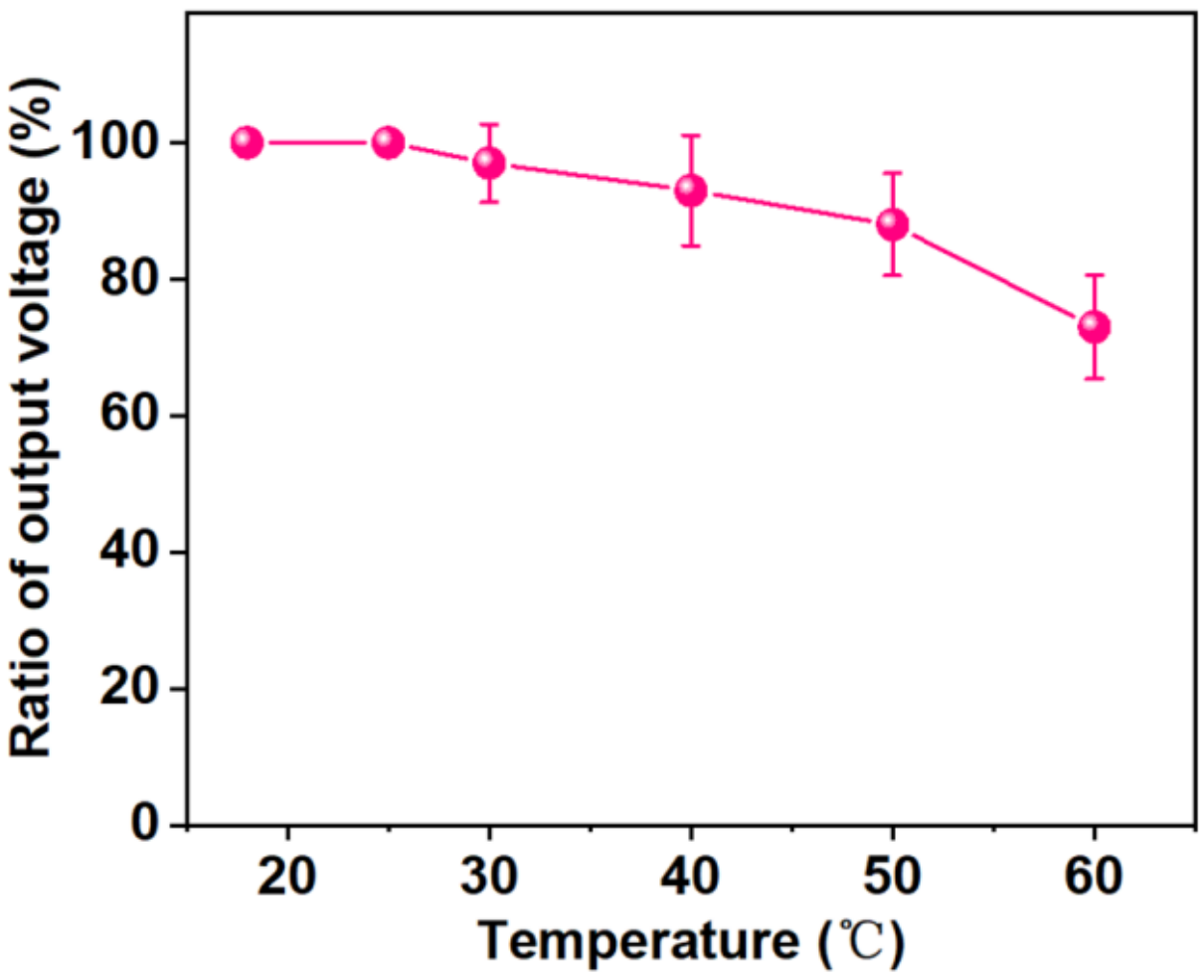

Figure S42 The influence of ambient temperature on the electric performance of the TENG with the lubrication of hexadecane after running-in with DMSO at the atmosphere (relative humidity ~20%RH).

2. Insufficient Evidence for Superhigh Charge Density and In-Running Modification: The reported "superhigh" charge densities and in-running modification phenomena require stronger justification. The authors suggest that ion release (e.g., from $BMImBF_4$) may shield the surface, but no direct measurements support this. Detailed electrochemical and spectroscopic evidence (e.g., ion concentration, interfacial ion accumulation) is needed to validate this hypothesis.

2. Response: Thanks so much for your valuable comments. In this study, there are two steps, i.e., the running-in process and the triboelectric period, for achieving high charge density. The electric outputs are very low through using the $BMImBF_4$, whether as the triboelectric lubricant (Fig. 2b) or as the running-in dissolvent (Fig. 2c). It has been widely identified in previous works[3, 12, 13] that the released ions from liquids could adsorb on the electric surface and shield the electrostatic effect, which results in a lower electric output. Accordingly, it is important to characterize the ion adsorption and accumulation on the electric surface. According to XPS analysis on the electric surface of a cleaned PI after running-in (Supplementary Fig. S5 and Table R1), there are large atomic concentrations of N, O, B, and F from $BMImBF_4$ on the running-in surface. TOF-SIMS was further performed to determine the interfacial accumulation of

$BMImBF_4$ components shown in Supplementary Fig. S6-7. A much higher concentration of $BMImBF_4$-related anions, such as B^-, F^-, and BF_4^-, was detected in the running-in PI surface (Supplementary Fig. S6). There are also obvious concentrations of released cations, e.g., $C_4H_9N_2^+$, $C_8H_{15}N_2^+$, etc., appearing on the running-in PI according to the mass spectra and 2D distribution images (Supplementary Fig. S7 and Fig. S8). Thus, the ions or related components from proton/ion dissolvents can strongly adsorb onto the electric surface, resulting in the shielding effect[3, 12, 13].

Table R1 Atomic concentration of the PI surfaces (at.%)

	**C1s**	**N1s**	**O1s**	**B1s**	**F1s**	**Al2p**	**S2p**
**Running-in with $BMImBF_4$**	51.32	7.62	18.58	5.89	16.58	0.00	0.00
**Virginal PI**	84.46	1.37	13.96	0.00	0.00	0.06	0.15

Changes as follows:

Page 6

For example, the electric outputs are very low through using the $BMImBF_4$, whether as the triboelectric lubricant (Fig. 2b) or as the running-in dissolvent (Fig. 2c). It has been widely identified in previous works[3, 12, 13] that the released ions from liquids could adsorb on the electric surface and shield the electrostatic effect, which results in a lower electric output. According to X-ray Photoelectron Spectroscopy (XPS) analysis on the electric surface of a cleaned PI after running-in (Supplementary Fig. S5), there are large atomic concentrations of N, O, B, and F from $BMImBF_4$ on the running-in surface. Time-of-flight secondary ion mass spectrometry (TOF-SIMS) was further performed to determine the interfacial accumulation of $BMImBF_4$ components (Supplementary Fig. S5, Fig. S6, and Fig. S7). A much higher concentration of $BMImBF_4$-related anions, such as B^-, F^-, and BF_4^-, was detected in the running-in PI surface (Supplementary Fig. S6). There are also obvious concentrations of released cations, e.g., $C_4H_9N_2^+$, $C_8H_{15}N_2^{+,}$ etc., appearing on the running-in PI according to the mass spectra and 2D distribution images (Supplementary Fig. S7 and Fig. S8).

Accordingly, it is probably because the released ions from these proton/ion dissolvents, e.g., $BMImBF_4$, are difficult to remove from the sliding interface, thus shielding the contact surface during the triboelectric period.

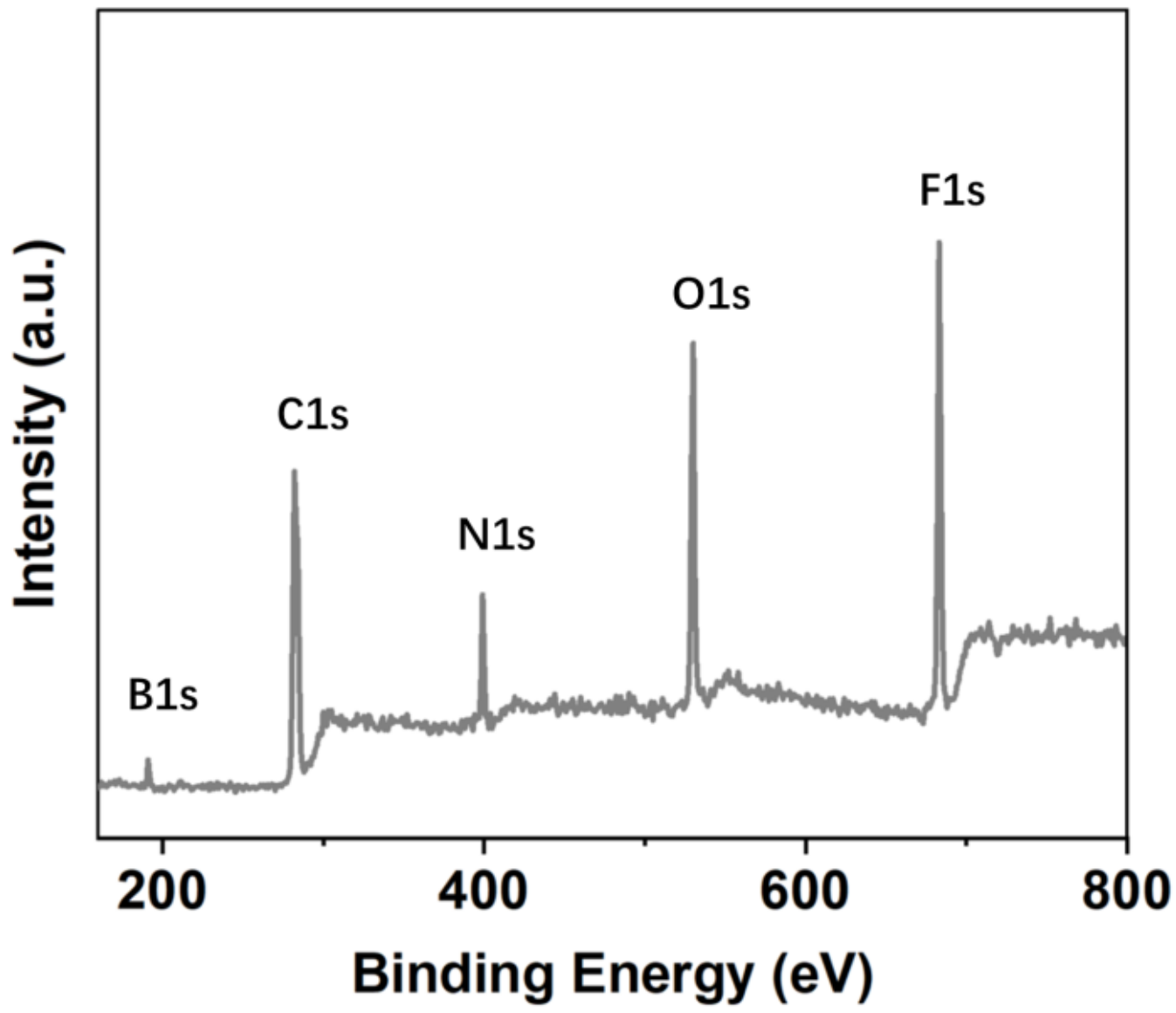

Figure S5 XPS survey spectra of the PI surfaces after running-in with $BMImBF_4$.

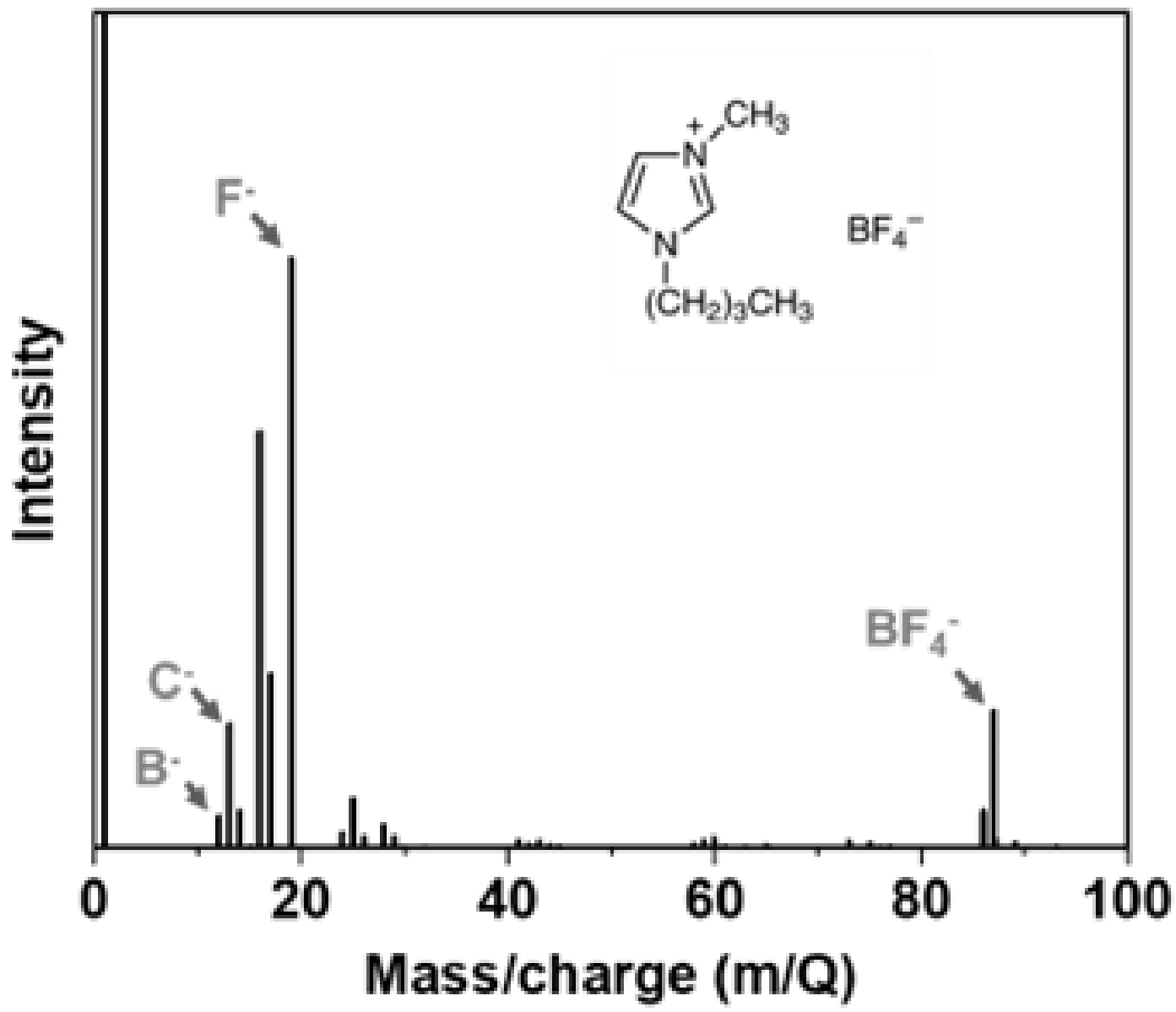

Figure S6 TOF-SIMS mass spectra of the anion on the PI surface after running-in process with $BMImBF_4$, inset showing the molecular structure of $BMImBF_4$.

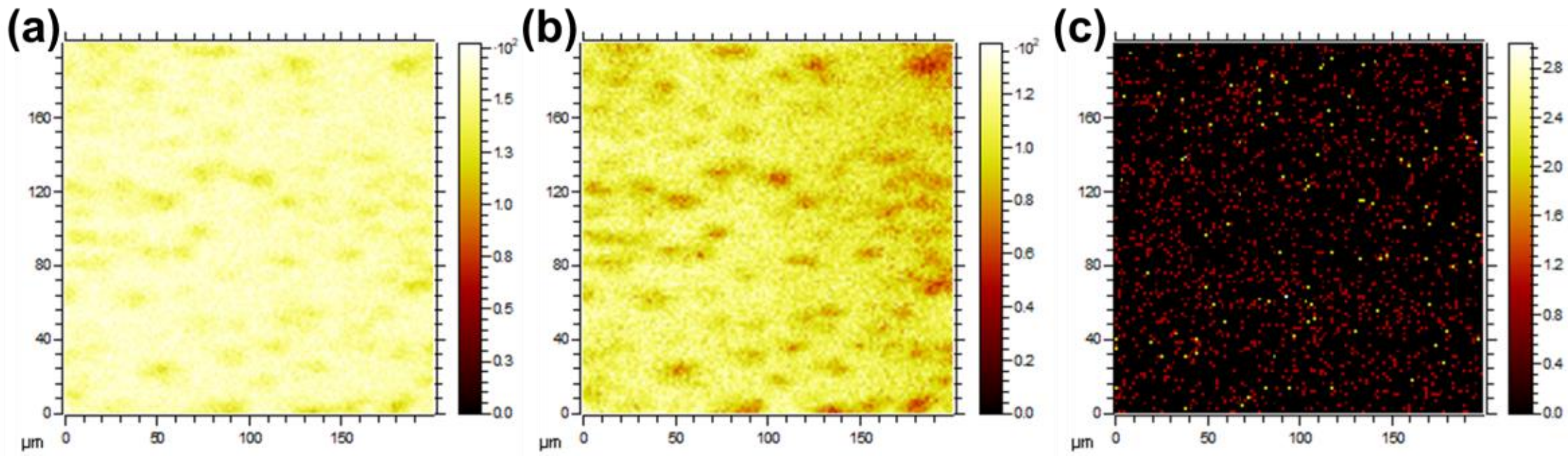

Figure S7 2D anion distribution images derived from the PI surface after running-in process with $BMImBF_4$: F^- (a), BF_4^- (b), and B^- (c).

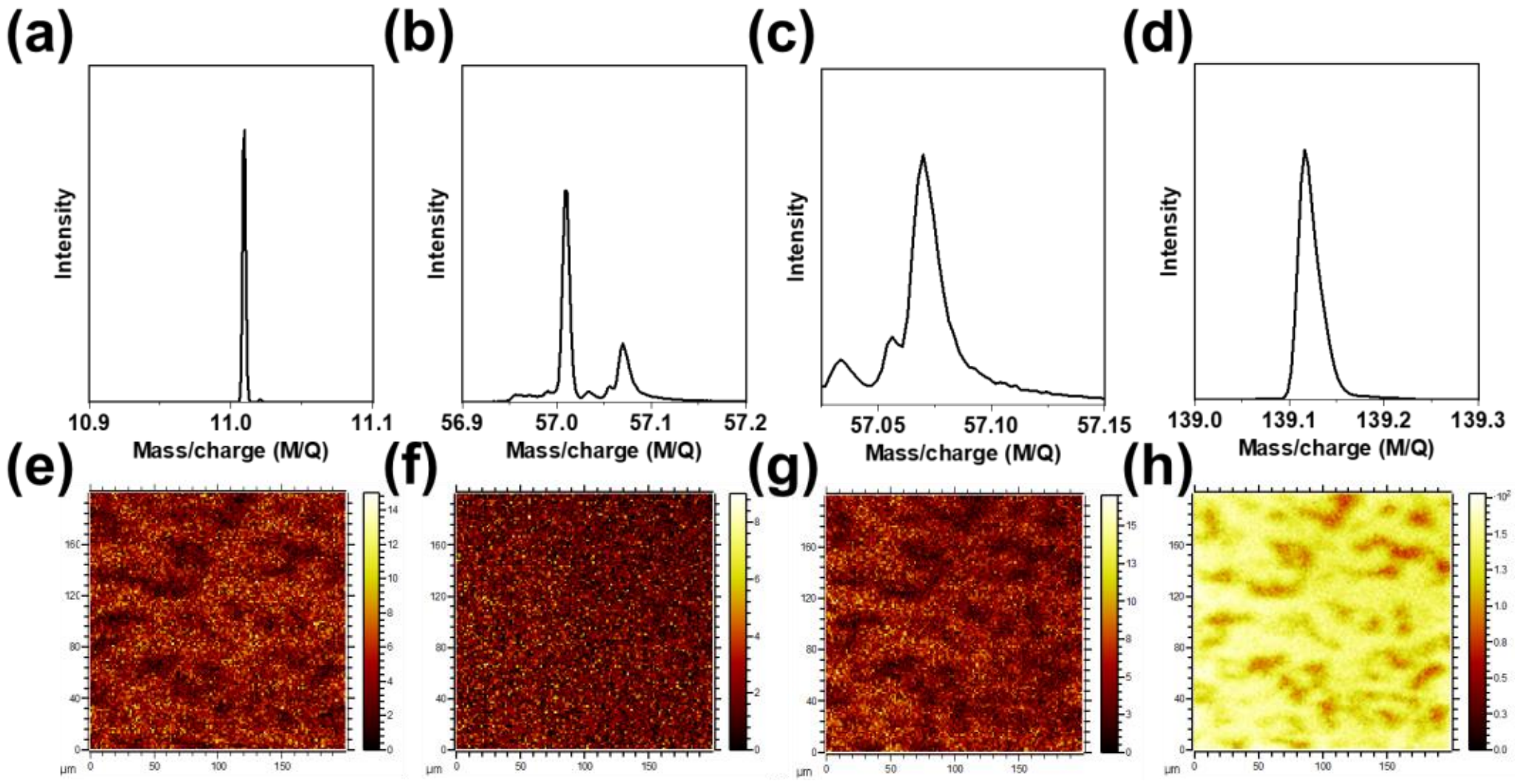

Figure S8 The TOF-SIMS mass spectra and 2D distribution images of the cation on the PI surface after running-in process with $BMImBF_4$: B^+ (a, e), $C_4H_9^+$ (b, f), $C_4H_9N_2^+$ (c, g), $C_8H_{15}N_2^+$ (d, h).

3. Surface Potential Analysis Missing:

Since surface potential critically governs TENG output, both bulk and localized surface potential distributions should be analyzed (e.g., KPFM, surface voltmeter mapping). These results would provide direct insight into the impact of surface modification on device performance.

3. Response: Thanks so much for your valuable comments. Accordingly, the surface potential of the PI surface using KPFM is carried out before running-in and after that for different cycles in the revised manuscript. Shown in Fig. 5b-d, the surface potential

changes obviously after the running-in process. From the extracted pixel distribution (Fig. 5b) with respect to the surface potential value, it can be seen that the change occurred after the first cycle, and there is a significant change in the surface potential after running-in for 100 cycles. Thus, the surface potential of the running-in region significantly dropped. Accordingly, it is evident from the KPFM measurements that the proposed running-in process leads to a much lower surface potential, which is correlated with a higher output in the TENG. It is because the reduction in surface potential was due to the transfer of charges from the AFM tip to the PI surface[14]. Thus, the more running-in cycles, the easier accepting charges on the surface, indicating that the running-in PI surface after more cycles has a stronger electron-withdrawing ability.

In contrast, the virgin PI surface exhibits a relatively high potential on the surface. This observation aligns with our findings that the ultrahigh charge density is able to be achieved with one running-in cycle. After 100 cycles of running-in, the triboelectric polarity of the PI changes to much more negative. It is because the scission and rearrangement of molecular chains occur quickly through the running-in optimization. Accordingly, the surface potential via KPFM measurements can successfully identify the change in the chemical structure of PI surfaces.

Apart from the surface potential, the dielectric analysis of the electric materials is also a good way to explain triboelectrification. Scanning force microscopy based on capacitance and electrostatic force is used for measuring the dielectric properties of insulating films at the nanoscale[15-18]. As shown in the following Figure R1, the detection principle involves defining the phase change of the probe under actual vibration state, in contrast to the free vibration phase[19, 20]. In EFM (Electric Force Microscopy), the relationship between the probe vibration phase and the force gradient can be expressed by formula (1) [16].

$$\tan(\varphi) \propto -\frac{Q}{K}F' \quad (1)$$

where φ is the vibration phase of the probe, K is the spring constant of the probe, Q is the quality factor, and F' is the force gradient acting on the probe. Under identical testing conditions and with the same probe, K and Q can be regarded as constants. The

long-range force acting on the probe is dominated by the capacitive force between the probe and the sample, which can be expressed by formulas (2) and (3)[21].

$$F = \frac{1}{2}\frac{\partial C}{\partial Z}\left(V_{tip} - V_S\right)^2 \quad (2)$$

$$F' = \frac{1}{2}\frac{\partial^2 C}{\partial Z^2}\left(V_{tip} - V_S\right)^2 \quad (3)$$

where V_{tip} is the probe voltage, V_S is the surface potential of the sample, Z is the distance between the probe and sample, the lifting height, and C is the system capacitance composed of the probe and sample. In addition to voltage factors, the force gradient F' is mainly determined by the probe sample capacitance structure and sample dielectric constant. The relationship between the vibration phase of the probe and voltage in EFM mode can be expressed as formula (4):

$$\tan\varphi \propto A\left(V_{tip} - V_S\right)^2, A = -\frac{Q}{K}\frac{1}{2}\frac{\partial^2 C}{\partial Z^2} \quad (4)$$

It means that there is a quadratic function relationship between tan ϕ and the electric potential difference (V_{tip}-V_s), in which the factor A is the coefficient of the quadratic term. The factor A is related to the capacitance between the probe and the sample according to the formula (4). According to the results of various probe-sample capacitance models[19, 20], the probe-sample capacitance increases with the increase of the dielectric constant ε. Therefore, the absolute value of the factor A has a positive relationship with the dielectric constant ε of the sample. With the remaining parameters basically fixed, the local dielectric properties can be analysed by comparing the slope of the phase change by regulating the bias voltage applied to the probe.

Therefore, EFM can be used to detect the changes in local dielectric properties of PIs in this study. For example, the phase evolution ~ φ of the running-in PI under different tip voltages compared with the virginal PI is shown in Supplementary Fig. S31. The degree of phase change increases with the increase of the tip voltage, in which the phase change of the running-in PI has a greater extent than that of the virginal PI. Furthermore, the surface potential of the PI sample is obtained through the KPFM analysis (Fig. 5b-c). According to the formula (4), the factor A can be thus obtained through establishing the quadratic function relationship between the tan φ and the electric potential difference (V_{tip}-V_s) shown in Supplementary Fig. S32. Accordingly,

the running-in PI has a larger absolute value of the factor *A* (~0.00112) than that of the virginal PI (~0.00101), so the local dielectric property of the PI after running-in is enhanced. The analysis of the dielectric property is based on a qualitative method, and more quantitative analysis will be carried out in a future study.

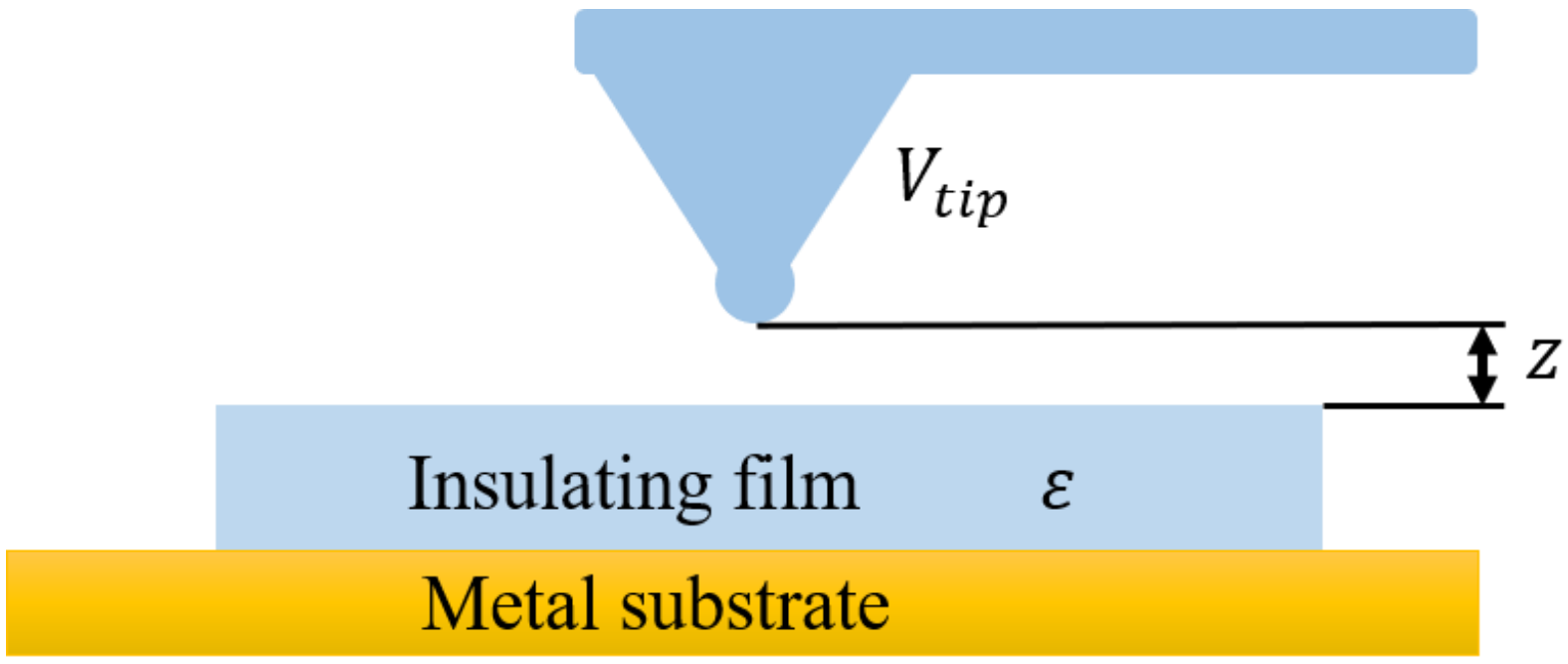

Fig. R1 Schematic diagram of dielectric analysis EFM detection.

Changes as follow:

Page 17

Accordingly, the surface potential of the PI surface using Kelvin Probe Force Microscopy (KPFM) is carried out (Fig. 5b-d), and it is obtained that the surface potential changes obviously after the running-in process. From the extracted pixel distribution (Fig. 5b) with respect to the surface potential value, the change occurs after the first cycle, and there is a significant change in the surface potential after running-in for 100 cycles (Fig. 5c). It is evident from the KPFM results that the proposed running-in process leads to a much lower surface potential, which is correlated with a higher output in the TENG. A lower surface potential, implying a more negative potential, indicates a stronger electron-withdrawing ability[15, 16]. This observation aligns with our findings that the ultrahigh charge density is able to be achieved with one running-in cycle (Fig. 5d). After 100 cycles of running-in, the triboelectric polarity of the PI changes to much more negative (surface potential ~ -6.98 V). It is because the scission and rearrangement of molecular chains occur quickly through the running-in

optimization. Accordingly, the surface potential via KPFM can successfully identify the change in the chemical structure of PI surfaces.

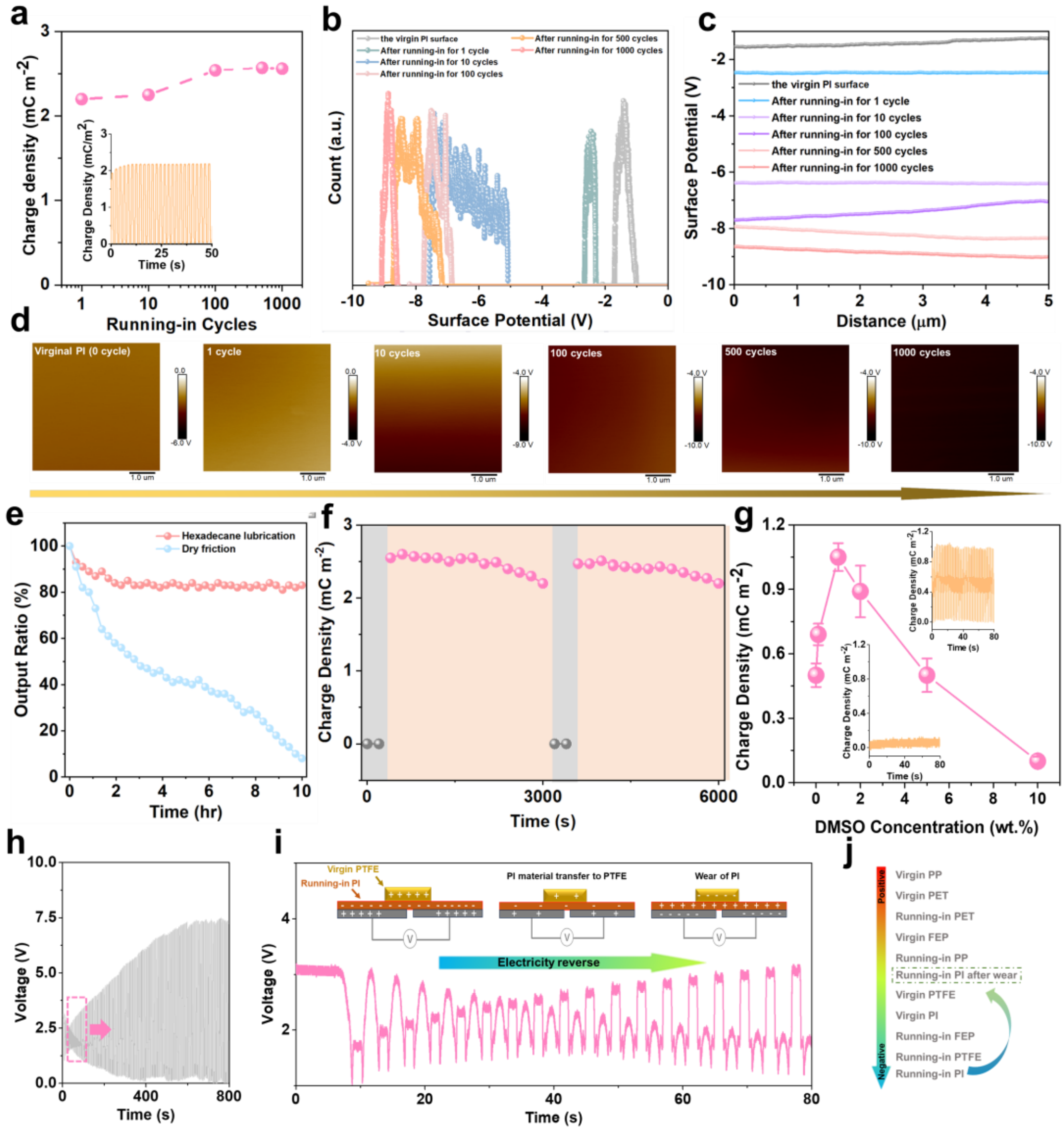

Fig. 5 Super-fast DMSO-assisted running-in strategies towards triboelectric enhancement and reversal effect. a Relationship between the running-in cycle and the triboelectric output. **b** Relative pixel distribution with respect to the surface potential value after running-in with DMSO. **c** Extracted line scan profile with respect to the surface potential value after running-in with DMSO. **d** Surface potential profile of the PI surfaces after running-in with DMSO. **e** Electric outputs of the hexadecane-lubricated TENG after running-in with DMSO compared with the dry-friction TENG in the long-term test. **f** Charge density of the running-in PI with hexadecane lubrication after intermittent running-in processes. **g** Charge density of the virgin PI lubricated by

the mixture of hexadecane and DMSO. **h** and **i** Triboelectric output of the friction pairs between the running-in PI with virgin PTFE, the inset in Fig. 5i demonstrating the electric reversal mechanism of the friction pairs. **j** Triboelectric series of engineering dielectric materials for lubricated sliding modes considering the running-in optimization.

Page 18

In addition, the dielectric analysis of the electric materials is also a good way to explain triboelectrification. Scanning force microscopy based on capacitance and electrostatic force is used for measuring the dielectric properties of insulating films at the nanoscale[5-8]. The detection principle involves defining the phase change of the probe under actual vibration state, in contrast to the free vibration phase in Electric Force Microscopy (EFM)[9, 10]. The relationship between the phase of probe vibration and voltage in EFM mode can be expressed as Eq. (4) (more details in **Methods**):

$$\tan\varphi \propto A\left(V_{tip}-V_S\right)^2,\ A=-\frac{Q}{K}\frac{1}{2}\frac{\partial^2 C}{\partial Z^2}\quad (4)$$

where φ is the phase of probe vibration, V_{tip} is the probe voltage, V_S is the surface potential of the sample, Z is the distance between the probe and sample, the lifting height, and C is the system capacitance composed of the probe and sample. It means that there is a quadratic function relationship between tan φ and the electric potential difference (V_{tip}-V_s), in which the factor A is the coefficient of the quadratic term. The factor A is related to the capacitance between the probe and the sample according to the Eq. (4). According to the results of various probe-sample capacitance models[9, 10], the probe-sample capacitance increases with the increase of the dielectric constant ε, so the absolute value of the factor A has a positive relationship with the dielectric constant ε of the sample. Therefore, EFM can be used to detect the changes in local dielectric properties of PIs. For example, the phase evolution ~ φ of the running-in PI under different tip voltages compared with the virginal PI is shown in Supplementary Fig. S31. Through establishing the quadratic function relationship between the tan φ and the electric potential difference (V_{tip}-V_s) (Supplementary Fig. S32), the running-in PI has a

larger absolute value of the factor *A* (~0.00112) than that of the virginal PI (~0.00101), so the local dielectric property of the PI is enhanced after running-in process.

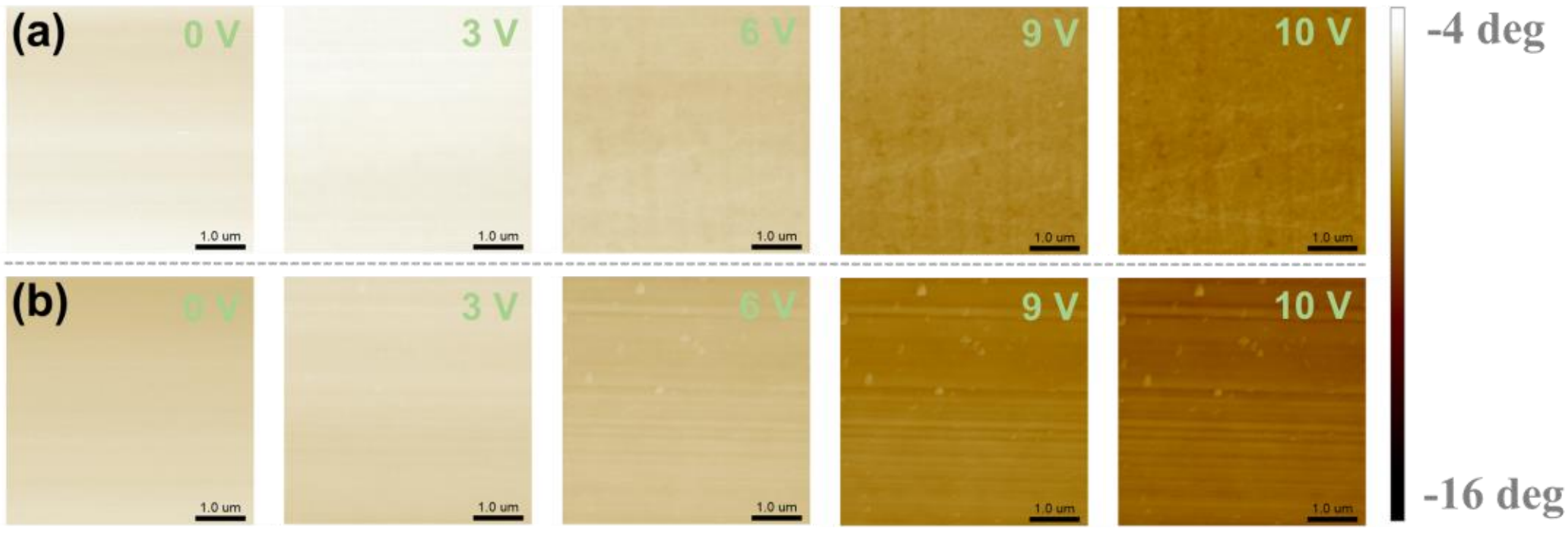

Figure S31 Electrical phase images via EFM for PIs after running-in for 0 cycle (a) and 100 cycles (b).

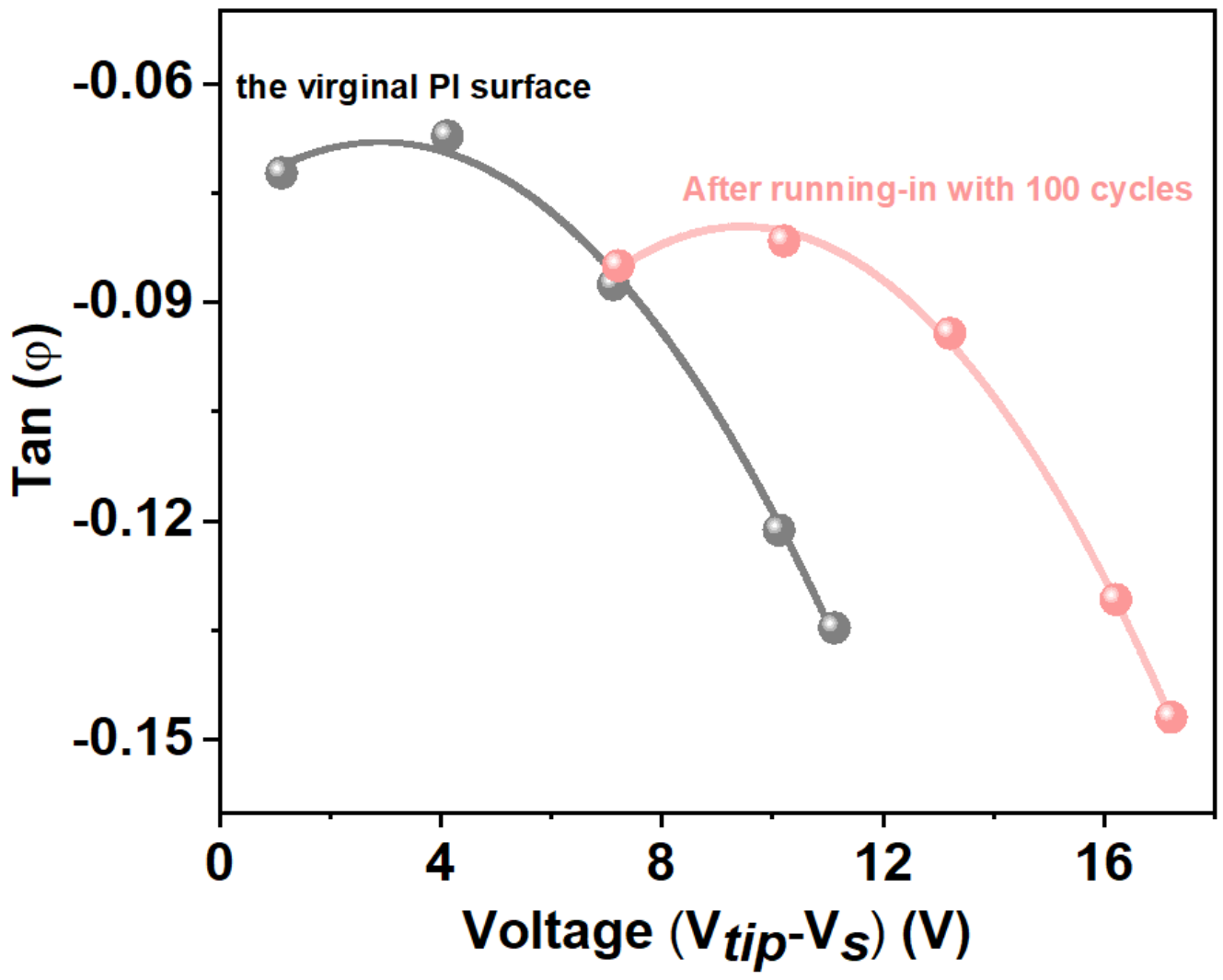

Figure S32 Characteristic curve of the tan φ of the PI as a function of the voltage between the tip and the sample surface (V_{tip}-V_s).

4. Lack of Comparative Performance Metrics:

A detailed comparison of voltage, current, power density, and internal resistance before and after treatment is necessary, especially in relation to standard TENG configurations. Current values appear lower than in comparable literature, despite higher power densities. The authors should clarify whether this discrepancy results from changes in internal resistance or other parameters.

4. Response: Thanks so much for your valuable comments. The TENG fabricated in this study is based on a typical sliding-mode. It should be noted that the structure of the TENG is almost the same as that in recent works[3, 22, 23] under the dry sliding/friction condition. Thus, the TENG operating under dry friction can be used as a standard configuration for comparison. The charge density of the TENG is greatly boosted through optimizing the running-in process. The output performances in terms of voltage and current are also added in the same way in the revised manuscript (Supplementary Fig. S2) and are shown in the following Fig. R2. It can be seen that the change trends of voltage and current are the same as the charge output for the DMSO-treated TENG and the compared samples. The voltage and current of typical dry-frictional TENG are as low as 37 V and 0.12 μA, respectively. Whereas, the electric output will be significantly improved through adding lubricants in the electric interfaces, which is confirmed from the result shown in Supplementary Fig. S2. Through the optimized running-in with DMSO, the voltage and current of the TENG increase to 680 V and 3.3 μA (Supplementary Fig. S2 and Fig. R2).

In addition, it is found that the TENG has a high internal resistance ~ GΩ through matching impedance analysis shown in Supplementary Fig. S10 and Fig. R2. The main reasons for high internal resistance are the dielectric properties of the triboelectric materials and the capacitance limitations of the device. The impedance of the TENG (Z) is expressed as $Z = \frac{1}{2\pi f C_s}$. Among them, Cs and f are the inherent capacitance and operation frequency of TENGs, respectively. The TENG commonly uses typical dielectric PIs with relatively low dielectric constants. The PIs-based TENGs have a very low capacitance ~ Cs, which ultimately leads to the extremely high internal resistance of the TENG[24]. Further, the internal resistance of the lubricated TENGs is higher than that dry-frictional TENG, which is because the hexadecane lubricant is nonpolar and insulating[3]. At the same time, the peak power density under various external loads of the PI-based TENG is obtained shown in Supplementary Fig. S10. The TENG lubricated by hexadecane after running-in with DMSO reaches a maximum of 18.5 W m^{-2} Hz^{-1} when the external circuit load is about 5 GΩ, which is much higher

than that of the hexadecane-lubricated TENG without a running-in process. The dry-frictional TENG as the standard sample has the lowest power density of 0.2 W m^{-2} Hz^{-1} at the external circuit load of 2 GΩ.

Lower current values appearing than in comparable literature are mainly due to the high internal resistance. The reason for the high internal resistance has been discussed above. The change in the device operation, i.e., sliding frequency, can directly affect the electric output of TENGs according to the second part of the equation from the first principle theory[25]. As shown in Supplementary Fig. S43, with the increase of sliding frequency, the output current increases obviously, which means the value of output current is proportional to the frequency, which aligns with previous works[23, 26].

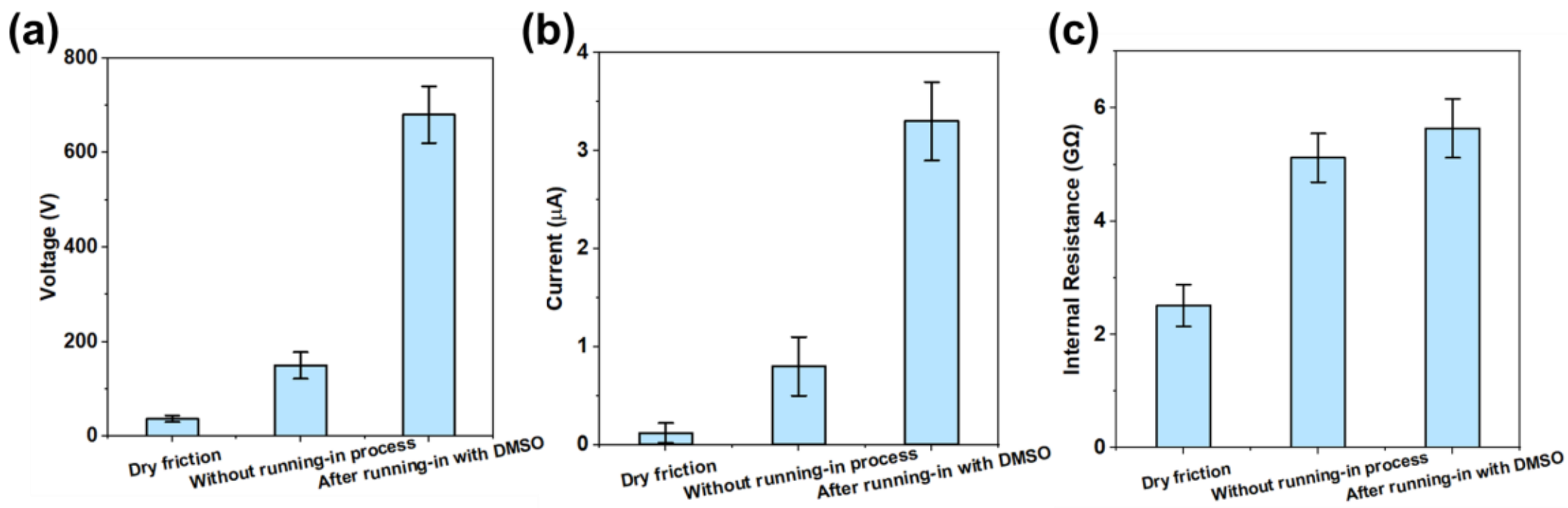

Figure R2 The voltage (a), current (b), and internal resistance (c) of the hexadecane-lubricated TENG (with or without running-in process), compared with dry-friction TENG.

Changes as follows:

Page 5

In addition, the PI-based TENG has much higher voltage and current than those without running-in treatment (Supplementary Fig. S2). The output voltage and current of the TENG lubricated by hexadecane after running-in with DMSO are about 680 V and 3.3 μA, respectively. In addition, the influence of lubricants during the triboelectric period is shown in Fig. 2b and Supplementary Fig. S2c-d.

Page 6

It is found that dissolvents, such as DMSO, DMA, DMF, etc., during the running-in process can boost the electric output of PI (Fig. 2c and Supplementary Fig. S2e-f)

Page 8

Further, the peak current and peak power density of the TENG are obtained under different external load resistance (Supplementary Fig. S9 and Fig. S10). According to the normalized peak power density as an essential metric[23, 27-29], the peak output power density of the TENG reaches a maximum of 18.5 W m^{-2} Hz^{-1} at an external circuit load of 5 GΩ, which is also higher than other works (Supplementary Fig. S11), representing the strong advantage of the running-in optimization strategy in achieving high-performance TENGs. In contrast, the dry-frictional TENG as the standard sample has the lowest power density of 0.2 W m^{-2} Hz^{-1} at the external circuit load of 2 GΩ (Supplementary Fig. S10). It is found that the internal resistance of the TENG is high, which is similar to other work[22]. The main reason is that PI-based TENGs have a very low capacitance, which ultimately leads to a high internal resistance of the TENG[24]. Further, the internal resistance of the lubricated TENGs is higher than that of the dry-frictional TENG because the hexadecane lubricant is nonpolar and insulating[3].

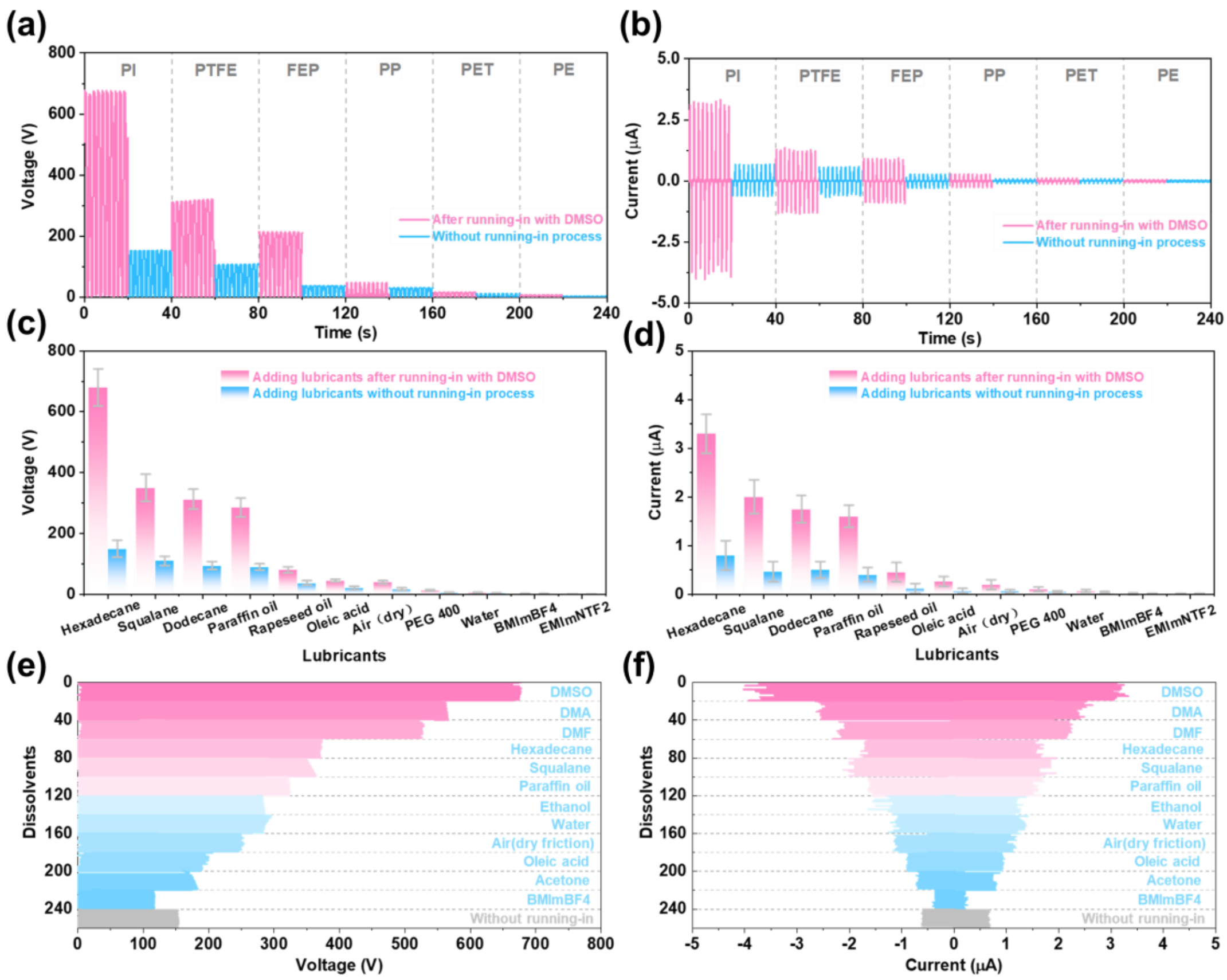

Figure S2 Output voltage (a) and current (b) of different engineering tribo-materials lubricated by hexadecane after running-in with DMSO. Output voltage (c) and current (d) of the engineering PI lubricated by different lubricants after running-in with DMSO. Output voltage (e) and current (f) of the engineering PI lubricated by hexadecane after running-in with different dissolvents.

On the other hand, the change in the device operation, i.e., sliding frequency, can directly affect the electric output of TENGs according to the second part of the equation from the first principle theory[25]. With the increase of sliding frequency, the output current increases obviously (Supplementary Fig. S43a), meaning the value of output current is proportional to the frequency, which aligns with previous works[23, 26]. However, the electric interface between the Al tribolayer and the PI surface will be further separated by the lubricant because of the hydrodynamic effect in terms of liquid lubrication theory[30]. Thus, the output charge decreases slightly at a high sliding

frequency (Supplementary Fig. S43b).

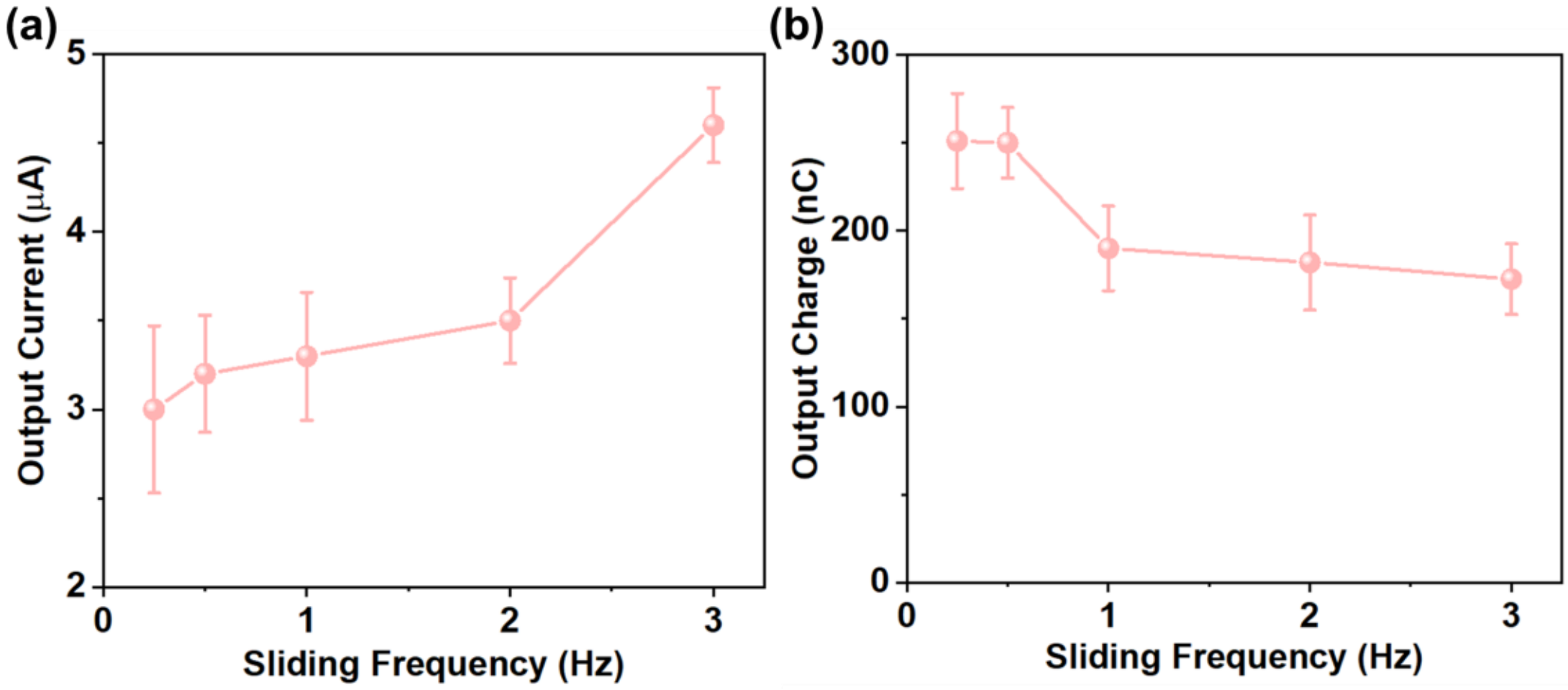

Figure S43 Output current (a) and charge (b) of the TENG lubricated by hexadecane after running-in with DMSO as a function of sliding frequency.

5. Internal Resistance and Calibration Methodology:

The reported intrinsic charge density (2.5 mC m^{-2}) after DMSO-assisted running-in lacks methodological transparency. There is no clear reference to standard calibration techniques (e.g., air breakdown suppression, guard-ring electrode geometries). Without proper calibration, fair comparison with other TENGs becomes unreliable.

5 Response: Thanks so much for your valuable comments. The TENG fabricated in this study is based on a typical sliding-mode. It should be noted that the structure of the TENG is almost the same as that in recent works[3, 4, 22] that consider the effect of interface lubrication and material modification on electric outputs. In detail, the TENG is fabricated through the composition of two plates using flat polymethyl methacrylate (PMMA) boards as the supporting substrate. The upper and lower plates of the TENG were Al electrodes attached to the PMMA (Fig. 1a and Methods). This study mainly focuses on the new triboelectricity-enhancing method through optimizing the running-in process rather than geometry designs. It is believed that the method proposed in this study is simple, effective, and practical.

Air breakdown suppression can be effectively obtained by adding proper lubricants at the electric interface[4]. In this study, during the triboelectric period, it is also found that the non-polar lubricants strongly enhance the electric output of TENGs

(Fig. 1b and Fig. 2). For fair comparison, all the running-in samples and virgin samples have the same lubrication condition during the triboelectric period, e.g., hexadecane (Fig. 2a), which can identify the reliability of the contribution of the running-in process. Apart from PI, the standard samples of PTFE and FEP are also used as a comparison calibration. It is well known that they have strong electron-capture capabilities. However, it is found that without running-in optimization, the charge densities of PTFE and FEP are lower than 0.5 mC m^{-2} even with the enhancement of hexadecane lubrication, which has no competition to previous works (Fig. 2d). Through the running-in process, the electric outputs of PTFE, FEP, and PI are all improved significantly.

6. Device Longevity and Wear:

Long-term stability is not addressed. A durability study (e.g., >10^4 cycles) is needed to demonstrate whether performance is stable or degrades over time. It would also be useful to report any physical degradation (e.g., thinning of the plastic film) post-cycling.

6 Response: Thanks so much for your valuable comments. The durability of TENG is very important for its practical applications. Accordingly, the long-term stability of the TENG is tested for 10 hours (~18000 cycles) in the revised manuscript compared with the TENG under dry friction shown in Fig. 5e. It is obvious that the output ratio of the TENG under dry friction slowly declines to 50% and 8% after 3 hours and 10 hours, respectively. Although the electric output of the TENG decreases gradually at the beginning of the triboelectric period (~1 hour), the value of the output ratio is very stable with a high charge density ~ more than 2 mC m^{-2} after 10 hours. Further, the output value of the TENG can increase to be larger within a fast running-in process of 200 s (100 cycles) (Fig. 5f). Thus, it is obvious that the DMSO-assisted running-in process together with hexadecane lubrication has good advantages for boosting the triboelectric output and durability of TENGs.

Apart from the long-term stability test of electric outputs, the tribological properties related to frictional loss and material degradation are also utilized to characterize the durability of the TENGs. As shown in Supplementary Fig. S44a, the

coefficient of friction of the TENG under dry friction is very unstable with a severe fluctuation at the beginning of sliding, and it slowly increases at the late stage of the test. Whereas, the TENG with the hexadecane lubrication has a much lower and more stable value of the coefficient of friction. In addition, the wear depth of the TENGs related to the material loss is much lower for the case of hexadecane-lubricated TENG than that without lubrication. After the long-term test, the TENG without lubrication shows a rough surface due to severe wear (Supplementary Fig. S44b). For the case of hexadecane-lubricated TENG, the PI surface shows a smooth surface with very slight scratch grooves.

Changes as follow:

Page 20

The long-term stability of TENGs is very important for the practical application, which was examined shown in Fig. 5e. After more than 10 hours (~18 000 cycles), the lubricated TENG exhibits superior electric durability and maintains 86% output (>2 mC m^{-2}) which is still much higher than previous works (Fig. 2d). Although there is a little attenuation on the electric output, the charge density of the lubricated TENG significantly increases within a fast running-in process of 100 cycles (Fig. 5f). The high electric output of the TENG is attributed to the regulation of amide-containing chemical chains into “molecular brushes” that extend toward the contact surfaces. These outspread groups could be worn during triboelectrification, probably resulting in a decrease in the electric output. In contrast, the same TENG device retains only 9% electric output under dry friction in air. In addition, the coefficient of friction of the dry-frictional TENG is two times as much as that of the lubricated TENG (Supplementary Fig. S44a). The PI surface suffers severe wear after dry friction in contrast to the PI surface with the hexadecane lubrication (Supplementary Fig. S44b). Thus, the TENG with the hexadecane lubrication has good electric durability and tribological performance.

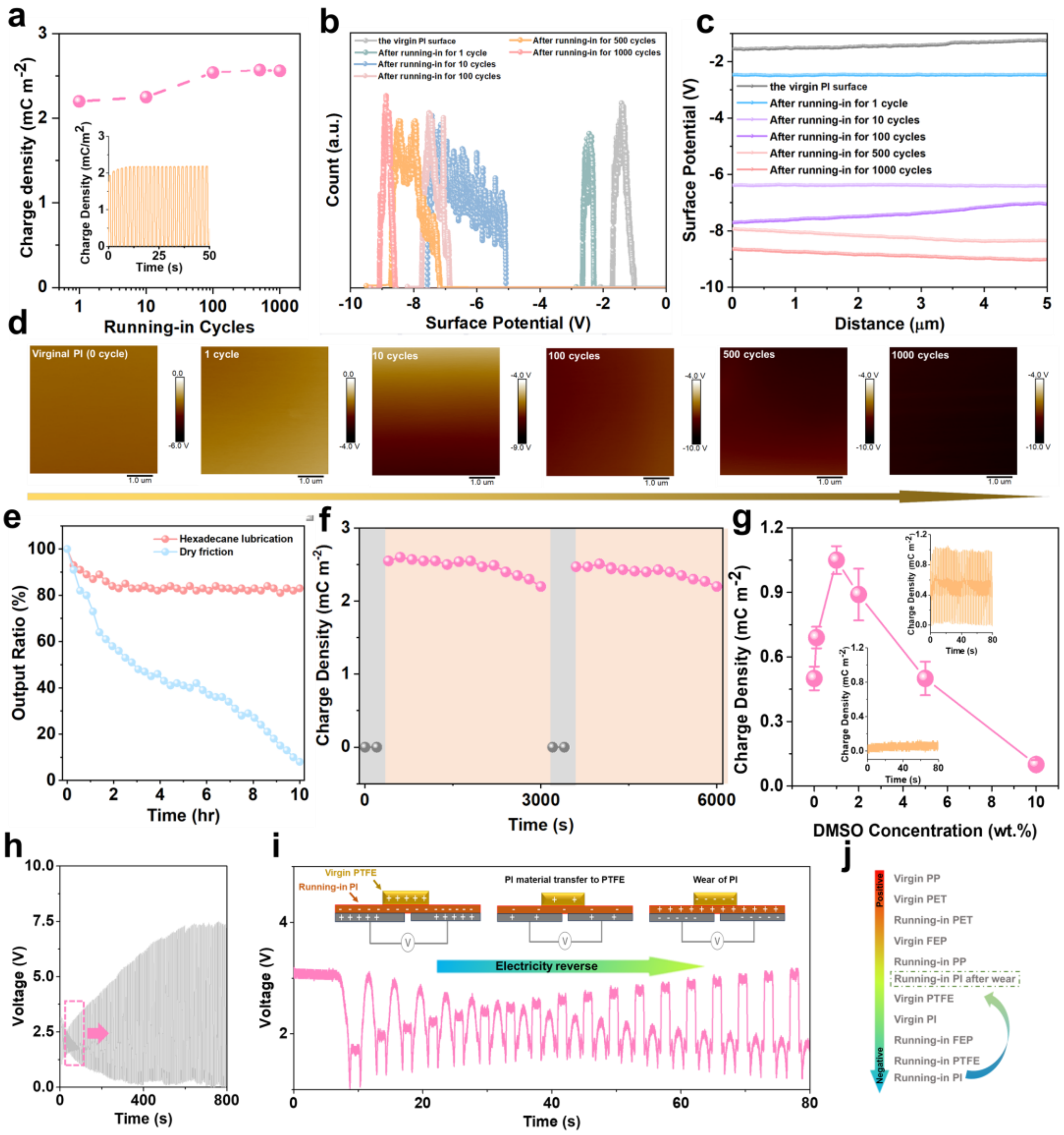

Fig. 5 Super-fast DMSO-assisted running-in strategies towards triboelectric enhancement and reversal effect. a Relationship between the running-in cycle and the triboelectric output. **b** Relative pixel distribution with respect to the surface potential value after running-in with DMSO. **c** Extracted line scan profile with respect to the surface potential value after running-in with DMSO. **d** Surface potential profile of the PI surfaces after running-in with DMSO. **e** Output ratio of the charge density for the hexadecane-lubricated TENG after running-in with DMSO compared with the dry-friction TENG in the long-term test. **f** Charge density of the running-in PI with hexadecane lubrication after intermittent running-in processes. **g** Charge density of the virgin PI lubricated by the mixture of hexadecane and DMSO. **h** and **i** Triboelectric output of the friction pairs between the running-in PI with virgin PTFE, the inset in Fig.

5i demonstrating the electric reversal mechanism of the friction pairs. **j** Triboelectric series of engineering dielectric materials for lubricated sliding modes considering the running-in optimization.

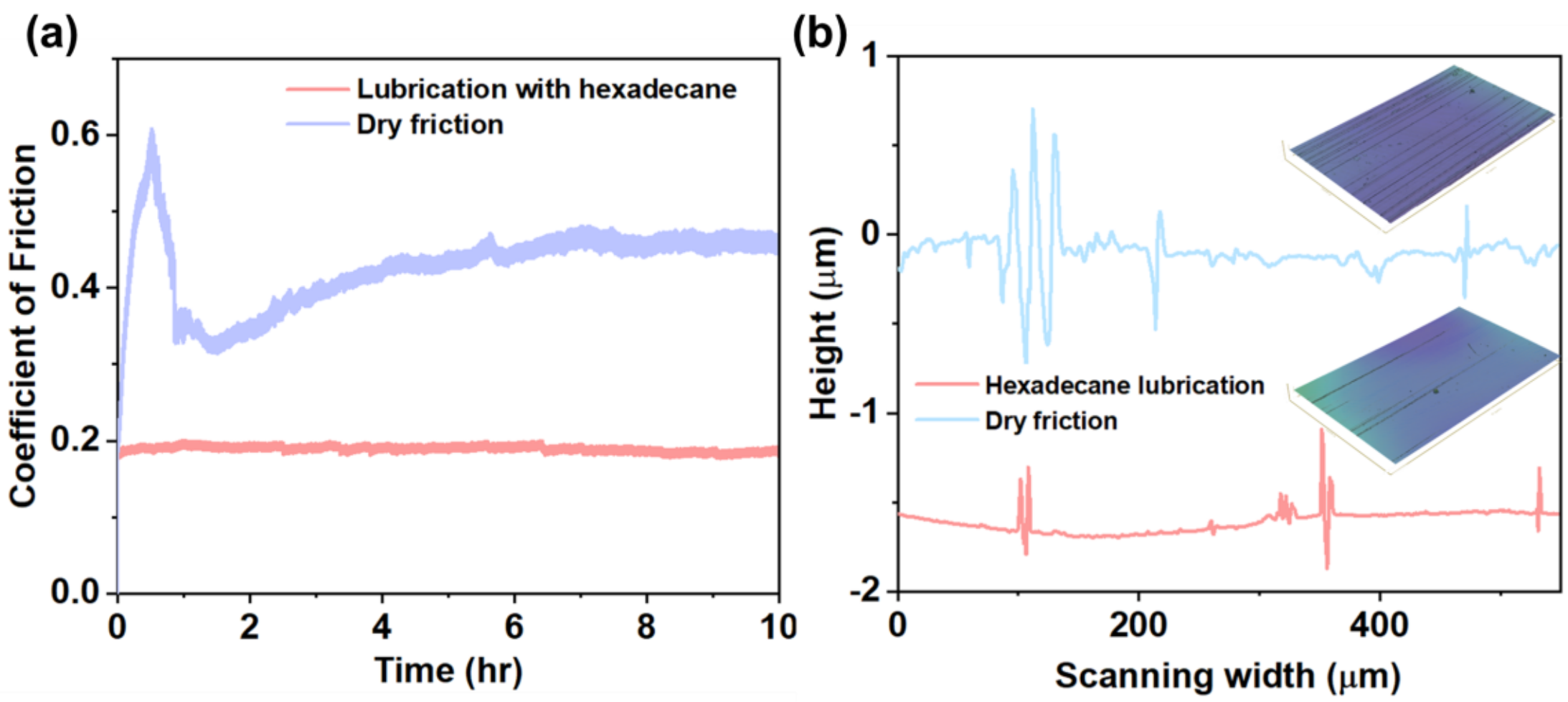

Figure S44 Coefficient of friction (a) and wear depth (b) of the hexadecane-lubricated TENG after running-in with DMSO compared with the dry-friction TENG in the long-term test, the inset in Fig. S44b showing the 3D morphologies after the long-term test.

7. Limitations of MD System Size:

The molecular dynamics simulations utilize short-chain PI (DP = 4, 25 chains), which likely exaggerates surface mobility and fragmentation. The authors should either increase chain length and system size or include coarse-grained simulations to verify that the results generalize to bulk-like behavior.

7. Response: Thank you for the valuable comment. We fully agree that system size and chain length may influence the apparent mobility and fragmentation behavior of polymer surfaces. Before deciding on the current model, we indeed attempted to enlarge the all-atom PI system (higher DP and more chains). However, because ReaxFF explicitly tracks bond breaking/formation, the computational cost increases nearly exponentially with both chain length and atom count. Even a moderate extension of the system size led to prohibitively long simulation times and insufficient sampling of reactive events, making such simulations impractical with available computational resources. We appreciate the reviewer’s suggestion to employ coarse-grained

simulations. However, coarse-grained approaches cannot describe the bond scission, radical formation, rearrangement, and subsequent generation of polar unsaturated groups that are central to this study. Since these interfacial chemistries are intrinsically reactive and require an explicit electronic-structure-based bonding representation, a coarse-grained model would not be able to reproduce the mechanistic details we aim to resolve.

Despite using relatively short chains (DP=4) as repeating units in PI, the current system is sufficiently large to capture the local chemical mechanisms of (i) friction-induced bond scission, (ii) generation of unsaturated functional groups, and (iii) solvent infiltration near reactive sites. These reactions occur within a few angstroms of the scission location and are governed by local environment rather than long-range polymer connectivity. Therefore, while longer chains may influence global mechanical response, the qualitative nature of the interfacial chemistry relevant to solvent adsorption is expected to remain unchanged. According to the molecular structure of PI, a model consisting of 4 polymerizations was also constructed for molecular dynamics simulation in recent works[31, 32]. We agree that system-size effects are an important direction for future work. We plan to explore multiscale strategies combining ReaxFF with higher-level coarse-grained models to evaluate bulk-level behavior, although this lies beyond the current study's scope.

8. Charge Accumulation and Interfacial Leakage:

The role of interfacial permittivity and conduction in charge generation must be rigorously examined. High apparent Q values may result from displacement or leakage currents through residual solvent films. Recommended tests include open-circuit decay, leakage I−V, frequency scaling, and blocking capacitor checks to eliminate non-triboelectric contributions.

8. Response: We sincerely appreciate the constructive recommendation, which allowed us to provide a more thorough discussion on the electrical characteristics of the TENG. After running-in with DMSO, superhigh electric output can be achieved through

lubricating with hexadecane (Fig. 2). Supplementary Fig. S39 shows the recorded charge decay of the TENG when the sliding process stopped. It is found that the decay dissipation is very low as similar to previous work[33]. After 2500 s, the residual charges on the surface are 99% of the original charges, which is because the charged surface is fully packaged by the hexadecane, so that the air breakdown is prevented successfully[34]. In addition, the dielectric leakage current is as low as 1 pA with a bias voltage from 200 V to 1000 V (Supplementary Fig. S40)[35]. It should be noted that the leakage current of the TENG after running-in is consistent with that of a virgin PI-based sample, which means the running-in process does not influence the charge decay and leakage.

Introducing an electron-blocking layer in the TENG can significantly affect the electric output[36]. In general, the electron blocking layer is fabricated at the interface between the dielectric tribo-material and an electrode[36-38]. In contrast, the TENG in this study is fabricated by the Al electrode and the PI directly without any blocking layers (Fig. 1), which is different from other works with blocking components[36-38]. In this study, during running-in with DMSO, the evolution of molecular chains occurs on the electric surface of PI, neither in the internal PI nor at the bottom of PI. That is, the strong electron-withdrawing capability is due to the molecular activation on the PI surface through running-in optimization. Further, the thickness of the activated molecular layer at the PI surface is lower than 10 nm (Fig. 3h-j) after running-in for 100 cycles. It is also found that the ultrahigh charge density is able to be achieved just with one running-in cycle (Fig. 5a). Such low-thickness structure is difficult to play an electron-trapping role in view of the blocking effect, because the thickness of the blocking effect is much higher (~100 nm) in the previous work[36].

The change in the device operation, i.e., sliding frequency, can directly affect the electric output of TENGs according to the second part of the equation from the first principle theory[25]. As shown in Supplementary Fig. S43a, with the increase of sliding frequency, the output current increases obviously, which means the value of output current is proportional to the frequency, which aligns with previous works[23, 26]. However, the electric interface between the Al tribolayer and the PI surface will be further separated by the lubricant because of the hydrodynamic effect in terms of liquid

lubrication theory[30]. Thus, the output charge decreases slightly at a high sliding frequency (Supplementary Fig. S43b). In this case, all compared electric performances are carried out at the same frequency in the study to eliminate non-triboelectric contributions.

Changes as follows:

Page 19

The charge leakage behavior of the TENG is carried out as shown in Supplementary Fig. S39. The dissipation of the charge decay is very low as similar to previous work[33]. After 2500 s, the residual charges on the surface are 99% of the original charges because the charged surface is fully packaged by the hexadecane, so that the air breakdown and leakage are prevented successfully[34]. In addition, the dielectric leakage current is as low as 1 pA with a bias voltage from 200 V to 1000 V (Supplementary Fig. S40)[35]. It should be noted that the leakage current of the TENG after running-in is consistent with that of a virgin PI-based sample, which means the running-in process does not influence the charge decay and leakage.

Page 20

On the other hand, the change in the device operation, i.e., sliding frequency, can directly affect the electric output of TENGs according to the second part of the equation from the first principle theory[25]. With the increase of sliding frequency, the output current increases obviously (Supplementary Fig. S43a), meaning the value of output current is proportional to the frequency, which aligns with previous works[23, 26]. However, the electric interface between the Al tribolayer and the PI surface will be further separated by the lubricant because of the hydrodynamic effect in terms of liquid lubrication theory[30]. Thus, the output charge decreases slightly at a high sliding frequency (Supplementary Fig. S43b). In this case, all compared electric performances are carried out at the same environmental condition and device operation.

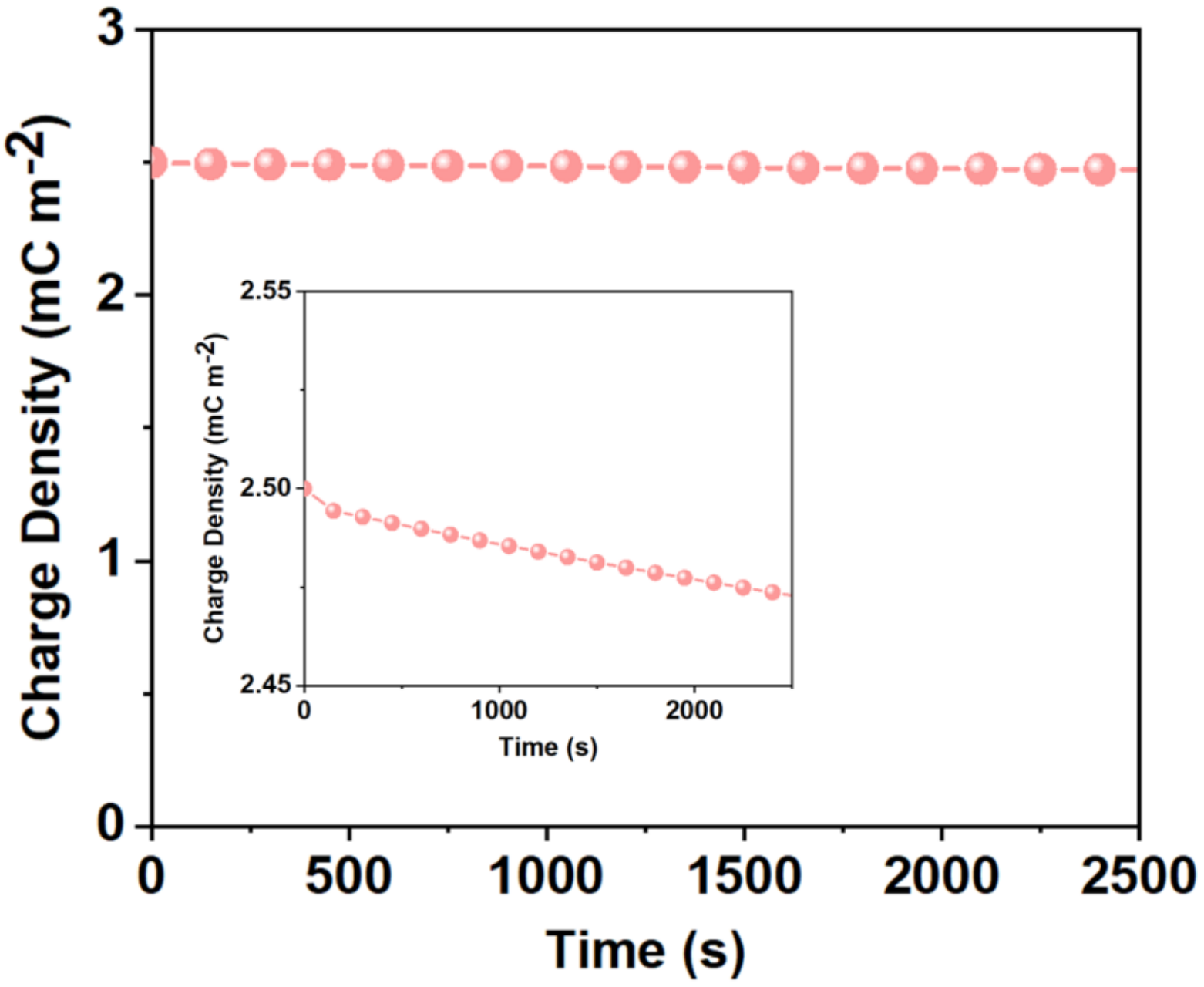

Figure S39 Charge decay of the TENG with the lubrication of hexadecane after running-in with DMSO

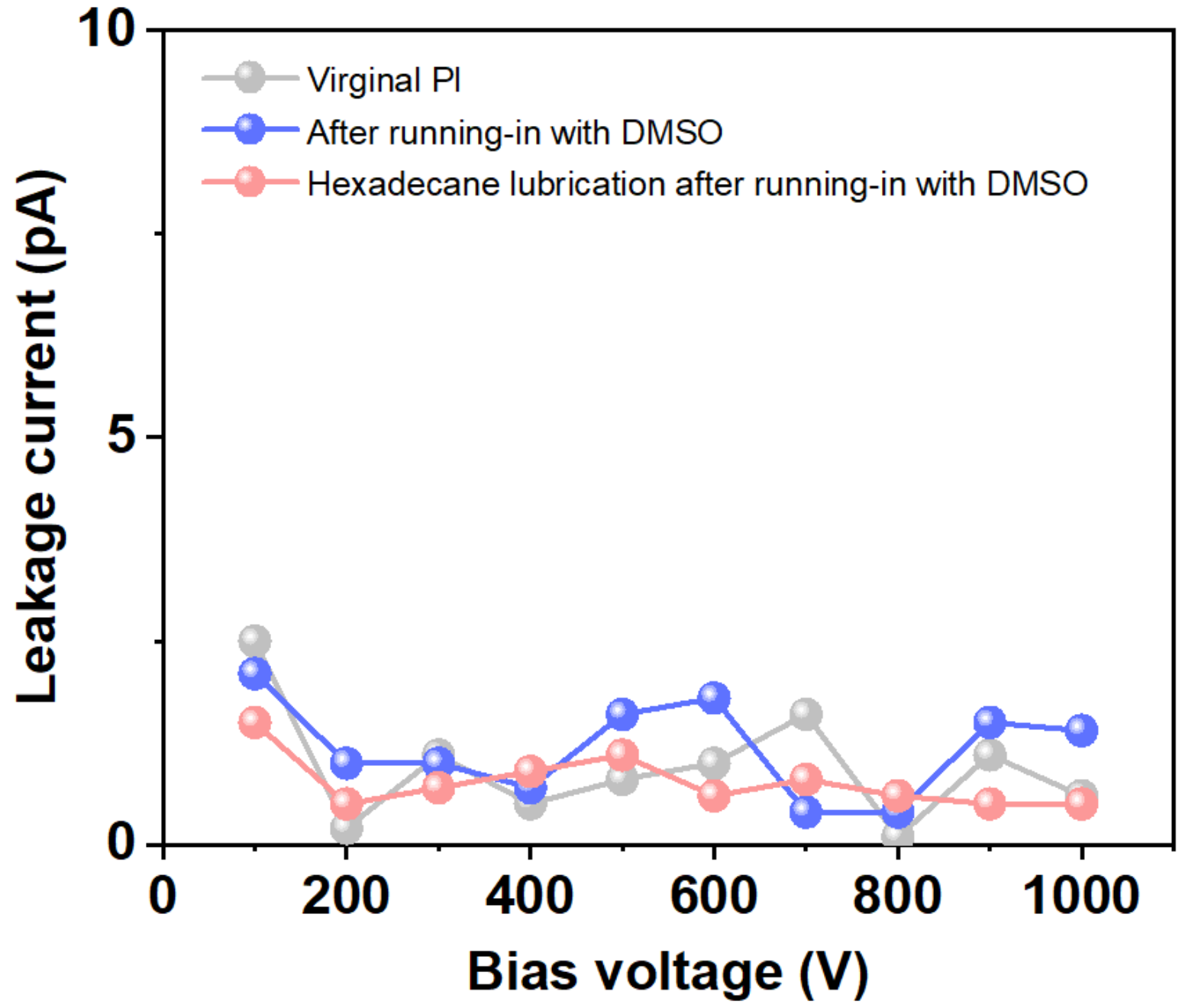

Figure S40 Leakage current of the PIs under different bias voltages

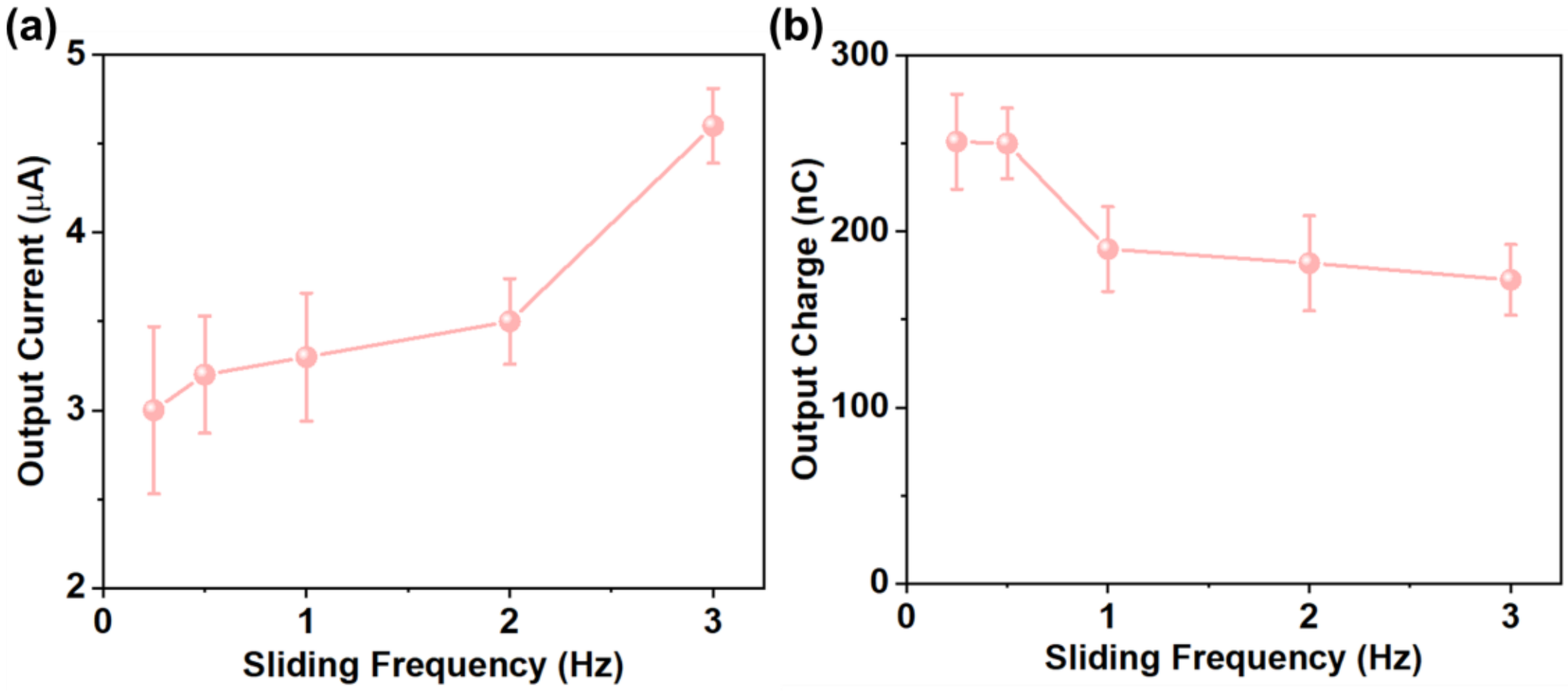

Figure S43 Output current (a) and charge (b) of the TENG lubricated by hexadecane after running-in with DMSO as a function of sliding frequency.

9. Aluminum Surface Representation:

Both MD and DFT simulations model bare aluminum surfaces, while experiments inevitably involve native Al_2O_3 layers and tribocorrosion. The relevance of pure Al to the actual interfacial chemistry is questionable. The authors are encouraged to simulate Al_2O_3 surfaces or discuss the limitations of neglecting this oxide layer.

9. Response: We sincerely appreciate the constructive recommendation. We acknowledge that aluminum surfaces under ambient conditions are easily covered by a native Al_2O_3 layer and that tribocorrosion may further modify the interface. After running-in process, the surface dissolvents, e.g., DMSO, are fully cleaned before the triboelectric period (Fig. 1b). Induced oxide matters are also worn and removed out during the cleaning process. Several recent studies have shown that severe sliding and friction-induced heating can locally disrupt or thin the oxide layer, transiently exposing metallic Al sites that actively participate in interfacial bond activation[39, 40].

Modeling bare Al surfaces is therefore commonly used as a limiting case to capture the fundamental interfacial reactivity. In the current preliminary calculations, we have indeed simplified the model by not explicitly introducing the natural oxide layer (Al_2O_3) on the aluminum surface. This simplification is based on the core scientific objective of this stage of research: to clarify the charge transfer mechanism between specific

functional groups of polyimide (PI) molecular chains and the pure aluminum surface at the atomic scale, at the moment of bond breaking. Our calculations aim to reveal the intrinsic electronic behavior of this initial reaction interface. If an amorphous Al_2O_3 layer with uncertainties in structure, thickness, and defect density is pre-introduced into the model, the complex electronic states of this passivation layer itself will strongly couple with the states of PI, making it difficult for us to clearly distinguish and attribute the source of charge transfer. Therefore, the current use of the pure aluminum surface model is a purposeful and strategic simplification. It allows us to establish a direct correlation between charge transfer and specific bond breaking processes under controllable conditions. While the presence of an Al_2O_3 layer may alter the quantitative strength of adsorption or charge transfer, it is not expected to qualitatively change the identified preferential N–C dissociation pathway in the PI backbone, which is intrinsic to the polymer chemistry. Incorporation of explicit oxide layers will be considered in future work.

10. Solvent–Polymer Interaction Mechanism Unclear:

The proposed mechanism of targeted N–C scission and solvent extraction lacks rigorous evidence. The authors must provide details of the ReaxFF parameterization, validate fragmentation pathways (e.g., energy distribution, scission probability), and compare with DFT-derived reaction barriers. Sensitivity to time step and thermostat parameters should also be reported.

10. Response: Thank you for the insightful comment. The proposed mechanism of friction-induced N-C bond scission and subsequent solvent extraction is primarily inferred from the time-resolved structural evolution observed in the ReaxFF-MD trajectories, rather than derived from a single predefined reaction pathway. Given the highly complex and multi-step nature of the polymer pyrolysis and frictional dissociation processes, it is impractical to capture every intermediate with quantitative reaction energetics in a single simulation. Instead, we analyzed the ReaxFF snapshots and bond-order evolution throughout the simulation to identify the most frequently

ruptured bonds and the resulting chemical species. ReaxFF-MD trajectory, bond scission event predominantly involves N–C bonds located adjacent to the imide linkages, leading to the formation of amine- and carbonyl-terminated fragments at the polymer surface, which is confirmed according to the TOF-SIMS results (Fig. 3h-j). In contrast, cleavages of C–C and C–O bonds are only occasionally observed within the simulated time window. This consistent pattern across independent simulations supports the proposed selective N-C dissociation mechanism. Although a full reaction-energy analysis is beyond the present work's scope, this mechanistic interpretation is physically reasonable and aligns with reported DFT results in the literature[41, 42], where imide N-C bonds in aromatic polymers exhibit lower dissociation barriers (~3.0-3.5 eV) compared with C-C bonds (~3.8-4.0 eV). Therefore, we believe the ReaxFF trajectories provide a reliable qualitative basis for elucidating the dominant dissociation and solvent–polymer interaction mechanisms under frictional conditions.

In addition, to ensure numerical stability of the reactive MD simulations, a small time step of 0.1 fs is adopted to accurately capture bond-breaking and formation. The system temperature is controlled using the Berendsen thermostat with a damping constant of 100 fs.

Changes as follows:

Page 13

The bond scission events predominantly involve N–C bonds located adjacent to the imide linkages, leading to the formation of amine- and carbonyl-terminated fragments at the polymer surface, which is confirmed according to the TOF-SIMS results (Fig. 3h-j). In contrast, cleavages of C–C and C–O bonds are only occasionally observed within the simulated time window. This consistent pattern across independent simulations supports the proposed selective N-C dissociation mechanism. It is physically reasonable and aligns with reported DFT results in the literature[41, 42], where imide N-C bonds in aromatic polymers exhibit lower dissociation barriers (~3.0-3.5 eV) compared with C-C bonds (~3.8-4.0 eV). Therefore, it is believed that the ReaxFF

trajectories provide a reliable qualitative basis for elucidating the dominant dissociation and solvent–polymer interaction mechanisms under frictional conditions.

Page 27

To ensure numerical stability of the reactive MD simulations, a small time step of 0.1 fs is adopted to accurately capture bond-breaking and formation. The system temperature is controlled using the Berendsen thermostat with a damping constant of 100 fs.

11. Lack of Detail in FEM Modeling:

The finite element model references fractal surfaces and Johnson–Cook plasticity, but no parameter values, mesh independence, or validation (e.g., via profilometry) are provided. A power spectral density (PSD) of the roughness profiles, mechanical parameters (e.g., yield modulus), and uncertainty analysis on contact area are essential for credibility.

11. Response: Thanks so much for this constructive comment regarding the details of our finite element modeling approach. We appreciate the opportunity to clarify and expand upon our methodology. In the revised manuscript, we have expanded in the **Method** part to include more explicit parameter values. The rough surfaces are Gaussian isotropic fractal surfaces generated by Fast Fourier transform with given power spectral density, characterized by cut-off wavenumbers k_l, k_r, and k_s. The PSD slope is -2(H+1), where H is the Hurst exponent (H=3-D). Three characteristic parameters impact surface topography: root-mean-square slope $(m_{02}+m_{20})^{1/2}$, Hurst exponent H, and wavelength ratio λ_l/λ_s. The Nayak parameter $\alpha=m_{00}\cdot m_{40}/m_{20}^2$, which characterizes the breadth of the surface spectrum, is provided for each case, where m_{ij} (i, j=0, 2, 4) represents the surface spectral moments and $m_{02}=m_{20}=m_2$ for isotropic rough surfaces.

Concerning mesh independence, our finite element model was verified with three mesh densities of 256, 512, and 1024 elements per direction based on previous work

shown in Fig. R3[43]. The real contact area shows a maximum difference of only 3.11% under different mesh densities, while the ABAQUS output (CAREA) shows 8.81%. For the finest mesh with m = 1024, the difference between the two methods is only 2.66%. In our study, the mesh density of m = 1024 was adopted for all calculations to ensure solution accuracy.

[REDACTED]

Figure R3. Verification of mesh independence of IPM and comparison with CAREA[43].

For validation, the finite element results of real contact area fraction versus dimensionless normal pressure have been compared against established solutions from the literature using identical rough surface parameters $(m_{02}+ m_{20})^{0.5}=0.1$, H=0.8, $\lambda_l/\lambda_s=4$, $L/\lambda_l=8$)[43]. Good agreement was achieved with the boundary element method results by Yastrebov et al.[44, 45], who applied periodic boundary conditions. Our results are also reasonably placed within the range of results given by Couto et al.[46], who used segment-based and node-based methods to calculate the real contact area.

The mechanical parameters for the Johnson-Cook constitutive model are based on aluminum, with the following values now explicitly stated in the revised methods

section: initial yield stress A=324 MPa, hardening constant B=114 MPa, strain hardening exponent n=0.42, and strain rate constant C=0.002. The Young's modulus is E=70 GPa and Poisson's ratio is ν=0.33. The dimensionless yield strength Y/E ranges from 0.002 to 0.01, covering the parametric study requirements. The contact is solved using the penalty function method with appropriate penalty stiffness scaling.

To address uncertainty, we performed five realizations for each loading case under the deterministic power spectral density to account for the randomness inherent in the generated rough surfaces. This approach ensures the reliability and statistical significance of our results. We have revised the manuscript to more clearly present these methodological details and the associated parameter values.

Changes as follow:

Page 24

The PSD slope is -2(H+1), where H is the Hurst exponent. Three characteristic parameters impact surface topography: root-mean-square slope $(m_{02}+m_{20})^{1/2}$, Hurst exponent H, and wavelength ratio λ_l/λ_s. The Nayak parameter $\alpha=m_{00}\cdot m_{40}/m_{20}^2$, which characterizes the breadth of the surface spectrum, is provided for each case, where m_{ij} (i, j = 0, 2, 4) represents the surface spectral moments. For validation, the finite element results of real contact area fraction versus dimensionless normal pressure have been compared against established solutions from the literature using identical rough surface parameters ($(m_{02}+m_{20})^{0.5}=0.1$, H=0.8, $\lambda_l/\lambda_s=4$, $L/\lambda_l=8$)[43]. Good agreement was achieved with the boundary element method results by Yastrebov et al.[44, 45], who applied periodic boundary conditions. Our results are also reasonably placed within the range of results given by Couto et al.[46], who used segment-based and node-based methods to calculate the real contact area. Concerning mesh independence, the finite element model was verified with three mesh densities of 256, 512, and 1024 elements per direction based on previous work[43]. The real contact area shows a maximum difference of only 3.11% under different mesh densities, while the ABAQUS output (CAREA) shows 8.81%. For the finest mesh with m=1024, the difference between the two methods is only 2.66%.

In this study, the mesh density of m=1024 was adopted for all calculations to ensure solution accuracy.

The mechanical parameters for the Johnson-Cook constitutive model are based on aluminum, with the following values now explicitly stated in the revised methods section: initial yield stress A=324 MPa, hardening constant B=114 MPa, strain hardening exponent n=0.42, and strain rate constant C=0.002. The Young's modulus is E=70 GPa and Poisson's ratio is ν=0.33. The dimensionless yield strength Y/E ranges from 0.002 to 0.01, covering the parametric study requirements. The contact is solved using the penalty function method with appropriate penalty stiffness scaling. To address uncertainty, five realizations are performed for each loading case under the deterministic power spectral density to account for the randomness inherent in the generated rough surfaces. This approach ensures the reliability and statistical significance of the results.

12. Electrostatics and Boundary Conditions in MD:

Simulations involving vacuum interfaces and long-range electrostatics (PPPM) should include corrections (e.g., Yeh–Berkowitz, non-periodic z-direction) to prevent spurious dipole interactions. Failure to do so may compromise RDFs and interfacial behavior predictions.

12. Response: We sincerely appreciate the insightful comment. In our classical MD simulations, the long-range electrostatics were treated using the particle–particle particle-mesh (PPPM) method. To minimize artifacts from periodic images, a sufficient vacuum layer was introduced above the polymer-solvent interface, effectively reducing direct interactions between mirror cells. Furthermore, for systems involving vacuum interfaces along the z-direction, we applied a slab correction to eliminate spurious dipole–dipole coupling caused by 3D periodic boundary conditions. Specifically, the kspace_modify slab command in LAMMPS was employed, which introduces an additional empty space along the z-axis and removes electrostatic interactions between replicated dipole layers. This setup ensures that the system maintains periodicity in the

x–y plane while remaining non-periodic in the z-direction, providing a more accurate representation of interfacial electrostatics and reliable RDF and structural analyses.

13. DFT: Solvent Effects Not Captured:

The DFT modeling assumes vacuum interfaces, despite the key role of DMSO in the hypothesized mechanism. The authors should incorporate solvent effects explicitly (e.g., via VASPsol or explicit DMSO layers) and recompute key parameters such as LUMO localization, charge transfer, and interfacial dipole moments.

13. Response: Thanks so much for this constructive comment. There are two key periods for achieving high electric outputs in this study, i.e., the running-in period with DMSO assistance, and the triboelectric period with the hexadecane lubrication. During the running-in period, friction induces the breakage of the relatively unstable N-C bonds in PI, resulting in the freedom to release amide groups. With the help of DMSO-driven extraction, the chemical chains containing amide groups are regulated to be "molecular brushes" outspread towards contact surfaces, among which the C=O bonds with strong electron-withdrawing capability are thus exposed and capture electrons from the contacting electrode. The mechanism of the above process underlying the DMSO solvent-treatment is elucidated comprehensively by molecular dynamics (MD) simulations, shown in Fig. 4.

The DFT modeling is thus introduced to demonstrate the atom-level triboelectricity of the PI with exposed C=O bonds in the triboelectric period under the lubrication of hexadecane. In this period, there are few DMSO components at the electric interface, which is because the electric surface is fully cleaned with acetone after the running-in process (Fig. 1). It should be noted that the DFT modeling assumes vacuum interfaces in the previous manuscript, which ignores the hexadecane lubricant at the electric interface between the PI molecule and the Al surface. To address this issue, in the revised manuscript, an implicit solvation model was employed to account for solvent effects of hexadecane, with a dielectric constant of 2.05 used to describe the solvent environment[47] (Density functional theory (DFT) in the part of **Method**). Thus, we have incorporated the hexadecane lubricant effect explicitly through VASPsol and

recompute key triboelectric parameters in the revised manuscript. Shown in Fig. R4 and Fig. 4i, considering the solvent effect of hexadecane, the transferring electrons are a little lower than those without considering the solvent effect.

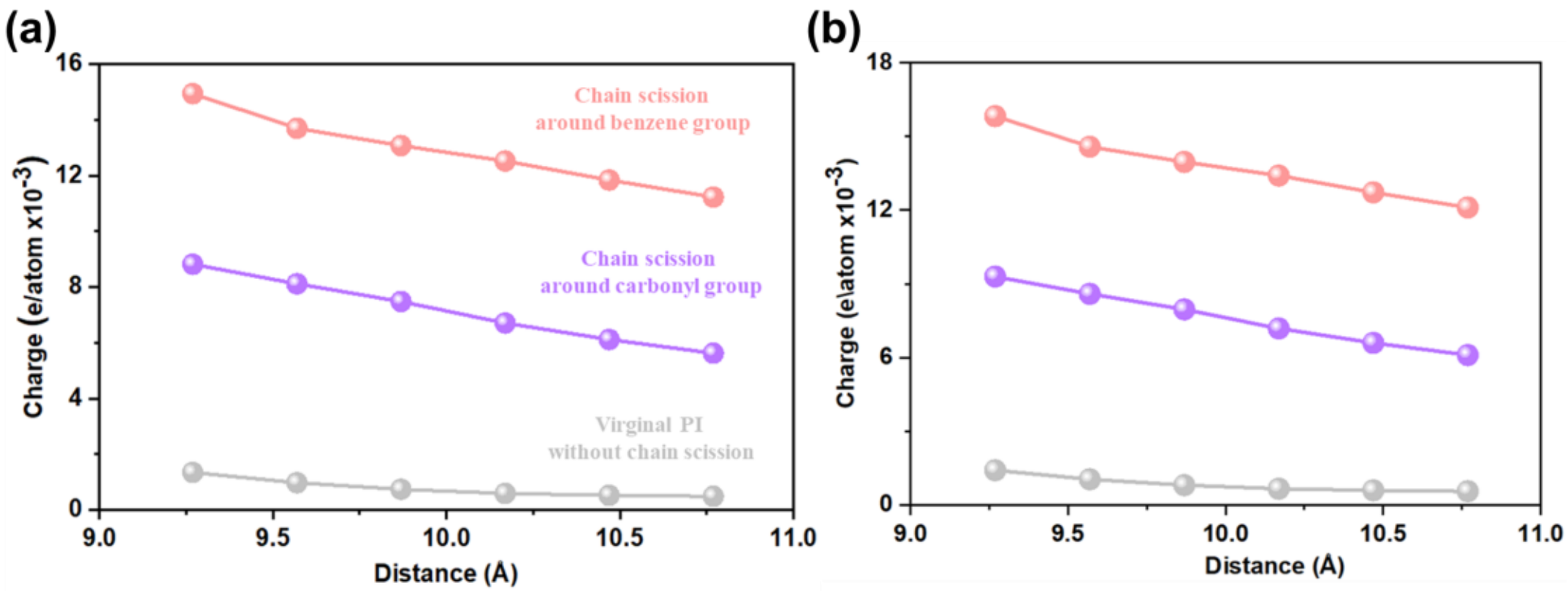

Figure R4 Average number of electrons obtained by each atom of PIs via DFT considering the solvent effect of hexadecane (a) and without considering the solvent effect (b).

Changes as follows:

Page 14

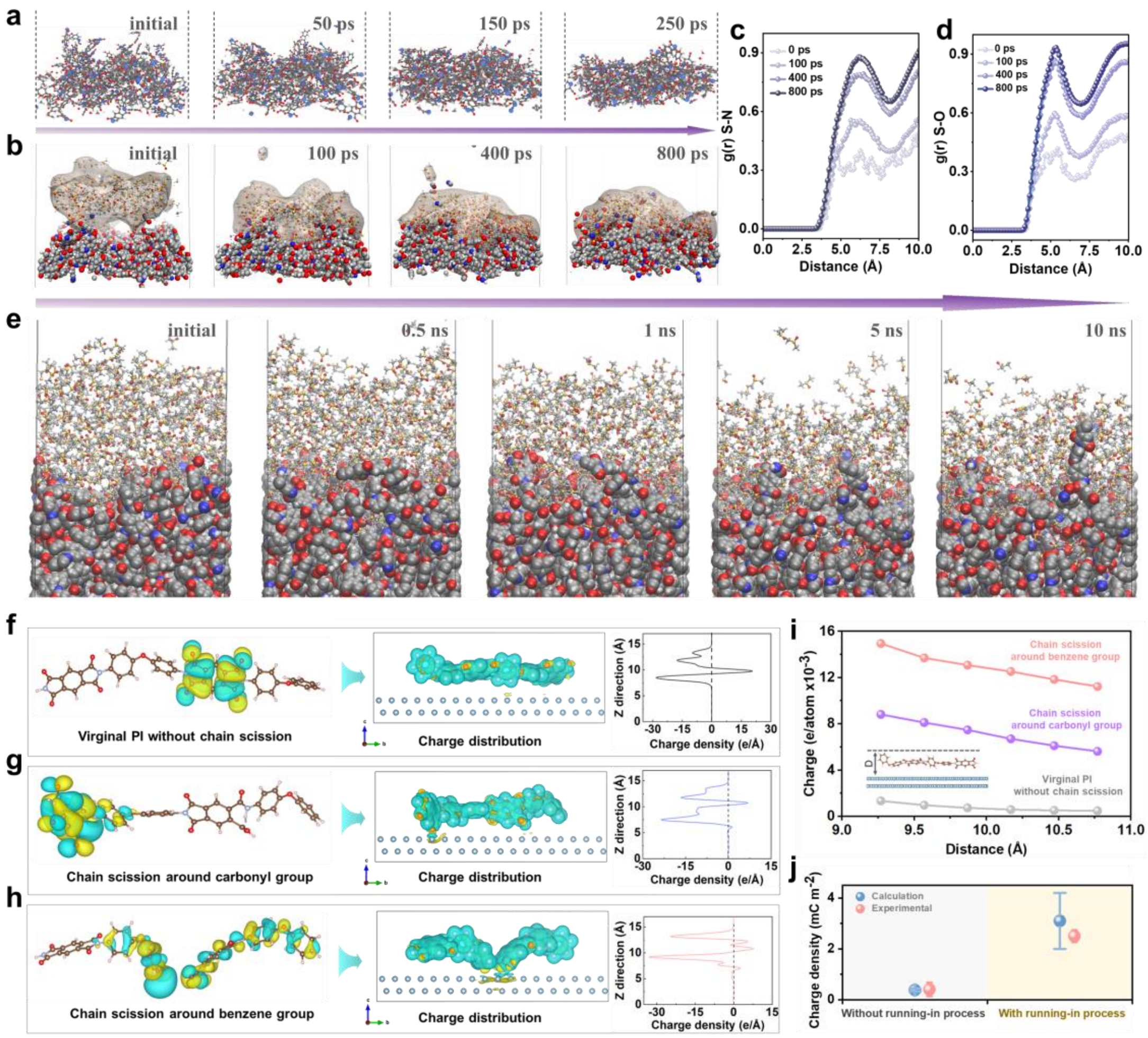

Fig. 4 The triboelectric mechanism of PI with DMSO-assisted running-in process achieved via MD and DFT simulation. a Relaxation process of the PI bulk phase after friction with Al performed by classical MD. **b** Time-dependent dynamics of DMSO molecules infiltrating into the running-in PI. **c** and **d** Radial distribution function between the S atom of DMSO and the N, O atom in the running-in PI. **e** Time-dependent MD analysis of the extraction effect of DMSO molecules on the broken chemical chains, e.g., amide groups, on the surface of the running-in PI. **f, g,** and **h** Lowest unoccupied molecular orbital (LUMO), charge distribution, and 2D charge density difference of PIs. **i** Average number of electrons obtained by each atom of PIs. Inset demonstrating the distance between the top of the molecules and the contact interface. J Charge densities from the calculations according to Figure 4i and from the experimental through lubrication without running-in process or lubrication after running-in with DMSO according to Figure 2b.

Changes as follow:

Page 15

For example, at a distance of 9.3 Å, the transferred electron of the running-in PI with the broken C-N bonds around carbonyl groups is 0.0149 e/atom, while the amount value is 0.0088 e/atom for the sample with the fracture of the C-N bond around benzenes. Accordingly, there is an average one order of magnitude higher amount of transferred charges for running-in PIs with the N-related chain scission in contrast to the virgin PI. Herein, it is greatly expected to obtain the charge density of PI regarding the DFT analysis. Accordingly, the charge density of PI is 3.46~ 4.2 mC m^{-2} based on the transferred charge of PI (**Methods**). It should be noted that the interface of PI/Al is hypothetical at a full contact state (ratio of contact area~100%). Considering the practical contact in the Al/PI interface with a real ratio of contact area of about 10% (Fig. 3b), i.e., 90% of interfacial atoms cannot contribute to charge transfer. Accordingly, as shown in Figure 4j, the calculated charge density (0.35~0.42 mC m^{-2}) is thus similar to the experimental result of PI (average ~0.4 mC m^{-2}) through adding lubricants of hexadecane, squalane, dodecane, and paraffin oil without running-in process (Fig. 2b). For the case of running-in with DMSO, the calculated value of charge density ranges from 2.31 mC m^{-2} to 3.93 mC m^{-2} regarding the transferred electron at the assumed condition of broken C-N bonds (Fig. 4i), meaning the value range contains the experimental charge density (2.5 mC m^{-2}) of PI lubricated by hexadecane after running-in with DMSO (Fig. 2a). The DFT results identify the attractive optimizing method for the triboelectric enhancement, and also agree with the experimental results very well (Fig. 4j).

Page 27

An implicit solvation model was employed to account for solvent effects of hexadecane, with a dielectric constant of 2.05 used to describe the solvent environment.

References

1. Erdemir, A. et al. Carbon-based tribofilms from lubricating oils. *Nature* **536**, 67-+ (2016).
2. Iwai, Y. et al. Quantitative estimation of wear amounts by real time measurement of wear debris in lubricating oil. *Tribology International* **43**, 388-394 (2010).
3. Wu, J., Xi, Y.H. & Shi, Y.J. Toward wear-resistive, highly durable and high performance triboelectric nanogenerator through interface liquid lubrication. *Nano Energy* **72**, 9 (2020).
4. Zhou, L.L. et al. Simultaneously Enhancing Power Density and Durability of Sliding-Mode Triboelectric Nanogenerator via Interface Liquid Lubrication. *Advanced Energy Materials* **10**, 9 (2020).
5. Li, Y. et al. Advanced Dielectric Materials for Triboelectric Nanogenerators: Principles, Methods, and Applications. *Adv. Mater.* **36**, 53 (2024).
6. Zhao, K. et al. Layer-Engineered Hybrid MoS2 Nanosheets for Synergistic Charge Trapping and Superlubrication toward High-Output Ultra-Durable Triboelectric Nanogenerators. *Adv. Funct. Mater.*, 16 (2025).
7. Xiao, K. et al. Improving Triboelectric Nanogenerators Performance Via Interface Tribological Optimization: A Review. *Adv. Funct. Mater.* **34**, 38 (2024).
8. Heo, D. et al. Nano-Oil-Barrier-Based Fluttering Triboelectric Nanogenerator. *Adv. Sci.* **12**, 10 (2025).
9. Sun, L.L., Lin, S.Q., Tang, W., Chen, X. & Wang, Z.L. Effect of Redox Atmosphere on Contact Electrification of Polymers. *Acs Nano* **14**, 17354-17364 (2020).
10. Li, W.T. et al. Adsorption-induced surface potential modulation and electron transfer of solid-solid triboelectric nanogenerators under gas atmospheres. *Nano Energy* **143**, 12 (2025).

11. Merlone, A. et al. Temperature extreme records: World Meteorological Organization metrological and meteorological evaluation of the 54.0°C observations in Mitribah, Kuwait and Turbat, Pakistan in 2016/2017. *Int. J. Climatol.* **39**, 5154-5169 (2019).
12. Zhao, J. et al. Real-Time and Online Lubricating Oil Condition Monitoring Enabled by Triboelectric Nanogenerator. *Acs Nano* **15**, 11869-11879 (2021).
13. Yoo, D., Jang, S., Cho, S., Choi, D. & Kim, D.S. A Liquid Triboelectric Series. *Adv. Mater.* **35**, 11 (2023).
14. Busolo, T. et al. Surface potential tailoring of PMMA fibers by electrospinning for enhanced triboelectric performance. *Nano Energy* **57**, 500-506 (2019).
15. Fumagalli, L., Gramse, G., Esteban-Ferrer, D., Edwards, M.A. & Gomila, G. Quantifying the dielectric constant of thick insulators using electrostatic force microscopy. *Appl. Phys. Lett.* **96**, 3 (2010).
16. Fumagalli, L., Ferrari, G., Sampietro, M. & Gomila, G. Quantitative Nanoscale Dielectric Microscopy of Single-Layer Supported Biomembranes. *Nano Lett.* **9**, 1604-1608 (2009).
17. Fumagalli, L., Ferrari, G., Sampietro, M. & Gomila, G. Dielectric-constant measurement of thin insulating films at low frequency by nanoscale capacitance microscopy. *Appl. Phys. Lett.* **91**, 3 (2007).
18. Houssat, M., Villeneuve-Faure, C., Dignat, N.L. & Cambronne, J.P. Nanoscale mechanical and electrical characterization of the interphase in polyimide/silicon nitride nanocomposites. *Nanotechnology* **32**, 13 (2021).
19. Qi, G.C. et al. Quantifying Surface Charge Density by Using an Electric Force Microscope with a Referential Structure. *J.Phys. Chem. C* **113**, 204-207 (2009).
20. Qi, G.C. et al. Characteristic capacitance in an electric force microscope determined by using sample surface bias effect. *J. Appl. Phys.* **103**, 4 (2008).
21. Li, D.A., Wang, X., Mo, X.Y., Tse, E.C.M. & Cui, X.D. Electronic gap characterization at mesoscopic scale via scanning probe microscopy under ambient conditions. *Nat. Commun.* **13**, 7 (2022).
22. Yang, P. et al. Radical anion transfer during contact electrification and its

compensation for charge loss in triboelectric nanogenerator. *Matter* **6**, 18 (2023).

23. He, W.C. et al. Boosting output performance of sliding mode triboelectric nanogenerator by charge space-accumulation effect. *Nat. Commun.* **11**, 8 (2020).
24. Wang, H.L., Guo, Z.H., Zhu, G., Pu, X. & Wang, Z.L. Boosting the Power and Lowering the Impedance of Triboelectric Nanogenerators through Manipulating the Permittivity for Wearable Energy Harvesting. *Acs Nano* **15**, 7513-7521 (2021).
25. Wang, Z.L. On the first principle theory of nanogenerators from Maxwell's equations. *Nano Energy* **68**, 10 (2020).
26. Zi, Y.L. et al. Harvesting Low-Frequency (<5 Hz) Irregular Mechanical Energy: A Possible Killer Application of Triboelectric Nanogenerator. *Acs Nano* **10**, 4797-4805 (2016).
27. Niu, S.M. et al. Theoretical study of contact-mode triboelectric nanogenerators as an effective power source. *Energy Environ. Sci.* **6**, 3576-3583 (2013).
28. Luo, Y.J. et al. Advanced energy harvesting from low-frequency ocean waves for lithium-ion battery applications. *Energy Environ. Sci.* **18**, 4821-4832 (2025).
29. Gao, Y.K. et al. Spontaneously established reverse electric field to enhance the performance of triboelectric nanogenerators via improving Coulombic efficiency. *Nat. Commun.* **15**, 11 (2024).
30. Gohar, R. & Cameron, A. Optical Measurement of Oil Film Thickness under Elasto-Hydrodynamic Lubrication. *Nature* **200**, 458-& (1963).
31. Du, Y.Z., Li, J., Wen, N., Zhang, Z. & Song, D. The pyrolysis of polyimide and epoxy resin by the ReaxFF molecular dynamics simulation. *J. Mol. Model.* **31**, 14 (2025).
32. Lu, X. et al. A ReaxFF-based molecular dynamics study of the pyrolysis mechanism of polyimide. *Polym. Degrad. Stabil.* **114**, 72-80 (2015).
33. Wang, J. et al. Remarkably Enhanced Charge Density of Inorganic Material Via Regulating Contact Barrier Difference and Charge Trapping for Triboelectric Nanogenerator. *Adv. Funct. Mater.* **33**, 11 (2023).
34. Chung, S.H. et al. Nonpolar Liquid Lubricant Submerged Triboelectric

Nanogenerator for Current Amplification via Direct Electron Flow. *Advanced Energy Materials* **11**, 8 (2021).

35. Wang, J. et al. Boosting the Charge Density of Triboelectric Nanogenerator by Suppressing Air Breakdown and Dielectric Charge Leakage. *Adv. Energy Mater.* **14**, 11 (2024).
36. Park, H.W. et al. Electron blocking layer-based interfacial design for highly-enhanced triboelectric nanogenerators. *Nano Energy* **50**, 9-15 (2018).
37. Firdous, I., Fahim, M. & Daoud, W.A. Performance enhancement of triboelectric nanogenerator through hole and electron blocking layers-based interfacial design. *Nano Energy* **82**, 10 (2021).
38. Jiang, H.X. et al. Charge-trapping-blocking layer for enhanced triboelectric nanogenerators. *Nano Energy* **75**, 7 (2020).
39. Zhang, Z.Y., Dandu, R.S.B., Klu, E.E. & Cai, W.J. A Review on Tribocorrosion Behavior of Aluminum Alloys: From Fundamental Mechanisms to Alloy Design Strategies. *Corros. Mater. Degrad.* **4**, 594-622 (2023).
40. Jimenez, A.G. et al. Oxide tribolayer breakdown on sliding metal contacts drives thermal ignition. *Tribol. Int.* **205**, 14 (2025).
41. Zhou, S.R., Zhang, L., Zou, L., Ayubi, B.I. & Wang, Y.W. Mechanistic Study of Atomic Oxygen Erosion on Polyimide Under Electric Fields: A Molecular Dynamics and Density Functional Theory Approach. *Molecules* **29**, 11 (2024).
42. Marochkin, II & Dorofeeva, O.V. Amide bond dissociation enthalpies: Effect of substitution on N-C bond strength. *Comput. Theor. Chem.* **991**, 182-191 (2012).
43. Zhang, H.B., Liu, S.L. & Wang, W.Z. On contact spots details of rough surface contact using morphologic image processing. *Mech. Mach. Theory* **192**, 21 (2024).
44. Yastrebov, V.A., Anciaux, G. & Molinari, J.F. The role of the roughness spectral breadth in elastic contact of rough surfaces. *J. Mech. Phys. Solids* **107**, 469-493 (2017).
45. Yastrebov, V.A., Anciaux, G. & Molinari, J.F. On the accurate computation of the true contact-area in mechanical contact of random rough surfaces. *Tribol.*

Int. **114**, 161-171 (2017).

46. Carneiro, A.M.C., Carvalho, R.P. & Pires, F.M.A. Representative contact element size determination for micromechanical contact analysis of self-affine topographies. *Int. J. Solids Struct.* **206**, 262-281 (2020).

47. Kamogawa, K. et al. Dispersion and stabilizing effects of n-hexadecane on tetralin and benzene metastable droplets in surfactant-free conditions. *Langmuir* **15**, 1913-1917 (1999).

A point-by-point response to the reviewers' comments in this stage (in principle to publish)

Reviewer #1: The reviewer recognizes that significant effort has been made to revise the manuscript, and that many aspects have been strengthened through additional experiments, analyses, and clarifications. These revisions have clearly improved the overall quality of the work. Nevertheless, despite these improvements, the manuscript still does not achieve the level of novelty and broad impact expected for publication in Nature Communications. The reported advances, while technically solid, remain incremental and are unlikely to substantially change current understanding or paradigms in the field.

Response: Thanks so much for your comments. In this study, a novel solvent-assisted running-in strategy is proposed to improve the triboelectric nanogenerator output. The improved charge density reaches 2.5 mC m^{-2}, which is a new record. This strategy also provides valuable insights for the study on triboelectric interfaces at the molecular and electronic levels. Especially, solvent-assisted running-in strategy in this study enables improved electrical output by leveraging engineering polymers without material modification, and provides valuable insights for the development of triboelectric nanogenerators. Thanks again.

Reviewer #2: During the revision, the authors have asked to revise the manuscript with additional experiments and scientific discussions. The authors have addressed all the comments with the point by point response. The present version of the manuscript is suitable for publication.

Response: Thanks so much for your valuable comments.

Reviewer #3: I recommend the revised manuscript for publication in Nature Communications.

Response: Thanks so much for your valuable comments.

Reviewer #4: The authors have adequately addressed the reviewers' comments with appropriate scientific justification. The manuscript is recommended for publication in Nature Communication.

Response: Thanks so much for your valuable comments.
